# Molecular Tools for the Detection of Nitrogen Cycling Archaea

**DOI:** 10.1155/2013/676450

**Published:** 2013-01-08

**Authors:** Antje Rusch

**Affiliations:** Department of Microbiology and Center for Ecology, Southern Illinois University Carbondale, 1125 Lincoln Drive, Carbondale, IL 62901, USA

## Abstract

Archaea are widespread in extreme and temperate environments, and cultured representatives cover a broad spectrum of metabolic capacities, which sets them up for potentially major roles in the biogeochemistry of their ecosystems. The detection, characterization, and quantification of archaeal functions in mixed communities require Archaea-specific primers or probes for the corresponding metabolic genes. Five pairs of degenerate primers were designed to target archaeal genes encoding key enzymes of nitrogen cycling: nitrite reductases NirA and NirB, nitrous oxide reductase (NosZ), nitrogenase reductase (NifH), and nitrate reductases NapA/NarG. Sensitivity towards their archaeal target gene, phylogenetic specificity, and gene specificity were evaluated in silico and in vitro. Owing to their moderate sensitivity/coverage, the novel *nirB*-targeted primers are suitable for pure culture studies only. The *nirA*-targeted primers showed sufficient sensitivity and phylogenetic specificity, but poor gene specificity. The primers designed for amplification of archaeal *nosZ* performed well in all 3 criteria; their discrimination against bacterial homologs appears to be weakened when Archaea are strongly outnumbered by bacteria in a mixed community. The novel *nifH*-targeted primers showed high sensitivity and gene specificity, but failed to discriminate against bacterial homologs. Despite limitations, 4 of the new primer pairs are suitable tools in several molecular methods applied in archaeal ecology.

## 1. Introduction

Archaea have been detected in virtually all types of extreme and moderate environments. They play multiple ecological roles, colonizing certain newly emerging habitats [1, 2], interacting with animals such as corals [3, 4], sponges [5, 6], termites [7], or ruminants, forming part of microbe-microbe symbioses [8–10], and driving numerous processes in the biogeochemical C, N, S, and Fe cycles. In addition to relatively well-studied isolates of extremophilic or methanogenic Archaea, uncultured representatives have been detected by their 16S rRNA genes or by metabolic genes that classify their owners into the guilds of sulfate reducers, diazotrophs, ammonia oxidizers, or methanogens. Despite their widespread occurrence, a mere handful of nonmethanogenic Archaea has been isolated from moderate habitats [11–13]. While such isolates are indispensable for insight into archaeal ecophysiology, they have been recalcitrant to cultivation efforts, so that our current ecological research on mesophilic Archaea largely depends on cultivation-independent methods. Molecular tools for the detection, quantification, diversity analysis, and measurement of gene expression in microbial guilds are sparse, however, when it comes to archaeal guild members. The present study addresses the need for such molecular tools, reporting the design and evaluation of primers/probes targeting the archaeal members of 5 guilds in the N cycle.

Processes of the biogeochemical N cycle that are known to be catalyzed by Archaea in extreme or moderate habitats include ammonia oxidation, dissimilatory reduction of nitrate to nitrite and of nitrite to ammonium, denitrification, N_2_ fixation and nitrate assimilation [14–18]. An overview of the processes considered in this paper, their key enzymes, and corresponding metabolic genes are given in Table 1.

The major oxidative pathway of the N cycle, nitrification, consists of the two-step oxidation of ammonium with O_2_ to nitrite and on to nitrate. The first rate-limiting step is mediated by ammonia monooxygenase (Amo), a key enzyme found in a variety of *β*- and *γ*-Proteobacteria [19, 20] as well as 4 cultivated members of the Thaumarchaeota [11, 13] and thermophilic Crenarchaeota [21, 22]. In addition, the encoding marker gene *amoA* has been recovered from archaeal enrichment cultures and numerous marine, freshwater, terrestrial, and engineered systems. A comprehensive review of archaeal ammonia oxidizers has been published recently [23]. Primers for the amplification of archaeal *amoA* genes (Table 1) have been applied successfully over the course of several years. Thaumarchaeal genomes in the curated KEGG and RefSeq databases [24, 25] include *nirA*, *nirD*, and *nirS* genes, that encode ammonifying and denitrifying nitrite reductases. The interesting possibility of reductive N metabolism in this widespread archaeal phylum strongly motivates the design of primers and probes that target specifically archaeal nitrate and nitrite reductase genes.

The reduction of nitrate to nitrite, the initial step of all reductive pathways in the N cycle, is mediated by dissimilatory nitrate reductases. Membrane-bound and periplasmic nitrate reductases (Nar and Nap, resp.,) occur in a wide range of heterotrophic bacteria and Archaea [14, 16, 26, 27]. The relative contribution of archaeal activity to overall nitrate reduction in natural ecosystems has not been quantified yet. Primer sets suitable for the amplification of marker genes *narG* and *napA* from archaeal nitrate reducers appear to be missing.

Further reduction of nitrite occurs via dissimilatory nitrate/nitrite reduction to ammonium (DNRA) or via denitrification to gaseous N compounds. Which pathway dominates may depend on the ecosystem under consideration and the ratio of electron donors and acceptors available [28]. The diverse guild of DNRA-mediating organisms comprises numerous bacteria and fungi as well as several thermophilic and halophilic Archaea. DNRA is catalyzed by the ammonifying nitrite reductases Nrf, of which no archaeal homologs are known, and NirA and NirB (Table 1). Although a small number of archaeal *nirA* and *nirB* sequences exists in nucleotide databases, no published primer sets for the detection of these marker genes are available.

Denitrification, an intensely studied process due to its relevance in agriculture, wastewater treatment, and greenhouse gases, consists of up to 3 steps (nitrite → NO → N_2_O → N_2_), depending on the presence and expression of the corresponding metabolic genes in the catalyzing organisms. The guild of denitrifiers includes members of over 60 bacterial and archaeal genera [29]. Key enzymes of this process are the nitrite reductases NirK and NirS, nitric oxide reductase (NorB), and nitrous oxide reductase (Nos), all of which are found in both bacterial and archaeal denitrifiers [14, 16, 30, 31]. Several primer pairs targeting the bacterial *nirK*, *nirS*, *norB* and *nosZ* genes have been designed and applied [32, 33], while among their archaeal homologs only *nirK* has been addressed with primers [31]. As few as 26 archaeal species are known to possess marker genes of denitrification [25]. Detection methods are currently limited to the observation of denitrifying activity in pure culture and the annotation of sequenced genomes. The availability of suitable primers and probes could greatly promote our research into the diversity, abundance, and activity of denitrifying Archaea.

The process of N_2_ fixation (Table 1), which strongly enhances N bioavailability, is particularly important in N-limited natural or agricultural systems. It is catalyzed by the anaerobic enzyme nitrogenase, which is found widespread among bacteria and methanogenic Archaea [34]. The *nifH* gene, encoding the nitrogenase reductase subunit of this enzyme, is the most sequenced marker gene for diazotrophs [35]. Its sequences form 4 major clusters, with archaeal homologs represented in 3 of them, and many species possess 2 or more *nifH* homologs belonging to different clusters [34]. As many as 42 universal and 19 group-specific primer pairs have been used to target bacterial and archaeal *nifH* [35]. Four of them showed high phylogenetic coverage in silico and good specificity for the target gene in vitro [35], but none was archaea-specific.

In the present study, primer pairs for the sensitive and specific detection of archaeal *nirA*, *nirB*, *nosZ*, *nifH*, and *napA/narG* genes were developed. These novel primers were then evaluated both in silico and in vitro for their performance in regard to (1) sensitivity towards archaeal targets, (2) discrimination against bacterial homologs (phylogenetic specificity), and (3) specificity for their target gene.

## 2. Materials and Methods

### 2.1. Degenerate Primer Design

For each of the target genes, functional orthologs were identified from the curated KEGG database [24], and all available archaeal nucleotide sequences were downloaded. These were 37 *nirA*, 3 *nirB*, 8 *nosZ*, 75 *nifH*, and 18 *napA/narG* sequences (deposited insupplementary file available online at http://dx.doi.org/10.1155/2013/676450). Alignments of orthologous archaeal sequences revealed no conserved regions of sufficient length (18–25 nt) to design primer pairs without degeneracy.

The software tool HYDEN [36] was applied to design gene-specific and archaea-specific primer sets, allowing for a maximum degeneracy of 128 as a tradeoff between sensitivity and specificity. Each primer was set to 20 nt length and up to 2 mismatches (up to 3 for both primers combined) with the target sequence. The archaeal gene sequences were then screened for binding sites of the novel degenerate primer pairs, using the Primersearch program of EMBOSS [37], in order to determine the length of the anticipated PCR products. Amplificate lengths were required to fall within the range of 300–1000 nt, which is considered the most suitable for many routine applications. Primer design was carried out by Higgs Consulting LLC (Gaithersburg, MD, USA).

### 2.2. Primer Evaluation In Silico

The novel primer pairs targeting archaeal metabolic genes were evaluated for sensitivity towards archaeal target sequences and discrimination against bacterial homologs. For each gene, all known or validated archaeal and bacterial reference sequences were obtained from the curated RefSeq nucleotide database [25]. Applying the Geneious Pro software (Biomatters Ltd.), these reference sequences were screened for primer binding sites, allowing for up to 1 mismatch. The percentage of archaeal sequences that can bind both primers in correct orientation was used as a measure of sensitivity, whereas the number of bacterial sequences binding both primers indicated their phylogenetic specificity.

### 2.3. Primer Evaluation In Vitro

#### 2.3.1. Real-Time PCR with Pure Culture Templates

The suitability of the novel primer pairs to amplify their archaeal target genes was assessed by real-time PCR. Genomic DNA from archaeal pure cultures, to be used as template DNA, was obtained from DSMZ (Braunschweig, Germany). Primers were synthesized by Integrated DNA Technologies (Coralville, IA, USA). Amplification reactions of 20 *μ*L volume were catalyzed by Sso7d-fusion polymerase in EvaGreen supermix (Bio-Rad Laboratories), with 0.5 *μ*M of forward and reverse primer and 0–7 ng template DNA. For each archaeal template, a range of DNA concentrations and primer annealing temperatures was tested during optimization of the PCR protocol. Reactions without template were run as negative controls. All amplifications were carried out and monitored by a MiniOpticon real-time thermocycler (Bio-Rad Laboratories). The thermocycler protocol consisted of an enzyme activation step of 3 min at 95°C, followed by 35 cycles of template denaturation (10 s at 98°C), primer annealing (15 s, range of temperatures) and extension (time as specified in Table 2, 60–64°C), then 7 min at 62°C. Subsequent melt curve analysis as well as threshold cycle and product concentration were used as first indicators of successful amplification.

Length and purity of the PCR products were determined by gel electrophoresis, using agarose gels (1.5%) in TAE running buffer exposed to 80 V (<60 mA) for 75–90 min. Gels were stained with Nancy-520 (Sigma-Aldrich, St. Louis, MO, USA) for 1 h and observed under transillumination with blue light. Green fluorescent bands of DNA were compared to a molecular weight marker (Fisher exACTGene 100 bp DNA ladder).

#### 2.3.2. Real-Time PCR with Environmental DNA

The performance of 3 primer sets (targeting *nirA*, *nosZ* and *nifH*) was tested in regard to sensitivity and specificity when applied to DNA from environmental samples. In July and August 2011, anoxic water samples were collected from 24 m depth in the Central Basin of Lake Erie and from 6 m depth in Lost Lake, a strip mine lake in Pyramid State Park, IL. These samples contained 3.2–4.4 *μ*M ammonium and 0.09–0.23 *μ*M nitrite, and while 3.4 *μ*M nitrate were measured in Lake Erie, no nitrate was detected in the strip mine lake (Rusch & Wham, ms in preparation). Up to 4% of DNA in Lake Erie has been identified as archaeal [38, 39], and archaeal cell abundance in Lost Lake ranged between 4% and 7% (Rusch & Wham, ms in preparation). Particulate material >0.2 *μ*m contained in 100 mL lake water was filtered onto polycarbonate membrane filters (Millipore); DNA was extracted using the Power Water DNA Isolation kit (MoBio Laboratories) and stored at −20°C until analysis.

All 22 DNA extracts were tested in triplicate for the presence of *nirA*, *nosZ,* and *nifH*. Real-time PCR as described above was carried out with the corresponding novel primer pairs, applying optimized conditions as specified in Table 2. Melt curve analysis, threshold cycle, and agarose gel electrophoresis as described above were used to evaluate the PCR products.

#### 2.3.3. Amplification, Cloning, and Sequencing of Target Genes from Environmental DNA

One DNA extract from partially oxygenated bottom water of Lake Erie (*nosZ* only) and 2 DNA extracts from anoxic waters of the strip mine lake were selected for the construction of small clone libraries in order to obtain target sequences from environmental samples. Amplification reactions of 50 *μ*L volume were catalyzed by OneTaq HotStart polymerase in Standard Buffer (New England Biolabs), with 0.5 *μ*M of forward and reverse primer and 30 ng template DNA, in an ABI Veriti thermocycler (Applied Biosystems). The thermocycler protocol consisted of 1 min at 95°C, followed by 40 cycles of template denaturation (20 s at 95°C), primer annealing (20 s, temperature as in Table 2), and extension (45 s *nifH*, 90 s else, 68°C), then 15 min at 68°C. The PCR products were purified (MoBio UltraClean GelSpin kit) before verifying length and purity by agarose gel electrophoresis.

Applying the TOPO TA cloning kit for sequencing (Invitrogen), purified PCR products were cloned into the TOPO TA cloning vector, and chemically competent OneShot TOP 10 cells were transformed with the vector, following the manufacturer's directions. Transformant cultures were plated on LB-Miller agar containing 50 mg L^−1^ ampicillin and incubated at 37°C overnight. From each library, 15–130 transformant colonies were picked for transfer into 50 *μ*L PBS buffer and sent to a GLP-compliant service lab (ACGT Inc., Wheeling, IL, USA) for direct colony sequencing.

DNA sequences that passed the sieving procedure described in the following paragraph were deposited in GenBank under accession numbers JX626144–JX626236.

#### 2.3.4. Processing and Evaluation of Clone Sequences

The sequencing reads were inspected visually for clarity; noisy or spurious reads were discarded. The remaining sequences were compared to the Genbank database by BLAST [40]; those of high similarity to vector sequences were discarded. Employing the Geneious Pro software (Biomatters Ltd.), sequences were searched for the location of forward and reverse primer, reverse complemented where necessary, and ends outside the primers were trimmed off. These shorter sequences were then translated in silico, trying all 6 possible reading frames. Translation products containing stops were discarded and from the remaining deduced peptide sequences, duplicates were removed. Unique peptide sequences were then scrutinized for protein family membership by comparison with the Conserved Domain Database of annotated protein sequences [41]. Members of nontarget protein families were discarded. All remaining peptide sequences were then compared to protein sequences in the curated RefSeq database [25], applying the DELTA-BLAST tool [42] with a BLOSUM62 scoring matrix. Alignment scores were used to identify the closest archaeal and the closest bacterial relative.

## 3. Results and Discussion

### 3.1. Primer Design

**Figure 1:**
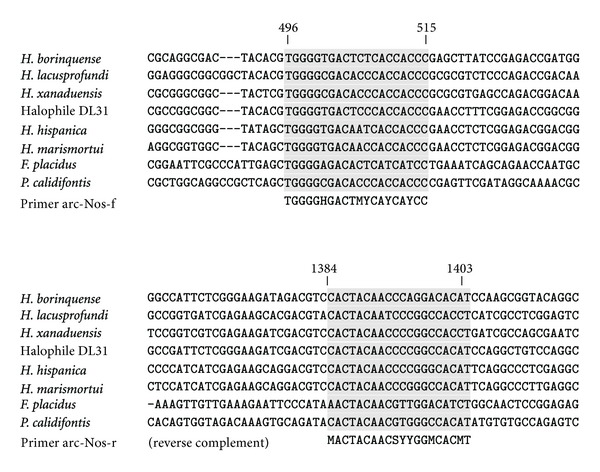
Alignment of *nosZ* gene fragments from 6 members of the order Halobacteriales, *Ferroglobus placidus,* and *Pyrobaculum calidifontis*, with binding sites of the novel primer pair arc-Nos-f/-r shaded. Reference positions given at the top refer to the *nosZ* gene of *Halogeometricum borinquense*.

Primer pairs were designed for the amplification of 5 archaeal target genes, based on all available reference sequences from thermophilic, halophilic, and methanogenic Archaea possessing these genes. Alignment of these orthologous sequences did not reveal any conserved regions of sufficient length (18–25 nt) to be targeted by primers without degenerate positions. The design of degenerate primers, allowing for maximum degeneracy of 128, aimed for an optimal tradeoff between sensitivity and specificity in the detection of target genes. The names and sequences of the resulting primer pairs are summarized in Table 1. An alignment of all archaeal *nosZ* reference sequences and the corresponding pair of primers is shown in Figure 1.

### 3.2. In Silico Primer Evaluation

**Figure 2:**
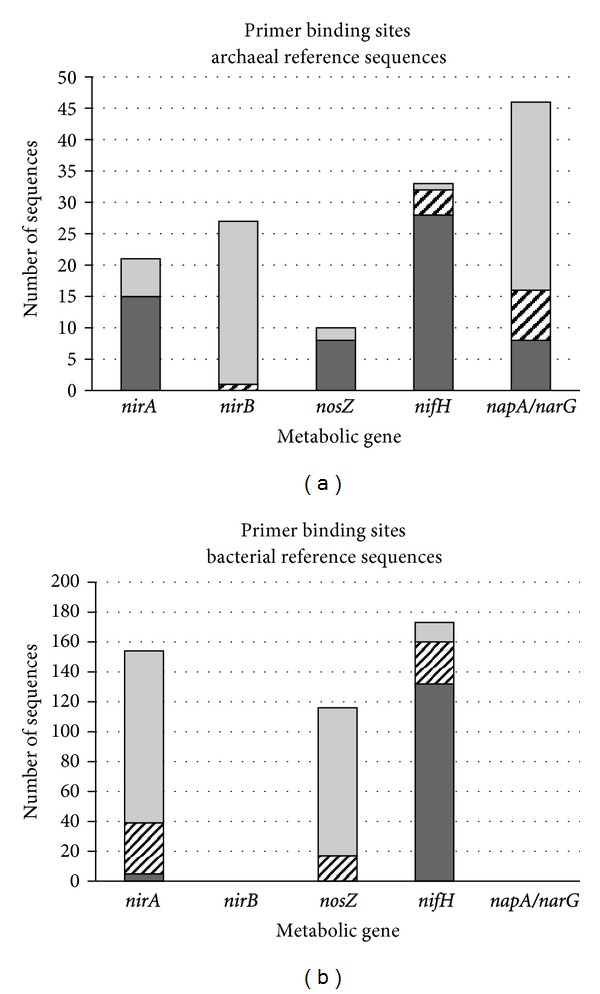
In silico evaluation of primer sets for the detection of archaeal metabolic genes. Archaeal (a) and bacterial (b) reference sequences containing the targeted metabolic gene were screened for binding sites for the gene-specific primers. Dark grey: sequences with binding sites for both primers; hatched: sequences with binding sites for one primer; light grey: sequences without binding site.

The newly designed primers were evaluated in silico for their effectiveness in binding to archaeal reference sequences and for their phylogenetic specificity as measured by the absence of binding sites in bacterial reference sequences. The results are illustrated in Figure 2. Primer set arc-NirA showed high affinity to archaeal *nirA* sequences, but little complementarity to bacterial ones. Likewise, primer pair arc-Nos turned out to be sensitive to most of the archaeal *nosZ* sequences and largely ignorant of bacterial homologs (Figure 2). These two primer sets meet the design goals of both sensitivity and phylogenetic specificity.

Primer pair arc-Nif found binding sites in most archaeal and many bacterial *nifH* reference sequences (Figure 2), indicating high sensitivity towards the target gene, but little if any discrimination between archaeal and bacterial homologs. Depending on the specific application, the codetection of bacterial *nifH* sequences may be tolerable or even desirable. For example, research addressing the entire guild of N_2_-fixing organisms can conveniently apply a single primer pair that offers great coverage of both domains.

Primer pairs arc-NirB and arc-Nred showed poor detection of archaeal reference sequences for the respective target genes (Figure 2). Screening for binding sites in bacterial *nirB*, *napA*, and *narG* sequences was dismissed as obsolete. The surprisingly low affinity to archaeal target sequences can be attributed to the different settings applied during design and evaluation of the primer sets. The smaller, possibly more stringently curated, KEGG database [24] was used in primer design, where accuracy was of prime importance. Primer evaluation, which focused on sensitivity and phylogenetic specificity, required a more inclusive approach and, therefore, was based on the larger and differently curated RefSeq database [25]. In addition, up to 3 mismatches per pair were allowed during primer design, with enhanced sensitivity in mind, whereas only 1 mismatch per primer was allowed during evaluation, under the scenario of relatively stringent PCR conditions. The value of arc-NirB and arc-Nred may be underestimated by the measures applied, and these primer pairs may perform much better with low-stringency PCR protocols and targets from natural rather than RefSeq environments.

### 3.3. In Vitro Primer Evaluation by Real-Time PCR

All newly designed primer pairs were tested for their capacity to amplify the target gene in actual PCR reactions. Using template DNA from positive control organisms, annealing temperature and extension times were optimized for each primer set. The results are summarized in Table 2. Under optimized conditions, all primer sets amplified DNA segments of the expected length. With *Halogeometricum borinquense* for a positive control, the use of primer pairs arc-NirA and arc-Nred led to the formation of additional products (Table 2), likely due to additional primer binding sites in this archaeon's genome. 

When 22 DNA extracts from water samples instead of pure cultures were used as PCR templates, products obtained with the arc-Nif primer pair formed a single band between 400 bp and 450 bp on the agarose gel. Amplification products obtained with arc-NirA or arc-Nos primers varied in size and number. While products from several samples were below gel detection (but visible to the real-time thermocycler), others formed 1 band within the expected size range, but also 2 and rarely 3 bands were observed. These unexpected products might result from additional primer binding sites within the same genome, but could also reflect the natural diversity of each target gene, existing in different lengths among the many gene owners in natural habitats.

### 3.4. Evaluation of Primer Specificity in Environmental Samples

**Figure 3:**
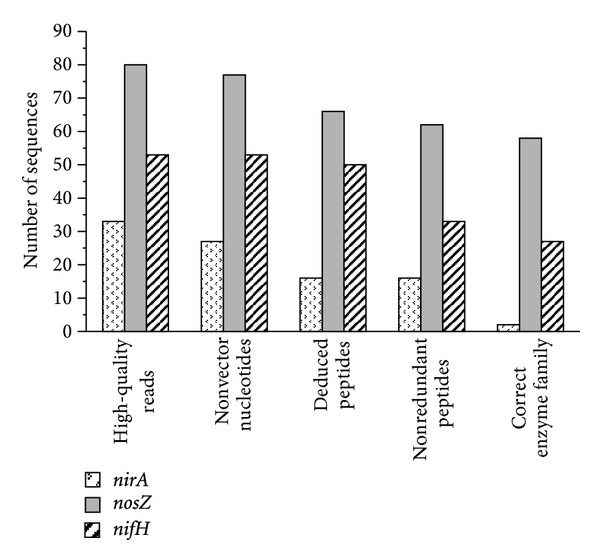
Sieving of sequences after cloning of PCR products obtained with novel primer pairs for target genes *nirA*, *nosZ*, and *nifH*. The 5-step refinement procedure included the removal of (1) low-quality sequencing reads, (2) empty vector sequences, (3) translation products with stops, (4) redundant peptide sequences, and (5) peptide sequences outside the targeted protein family. Where the number of retained sequences decreased steeply indicates potential sources of error in practical applications of the primer pairs.

The 3 primer sets that appeared most promising from in silico evaluation were tested in PCRs with template DNA from lake water samples. Subsequent cloning and sequencing of the products generated 33–80 nucleotide sequences for each primer pair (Figure 3, first set of columns). These sequences were refined further and evaluated in regard to specificity for the target gene. Figure 3 illustrates the loss of sequences during each of the refinement steps. Only very few sequences originated from empty vectors; thus, the ligation and transformation procedure gave no reason for concern. 

Losses during the in silico translation of DNA sequences into deduced peptides (2nd-to-3rd set of columns in Figure 3) were due to the occurrence of internal stops in all possible reading frames. Such sequences represent amplificates of noncoding regions; their number was negligible for *nifH*-targeted and tolerable for *nosZ*-targeted amplifications, but amounted to one-third of the *nirA*-targeted ones. Thus, noncoding DNA may be a significant byproduct of PCRs with arc-NirA primers.

Redundancy of deduced peptide sequences (3rd-to-4th column in Figure 3) can generally originate from naturally low diversity of the targeted guild in the sample or from primer bias reducing the detectable diversity. Peptide sequence redundancy was observed to a minor extent only in all 3 primer pairs tested.

In the last refinement step, peptide sequences were tested for putative membership in the targeted protein family. Losses in this step represent amplificates of nontarget genes. Only 2 of originally 33 PCR products obtained with arc-NirA primers were found to belong to the targeted family (Figure 3). This primer pair is likely to amplify nontarget genes in addition to noncoding DNA regions. While performing well in silico (Figure 2) and on archaeal pure cultures (Table 2), primers arc-NirA failed to show satisfactory specificity for their target gene in the presence of numerous competing DNAs in environmental samples. Sequences resulting from PCRs applying primers arc-Nos or arc-Nif, in contrast, rarely fell outside their target family (Figure 3). Note that nitrogenase reductase populates two protein families: one of them comprising NifH exclusively and the other including NifH and several NifH-like proteins, which are all involved in chlorophyll or bacteriochlorophyll synthesis. Affiliation with the latter family does not necessarily imply NifH identity, unless the presence of phototrophic organisms can be ruled out.

In order to verify the archaeal origin of the amplified sequences, the corresponding peptide was compared to the RefSeq database of protein sequences [25]. Its closest relatives among archaeal and bacterial protein sequences were identified, and the corresponding sequence identities were compared. Results differed noticeably with the choice of alignment algorithm and scoring matrix and, therefore, should be viewed with caution. In addition, databases are strongly dominated by bacterial sequences; for example, among the known sequences of key enzymes of denitrification (NirK, NirS, NorB, and NosZ), only 1% are of archaeal origin [30]. Due to this bias, identifying the closest neighbor of a novel sequence may not necessarily indicate its correct affiliation, especially when alignment scores and sequence identity are low.

**Figure 4:**
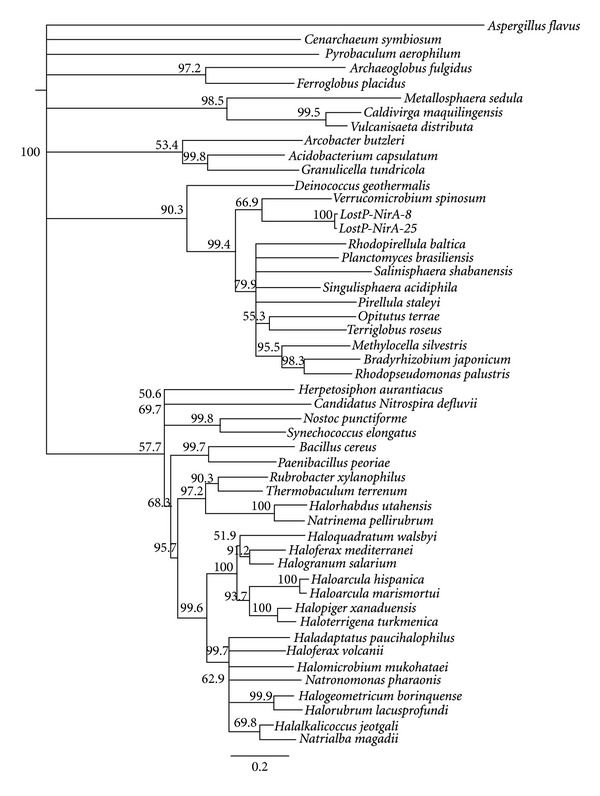
Neighbor-joining tree of ferredoxin nitrite reductase (NirA) sequences obtained from KEGG [24], RefSeq [25], and 2 novel clones (LostP-NirA-8 and -25). All sequences were truncated to the segment between the binding sites of primer pair arc-NirA, aligned by a ClustalW algorithm with BLOSUM cost matrix, and tree construction followed a Jukes-Cantor model with bootstrap resampling (1000 replicates). The eukaryotic NirA sequence of *Aspergillus flavus *was used for an outgroup. Branch labels indicate bootstrap support in %; branches with <50% support were collapsed.

The relatedness of NirA sequences deduced from lake water clones to homologous segments of archaeal and bacterial reference sequences is illustrated in Figure 4. Both novel sequences appear to group with representatives of the deep branching bacterial phylum *Verrucomicrobia*, showing 47% identity with their closest relative.

Fifteen NosZ sequences were found less than 35% identical with their top matches in both the archaeal and the bacterial domain, so that it was impossible to decide their affiliation. The remaining 43 NosZ sequences, forming 2 clusters, showed around 90% sequence identity with NosZ of the bacterial candidate species *Accumulibacter phosphatis*, compared to around 30% identity with their best match among archaeal sequences. These *nosZ* clones are most likely of bacterial origin, despite the excellent discrimination of primer pair arc-Nos against bacterial *nosZ* genes (Figure 2). The formation of bacterial byproducts may be promoted by the low overall abundance of archaeal cells in the lake water samples (4–7%, Rusch and Wham, ms in preparation). Under scarcity of archaeal targets, even the disfavored bacterial targets may compete successfully for primers by virtue of their overwhelming abundance. In habitats supporting slightly higher abundances of Archaea, the arc-Nos primer set may primarily detect archaeal targets, owing to its excellent phylogenetic bias (Figure 2).

The NifH or NifH-like sequences generally exhibited between 50% and 90% sequence identity with the best aligning archaeal and bacterial NifH sequences. Several of the clones were convincingly closer to their top bacterial match, while others showed almost equal distance to their archaeal and bacterial top match. Conclusive judgment on domain affiliation is not possible here, given the database bias and methodical uncertainty discussed above. Based on in silico evaluation (Figure 2), the arc-Nif primers were not expected to be domain-specific anyway.

## 4. Conclusions

Complementary culture-based and culture-independent ecological research makes our strongest approach to elucidating the complex network of microbial functions in ecosystems. In the much underexplored archaeal domain, however, the paucity of suitable molecular tools may hamper culture-independent investigations. This study addressed the need for such tools by designing and evaluating primer pairs for the amplification of 5 archaeal metabolic genes of relevance in the biogeochemical N cycle. Sensitivity towards archaeal target sequences is essential for the detection of novel Archaea by PCR amplification with these primers. Beyond mere detection, sensitive gene-specific primers are also important for assessing guild diversity by clone-based or direct sequencing surveys. Advanced applications include the quantification of the target guild in the metagenome or metatranscriptome of a microbial community. Finally, successful primers often double as probes in mRNA-targeted fluorescence in situ hybridization (FISH) for quantifying and localizing gene expression.

The novel primer pairs designed for amplifying archaeal *nirB* and *napA/narG* genes were not found sensitive to many of their target sequences (Figure 2), thus not suitable for application in environmental studies. Primers arc-NirB were applied successfully in real-time PCR with template DNA from a positive control organism (Table 2); they may prove useful in pure culture experiments, such as measuring gene expression for ecophysiological assays.

In silico, primer arc-NirA showed both sufficient sensitivity to archaeal target sequences and the desirable discrimination against bacterial homologs (Figure 2). When evaluated in vitro on DNA from archaeal pure cultures, these primers performed excellently with 2 of the 3 species tested (Table 2). They are expected to prove useful in qualitative and quantitative pure culture experiments. When tested on DNA from lake water samples, however, the *nirA*-targeted primers produced a significant number of amplicons from noncoding DNA and nontarget genes (Figure 3). They are not suitable for quantitative assays in environmental samples, but could be applied in qualitative surveys of guild diversity with subsequent removal of false positives.

The novel *nosZ*-targeted primer pair showed great sensitivity and phylogenetic specificity (Figure 2), performed reliably in real-time PCR with all 3 pure cultures tested (Table 2) and generated tolerable numbers of nontarget amplicons from lake water DNA (Figure 3). This primer pair is considered suitable for the full range of applications targeting archaeal denitrifiers. When used in environments of very low archaeal abundance, some bacterial byproducts are possible, though.

Primers arc-Nif exhibited excellent sensitivity towards both archaeal and bacterial *nifH* sequences (Figure 2), generated the expected products from pure culture DNA (Table 2), and amplicons from noncoding DNA or nontarget genes were rare (Figure 3). Missing phylogenetic specificity, this primer pair is suitable for all pure culture experiments, but must be used with caution in environmental applications. Qualitative surveys of diazotroph diversity using arc-Nif primers will return sequences from both domains, which can be separated afterwards. Whether this novel *nifH*-targeted primer pair is any superior to the existing ones [35] remains to be seen.

## Supplementary Material

This file contains all known archaeal sequences of the metabolic genes nirA, nirB, nosZ, nifH, napA and narG (KEGG database, 2012).

## Figures and Tables

**Table 1:** Primer sets for the PCR amplification of archaeal genes that encode key enzymes of nitrogen metabolism.

Process in N cycle	Key enzyme	Target gene	Primer name	Primer sequence	Reference
NH_4_^+^ → NO_2_^−^	Ammonia monooxygenase (E.C. 1.14.99.39.)	*amoA *	arc-Amo-f arc-Amo-r	5′-STAATGGTCTGGCTTAGACG-3′ 5′-GCGGCCATCCATCTGTATGT-3′	[18]
NO_2_^−^ → NH_4_^+^	Ferredoxin nitrite reductase (E.C. 1.7.7.1.)	*nirA *	arc-NirA-f arc-NirA-r	5′-AAYMTSCCNCGGAAGTKSAA-3′ 5′-AGAACTCCBTRCCSGTRCAS-3′	This study
NO_2_^−^ → NH_4_^+^	Ammonifying nitrite reductase (E.C. 1.7.1.4.)	*nirB *	arc-NirB-f arc-NirB-r	5′-ATGCTGAGCCATTAYATAGC-3′ 5′-CCGTTGTACTCGGCRCAGTC-3′	This study
N_2_O → N_2_	Nitrous oxide reductase (E.C. 1.7.2.4.)	*nosZ *	arc-Nos-f arc-Nos-r	5′-TGGGGHGACTMYCAYCAYCC-3′ 5′-AKGTGKCCRRSGTTGTAGTK-3′	This study
N_2_ → NH_4_^+^	Nitrogenase reductase (E.C. 1.18.6.1.)	*nifH *	arc-Nif-f arc-Nif-r	5′-TAYGGAAARGGNGGNATYGG-3′ 5′-CCNCCRCAGACRACRTCNCC-3′	This study
NO_3_^−^ → NO_2_^−^	Dissimilatory nitrate reductase (E.C.1.7.99.4.)	*napA, narG *	arc-Nred-f arc-Nred-r	5′-CGACTGGTAYKCVGAYCTHC-3′ 5′-GTCRGYGTKRWACCAGTSGK-3′	This study

**Table 2:** In vitro evaluation of primer pairs by real-time PCR. DNA extracted from archaeal owners of the target gene served as positive control template during the optimization of annealing temperature (*T*_ann_) and extension time (*t* ext).Expected lengths of the amplification product refer to distances between primer binding sites in the archaeal sequences that were used during primer design. Observed product lengths were determined by agarose gel electrophoresis of the actual PCR products.

Primer pair	Positive controls	*T*_ann_ in °C	*t* ext in s	Product length in bp	
				Expected	Observed
arc-NirA-f, -r	*Halorubrum lacusprofundi* *Haloarcula marismortui* *Halogeometricum borinquense *	62	75	660–750	700 700 700, 350
arc-NirB-f, -r	*Thermococcus sibiricus *	64	90	680	700
arc-Nos-f, -r	*Halogeometricum borinquense* *Halorubrum lacusprofundi* *Pyrobaculum calidifontis *	64	75	910–1030	950 950 1050
arc-Nif-f, -r	*Methanotorris igneus* *Methanosarcina acetivorans *	60	60	360–415	400 400
arc-Nred-f, -r	*Halogeometricum borinquense *	64	90	760–1030	several bands
