# Supplementary material for: Molecular Tools for the Detection of Nitrogen Cycling Archaea
Source: Archaea. 2013 Jan 8;2013:676450. doi: 10.1155/2013/676450 (PMC3556428; doi:10.1155/2013/676450)
Supplement: Supplementary file 1 — This file contains all known archaeal sequences of the metabolic genes nirA, nirB, nosZ, nifH, napA and narG (KEGG database, 2012). [file 676450.f1.pdf]

>hbo:Hbor_04420-Ar sulfite reductase, subunit beta (hemoprotein); K00366 ferredoxin-nitrite reductase [EC:1.7.7.1] (N)
ATGCCCACGGACGTCGAGCGTTGGAAATCTGAGGTGTACGGGAACGAAATACGCGAACATCTGTACGAGTTCG
CAGAAGAAGGATGGGACGCAATCCCCGAGGACGAACACGACGAGTGGTTCGAGCGATTCAAATGGTGGGGACT
GTACCACCAGCGGAACGGGCAGGAGTCGTACTTCATGATGCGGATTGGGACGCCAAACGGCGTTCTCGAACCG
GGGCAACTGCGCGTCGTCGGCGAAATCGCAGCGGAGTACGCCACCGGTCCGGGGACGAATCCGATCTTCGGTG
ACGCTTATGCCGACTTCACGACCCGGCAGTCGATCCAACTACACTGGATCAACATCGAAGACGTGCCCGCGAT
CTTCGAGAAGATGGAGAAAAACGGGTTGTCCGCCCAGCAAGCGTGTGGTGACTCGTGGCGCAACATCGTCGGC
AACCCCGTCGCCGGGAAGGACGCTCAGGAAGTCATCGATGCGTGGCCCGTTATCCGCGAACTCAACGAGACGT
TCAAAGGCAACGAGGATCACTCGAACCTCCCGCGTAAGTGGAAGGTTTCCGTGACGGGGTCCGCGGACGGGTC
CGGACAGGGGGACATCAACGACCTCGCCTTCGAACCGGCGTACAAGGAGGTTGACGTCGAAGAGAAGGTCGGT
TTCAACGTCCGCGTCGGCGGCGGTCTCGCCCGCAACGAGGAGCGCTTTGCCCGCGACATCGACGTGTGGGTCG
CTCCCGAGAACGTCTCCGACGTGGCGGGCGGCCTCTCTGCGCTCTTCCGCGACAACGGCGACCGCGAAAACCG
CTACAACGCTCGTATCAAGTTCCTCGTAGACGAGTGGGGGCCGGAGAAGGTTCGCAACGTCCTGCAGGAAGAG
TACGTTGACTTCGAACTCCACACCGCAGGCCGCGACGTCCGCGAGGAGTACACGTACAACACCGGCAGTGGCG
GACGAAACGACCTGATCGGCGTCCACGAACAGAAGGACGGCAACTACTTCGTCGGCTTGAACGTTCTCGTCGG
CCGCATGGGTGCCGAAGACGTACTCGAACTCGCCGACCTCGCGGACGAGTACGGGTCCGGCGAAGTCCGTCTC
TCTCAGCGGCAGAACGTCATCGTCACCGACGTACCCGAAGAAGACCTCGACGACTTCCTCGGCGAAGACCTGC
TGGAACACTACTCGCCCGACCCGTCACCGTTCATGCGTGGCTCGGTTGCGTGTACCGGTACCGAGTTCTGTTC
GCTCTCTATCGTGGAGACGAAGAACCGACAAGTCCGCTACGCGCGGTGGCTGAAGGAGAACGTCGAACTTCCC
GACGACATCGATCAGTTCCACATCCACCTCTCCGGATGTACGGCCTCCTGCGCACAACCGCAGATTGCGGACA
TCAGCCTCCGCGGTATGAAGACCCGAAAGAACGGCGAACCCGTCGAAGCACTCGACGTTGGCCTCGGCGGCGG
TCTCGGCGAGGAACCGCAGTTCGCACGCTGGGTCACCCAACGGATTCCCGTAGACGAGGTTCCGGGTGCAATC
TCGAATCTGCTGGATCAGTTCAAAGCGAACCGTGAGGACGGCGAGTCGTTCCGCGAGTTCGTCGCCCGGCACG
ATGACGAGGAACTCGACGAGTTCGTCGAACCCGAGGAAACGGACTACGAGGACCCGATGATGCACAACACGAA
GATGACGTGGTACCCCTACGCCGACGAGGACTCTATGGACGCGAGTCCAGCCCCGACGTTCCCTGACGACTCA
CCGATGTCGGCGGACGATTGA
>hbo:Hbor_39920-Ar sulfite reductase, beta subunit (hemoprotein); K00366 ferredoxin-nitrite reductase [EC:1.7.7.1] (N)
ATGAACACTGTTGAACGATGGAAACAAGAACAACATCCGCTCGATGTTATCGAGAACGTCTACGAGTACGCTG
AGGGAGATTTTTCTTTCGAGGAGATTGAGGCACAAGCAGGCGACGGCGAGTGGGAGCGTCTGAAATGGGCCGG
AATGTACGCCCACGGACGCCAGGACGACTACTTCATGCTTCGGACCAAAGTTCCCGGGGGATTTCTCACGCCT
GAACAGGCTGAAGCCATCGGTACGGTTGCCGACGAGTACGCTACCGCTCCCGAGGAGCACGGTGGCGAGGCAC
AAAACGCAATCTGGGGTGACGCATTCTTGGATCTCACCACCCGTCAGGACGTCCAGATGCACTGGATCGCGGT
CACCGACATACCCGCGATCTGGGACACGTACGAAGCAGTTGGCTTGACCACGATTCAGGGCTGTGGTGACTCC
GCACGCAACGTCCTCGGCTGTCCGGCGGCCGGTCTTGACGATCACGAATGCTTCGATGCTCAGCCAGTTATCG
ATGCTGTCTCGGACTTTTTCACCAATAATCGGGAGTACGCCAACCTCCCGCGGAAGTTCAAGATGACCATAAC
CGGGTGTCAGGAGGACTGCGCCCAGTCCCAGATCAACGACGTCGGTCTGGTCCCGGCTCGAAAGCATGTTGAC
GGCGAGGATATCTACGGCTTCCATGCCCGCGTCGGTGGTGGTCTCTCAGATGGTCCGCGGATGGCCTCGAATA
TCGATGTCTTCATCCGACCCGAAGACGCCGTGGAGTTCTGTCGTGCGGTCGCTCAGACGTTTAAAGAACTGGG
TGATCGAACCAACCGTGGACTCTGTCGGATGCGCTATCTCGTCCAGCAGATGGGACCGAGTACATTTGAGGCG
GCTGTTCGCGACCGCTGTGATGTCGATCTCCACTCTCGAGGACAGGATCTCACCCGTGGTTACCGGGGTGATC
ACGTTGGCGTCCATCCTCAAAAACAGGACGGCCTGTTCTATGTCGGTTTCAACGTTATCGCCGGACGAATGGG
TGGCGACGAACTTACGGCGGTTGCACGAGCAGCCAAGAAATACGGAACCGACGATACCTCGATTCGGTTGGCG
ACTGACCAGAACTTCCTTCTCACTCATATCCCCGAAGACAATCTTTCGGCACTACTGAACGAACCGTTCGCCC
GGAAGTACGAACACAATCCTGGGCCGTTTGCAAGGGGTGTGGTCGGGTGTACGGGCTCGGAGTTCTGCAGCTA
CGGGATTATCGAGACGAAAAAGCGTGTTTACCGCTGGGCACGGGCACTAGACCGCCAGATAGCCACGCCGGAC
GACCTTTCGGTGGTTCGGATGCACATGTCGGGTTGTTCGGCGTCCTGTGCACAGCCCCAAATCGCGGATATCG
GATTCCGCGGCGAGACGGTTGTGGTGGACGACTCCGAAGGAAGCACGAACGAAGCGGGTGATAATATCACCGA
GGGTATGGATGTCGGCCTCGGCGGTAGTCTTGGGCCCGACAACGAGTTCCTCGATTGGGTTGCGCACGCAGTC
CCTGCGCAGGCCGTGATTCCGGCACTACAGCAACTATTCACCGTCTTTACCGAAGAGCGCGACCCCGACGAGC
GATTCTACGAGTGGGCCCGCCGGGTGAGCAACGACCGCCTTCGAAGGATCATGCAACGGGCGGACGCTAACGT
TTCCGGAGGGGTCGCTCATGACGGCTGA

>htu:Htur_0174-Ar nitrite and sulphite reductase 4Fe-4S region; K00366 ferredoxin-nitrite reductase [EC:1.7.7.1] (N)
ATGGTGCATAAGAAAGAAGAACAGAAGGAAGACTGCTACGGCGACGAAGTGCGGGAACACATCGAGCGGTTCG
CCGAGAACGGCGGCTTCGAGGCCATCCCGGACGACGAGATCGACAAATGGACCACCCGGTTCAAGTTCTGGGG
CGTGTTCCAGCAGCGCTCCGGCCAGGAAGAGTACTTCATGATGCGGCTGACCAACGCCAGCGGCATCTTGGAG
CCGGAACAGCTGCGCCGCATCGGCGAAGTCGCGAAGGAGTACGCGAAGGGTCCCGTCGCAAATCCCGAATTCG
GGAATGGGTGGATCGACCTGACGACCCGACAGTCCGTCCAGCTCCACTGGCTCAAACTCGAGGATATCCCCGA
GATCTGGGAACAGCTCGAGGCCGTCGGCGTCCACTCCCGCTCGGCGGGCGGTGATACGATGCGCAACATCTCC
GGCTGCCCGCTCCACGGCAAGGCCGAGGAGTTCGTCGAAGCCGGTCCCCTGCTCGAGCGCTTCGAGGAGGAGC
TTCGCAAGGACGACGCGCTCGCGAACATGCCCCGGAAGTTCAACATCAGCGCGTCGGGCTGTACGGTCGGCTG
CGCACAGGACTCGCTGAACGACATCGGGTTCGAGCCGGCGACCAAGGAGATCGACGTCTCCGGCGGAGCCGGA
GGCTCGTCGGACTCGTCCGACGGTGGCGAAGAAGTTCGAGGATTCAACGTCCGCATCGGCGGCGGGCTCGGCG
GCCGCCAGCCCCGCGCCGCGACGCCGCTCGATATCTTCGTCCGGCCCGAAAACGCCTACGAGGTCGGCCGCGG
CTTCGTCGAACTCTACCACGACTACGGCAACCGGCAGAACCGCTCGAAGAATCGCGCCCGGTTCTTCGCCGAG
GACTGGGGGATGGAGAAGATCCGCGAGACGCTCCAGGAGGAGTACGTCGACTTCGAAATGCACACCGCCGGGG
AGGACTTCCGCGAGGAGTACACCTACAACGCCGGCCGCCCCGTCGAGGACGGTCAGCACGACCACGTCGGTGT
CGGCGACCAGAAGGACGGACAGAACTACGTCGGCCTGAGCGTTCCCGTCGGCCGGCTCCCGGCCGAGGACGCC
ATCGAACTGGCCGATCTGGCCGACGAGTACGGCTCCGGCGAGGTCCGGCTGACCCGCCGCCAGAACCCCGTCA
TCGTCGACGTCGCCGACGAGGACCTCGAGGAGCTGCTCGCGGAACCGATCCTCGAGGAGTACCCCGCCGAGCC
GAGCCCCTTCGAGCGCGGGGCGATGGCCTGTACCGGCACCGAGTTCTGCTCGATCGCGCTGACCGAGACGAAG
GGTCGGATGGCCCGCATGCTGCGCTGGTTCAACAAGAACGTCGAGCTGCCCGACGACGTCGGCAAGATCAAGA
TGCACTACTCCGGCTGTACGGCCGACTGCGGCCAAGCGATGACCGCCGACATCGGCCTGCAGGGCATGCGCGC
GCGCAAGAACGGTGAGATGGTCGAAGCCTTCGATATCGGCGTCGGCGGCGGCGTCGGCGAGGACCCCTCCTTC
ATCGACTGGATTCAACAGCGCGTGCCCGCCGACGAGGCCCCCGGCGCGATCCGGAACCTGCTCGAGGCCTACG
CGGCTCATCGTAGCGAGGGCCAGGCGTTCCGCGAGTGGGTCGAGGCGACGGGCGAGGAACAGCTCGTCGAGTT
CTGCGAACCCGAGGAGACCGACTTCGAGGCCCCGTACATGGACGACGCCAAGCAGTCTTGGTACCCCTTCGCC
GAGAGCGAGTCCGCGGCGGCCGCCGCCGGCGAGGGATCCGTGGCCCCGTCCGACGACTGA
>htu:Htur_0241-Ar nitrite and sulphite reductase 4Fe-4S region; K00366 ferredoxin-nitrite reductase [EC:1.7.7.1] (N)
GTGGTATCTATGAATACGACGGAACAATACAAACAGAACAAGCACCCGCTCGACGTCATCGACGATGTCTACG
ACTACGCCGACGAGCAGCTGAGCTTCGAGGAGATCGAGGAGCGCGCCGGCGGCGGCGAGTGGGAGCGCCTGAA
GTGGGCCGGCATGTACGCCCAGAAGCAGGAGGGTTACTTCATGATCCGGACTAAGGTGCCGGGCGGCAAGCTC
ACGCCCGAACAGGCCGAGGTCATCGGCGAGGTCACCGAGGACTACGCCGTCGCTCCCGAGGAGTACGGCGGCG
AAGAGCAGAACGAACTCTGGGGCGACGCCTACCTCGACATCACGACGCGACAGGACATCCAGAAACACTGGAT
CCGCGTCGAGGACGTCCCGGAGATGTGGGAACGGTACGATGAGGTCGGCCTGACGACGGTTCAGGGCTGTGGC
GACTCGGCCCGGAACGTTCTGGGTTGCCCCGCTGCCGGCCTCGACGACCACGAGTGCTTCAACGCGCAACCGG
TCATCGACGCCGTCTCGGACTACTTCACGAACAACCGCGAGTACGCCAACCTCCCGCGAAAGTTCAAGATCAC
GATCACCGGCTGCGCCCACGACTGCGCGCAGTCCCAGATCAACGACGTCGGTCTCGTCCCCGCGAAGAAGGAG
GTAGACGGCGAGTACCTCTACGGCTTCCACGCCCGCGTCGGCGGCGGCCTCTCCGACGGCCCGCGGATGGGCT
CGGAGCTCGACGTCTTCATCCGACCCGAGGACGCCGTGGAGTTCTGCCGCGCCGTCGCCCAGACGTTCAAGGA
GCTCGGCGACCGCAACAACCGCGGCGTCTGCCGCATGCGCTACCTCGTCGAGCAGATGGGCCCCGAGAAGTTC
GAGGAGGCCGTCCGCGACCGCTGTACGATCGACCTACCCACCGGCGGCGAGAACTTGACCGTCGGCTACCAGG
GCGACCACGTCGGCGTCCACGAGCAGAAGCAGGACGGACTCAACTACGTCGGCTTCAACGTGATCGCCGGCCG
CATGGGCGGCGACGAGTTCGCCGAGGCCGCCCGCGCCGCCGAGAAGTACGGGACCGAGGACGCCTCCGTGCGT
CTGGCGACCGATCAGAACTTCCTGATCACTCACATCCCCGACGAGAACGTCGACGACCTGCTGGCGGAGCCGT
TCGCCCGGGAGTACAGTCCCGATCCGGGGCCGTTCTCCCGCGGCGCGGTCGGCTGTACGGGCAACGAGTTCTG
TAACTACGCCATCATTGAGACGAAAAAGCGAACCAAGCGGTGGGCCCGCGAGCTCGACGAGCGCATCGACGTC
CCCGACGACATCGAGGCCATCCGGATGCACATGTCCGGCTGCTCGGCCTCCTGTGCCCAGCCCCAGATCGCCG
ACATCGGCTTCCGCGGCGAGACGGTCAAACTCGAGGACGAGAACAGCACCAACGCCGAGGGCGACAACATCGT
CGAAGGCATGGACTTCGGACTCGGCGGCTCGCTCGGCGCCGACAACGAGTTCCTCGACTGGGTCGAGAACGCC
GTGCCCGCCCAGTCCGTGATTCCGGCGCTCGAGCAGCTGTTCGAGGCCTACAGCGAGGGCCGCGACGACGGCG
AGAAGTTCTACGAGTGGACCCGCCGCATCGACAACGAGCGCCTCCGACAGATCATGCAAGGGGCCGACGCGCC
GGTTGCACGAGGTGTTGCCCATGGGGACTAA

>htu:Htur_2855-Ar nitrite and sulphite reductase 4Fe-4S region; K00366 ferredoxin-nitrite reductase [EC:1.7.7.1] (N)
ATGGCAAGTGATGTCGAGCAGTGGAAGGACGAACTCTACGGAACCGATATTCGCGACCAGATCGAATTCTTCG
CCGAGGAGGGATGGGAGTCCATCCCCGACGACGAACACGACGCCTGGTTCGAGCGGTTCAAGTGGTACGGCCT
GTACCACCAGCGAGCCGGCCAGGAATCGTATTTCATGATGCGAATCGGTCCACCGGGGGGCGTCCTCGAACCG
GGCCAGTTTCGACGCATCGTCGAAATCGCGGACGAGTTCTCCCGCGGTCCCGTCGAAAATCCCGAGTTCGGCA
ACGGCTGGCTCGACGTAACGACTCGTCAAGCGATCCAGCTCCACTGGATCCGCCTCGAGGACGTGCCCGAGAT
CTTCGAGCAACTCGAGGACGTCGGTCTCTCGACGGTCCAGGCCTGTGGTGACTCCTGGCGAAACATCGTCGGC
TGTCCGGTCGCCGGCAAGGACAAACACGAGCACGTCGACGCCGACTGGCTCGCAACCGAGCTTCACGACACGT
TCAAAGAGAACGAGGCGCACTCGAACCTGCCGCGGAAGTGGAAGGTCTCGGTGACCGGCTGTGACGAGGGCTG
CGGACAGGGCGACATCAACGACCTCGCGTTCGAGCCCGCCGAGAAGGAGATCGACGGGGAGAGCGTGACGGGC
TACAACGTTCGCGTCGGCGGCGGTCTCTCGCGCAACGAGCCGCGCTTCGCGCGCAACATCGACGTCTTCGCAA
CGCCCGAGCAGGCCGTCGAGGTCGCCGGCGGGCTCTCCGCGCTGTTCCGCGACCACGGCGACCGCGAGGACCG
CTACAACGCGCGCATCAAGTTCCTCGTCGACGAGTGGGGCCCCGACAAGGTCCGCGAAACGCTGCAGGAGGAG
TACGTCGACTTCGAGCTCGAGACCGCGGGCGAGGACCTCCGCGAGTCGTACTCGTACAACGCGGGCGAGGCCG
ACGGCAAACACGACCACGTCGGCGTTCACGAGCAGGTTGGCGGCGACTACTACGTCGGACTGAACGTCCTCGT
CGGTCGAATCGGCGTCGACGACGGCTACGCGATCGCCGACGCGGCAGAGGAACACGGCTCGGGCGAGGTACGA
CTCACCCAGCGTCAGAACATCATCCTCACCGACGTGGACGAGGCAAACGTGGACGACCTGCGCGCCGAGCCCG
TCCTCGAGGAGTACAGTCCCAATCCCCACCCGTTCCAGCGCGGTTCGATCGCCTGTACCGGCACCGAGTTCTG
TTCGCTCTCGATCGTCGAGACGAAGAACCGGCAGGTTCGCTTCGCCCCGCTGGCTCAAAGCGAACGTCGAGCTT
CCGGAGGGCGTCGAGGACTTCCACATCCACCTCTCCGGCTGTACCGCGTCGTGCGCCCAGCCCCAGATCGCCG
ACGTCTCCCTGCGCGGGATGAAGACGCGCAAGAACGGCGAACCCGTCGAGGCGCTCGACATCGGCCTCGGGGG
CGGTCTCGGCGAGGAGCCCCAGTTCGCCTCGTGGGTCACCGAGCGCGTCCCCGCCGACGAGGTCCCCGGCGCG
ATCGCGAACCTGCTCGAGAACTTCGCCGAGGCCCGCGAGAGCGAGGCACAGAGCTTCCGGGAGTTCGTCGCCG
ACCGCGACGACGAGGAGCTCGCCGAACTCGTCGAGCCCGAGGAGACCAGCTACGACGACCCGTACATGCACAA
CACGAAGCGCACCTGGTACCCCTACGCCGAGGACGACGACCTCGACTCGAGTCCCGCTCCCGCGCGGGCCGAC
GGCACGCCGATCACCTCGGACGACTAA
>htu:Htur_3039-Ar nitrite and sulphite reductase 4Fe-4S region; K00366 ferredoxin-nitrite reductase [EC:1.7.7.1] (N)
GTGAGCGACGCACCGCACGTCGTCTCCTCGTCGTGGCTCGAGGCCCACCGCGAGTCGGTGACGGTCGTCGACG
TCCGCGAGGAGCGCGATTACGAGCAACTGGGACGGATTCCGGGCGCGGTCAACGTCCCGACCAAGGCGTTCCG
TGATCCGAGCAGTGTCGCCGAGGGGAAACTGCCCGGCGCGGCGGCCTTCGCCGATTGCATGAGCGAGGCAGGG
ATCGAGCGCGGCGATTCCGTCGTCGCCGTCGATGACGAACGCGGCGTCAACGCGGCCCGGTTCCTGCTCACCG
CGACCGTCTACGGTCACGAGGGGGACCTCTACCTGCTCGACGGTGGTCTCGAGGCGTGGCTCGAGGAGATCGA
GGCCGACCTCGAGACGGCTGCACCGGGGACGGAGTCGACGCCGACCCTGACGGCGTACGAGGCCGCCAGACGC
GACGACGCCCCGATCGTCGACCGCGAGGGCGTCGAAGCGGCCGTCGAGGGCGACGCCGTTGTGGTTGACACCC
GAACGGCCGCCGAGTACGATCAGTCGCACATCCCTGGCGCAGTGCAACTCGGTTGGGAGGCCCTGCTCGAGGA
CGAGACCGGCCGACTGAAGCCCGAGGACGAACTCGAGTCGCTGCTCGCCGACCGGGGGATCGCTCCGGATGAT
CGGATCGTCCTCTACTGTAACACCGCGCGGCGGTTGAGCCACACCTACGTCGTCCTGCGGCACCTCGGCTACG
AGAACGTCGCGTTCTACGAGGGGAGCCTGACCGACTGGGTCCGCGCGGAGGCCCCCGAGTGGGATCCCGTCGA
CCTCAAGCGGCAGGTCCGCGCCTACGCCGACGCGGGCGGGTTCGACGCGATGGTCGCGGAACTCGGCGAGGAC
GTCCTCAACCGGTTGAAGCTGATCGGCCTCTACCACCAGAAACAGCGGGGCTACTTCATGCTCCGCACGCGAG
CGCCCGGCGGAATCCTCACCGCCGAGCAGGCCCGCGTCATCGGCGAGGTGGCCGACGAGTTCGCCCGGGCGCC
CGAGGCGTACGGCGGCCCCGACCAGAACCCCGTCTTCGGCGACGGCTACCTCGACGCGACCACGCGACAGGAC
ATCCAGATGCACTGGATTCGGATCGAGGATATCGCTGAGATCTGGGACCGGTACGACGAGGTTGGCCTCGAGA
CGATGCAGGCCTGTGGCAACTCGGTTCGCAACGTCGTCGGCTGTCCCGCCGCGGGGATCGACGCCGACGAGAC
GATCGACGTCCGGCCGACCGTCGAGCGCGTCAGCGAGCGGTTCCTCGGCGACCACCCCTACGCCAACCTGCCG
CGGAAGTTCAAGGTCAGCATCACGGGCTGTCACGAGGACTGCGCCCGATCGGGGATTCAGGATCTGGGGCTGA
CGCCGGCCGTCAAGGACGGCCGCGACGGCTTCGTCGCGCGGGTCGGCGGGGGCCTCTCGGACGGCCCGCGGGT
CGCTAGCGATATCGACCTCTTCGTCGAACCAGAGCAAGTCGACGACCTCGTCGCTGCGATGGCCGACCTCTTC
ATGGACCGGGGCAGCTACCTCGACACCGCCGTCAACCGTCTGCGGTTCCTCGTCGAGGAACTCGGCCCCGAGC
GCTTCAGGGCGGAACTCGAGTCCTACGCCGACTTCGCGTTCGAATCACCTGACGAGGCGCTGACGACCGACTA
CCGGGGCGACCACGTCGGCGTCCACGAGCAGGACGACGGCCGCTCCTACGTGGGGTTGAACGTCCCGACGGGT
CGAATGGGCGGCGACGAGTTCGCGGAACTGGCGCGACTCGCCGACGACCTCGGCGACGGCGAACTTCGGCTGA

CGCCCAACCAGAACGTACTTGTCCCCCACCTCGCGGACGACGCGCTCGAGGCGTTTCTCGACGAACCGGTCGT
CGAACGTTACAGCCCCGATCCCGGGCCGTTCACGCGCGGAATCGTCACCTGTACGGGCCGGGAGTTCTGCAAC
TACGGGATCATCGAGACGAAGAACCGCGCGATCAGGTGGGCCCGCGAACTGGACGACTGGGCCGAGGAAGTCG
GTATCGCGGACGACCACGACGCGATCCGCGTTCACATGTCCGGCTGCTCGGCCTCCTGCGCCCAGCCCCAGGT
CGGCGATTTCGGCCTGCGCGGCGAGGTCTACCGCGACGACCACGAGTCCGGTCGCGCGGCCGACCTCGGACTC
GGCGGGGACCTCGGCGACGACGAATTCATCGACTGGCTCGTCGGGAAGATTCCGATCGACGACGTGCCGGACG
TGATTCGGGAGACAATGCTCGCGTACGAGAGCGACCGCGGGGCGGGCGAATCGTTCGCCGACTGGATCGACCG
GAAGTCCGACGCGGAACTGCGCGAAATCGTTACGGAACGACCGAGGACCGACCCGCCCGCCGTCGGCACGGAG
GTGAGCTGA
>vdi:Vdis_1436-Ar nitrite and sulphite reductase 4Fe-4S region; K00366
ferredoxin-nitrite reductase [EC:1.7.7.1] (N)
GTGTCTTCGTTTAAGTACTCGGACGTTTACAGGATTGTGTATCCAAGGAGGCATGAGGGTATTTATAAGAACC
GTGGCGATAATCCCTGGGTATCAATAAGGATTAGGCAGGGTGTCGGTCGTGACCCAAGCAAGTGGTTTTCGAA
CCAATTAAGGGTTATTGCCGAGGTCAGTAAGGTTTACGGTGATGGTAGGGCCATGCTCGGCACCAGGGGTGAT
ATTGAGATCTTCAAGGTCGATTTCAGACTTTTCGATGAGGTAGTAGCCAAGCTTAAGGAGGTCGGCCTTGACC
CAAGGGACTCCTGCGGAAACTCTGTTAGGAATCCAATACCGTGCACCTCCCAGTTCTGCCCATATGCCCACAT
AAACGCTGAGGCGTTGTCCACATTCATAGGCGATTACTTTAGGTATAGGAGTGAGTATGAGCAACCCTCGTTA
CCTCACAGAATTAAGATATCAATTTCGGCGTGCGAAAGGGCTTGCGCGGTGCCCGTTGCCCAGGACATAGGCA
TTGTGGCCAAGCCCAATGGTAAGTTCGACGTGTACCTGGGCGGTGGAATAGGTGAGCACGCCTTCAGCGCTAA
ATTAGCCTTTACTGACGTTGATGCGGAGGATTTACTACCAATCTGTGTGGGTGTTGCCGAGGTCTTTAGGAGG
TCTGGCGAGAGGAGGGGATTCAAGTGGGTGGTTAAGTTACGTGGTCTTGATAAGGTTAAGGAGGAGGTTATGG
CAATAGCCAGGGAGATCAAACCTAAGCTACCCAAGCCGCCCGATTTGACTCCAAGGTTCATTAAGTCAAGGAT
TGTGAAGGTCCCATACCCAACTGGCTGGGTTACCGCTGATGAATTGATTAAGTTGGCTGATGTGGCTGAGAGG
TATGGGTTTGGCTACGTACTATTATTTAATAACCAAAGCGTCTACATACCAATCCGTGAGGGGGTCAATAAGA
TTGAGGAATTGAACAATTATGAGATTAGGGATCCAGGGCCCATATATCCCGAGGGCCCAATAACCGTTGCATG
CCTAGGGAATGAACTATGCCCACCAGGCCTAATAGACACCACCGGGCTCGGCCGTAAAATACACGTACACTTA
AGTAGGCTCAATGAGCCAATTAGGGTCGGCATTAGTGGGTGCACGCATGATTGCGGCATGTCGTGGGTCTCGG
ACATAGGTTTCGAACCATTTGGTTCTAAGACGAGGATCTTGATAACAATGGGTGGTTCACCGACGGCCCTGGG
CTTTGTCATTGGCACGGTTGACCCAGGGGATGCGGAGCTCGCGGCGCAGGTTTTACTTGAGATGTATAGGGAG
TCAGGTGTTCGTGGGGTTAGGGACTTCGTGACTAAGGTTGGCGTTGATACTATTAGGAGCGAGCTGGTTAAAC
GTGCGCCTTCCTTTAAACCGCCGGATAAGGACATTAGTATTGTTTTTGGGCCTGACTAG
>vmo:VMUT_0162-Ar nitrite and sulphite reductase 4Fe-4S region; K00366
ferredoxin-nitrite reductase [EC:1.7.7.1] (N)
GTGGTTTATCCGAGGAGGCATGAGGGTATTTATAAGAACCGTGGTGATAATCCCTGGGTCTCGATAAGGATTA
GGCAGGGTGTTGGTCGTGACCCAAGTAAGTGGTTTTCAAACCAATTAAGGGTTGTTGCTGAGGTTAGCAAGGC
GTATAGTGATGGTAGGGCGATGCTTGGAACGAGGGGTGATATTGAGATTTTCAAGGTCGACTTTAGGCTCTTT
GATGAGGTTGTGACTAGGCTTAAGGAGGCTGGTCTCGATCCCAGGGATTCCTGCGGTAACTCTGTTAGGAATC
CAATACCATGCACGTCGCAGTTCTGCCCATATGCTCATGTTAATGCTGAGGTTTTGTCGACATTCATAGGTGA
TTACTTTAGGTATAAAAGTGAGTATGAGCAACCATCGCTTCCGCACAGGATTAAGATATCAATATCAGCATGC
GAGAGGGCGTGCGCGGTGCCCGTGGCTCAGGATATTGGTATTGTGGCTAAGTCCAATGGTAAGTTCGACATTT
ACCTGGGTGGTGGTATTGGTGAGCATGCATTCAGTGCCAAGTTGGCTTTTACTGACGTCGATGCTGAGGATTT
ACTTCCAATCTGCATGGGCGTCGCCGAGGTCTTTAGGAGGTCTGGCGAGAGGAGGGGGTTTAAGTGGGTTGTT
AAGTTGCGTGGTCTTGATAAGGCTAAGGAGGAGGTATTGGCAATAGCCAGGGAGGTAAAGCCTAAACTACCCA
AGCCACCTGATCCAACGCCCAGGTTCATTAAATCAAGGATTGTAAAGATTCCATACCCAACCGGCTGGGTTGC
CGCCGATGAATTAATTAGGTTGGCCAATGTGGCTGATAGGTATGGGTTTGGCTATGTATTGCTATTCAATAAT
CAAAGCATATACATACCAATCCGTGAGGAGGTTGATAGGATTGAGGAATTGAGCAATTACGAGGTCAGGGATC
CAGGGCCCATGTACCCTGATGGACCAACAACAGTTGCATGCCTAGGTAATGAATTATGCCCACCAGGCCTAAT
AGACACCACCGGGCTCGGCCACAAAATACATATTCACTTAAGTAAGTTGAATGAGCCAATTAGGGTTGGAATT
AGCGGTTGTACCCACGACTGCGGTATGTCGTGGATCGCGGATATAGGCCTTGAACCATTCGGTTCAAAGACGA
GGATATTAATAACGATGGGTGGTTCACCGACCGCGTTGGGCTTCGTCATCGGCACTGTCGATCCAGGCGATGC
AGAGCTCGCAGTACAAGTACTTCTTGACATGTATAGGAGGTCTGGTGTTCGTGGGGTTAGGAATTTCGTGGTC
AAGGTCGGTGTTGATGCCATAAGGAGTGAGTTAACTAGGCTTGTGTCCTCGTTCAGGTCGCCGGATAAGGACA
TAAACATAGTCTTTGGGCCCGACTGA

>cma:Cmaq_0290-Ar nitrite and sulfite reductase 4Fe-4S subunit; K00366 ferredoxin-nitrite reductase [EC:1.7.7.1] (N)
GTGTTCAAGTATTCTGATGTACATAAGTTGGTTTACCCAAGGAGGCATGAGGGGGTTTACAAGAATCGTGGTG
ATAACCCATGGGTCTCGGTGAGGATTAGGCAGAGTATTGGCCGTGACCCAAGTAAGTGGTATTCAAGCCAATT
AAGGGTTATTGCAGAGGTTAGTAGGAGGTATGGTGATGGTAGGGTAATGCTTGGGACCAGGGGTGATGTTGAG
ATCTTTAAAATCGATTACAGGCTTTTCGATGAAGTTACGGCTAAGCTTAAGGATACTGGACTTGACCCAAGGG
ACTCCTGCGGCAATAGCATCAGGAATCCAATACCATGCCCCAGTAACCACTGCCCACTGGCCCACGTGAATGC
CGAGGCCTTATCAGTATTCATAGGGGATTACTTCAGGTATAAGGGTGAGTATGAGGGGTCGGCATTACCGCAT
AGGATTAAGATATCGATTTCAGCCTGTGAGAGGGCCTGTGCAGTACCTGTGAGCCAGGATGTGGGAATTGTGG
CTAAGCCTAATGGTAAATTCAACATATACCTAGGCGGGGGTATTGGTGAACATGCATTCACAGCTAAATTAGC
CTTCAGTGACGTGGAACTGGAGGATATACTACCGTTAATAGTTGGTGTGGTTGAGGTCTTTAAGAGGAGTGGG
GAGAGGAGGGGTTTTAAATGGTTCATTAAGCTTAATGGATTCGATAAAGTTAAGGAGGAGGTATTGGCAGTGG
CTAGAGAGGTTAAGTCAAAGCTTCCTAAACCACCCGACGTTAAGCCAACATTCATTAACACCAGGGTTATTAA
GATGCATTACCCAACGGGCTGGGTTACCGCCAGTGAATTAGATGAGTTAGCGCATGCTGCTGATGAGTATGGT
TTAGGCTACGTAGTGTTGTTTAATAATCAAAGCATCTACATACCCATTAGGGATAATGTTAAGGAGATTAGGG
GAATTAATGGTGAAATTAAGGAACCAGGACCATACTACCCAGAGGGCCCAATAACAGTTGCCTGCGTTGGTAG
TGAACTCTGTCCGCCAGGTCTAATAGACACAACGAGCCTAGGCCGTAAAATACACGTACATATCAGTGGATTA
AAGGAGCATATTAGAGTCGGCATTAGTGGCTGTACCCATGATTGCGGCATGAGCTGGGTCTCAGACATCGGCT
TCGAGCCTTTCGGCTTCAAAACAAGGATACTGGTCACAATAGGGGGTGCGTCAAGGCAATTAGGCTTTATACT
AGGCACTATCGACCCCAGTGAAGCTGAGTTAACGGCCAAGGTTATATTAAGCATGTATGTGAAGTCTAAGGCA
CCCAACATAAGGGAGTTTGCGGTGAGGACTGGTATTGACGCCATTAGGGATGAGTTAAGGAGGCAAGTACCAT
CATTTCAACCACCCAATGATAGTATTACTGCATTATTTGGACCTGATTAA
>hje:HacjB3_00040-Ar nitrite and sulfite reductase 4Fe-4S subunit; K00366 ferredoxin-nitrite reductase [EC:1.7.7.1] (N)
ATGCCGACCAACGTGGAGGAGTGGAAAGACGAGGTCTACGGCACTGACATCCGTGCCCATATGGAGCGCTTCG
CCGAGGAGGGCTGGGACGCGATCCCCGAGGACGAACACGACGCCTGGTTCGAGCGCTTCAAGTGGTACGGGCT
GTACCACCAGCGCGCCGGCCAGGAGAGCTATTTCATGATGCGGATCGGCCCGCCAAACGGCGTGCTCGAACCC
GGTCAGTTGCGAGCCATCGGCGAGGTCGCGAAGAAATATTCGACCGGACCCGCAGAAAACCCCGAGTTCGGCA
ACGGCTGGGCCGACTTTACGACCCGCCAAGCCATCCAACTGCACTGGATCAACATCGAGGACGTCCCCGCCAT
CTGGGACGAACTGGAGGAGGCCGGCCTCTCGACGCTGCAGGCCTGTGGGGACTCCTGGCGCAACATCGTCGGC
TGTCCGGTCGCCGGCAAGGACAAGCACGAACACGTCGACGCGCTCGATACCATCCACGAACTCCACGACACCT
TCAAGGGCGACGACGATCACTCGAACCTCCCCCGCAAGTGGAAGGTTTCCGTTACGGGGTGTGACGAGGGCTG
TGGACAGGGTGACATCAACGATCTGGGTCTCGAACCCGCCGAGAAGAACGGCGAGACGGGCTTTAACGTCCGC
GTCGGTGGCGGACTCGCGCGCAACGAGCCCCGCTTCGCCCGCGACATCGACGTCTTCGTCACGCCCGAACAGG
CCGCCGACGTCGCCGGCGGTCTCTCGGCGCTGTTTCGCGACCACGGCGACCGCGAGAACCGCTACAACGCCCG
GATCAAGTTCCTCGTCGACGAGTGGGGCCCCGAGAAGGTCCGCGACGTCCTCCAGGAGGAGTACGTCGACTTC
GAACTCGAGACCGCGGGCGAGGACCTCCGGGAGAACTACTCCTATAACGCCGGCACCGCCGACGGCCACGCCG
ACCACGTCGGCATCCACGAACAGAACGACGGGAACTACTACGTCGGACTGAACGTCCTCGTGGGTCGGATGGG
CGCGGACGAGGTGATCGAACTCGCCGACATCGCCGACGAATACGGTTCGGGCGAGGCCCGGATCACCCAGCGC
CAGAACCTGATCCTCACCGACGTCCCCGAGAAGAACCTCGACGAGCTCCGCTCGGAGCCCCTGCTCTCGGAGT
ACAGCCCCGATCCCCACCCGTTCCAGCGCGGGTCGATCGCGTGTACCGGCACGGAGTTCTGCTCGCTGTCGAT
CGTCGAGACGAAAAACAGGCAAGTGCGCTTCTCGCGCTGGCTCGTCGAGAACGTCGAACTCCCCGACGGCATC
GAGGACTTCCACATCCATCTCTCGGGTTGTACGGCCTCCTGTGCCCAGCCCCAGATCGCCGACGTCTCGCTGC
GGGGCATGAAGACCCGCAAGGACGGCGAGCCGGTTGAGGCGCTCGACATCGGGCTGGGCGGTGGCTTGGGCGA
GAACCCCAACTTCGCCGACTGGGTGGTCCAGCGCGTGCCAGCAGACGAAGTGCCCGGCGCGATCGCGAACCTG
ATCGAGGGCTTTGCCGCCGAGCGCGAGGGCGACGAGACCTTCCGCGAGTTCGTCGTCGAGCGCGACGAGGAGG
AACTCGCCGACCTCTGTGAGCCCGAGGAGACCTCCTATACGGATCCGTACATGCACAACACGAAACAGACGTG
GTACCCCTACGCCGACGAGGACGACATGGACGCCAGCCCCGCGCCGACGTCCGCCGACGACTGA
>hje:HacjB3_10150-Ar nitrite and sulfite reductase 4Fe-4S subunit; K00366 ferredoxin-nitrite reductase [EC:1.7.7.1] (N)
GTGAACACGGTAGAGGAATGGAAACGGGAGAAACATCCCCTCGACGCAGTCGAGGACGTCCACGAGTACGCCG
CGGCGGGGCTGTCGTTCGACGAGATCGAGGAGCGGGCGGGCGAGGGCGTCTGGGAGCGCATGAAGTGGACCGG
AATGTACACCCACGGTCGCCAGCGGGGGTATCTGATGATGCGCACGAAGGTCCCCGGTGGTCGACTAACCCCC
GAGCAAGCCGAGGTGATCGGCGAGGTCGCCACGGAGTTCGCGACCGCACCCGAAGAATACGGCGGCAGCGAGC

AAAACGACCTGTGGGGCGACGCCTTCCTCGACGTCACGACCCGACAGGACGTTCAAACGCATTGGATCGAGAT
CGAGGACATCCCCGAGATCTGGGAGCGCTACGACGAGGTGGGGCTGACGACGATCCAGGGCTGTGGCGACGGC
GCGCGAAACGTGCTCGGCTGTCCGGCCGCCGGTCTCGACGATCACGAGTGTTTCGACGCCCAGCCCGTTATCG
ACGCAGTCTCGGACTTTTTCACGGGCAATCGCGAATACGCGAACCTGCCCCGGAAGTTCAAGATGACGATCAC
CGGCTGCAAACACGACTGTGCCCAATCGCAGATCAACGACGCCGGGCTGGTGCCGGCCCGAAAACGGATCGAC
GGCGAACCCCACTACGGCTTTCACGCCCGCGTCGGCGGCGGCCTCTCCGATGGCCCGCGCATGGCCTCGGAAC
TCGACGTCTTCGTGCGCCCGGAGGACACAGTCGAGTTCTGTCGTGCCGTCGCCCAGACGTTCAAGGAGCTCGG
CGACCGGCACAACCGCGGGGTCTGTCGGATGCGCTATCTCGTCCAGCAACTCGGCCCCGAGAACTTCGAGGCG
GCCGTCCGCGATCGGTGTTCGGTCGACTTGCGCGAACGGGGCGAGGGCCTCACCGAGGGCTACCGGGGCGATC
ACGTCGGCGTCCACGACCAGCGAGAGCCCGGATTGAACTACGTCGGGTTCAACGTGATCGCCGGACGAATGGG
CGGCGAGGAGTTCGCCGCAGCCGCCCGCGCCGCCCGCGAGTACGGCACCGAGGAGAGTTCGGTCCGGCTGGCT
ACGGATCAGAACTTCCTCATCACCCATATCCCCGCGGAGAACGTCGAGGACCTACTCAACGAACCCTTCGCCG
CCGACTACCAGCCCGATCCCGGCCCGTTCTCGCGGGGGGCGGTCGGCTGCACCGGCAGCGAGTTCTGTAACTA
CGGCATCATCGAGACGAAAAACCGCGTCAGGCGGTGGGCACGCGAACTCGACGAGCGCATCGAGACGCCCGAG
GACCTCGAGGTGGTGCGAATGCACATGAGCGGCTGTTCGGCCTCGTGTGCCCAGCCCCAGATCGCGGACATCG
GCTTCCGCGGCGAGACCGTGCAACTGGACACGGATGGCGACGGAGATGCCGACGACATCGTCGAGGGCATGGA
CTTCGGCCTCGGGGGGAGTCTCGGCACCGACAACGCGTTTCTCGATTGGGTGGAGACGGCCGTTCCTGCCGAT
GCCGTCATTCCCGCGCTCGAAACGCTCTTTTCGGCGTACGCCGAGGAACGCGAGGACGGGGAGCGCTTCTACG
AGTGGACGCGGCAGGTCGAGAACGACCGCCTGCGGGCGGTGATGCAGGGTGCGGATGCGCCCGTCTCGAAGGG
GGTGGCCGCCGATGACTGA
>hxa:Halxa_1971-Ar nitrite and sulfite reductase 4Fe-4S region; K00366
ferredoxin-nitrite reductase [EC:1.7.7.1] (N)
GTGAGCGACGGTCCGACGGTCGTCTCCCCCGACTGGCTCGAGACGCACCGCGATGACGACGGAGTAGTCGTCG
TCGACGTCCGCGAGGCCCGCGACTACGCGGAACTGGGCCACGTTCCGGGCGCGGTTAACGTCCCAACCGAGGT
GTTCCGCGATCCGAGCAGCGTCGCCGCCGGAAAACTTCCCGCGGCCGACGACTTCGCTGCCCTCATGCGGGAG
GCCGGCATCCGCGAGGGCGACGCCGTCGTCGCCGTCGACGACGCCAACGGCGTCAACGCGGCCCGCTTTCTGC
TCACCGCGATCGTCTACGGCCACGACGGCCCGCTGTACCTACTCGACGGCGGCCTCGAGGCCTGGCTCGAGGC
GGGCGGCAACCTCTCGGACGAGGACCCCGACCCCGAGCCGACGAACTACGAAGCCGAACGGGACGCCGGCGCG
CCGCTGGTCGACCGCGCTGGCGTCGAGGAAGCGGTCGAGGGCGACGCGGTCGTCGTCGACACCCGGACGGCCG
CCGAGTACGACCAGTCTCACATCCCCGGCGCGGTGCAACTGGGCTGGGAGGACCTGCTCGAGGAGTCGGGGCG
GCTGAAACCCGAGGCCGAACTCGAGGAACTGTTGGCCGACCGCGGCATCACCCGCGACGAGCGGATCGTCCTC
TACTGTAACACTGCACGGCGACTCAGCCACACCTTCGTCGTCCTCCGAGACCTCGGCTACGAGAACGTCGAGT
TCTACGAGGGCAGTCTGACGGATTGGGTCCGCGCCGAGGCACCCGAATGGGACCCCGTCGAACTCAAGGAGCA
AGTGCGGGCCTACTCGAGGGGCGGCGGCTTCGAGGCGATGGTCGAGGAGTTGGGCGACGACGTGCTCAATCGG
CTGAAGCTCATCGGGCTCTACCACCAGAAGCAGCGGGGCTACTTCATGCTCCGGACCCGCGCCCCGGGCGGGA
TCCTCACCGCCGAGCAGGCCCGGACGATCGGCGAGGTCGCCGACGAGTTCGCCCGCGCCCCCGACGAGTACGG
CGGCGCCGATCAGAACCCCGTCTTCGGCGACGGCTTCCTCGACGCGACCACCCGGCAAGACATTCAGATGCAC
TGGATCGAGATCGAGGACGTCGCCGAAATCTGGGACCGCTACGACGCGGTCGGCCTCGAGACCATGCAGGCCT
GCGGCAACTCGGTGCGCAACGTCGTCGGCTGCCCCGCCTCGGGGATCGACCCGGACGAGACCGTCGACGTCCA
GCCGGTCGTCGAGCGGGTCAGCCAGCGGTTCCTCGGTGACCACCACTACGCGAACCTCCCGCGGAAGTTCAAG
GTCAGCGTCACTGGCTGTCACGAGAACTGCGCCCGCGCGCAGATCCAGGACCTCGGGCTGACGCCCGCCCGGA
AGGACGGCCGCGAGGGGTTCGTCGCGCAGGTCGGCGGCGGCCTCTCGGACGGCCCGCGGATCGCTAGCGACAT
CGACCTGTTCGTCGACCCCGAGGACGTCGACGATCTCGTCGCCGCGATGGCCGACCTGTTCATGGACCACGGT
AGCTACCTCGATACGGCCGTCAACCGGCTTCGTTTCCTCGTCGAAGAATTGGGGCCCGAAACGTTCCGCGAGG
AACTCGAGACCTACGCCGATTTCACGTTCGAATCTGCCGCGGATGCCGAGCGCCTGACGACCGACTACCGCGG
CGACCACGTCGGGGTCCACGAGCAGGCCGACGGCCGCTCCTACGTCGGGTTGAACGTCCCGACCGGGCGGATG
GGTGGCGACGACCTCGCGGAACTGGCCGCTCTCGCCGACGAGTTGGGCGGCGGCGAACTGCGACTGACGCCCA
ACCAGAACGTCCTCGTTCCCCACCTCGGGGACGATGACCTCGAGCGGTTCCTCGAGCACCCGCTGCTCGAGCG
GTACAGCCCCGATCCGGGCCCGTTCACGCGCGGGATCGTCACCTGCACGGGCCGGGAGTTCTGCAACTACGGG
ATCATCGAGACGAAGAATCGCGCGGTCAAGTGGGCTCGCCAGTTGGACGAGTGGGCCGAAGAAGTCGGCATCG
CGGACGACCACGACGCGATTCGAGTCCACATGTCCGGCTGCGCGGCCTCCTGTGCCCAGCCCCAGTTGGGCGA
CTTCGGACTGCGCGGCGAGGTCTACCGCGACGACTTCGAGTCCGGTCGCGCAGCGGACATGGGGCTGGGCGGC
GATCTCGGCGACGACCAGTTCATCGACTGGCTCGTCGGCAAGATCCCGATCGAGGACGTGCCGAGCGTCATCG
AGGCGACGGTGCAGGCCTACGAGGACGACCGCGAGCCCGACGAGTCGTTTGCCGAGTGGACAAACCGCACGTC

GAACGCGGACCTGCGCGAGATCATCGCCGAACAGCCGGCGCGCGATCCGCCGGCGATCGGGACGGAGGTGAGC
TGA
>mse:Msed_0880-Ar nitrite and sulfite reductase 4Fe-4S region; K00366 ferredoxin-nitrite reductase [EC:1.7.7.1] (N)
ATGAAATATAGTGAGAAATTTAAATCACTCTACCCAGAAAGATTTAAGGGAATTTATTCAAGCAGAGGAGATA
ACGATCTTATCTCCATAAGGATAAGGCAAGGAAAAGAACGGGATCCCTCTAGATGGTGGTACCAGCAACTAGA
GGTCTTAGCCAATATATCTAAGAAAGGAGATGGAAGAGTTCATTTTACAACAAGGGGAGATGTAGAACTTTAC
GGAATTCAGCTATCTGATAAGGAGAAGGTGCTGAGAGAGCTTGAGAGTGTCGAGTTGGATCCAAGGGATTCCT
GTGGGGCATCGGTTAGAAACGTCCTTCCATGTCCATCTTACATTTGCCCCTTTGCTAAGACAGATGCAATTAA
GGCCTCCCTTGAGATAGCTTCCTTCTTCAGACATAATCCAGAGTACGAATTTCCCAAACTTCCAAAAAGGGTA
AAGATCTCTGTCTCGGCATGTGAAAAGGGATGTTCGGTTCCCGTAATAATGGATGTGGGAATTGTAGCCAGAG
GTGAAGATCTCTTCGACGTGTGGGTGGGTGGGGGAATTGGAGACCATGATTTTCAGGGCATCAAGCTATTTGA
GGGAGTTACGCTCGCTGACGCCAAGAGGATCTCCATAGCGGTTACCAACATCCTGAAGCGAGAGAATGAGAAA
AGGGGATTCAAGTGGGTAGTGCAAAAATACGGTTCCGAAAGGGTTAAGGAAATGATCCTGAATGAAATTGAAA
AGATTCAGATAGAGCGGATTAAGGGCATACCATCCTCACACCTCAACGCAAAGTTAATGGTTGTTAGAACTAG
GGGTGGTTGGCTGAATTGGGAGGAAGTCGAAGAAGTTGCCAAGATTGCAAGGGAGAACTTAGGTTTTGTCTCG
CTCTTTAATTCGCAGGCGATATTCATTCCTGTCAGGGAGTTACCTAAAGGTGCAGAGTCCGTTGAATATCCTT
ACCTTAAAAATGTTGTCGAGGCATGCATTGGAGATGATTATTGCCCTCCTGCAATAATTTCTACTACAAAAAT
GGCCGAGGATCTGTTAAAAACTGCCTCAGACAGTAAAATGCGTATTCATATCTCCGGCTGTTCCCACTCGTGT
GGAAGGCATCAGGTTGCGGATTTAGGATTCAGAGCAGTTATGAGAAACGGGAAAAGAGCTCTGAAAATATATG
TAAATGGCAATAATTATAGCATAGGAAAAGTGGTAGGTGAAGTCGACTACGAGGATTACCTTCTAGTTGTGGA
CAAGTTAAAGGGGATAGACTTAGAAAATATTGAAGAAATAAGGAGAAAATTAATGGAAATTCCTTCCTTTAAA
GCTGGTGGTCAGAGTGAGGAATAG
>hla:Hlac_0864-Ar nitrite and sulfite reductase 4Fe-4S domain-containing protein (EC:1.7.7.1); K00366 ferredoxin-nitrite reductase [EC:1.7.7.1] (N)
ATGCCAACGGACGTCGAGAACTGGAAATCGGAGGCGTACGGGCGAGAGGTCCGCGACCGCTTGTTCGAGTTCG
CCGAGGAGGGGTACGACTCGATCCCGGACGACGAGCGGGACGCGTGGTTCGAGCGGTTCAAATGGTGGGGCCT
GTACCACCAGCGGAACGGACAGGAGGGGTACTTTATGATGCGGATCGGGACCCCGAACGGCGTGCTCGAACCG
GGACAGCTCCGAACTATCGGCGAGATCGCCGACGAGTACGCTCGCGGTCCCGGGACGAACCCGATCTTCGGCG
ACGCGTACGCCGACTTCACCACTCGACAGTCGATCCAGCTCCACTGGATCGAACTCTCGGACGTGCCGGCGAT
CTTCGAGAAGCTCGCCGCGAACGGCCTCTCGACGCAGCAGGCGTGTGGCGACTCGTGGCGGAACATCGTCGGG
AACCCGGTCGCCGGCAAGGACGGACAGGAGGTCGTCGAGGCGTGGCCGGTGATCCGCGACCTTAACCAGACGT
TCAAGGGAAACGACGACCACGCGAACCTCCCCCGGAAGTGGAAAGTGTCCGTGACCGGGTCCGCGGACGGCTC
CGGGCAGGGCGACATCAACGACCTCGCGTTCGAGCCCGCGTACAAGTCGATCGACGGAGACGGAGAGGGCGAC
GAGAGCGAGGACGCAGTCGGCTTCAACGTCCGCGTCGGCGGCGGGCTCGCGCGCAACGAGCCGCGGCTCGCCC
GCGACATCGACGTCTGGGTGCCACCGGAGCAAGTATCAGATGTCGCCGGCGGGCTCTCCGCGCTGTTCCGCGA
CTACGGCGACCGCGAGGACCGGTACAACGCGCGGGTGAAGTTCCTCGTCGACGAGTGGGGCGCAGACAAAGTT
CGCGAGACGCTTCAGGAGGAGTACGTCGACTTCGAGCTCGAACCCGCCGGGCGCGACGTTCGCGAGGAGTACA
CCTACAACGCCGGCGAGGGCGAGCGCAACGACCTGATCGGCGTCCACGACCAGAATGACGGCCGGAACTTCGT
CGGGCTGAACGTGCTCGTCGGACGGATGGGTGCTGACGACGTGCTCAACCTCGCCGACCTCGCCGAGGAGTAC
GGCTCCGGCGAGGTCCGGCTCTCGCAGCGCCAGAACGTCATCGTCACCGACGTGCCGGACGACGCCCTTGACG
ACTTCCGCGACGAGCCGCTGCTCGACCACTACTCGCCCGACCCCTCCCCGTTCATGCGCGGCTCGGTCGCGTG
TACCGGTACGGAGTTCTGCTCGCTGTCGATCGTCGAGACGAAGAACCGGCAGGTCCGGTTCGCCCGCTGGCTG
AAGGTCAACGTCGACCTCCCCGCCGACGTCGACGAGTTCCACATCCACCTCTCGGGGTGTACCGCGTCGTGCG
CGCAGCCGCAGATCGCCGACGTGAGCCTGCGCGGGATGAAGACCCGCAAGGACGGGGACCCGGTGGAGGCGCT
CGACGTGGGCCTCGGCGGCGGGCTCGGCGAGAACCCGCAGTTCGCCCGGTGGGTCACCCAGCGCGTGCCCGTC
GACGAGGTTCCGGGCGCGATCGCGAACCTGATCGATAGCTTCGCGGCCGAGCGCGACGAGGGCGAGTCGTTCC
GGGCGTTCGTCGCCCGTCACGACGACGACGAGCTAGACGCCTTCGTCGAGCCCGAGGAGACCGACTACGAGGA
CCCGATGATGCACAACACGAAGCGCACCTGGTACCCGTACGCCGAGAACGATACGATGGAGGACGCGCCGCCG
ACGCCGGCGGACGACTGA
>fpl:Ferp_2220-Ar nitrite and sulfite reductase 4Fe-4S domain-containing protein; K00366 ferredoxin-nitrite reductase [EC:1.7.7.1] (N)

ATGATGGAGATCAAAATTGAGCTTGGCGAAATTGGGAAATTTATAGGTAAGTACTCCTTGGGCAGAGACAACG
GAAATGGATCATTGCACTTTCTCAGGATAAAAATTCCGGCTGGGCAAATTACGAGCGACAAACTTAGAGAGAT
AGCGAAGCTTTCGAGAAAGTACGGAAGAGGCTACGCTGAAATAACCGACAGGCAGGATATTCAGCTCCACTGG
ATAGACAGCGAAAAAGCTTTGGAAATTTTTGAAGAGCTGTACAGTCTCGGCTACACGACCGATCTCTGCGGGC
AGGGATTCAGCGGAGCTTGCTATGGTGACGTAAGAAATATAGTTTCTTGCCCTCTAAGCGGAAAAATTTCCGA
TTTCGACGTTGCAAAGCACGCAAAAGAGCTTACGGAATTCTTCTCGGGAAATCCAGAGTTCATAGATCTTCCG
AGGAAGTTCAAGATCGCTTTCACCGGATGCGGAGGAGATTGCGTAAGGCTCGGAATAAACGATCTCGGAATGT
ACGGAGTCGAGCTCGACGGGAGAAGAGGTTTCGTTCCCTTCGTAGGAGGAAGCATAGGGGCAAGCCATCCCGG
ACCCACTCTCGCTAAAAATCTCGGTGTTTTCGTTCCGGAAGAGAAAGTTTTCGAGTTCGTTAAGGCTGTTGTG
GAAATTCACAGAGACCATTCGAGCAGAGAGAGCAAAGCGAAAGCGAGATTCAAAAATCTCGTAAATTCTTGGG
GGATCGAGAAGCTTAGAAGCGTGATAGAGGAAAAAATTGGGAAGCTTGAAAGAGTTGAGGGAATTGAGTTGAG
ACATGCTGATCACAACGGAAGCGGCGAGCAGGTTAACGGCTTGTACTATTACACTCTGCCTATTATAGGCGGA
GTTCTCAACGCTGAAAAGCTTGAATTCATAGCCGACATGGCTGACAAGCACGGAGAAGGAGAGGTAAGGCTAA
CTCCTCAGCAGAACGTTACGTTTGTGGATGTTAAGGATGTCGAAAAGTTGAGGAGGGATCTTGAGAAGACGTT
CGAGATAAGAGAAGGACAAGTATACTACAACTCCATAGCCTGTGCCTCGAACTTCTGCGGAAAACGAGGGAT
GTTCACGCCAAGGATCTTTTGAGAAAATTGATAGAAGTTTGCGAAAGGAAGGGAGTGAAGGATATCAGAATAA
ACGTCTCCGGCTGCCACAACGACTGCGGCTGCGTTAACGTTGCCGAATTCGGATTTATTGCGAGAATGGTAAG
AAAGAACGGAAAAGTCGTTCAAGGTTACGACATCTACATCGGCGGAGATTTTGCGAAGCTGAAAATGTCGAAG
CCTTATAAGACTGGCTTGACAGCTGAAGAGGTTATTGAGGAGTTCGAAAAGCTAATATCGAGGTGA
>hut:Huta_0176-Ar nitrite and sulfite reductase 4Fe-4S domain-containing
protein; K00366 ferredoxin-nitrite reductase [EC:1.7.7.1] (N)
ATGAGTGCTATCGAATTCATCCCGCCGGCTGCGCTCGGCGACCGAGACGCGGCGATCGTCGACGTCCGCGAGG
CGGCGGCGTACACCGACCTCGGTCACGTCCCGGGCGCAGCCAATATCCCAGTCGATCGGTTCCGGGACCCGAC
CGGGATCGCAAGGGGGATGCTGCCCGATCCGACGGACCTGGCAACGTGGCTCGGTGAGGCCGGAATCAGCCCC
ACCGATCCAGTGATCGCCTACGACGACGACTGTGGCGTCTACGCCGCTCGCTTCCTGGCGACGCTTGCGGCGT
TCGGTCACGATGGCGACCTGTATCTCCTGGACGGGGACTACAGCGTCTACGAACGCGAGGCAGACGTCACGGT
CGAACAGCCGGACGTCGAGCCTGTCGAATACGAACCGGACGACCTCGACGAGACTCTTCTCGCCGACCGCGAG
GACGTCGAGGCGGCAGTCGACGGCGAGGCGATCGTCGTCGACACCCGGACCGAACCGGAGTTCGAGCAGGCCC
ACATTCCCGGGGCCGTCCAGCTCGACTGGAAGGTTTTCGTCGACGACGAGACCGGACGACGACGGAGCGTCGA
AGCGATCGGGTCAACCCTCGCTGAACACGGCCTCGAACCCGATCGACCGGTCGTCCTCTACTGTAACACGGCT
CGCCGACTGAGTTACGTCTTTGCGGTACTCGAAGACCTCGGCTACGGGGACGTCAGGTTCTACGAGGGGAGCC
TCGAAGACTGGCTTCGCACCGAGACCGACGACTGGGACCCCGCCGAGATCAAGCGCCGCGTCCGCGAACACGC
CAACCAGGGACCAGCGGCGGTCAAAGAAGCCCTCGGGGAGGACGCCGCGGCGAAGCTCAAGCTGGTCGGCCTC
TACGGGCAGAAGCAAAGCGGCTATTTCATGTTCCGGACGAAGATTCCGGGCGGTGTCCTGACAGCCGACGCGG
CCAGGGCGCTCGGGACCGTCGCCGAGGAGTACGCCACGCTGCCCGAAGAGCGCGACCCCAAAAGGTCGCCGTT
CGGTGACGGGTACCTCGACGTGACTACCCGTCAGGACGTGCAGTTCCACTGGATCCGAATGGCGGACGTTCCC
GAGATCTGGGAGCTGCTGGATCCGGCCGGCGTCTCGACGTTCCAGACCGGCGGCAACTCCGTCCGGAACGTCG
TCTCGTGTCCGGCAGCGGGCGTCGCCGACGACGAGGTGCTCGACGCCCGCCCGGTCGCCGAGGCGATCACCGA
GGCGTTTCTGGCCGACCGCCGGTACGCCAACCTGCCCCGGAAGCTGAAGGTTAGCGTCAACGGCTGTCGCGGG
GCCTGTGCCCAGCCGGAGATCAACGACCTCGGATTCACCCCCGCGCGGAAGGGCGATCGGGTGGGCTTCAACC
TCGCCGCCGGGGGCGGCCTCTCGGACAGCCCCCGCGTCGCCAGCGACCTCGATGTCTTCGTCGAACGGGACCA
GGTCGTGGACGTGGTGCGAGCCACGGCGGACCTGTTCATCGAACACGGGAGTTATCTCGACACGGCAGTCAAT
CGCCTGCGCTTTCTCGTCGAGGAGTGGGGGGCCGAACAGTTCCGCGAGGAACTACAGCGCTTTGCCCCCTTCG
AGTTCGAATCTGCCGGTGAGGACCTTGTCACGCATCACCACCCCGACCACGTCGGCGTCCACGAGCAGGCCGA
CGGAGACAATTACGTCGGCCTCTCGATCCCGGTCGGCCGGATCGACGGCACGGACTTCCGGGGGATGGCCGAT
CTCGCGGAATCCTACGGGAACGGCGAGATTCGATTGACCACCCAGCAGAACCTCCTGCTTCCGGACGTTGCCG
ATGGGGATCTCGACGACCTGCGGGCCGAACCGTTGCTCGACGAATACTCCCCCGATCCCGGGCCGTTCACACG
CGGCGTCGTCACCTGCACCGGTCGGGAGTTCTGTAATTACGCCCTTGTCGAAACAAAAGCCCGCGCGAAACGC
TGGGCCGCCGAACTCGACGAGCGGGTCGACATCGATCAGGATCGGGTCGGGCTGCGCTTCAGCGGGTGTACCG
CTTCCTGCGCCCAGCCACAGATCGACGACATCGGCCTGCGTGGGGAAACTCGCCAGACCGACGACGGAGTGGA
GAGTGCTGTCGACATCGCCGTCGGCGGCAAACTCAATGTCGATCCACAGTTCGCGACCTGGATCGCACCACGC
GTCCCCATCGAGTCCGCCCCCGATGCGATCGAGCGACTGGTCGCGGTCTTCGAACGCGAAGGGCGCGATTGTG
AGCAACTCCACGAGTTATGTCGACTGGCGGACGATGATCGACTCGCGGAGGTACTGCATCCAGCAGCAATAGA
TCCTGTCGAAGCCGCTGCAAACGGAGGCCGAACCGATGCCGACTGA

>hut:Huta_1327-Ar nitrite and sulfite reductase 4Fe-4S domain-containing protein; K00366 ferredoxin-nitrite reductase [EC:1.7.7.1] (N)
ATGAATACAGTCGAACAGTGGAAACAGGAGAAACACCCCCTCGACGTGATCGAGGACGTCACGGAATACGCAG
AGGATGGCCTCTCCTTTTCCGAGATCGAGGATCGGGCGGGCGACGGCGAGTGGGAGCGCCTGAAGTGGGCCGG
GCTGTACGCCCACGGTGAGCACGACGATTTCTTCATGAAACGGACGAAGGTGCCCGGCGGGTACCTCACGCCC
GAGCAGGCCGAGGTGATCGGCGAGGTGGCCGAGGAATACGCCACCGCTCCAGCCGAACACGGTGGCGAGGACC
AGAACGAAATCTGGGGCGACGCGTATCTCGACATCACGACGCGTCAGGACATCCAGATGCACTGGATCGAGGT
CGAGGACGTCCCCGAGATCTGGGCGACCTACGACGAGGTCGGCCTGACGACGGTCCAGGGGTGTGGCGACTCC
GCGCGGAACGTCCTGGGGTGTCCGGCCGCCGGCCTGGACGGCCACGAGTGCTTCGACGCCCAGCCCGTGATCG
ATGCCGTCAACGAGTTCTTCACGGAGAACCGGGAGTACGCCAACCTGCCCCGGAAGTTCAAGATGACGATCAC
CGGCTGTGCCCACGATTGTGGCCAGTCCCAGATCAACGACGTCGGGCTGACGCCGGCCCGGAAGGAAGTGGAC
GTCTCCGGCGAAGCCGGAGGCTCGTCGGACTCGTCCGACGGTGGGAAGGACATCTACGGCTTCCACTGCAAGG
TCGGCGGCGGGCTCTCCGATGGCCCGCGGATGGCCACGCAGATGGATGTCTTTGTTCCACCCGAAGACGCTGT
CGAGTTCTGTCGTGCCATCGCCCAGACGTTCAAGGAGCTGGGCGATCGGTCGAATCGCGGCGTCTGCCGGATG
CGCTACCTTGTCGAGCAGCTCGGCACCGAGGAGTTCGAGGCTGCGGTCCGACGGCGCTGTAGCGTCGATCTCC
CGGAGAGCGGCGTCGACCTCACCGAGGGCTACGCCGGCGATCACGTCGGCGTCCACGACCAGAAACAGGACGG
CCTGAAGTACGTCGGCTTCAACGTGATCGCGGGCCGGATGAGCGGCGAGGAGTTCGTCGAGGCCGCCCGCGCG
GCGAAGAAATACGGCACCGACGACGCCTCGATCCGGCTGGCGACCGACCAGAACTTCCTGATCACGCACATTC
CGGCCGAGAACGTCGGCGACCTCATGGCCGAACCGTTCGCCCGGAAGTACAGCCCCGATCCCGGGCCCTTCTC
TCGGGGCGCGGTCGGCTGTACCGGCTCGGAGTTCTGCAACTACGGCATCATCGAGACGAAGAATCGGGTCTAC
CGGTGGGCGAAGGCCCTCGACCGCCGCATCGAGACGCCCGACGATCTGGATGTCGTCCGGATGCACATGTCCG
GCTGTTCGGCCTCCTGCGCCCAGCCACAGATCGCCGACATCGGGTTCCGCGGGGAGACGGTGAAGGTCGACGA
TCCCGAGGACTCGACGAATTTCGAGGGCGACGCCATCGTCGAGGGGATGGACTTCGGCCTGGGCGGCGCGCTC
GGGACCGACAACGAGTTCCTCGATTGGGTCGAGACCGCGGTGCCCGCCCGGGCGGTGATTCCCGCCCTGGAGG
AACTGTTCGCGGCCTACACCGAGGAGCGCAACGAGGGCGAACGGTTCTACGCGTGGTGTCGCCGTGTCGAGAA
CGACCGCCTGCGATCGATCATGCAGCGGGCCGACGCGCCCGTCGCGGCCGGGGTCGCCCAGGAGACGGGCGGC
CGGGGGTCGAGTGATGACTGA
>hut:Huta_1758-Ar nitrite and sulfite reductase 4Fe-4S domain-containing protein; K00366 ferredoxin-nitrite reductase [EC:1.7.7.1] (N)
ATGCCCTCGAAGGTCGAGCGATGGAAGGACGAGACTTACGGGATGGAGATACGCGACCACCTCGAACGATTTG
CCGAAGAGGGGTTCGACGCCATCCCGGAGGACGAACGCGACGCGTGGTTCGAGCGGTTCAAATGGTGGGGACT
GTACCACCAGCGGAAGGGCCAGGAGAGTTACTTCATGATGCGCATCGGGACGCCGATGGGCCGGATGACGCCC
GCACAGCTCCGGACCGTCGGCGAGGTGGCCCGCGAGTACGCCACCGGCCCCGTCGACAACCCCGAGTTCGGCG
ACGCCTACGCCGACTTCACGACGCGCCAATCGATCCAGCTCCACTGGATCAAGATCGAAGATATCCCTGACAT
CTTTGACGAACTGGAGTCTGCGGGTCTCTCGACCATCCAGGCCTGCGGTGACTCCTGGCGCAACATCGTCGGC
TCGCCGGTCGCTGGCCGGGATGCCGACGAACTCGTCGATGTTTGGCCCATCGTCGAAGAGCTCCACGACCGTT
TCAAGGGCAACGATCTTTACACGAATCTTCCCCGCAAGTGGAAGGTCGCCGTCACCGGCGACCGTCGCGGTGC
CGGCCAGGGCGATATCAACGACCTCGCCTTCGAACCGGCGGTCAAGGAGATAGACGGCGAGGACATCGCCGGC
TTCAACGTCAACGTCGGCGGCGGCTTGGCTCGCAAGGAGCCCCGGCTGGCCCGCAGCATCGACGTCTTCTGCC
GACCCGAGAACGCTGCCGAGGCCGCCGCCGGCCTCTCCGGACTCTTCCGGGACTACGGCGACCGTGAGGACCG
GTTCAACGCCCGGATGAAGTTCCTCGTCGACGAATGGGGAACCGAGAAGATCCGGTCGGTGCTCCAGGAGGAG
TACGTCGAGTACGACCTCCCGACGGCGGGCCAGGGCCTCCGGGAGGAGTACGACTACAACGCCGGGCGGTCGG
ACGCGCCGGGCGACTACGTCGGTGTCCACGACCAGGCCGACGGCCAGCATTTCGTCGGGCTCTCGGTGCTCGT
CGGGCGGATGGCCGCAGAGGACGTGATCGAACTCGCGGACTTGGCCGAGGAGTACGACTCCGAGATGATCGGC
TTGACTCAACGTCAGAACGTCATCGTCGGTGACATCGCCGAGTCGGACCTGGACGACTTCCTCGCGGAGTCGG
TCCTCGAGAACTACTCGCCCGACCCCCATCCCTTCATGCGTGGCTCGATCGCGTGTACGGGGACCGAGTACTG
CTCGCTGTCGATCGTCGAGACGAAAAACCGGATGGTCCGGTACGCCCGCTGGCTCAAGGACAACGTCCCGGTG
CCCGAGGGCGTTTCGGACTTCCACATCCACCTCTCTGGGTGTACGGCCTCGTGTGCCCAGCCCCAGATCGCCG
ACATCAGCCTCCGCGGGATGAAGACCCGCAAGGACGGCGAGGCGGTCGAGGCCTTCGACGTCGGGCTGGGCGG
TGGCCTCGGCGAGAACCCACAGTTCGCCGACTGGGTGGAGATGCGCGTCCCCGCTGACGAGGTCCCGGGCTAT
ATCGCCAACCTGCTCGAGGTTTACGAGGCCAACCGTGAGGGTCAGGAGTCCTTCCGGGAGTTCGTCACCGACC
GCGAGGAGGACGAACTCTCGGGGCTCGCCGAGCCCGAAGAGACGAGTTACGAGGACCCGTACCTCGGCAACAC
GAAGATGACGTGGTATCCCTACGCCGACGAGGAGGAGATGAGCGCCTCGCCAGCACCGACGAACGCCAGCGGC
GAACCCATTCCCACGGACGATTGA

>hla:Hlac_1407-Ar nitrite/sulfite reductase hemoprotein beta-component ferrodoxin domain protein; K00366 ferredoxin-nitrite reductase [EC:1.7.7.1] (N)
ATGGCCAACAAAAAGGAGGACTGGAAATCGGAGATGTACGGCGACGAGGTTCGCGAGAAGCTACTGGAGTTCG
CGGAGACGGGGTTCGAGTCGATCCCGGAAGACGAGCGGGACGCGTGGTTCACCCGGTTCAAGTTCTGGGGAGT
GTTCCACCAGCGCTCGGGCCAAGAGGGCTACTTCATGATGCGGCTGACGAACGCCAACGGGATCTTGGAGCCC
GGTCAGCTCCGCGCGATCGCCGAGGTCGCCCGCGACTACGCGTCCGGCCCGGTGTCGAACCCCGAGTTCGGCG
ACAGCTGGATCGATCTGACGACCCGCCAGTCCGTCCAGCTCCACTGGATCAAACTCGAAGACGTCCCGGAGAT
CTGGGAGAAGCTCGAATCGGTCGGCGTGACCACCCGGTCGTCGGGCGGCGACACGATGCGGAACATCTCGGGC
AGTCCGGTCGCCGGCAAGGACGCCGACGAGGTGATCGACACGCTCCCGCTGCTCGAACGGTTCCAAGAGGAGA
TCCGCGGCGACGACGACCTCTGTAACATGCCCCGGAAGTTCAACATCAGCATCTCCGGGACGCCCGAGGGCGG
CGCGCAGGACGCGATCAACGACATCGGACTCGAACCCGCGAAAAAAGAGATCGACAGCGGGGAGACGCTCGGC
TTCAACGTGCGCGTCGGCGGCGGGCTCGGCGGGCGAGAGCCCCGCGTCGCCCGCCCGCTGGACGTGTTCGTGA
CCCCGGACGAGGCGTACGACGTCGTCCGCGGGTTCGTCGAATTGTACCACGACCACGGCGACCGGCAGGTCCG
CGCGAAGAACCGCTCGCGCTTCTTCGTCGACGATCACGGCACCGAGTGGATCCGCGACCTGCTCGACGAGGAG
TACGTCGACGCCGATCTGCGGACCGCCGGCGAGGACATCCGCGACGAGTACACGTACAACGCCGGTCGGACCA
CCGAGGACGGGAAGCAGGACTACACCGGCGTCCACGAGCAGAGCGACGGAAGGCGGTACGTGGGCCTCAGCGT
CCCAGTCGGTCGGCTGCCGGCCGAGGAAGCGATCGAGCTCGCCGACCTCGCCGACGAGTACGGCTCCGGCGAG
GTCCGGCTCACGCGCCGGCAGAACCCGATCATCGTCGACGTGAACTCCGACCGCGTGGACGAGCTGCTCGCGG
AGCCGCTGCTCGCGAAACACCGGCCCGAACCGAACCGGTTCGTCCGCGGGGCGATGGCCTGTACCGGCACCGA
GTTCTGCTCGATAGCGCTCGTCGAGACCAAGACCCGAATGGCGACGATGCTCCGCTGGCTCCGCGAGAACGTC
GACCTGCCGGACGACGTCGACCAGCTCAAGATCCACTTCTCCGGCTGCACCGCCGACTGCGGACAGGCCAACA
CGGCGGACATCGGCCTGCTCGGCATGCGAGCGCGGAAGGACGGCGAGATGGTCGAGGCGGTCGACATCGGCGT
CGGCGGCGGGATGGGCGAGGAGCCGTCCTTCGTCGACTACGTCCAACAGCGGGTGCCGGCCGACGAGGCCCCC
GGAGCGATCCGGAACCTGATCGAGGCGTTCGCGGAGCGCCGGGCGCCGGGCCAGACGTTCCGCGAGTGGGCGG
ACGCGACCGACACCGAGACGCTCGCGGCGCTGTGCGAGCCCGAGGAGACGGACTACGTCGACCCGTCGCTCAC
CGACGCGAAGCAGTCGTGGTACCCGTTCGCCGAGGAGGGGACGACGGCGGCGAATCCGGAGGTGGCCTCCTCG
GATGACTGA
>hwa:HQ1116A-Ar nirA; ferredoxin--nitrite reductase (EC:1.7.7.1); K00366 ferredoxin-nitrite reductase [EC:1.7.7.1] (N)
ATGCCGACGGACGTCGAACGCTGGAAATCAGAGGTGTATGGGCAGGAAATACGCGAGCATCTCTTTGAGTTCG
CTGAGGAGGGATATGATACAATCCCGGAGGATGAACGAGATGCATGGTTTGAGCGGTTCAAATGGTGGGGTCT
TTACCATCAGCGAAACGGACAAGAAGGTTACTTTATGATGCGGATCGGGACACCAAACGGTGTCATGAAGCCT
GGTCAGCTTCGCGTAGTCGGTGAAATTGCATCCGAATATGCCACTGGTCCTGGTGTGAATCCAATATTCGGTG
ATGGATACGCTGATTTCACAACGCGACAGTCAATCCAACTTCACTGGATTGAATTATCAGATATCCCGGATAT
CTTCGAGAAGCTTGAAGAAAACGGGCTTTCGACACAACAGGCATGCGGTGACTCATGGCGAAATATTGTTGGA
AATCCTGTTGCCGGTAAGGATGCACGTGAGGTTATCGATGCATGGCCAGTCATTCATGACCTCAATGAGACAT
TCAAGGGGAATGATGATCATTCCAATCTGCCACGAAAATGGAAGGTTTCTGTTACCGGTACGCCTGATGGTTC
CGGTCAAGGCGATATCAACGACCTTGCGTTTGAACCAGCCTACAAAACGATTGATGGTGAGGAGACTGTTGGA
TTCAACGTTCGTGTTGGAGGCGGTCTTGCTCGTAACGAGCCACGATTTGCTCGTGACATCGATGTTTGGGTCC
CTCCAGCGGATGTGTCAACAGTCGCAGGTGGACTTTCAGCGTTATTCCGAGATAACGGCGATCGTGAAGATCG
ATATAACGCCCGTATCAAGTTCCTTGTCGACGAGTGGGGTGCAGACAAAGTTCGTGAGACACTCCAATCGGAG
TATGTTGACTTCGAGTTACACACGGCTGGACGTGACGTTCGAGAGGAATACACATACAATACCGGAGATGGTG
ACCGCAATGACCTGATTGGGGTACATGAACAGCACGACGGATCATACTTTATTGGATTGAATGTCCTTGTTGG
GCGAATGGGAGCATCTGATGTGCTTGAACTCGCTGATCTCGCCTCTGAATACGGATCTGGTGAAGTTCGACTT
TCTCAGCGACAGAATATCATCGTTACGGATATTCCAAAGGATGCTGTTGATGAATTCACGAGTGAGCAACTTC
TCGAAAATTACTCACCAGACCCATCTCCATTCATGCGGGGATCTGTTGCCTGCACAGGTACTGAATTCTGTTC
GTTATCTATTGTCGAGACAAAGAACCGACAAGTTCGATACGCTCGATGGCTGAAAGACAATGTCACGCTTCCG
GGTGACGTTGACGAGTTTCATATCCACCTATCAGGATGCACTGCTTCCTGTGCACAACCACAGATTGCAGATA
TCAGCCTTCGAGGGATGAAAACGCGCAAGAATGGGGAGCCTGTTGAGGCTCTCGATGTTGGTCTTGGCGGTGG
ACTTGGTGCAGAGCCACAGTTCGCACGCTGGGTGACTCAACGTGTTCCTGTTGATGAGATTCCCGGTGCAATC
TCAAATCTGATTGAAACGTTTGCTGCTGAGCGTAACGATGACGAATCATTCCGTGCGTTCATCGATAGACATG
ATAATGATGAGCTTGATTCATTTGTTGCGCCTGAGGAGACCGATTATGAGGATCCAATGATGCACAATACCAA
GCGTACGTGGTATCCATATGCAGAGAATGACGGTCTTGATGCCGGACCATCTGCACCCGCGGACGACTAA

>hwa:HQ2212A-Ar nirA; ferredoxin--nitrite reductase (EC:1.7.7.1); K00366 ferredoxin-nitrite reductase [EC:1.7.7.1] (N)
ATGACGACAAAGAAAGAACAGTGGAAGGATGAGATGTACGGTGACGAGATACGAGAGAAGATTCTTGAGTTTG
CCGAGTCCGGATGGGACTCGATCCCAGAGGATGACCGCGAAATGTGGTTTTCCCGATTTAAATTCTGGGGTGT
CTTCCACCAGCGGTCTGGGCAGGAGAGTTACTTTATGATGCGATTGGCGAACACCAACGGCGTCCTGAACGCC
GAACAACTCCGCGTCATTGGAGAGGTCGCCGGAGAGTACGCTATCGGTCCAGTCTCAAACCCAGAGTTTGGCG
ATGCGTGGATTGACTTCACTACCCGACAATCGATACAACTTCATTGGCTCAATCTTGAAGATGTGCCAGAAAT
CTGGGAAAAAATTGAATCCGTCGGATTATCATCTCGGTCGTCTGGAGGCGACACAATGCGTAATATCTCTGGG
TGTCCGGTTGCTGGGCGAGACAAACATGAGTTCGTCGACGCACAACCGGTACTTGATGAGATATCCACGAAAC
TCCGCGCTGACGATGACCTTGCAAACATGCCTCGAAAGTTCAACATCAGTGTGACCGGTTGTCGTGAGGGATG
TGCGCAGGACTCGATTAACGGCATCGGACTTGAACCCGCAGAAAAGACGATTGACGGCGATACAACATGGGGA
TTCAACGTCCGCGTTGGCGGCGGTCTCGGCGGTCGTGAGCCACGCAAAGCACGGAGTCTTGATATCTTTGTCA
CGCCCGACAACGCATATGATGTTGTCAGAGGATTTGTCGAACTGTACTTTGAGACCGGAGATAGAGAAAACAG
ACAAAAAAACCGAAGTCGCTTTTTTGTTGATGATTGGGGGACGGAAAAGATCCGTGACCAACTCCAAGAGTCA
TACGTTGACTTTAATCTCCGGCGCGAAGGAGCGGATTGTCGCGATGAATATACGTATAATGCCGGTAAGTCAC
CAACAGACGGCAAACATGATCACATCGGCGTTCATGAGCAAACGGACGGCACCTACTACGTCGGTCTCTCGGT
GCCAATTGGGCGGCTCACTGCTGGTGAGACCGTCCGACTTGCCAATCTAGCGGAGATACACGGGTCTGGAGCA
ATGCGACTCACGCGACGACAAAACCCGCTGATCTTAGATGTCCCCGAGTCAGAACTCGACGCGCTCTTGACGT
CTGATTTGCTTGAGACTCACAGTCCCGAACCAAATGTGTTCACGCGAGGTGCAATGGCGTGCACAGGCACGGA
GTTCTGCTCGCTTGCGCTGACGGAGACGAAAGCTAGAATGGCAACGCTGCTCCGATGGCTGCGGTCGAATGTG
GACCTACCAGATGACATCGAACAGATCAAAATTCATTATTCCGGTTGTACGGCTGACTGTGGTCATGCAACGA
CTGCCGACATCGGATTTCAGGGAATGCGAGCGCGGAAAGACGGAGAAATGGTTGAGGCGATTGATGTTGGCGT
TGGCGGCGGCATCGGTCCTGACCCCTCTTTCGTCGAGTGGATCAGACAGCGTGTGCCGGCAGACGAGGTTCCT
GGACTCTTGCGGAACCTTCTTGAAACGTTCGCAGCACACCGCGAAAAGGGACAAACGTTTAGACAATGGGTTG
AGGCGACCCAGGAGGAGGTGCTTATCGAAGCCGCCGAGCCGGAAGAAACCGATTACGAGGACCCGTGTCTCCA
CGACGCAAAAGGATCATGGTACCCGTTTGCAGATAGTGAGGGACCTGCTCCAACTGCCCCCGACGACACGTCA
CTAGCGACTGAAAGCGATGACTGA
>afu:AF0164-Ar nirA; ferredoxin-nitrite reductase; K00366 ferredoxin-nitrite reductase [EC:1.7.7.1] (N)
ATGGCAATTGAAGTCGAGCTTCACAAACTGCGGGAATGGATGTGGAAATACTCCCTCGGCAGAGATGGGGGCA
ATAATTCTGATCACTTTCTTCGTATAAAAATTCCGGGCGGGATAATTTCACCGGATCGGCTGAGAGGTGTTGC
TGAGCTATCAGATAAATATGGGAGAGGTTATGCTGAGATTACGACGAGGCAGGACATACAGCTTCACTGGATC
GAGGCTGAGGATGCTCTGGAAATCTTCGCTGAGATGGAAAAGCTTGGCTTCACAACAGACCTGTGCAGTCAGG
CTTTTGCCGAAACATGCCACGGTGATGTGAGAAACGCCGTCTGCTGCCCTCTGTCTGGGAAAAAGGGATACGA
TGCTTACTGGCTTGTTAGGGAGATTACGGATTTCTTCTCAGGCAACCCGGATTACGTCAGCCTTCCGCACAAG
TTCAAGATTGGCATCTCTACTTGCGAGAGCAACTGCATAAGGGAGGAGGCGCAGGACTTGGCAATGCAGTGGA
ACGGCGAGGGGTTTGTACCGCTTGTTGGGGGAGGACTGGGGGCTTCGAAGCCCGGAGTCAGGAAGGCAAAGAG
CTTGAGAATCGTTGTTTACGAGGAGGATGCCTTCGATTTTATAAGGACGGTCATAGACATCTACAAAGACTTC
AGAGCTGAGAGAAAAGCAGAGGCAAGGTTCAAGAACTTTTTTGAGACTGCTGGATGCGAGTGGCTGAGAAGAG
AAATTGAGCTCAGAATGGGTGTTAAATTCGATTTAGCTGATGATTTCAACTGCAATCCGCCATCAGAGCACAC
TGAGGGCTTGCAGGATGATGGCAGCAACTACTACACCCTTCCTGTGTTCGCAGGTATCCTCGACTCGGAAAGG
CTGAGCTTCATTGCTGAGGTTGCTGAGGAGTTTGATGGAGGAATCAGGCTCACACCATGGCAGAACGTTGTTT
TCACCGATATTGACGACTTGAAGGCTTTGAAAGAGAGGCTTTCCGAGCAGTTTGACCTCAGTGCTCCAAGAAT
GCACATCGCCTGTGCATCGAACTTCTGCGGTAAGACGCTGGTGCACGCAAAGGACGTTCTCAGGCACATTCCA
GCGAGCGATAGATTTGTGGGGGTGAGCGGATGCAGCAACGCCTGTGCCTGCCATCCTCTTGCTGAAATCGGAC
TTTGTGGGAAGGTTAAAGCAGGAAAGCAGCTCTACGACGTCTTCATTCACGGCTGGAAGGAGGCGGAGAACCT
GAGCGTTGAGGAGGCGGTTGAGCTCATTAAAAAACATCTTGGTGATGGATATGGAGCTAAAGCTGATTGA
>nph:NP4004A-Ar nirA_3; ferredoxin--nitrite reductase 3 (EC:1.7.7.1); K00366 ferredoxin-nitrite reductase [EC:1.7.7.1] (N)
ATGACGCTGGATATCGACACTATCGCTCCCGAAGCGCTTGCGGACGAGCGGGAGCAGTACACCATCGTCGACG
TTCGGGAGCCGAGAGACTACCGCGAACTCGGGCATATCCCGGGAGCGGCCAACGTTCCGTTCGAGCGGATTCG
GGACCGCAGCACCGACGCCGCGGGCCGACTCCCGACACCGGCGACGGTTCGACAGCAGCTATCGGCTGCCGGT
GTCGAGCAAGGGGACCCACTCGTCGCGTATGACAACAGCCGCGGCGTTGAGGCGGCTCGATTTCTGCTAACGG
CGGCGGTTTACGGCCACGACGGACCGCTGTATCTCCTTGAGGGTGATTTCGATGTCTGGCAGCACGACAACGA

GACGGAACGCGGCCCGGGGCCGGAGGGTTCCTCGGAGTACGGCGCGGTGGCGCTCGATGAGGACGCCATCGTC
AACCGTGCGACCGTCGAGGCGGCCATCGACGCGGACGAAGGGGCCGTACTGGTCGATACGCGAACGGCCGGCG
AGTACGAGAGCGCCCACCTGCCGGGGGCAGTGCATCTCGGCTGGGAGGCCTTCGTTGACGCCGAAACCGACCG
ACTCAAGCCGGAGTCGGAGCTGCGGACGCTGCTGGAGAGCCGCGGCCTCTCGTCCGACGACGAGATACTGCTC
TACTGTAATACAGCCCGGCGGCTTAGCCACACCTACGCCGTTCTCTCGTCGCTTGGCTACGATGAGGTCCGAT
TCTACGAGGGCGAACTTACCGGTCTGGTGCGGGCGAACTCGCCCGCGTGGGACCCCCAAGAGCTCTACCACTC
AGTGAGGGCGGTCGCCCCGGAAGGGTTCGACGCACTGCCGGCCGAACTCGGTGACGATATCTTCAGCCGCCTC
CACCTGCTCGGACTCTACACGACCCGGCAGGACGGCTACTTCATGCTCCGAACGAAGATTCCCGGCGGGCAGC
TGACAGCCGAACAGGCTCGCACTGTCGGGCGAGTCGCCGACGAGTTCGCCACCGCGCCGCCGGAACACGGCGG
CAGCGAGCAAAACCCCGTCTTCGGTGACGGCTTCCTTGACGTGACAACACGGCAGGGCATCCAGATGCACTGG
ATTCGCATCGAGGACATGCCCGAAATCTGGGACCGGTTTGAGTCGGTCGGGCTGACGACGATTCAGGCAAGCG
GGAACACGCTCCGGAACGTCGTCATCTGCCCGGCAGCGGGAGTCGGCCACGAGACGGTCGACGTTCGCGGGCT
CGCGGAGGATGTCGCCGACGCCTTCGAGGGGAGTACCCGGTATGCGAACCTCCCTCGGAAGTTCAAAGTCAGT
CTCTCGGGCTGCCACGAAAACTGTGGCCGCGCGGAGCTACAGGACCTCGGATTCGTCCCGGCGGTCAAGGACG
GCCGCGACGGCTTTGCTGTCAAGGTCGGCGGCGGCCTCTCCGACGGGCCGCGAGCGGCGACCGACCTCGGCGT
TTTCGTTCCACCGGAGCGAGTCGTCGACCTCTCGCTTGCGGCTGCGGACCTGTTTATCGACCACGGTGCGTAC
CTCGATACTGCAGTCAATCGCTTGAAATACATCGTCGACCGCTGGGGGACCGACCGATTCCGCGAGGAACTCG
AATCCTACGTCGAGTTCGAGTTCGAGCCCTACGACGAGCTGTTGACAACCGAGTACCGCGGCGACCACGTCGG
TATCCACGAGCAAGCCGATGGGAACCACACGGTTGGGCTGAACCTCCCGACCGGTCGAATCTGCGGCGATGAG
CTACAAACGCTCGCCGACATCGCCGAGCGGTACGGCAGCGGCGAGGTCCGGACGACGGCGAACCAGAACCTCG
TCGTCCCCGGCGTCCGTGGCGAGGTGCTCGAATCGATGCTCGGCGAGCCGCTGCTTTCGTCGTACAGTCCCGA
CCCGGGGCCGTTCTCGCGGGGTATCGTCACCTGTACCGGCCGGGAGTTCTGCAAATACGGCGTTATCGAGACA
AAGAGCCGCGGCTATCGGTGGGCGAAGGAACTCGACGCATGGCTGGACGACGCCGATATTCCAGAGTCCGCTG
TCCCTGAAGCCGTTCGTATTCATATGAGCGGCTGTTCGGCGTCGTGTGCGCAGCCACAAATCGGCGACATCGG
GCTGCGCGGCGAAGCCTATCGCGACGAGACCCAAGCCGCACAGGCGGTCGATGTCGGCCTCGGCGGCGACCTC
GACCGCGACCAGTTCGTCGACTGGGTCTCCGGCCGCATACCGGTTGGCGAAGTGCCGACAGCGGTAAAACGGG
TCCTTCGGCGGTTCGTCGAGACCCGACGGGACGGCGAGACGTTCGCCGAGTGGGCCGACCGGACCGACACCGA
ACGACTAGACCGAATCGTCACGACAAGCACACACCCACAGGAGGCAGACTGA
>nph:NP1146A-Ar nirA_2; ferredoxin--nitrite reductase 2 (EC:1.7.7.1);
K00366 ferredoxin-nitrite reductase [EC:1.7.7.1] (N)
ATGCCGAGCAAGGTCGAAGCCTGGAAAGACGAGGTCTACGGAACGGAAATCCGTGACCACCTCTTCGAATTCG
CCGAGGAGGGCTGGAGCGCCATCCCCGAGGACGAACGTGACGCGTGGTTCGAGCGGTTCAAATGGTGGGGGCT
GTACCACCAGCGGAAGGGCCAAGAGAGCTACTTCATGATGCGTATCGGGACGCCGAACGGTCGGCTGACACCC
GACCAGCTTCGGGTCGTCGGCGAAATCGCCGACGAGTACGCCCGCGGACCCGTCGACAACCCCGAATTCGGCG
GGGCATACTGTGACTGGACGACGAGACAGGCGATTCAGCTCCACTGGATTCAGCTGCAGGACGTCCCCGAAAT
CTTCGACAAGCTGGAGGCGAACGGCCTCTCGACGATGCAGGCCTGTGGCGACTCGTGGCGGAACATCGTCGGC
TCGCCGATGACCGGCCGGGACGCCAAAGAGCACATCAACGCGTGGCCGGTTATCAAGGAGCTCAACGAGACGT
TCAAGGGCAACGACGACCACTCCAACCTCCCTCGCAAGTGGAAGGTCGCTGTCACGGGCGACACCCGTGGGTC
GGGACAGGCCGAAATCAACGACCTCGGCTTCGAGCCCGCTGTCAAGGAAATCGACGGCACGGAGACGAAAGGG
TTCAACATCCGCGTCGGCGGCGGCCTCTCCCGGAAGGAGCCGCGCTTTGCGCGCGATATTGACGTCTTTGTCA
CCCCCGGGGAGGTCGCCGACGTTGCCGGCGGGATTTCGGCGCTGTTCCGCGACCACGGCGACCGTGAGGACCG
CTTCAACGCCCGCATCAAGTTCCTCGTCGACGAGTGGGGGACCGAGAAGCTCCGGCGCGTGCTGCAGGAAGAC
TACGTCGACTTCGAGCTGAAGACGGCCGGCGAGGACCTCCGCGAGCAGTACAGCTACAACGCCGGACGGAACG
ACGAGGTCGGCGACTACGTCGGTGTCCACGAGCAGACGGACGGCAACTACTTCGTCGGTCTCTCGGTGCTTGT
CGGCCGAATGGGAGCACAAGAGGTCATCGAACTGGCCGACCTCGCCGACGAATACGGCTCGGAGCTCATCGGC
CTCACACAGCGACAGAACATCGTCGTCGCCGACATCGCCGAGGAGGACCTCGATGCATTCCTCGAGGAGGACC
TGCTGGAGTACCACTCGCCGGACCCCCATCCCTTCCTGCGCGGGTCTATCGCCTGTACGGGCACCGAGTTCTG
CTCGCTGTCCATCGTCGAGACGAAAAACCGAATGGTCCGCTATGCCCGCTGGCTGAAGGACAACGTCGATGTC
CCCGAGGGCGTCTCCGAGTTCCACATCCATCTGTCGGGCTGTACGGCCTCCTGTGCCCAGCCGCAGATTGCCG
ACATCTCCCTGCGCGGTATGAAAACCCGCAAGGACGGCGAGCCGGTCGAGGCCTTCGACATCGGGCTCGGCGG
CGGCCTCGGCGAGAACCCGCAGTTTGCCGACTGGGTCGAGATGCGCGTGGCCGCCGACGAAGTGCCCGGCTAC
ATCCAGAACCTCCTTGCCGTCTACGAAGCCGAACGGGAGGGCGACGAGTCGTTCCGGGAGTTCATTGCCCGCC
ACGGCGAAGACGAGTTGGGCGGCCTCGCGGAGCCGGAGGAGACCTCCTACGAGGACCCGTACCTCGAGAACAC

GAAGATGACGTGGTATCCGTACGCCGAGGAGACGGAGATGGACGCCTCCCCGGCCCCGTCGCCGGCTGACGAC
TAG
>nph:NP4224A-Ar nirA_1; ferredoxin--nitrite reductase 1 (EC:1.7.7.1); K00366 ferredoxin-nitrite reductase [EC:1.7.7.1] (N)
ATGCCGCATAAAAAAGAAGCGTACAAGGAGGAGTTGTACGGTGACACAGTACAGGAGAAAATCCTCGAGTTCG
CCGACCGCGGGTTCGATTCTATTCCCGAGTCGGAGCGCGACGAGTGGTTCTGCCGGTTCAAATTCTGGGGGCT
CTTCCACCAGCGCTCTGGACAGGAAGGCTACTTCATGATGCGCTTGACCAACTGTAACGGTCGGCTCGACCCG
GGACAGCTTCGAGCAATCGGCGAAGTCGCCGACGAATACGCGACAGGTCCCGAAACGAACCCCGAGTTCGGCA
ACGGGTGGATTGACCTGACGACGCGGCAGTCGGTACAACTCCACTGGATACGGCTCGAAGACGTTCCGGATGT
GTGGGACAAACTCGAAGCGGCCGGTGTTACGACCCGCTCGTCGGGCGGCGATACAATGCGGAACATCGTTGGG
TGCCCGGTTGCCGGCAAGGCCGACGACGAAATCATCGACACCCAGCCGATTCTTGACCGGTTCCAAGACGAGA
TCCGGGATGATGACGCGCTCTGTAATATGCCGCGGAAATTCAACATCTCTATTACAGGGACGCCGGAGGGCGG
CGCACAGGACTCGATCAACGATATCGGACTCGAACCAGCACAAAAAGAGATTGACGGCGAGGAGCAGCTCGGA
TTCAATGTTCGCGTCGGCGGCGGTCTGGGCGGCCATGAGCCGCGGGTTGCGCGTCCGCTCGACGTCTGGGTCG
CCCCCGACCGTGCGGTCGACATCGTGCGCGCTTTCGTCGAACTGTACCACGATTACGGGGACCGCGAAAACCG
CAGCAAGAATCGGTCACGGTTCTTCGTCGATGATAACGGACCCGAATGGATCCGAGAGACACTGCAGGCGGAG
TACGTCGACTGGGAACTCAAAACCAGCGGCGCGGATATGCGGGATAGATACACCTACAATGCCGGGCGTCCCA
GCGAAGACGGCGCATTCGACTACGTCGGCGTCCACGACCAAAAAAACGGGAAGAAATGGGTCGGTCTCAGTGT
TGCCTGTGGACGGCTCGAATCACAGGAAGCGATTCGGCTTGCGGAACTCGCCGCGGAGTACGGTTCGGGTGAA
GTTCGGCTAACGCGCCGACAGAATCCGCTGATTATGGATGTCGACCCGAACAGGGTTGATGACCTGCTCCGAG
AGCCCTTGCTGCACAAACACACGCCCGAACCAAACCCATTCGTCCGCGGCGCGACCGCCTGTACCGGCACGGA
GTTCTGTGGACTCGCCCTAACCGAAACGAAGGCCCGAATGGCCCGGATGCTCCGGTGGCTGCGGGACAACGTC
GACCTGCCCGAGGACGTGTGCCAGCTTAAAATTCATTTCTCCGGATGCACGGCCGACTGCGGACAGGCCAACA
CCGCCGACATCGGCTTGCTCGGGATGCGGGCCCGGAAAGACGGTGAGATGGTCGAAGCGCTAGATATCGGCCT
CGGTGGCGGTATCGGTGAGGACCCATCGTTTGTCAACTGGATTCGCCAACGCGTCCCCGCCGACGAAGTGCCT
GGCGCGATCAAAAACATCGTCGAAGCGTACGCGGCCCACCGCTTTGCGGGACAGACGTTCCGTGAGTGGGTTG
CTGCGACGAGCGACGAGCAACTGATTGAGCTGTGTGAGCCTGTCGAGACGAGCTACGACGACCCGTGTCTCTA
CGACGCCAAGCAGTCGTGGTATCCGTTCGATGCAGAAAAGCAGGCCACCAAGGGCGTCCAAGGTGCTGACGAC
TAG
>hhi:HAH_3043-Ar nirA2; ferredoxin-nitrite reductase (EC:1.7.7.1); K00366 ferredoxin-nitrite reductase [EC:1.7.7.1] (N)
ATGGCACATAAAAAGGAGGAGTACAAGGCGGAGCTGTACGGCGACGAGGTACGGGAGAAGTTAGAGGAGTTCG
CCGAGAAGGGCTGGGAGTCGATTCCCGAGGACGAACGGGAGAAGTGGTTCTCGCGGTTCAAGTTCTGGGGGGT
GTTCCACCACCGCGGCGGCCAGGAGTCGTATTTCATGATGCGGCTGACCAACTGCGGCGGCGTCCTGGAGCCC
GACCAGCTTCGGGCCATCGGCGAGGTCGCTCGGGACTACGCGAAGGGACCGGCCGAGAACCCCGAGTTCGGGA
ACGGCTGGATCGACCTCACGACGCGACAGTCCATCCAGTTGCACTGGCTCAAGCTCGAAGACATCCCGGAGAT
CTGGGAGAAACTCGAAGCCGTCGGCGTCTCCTCGCGCTCGGCGGGCGGGGACACGATGCGGAACATCTCCGGC
TGCCCGGTCGCCGGCAAGGCCGAGGAGTACGTCGAGTCCCGCCCGATTCTGGACGAGATTCAGGAGACCATCC
GCGACGATAACGACCTGGCCAACATGCCCCGGAAGTTCAATATCTCGGTGACGGGGTGCAAGCAGGGGTGTGC
CCAGGACAGCATCAACGACATCGGGCTGGAGCCGGCCCACAAGTTCATTGCTGGCGAGGAAGTCGAGGGGTTC
AACGTCCGCGTCGGCGGCGGCCTCGGCGGCCGCGAACCGCGCGAAGCCCGTCCGCTCGACCTGTTCATTCGGC
CCAAACACGCCGTCGAGACCGTGCGGGCCTTCGTCGAGTACTACCACGAAGCGGGCAACCGCCAGAACCGCTC
GAAGAACCGCGCGCGCTTTTTCGTCGATGAACACGGTACTGACGCCATCCGGGAAGAACTCGACGAACGGCTT
GATTTCGAGTTCGAAACCGCCGGGACCGACTTCCGCGGGGAGTACACGTACAACGCCGGCAAGCACACGGAGT
ACGGTGCCCACGACCACGTCGGCGTCTACGACCAGAAAGACGACAAGAACTACGTCGGCCTCTCGGTGCCGGT
CGGCCGACTCTCCGCCGAGGACGCCATCGAACTCGCGGATCTCGCCGACGCCTACGGCTCCGGCGAGGTGCGA
CTGACCCGCCGGCAGAACCCGCTCATCATGGACGTACCCGACGGGAATCTCTCGAACCTCCTCAACGAGCCGC
TGCTGGAGAAACACTCGCCCGAGCCGAACCCGTTCGTTCAGGGCGCGATGGCCTGTACCGGGACGGAGTTCTG
CTCGCTCGCGCTCACGGAGACGAAGGCGCGGATGGCCCGCTTGCTCCGCTGGCTCGGTGACAACGTCGACGTG
CCCGACGACGTGGACCGCATCAAGATGCACTTCTCGGGCTGTACGGCCGACTGCGGCCAGGCGATGACGGCCG
ACATCGGCTTACAGGGGATGCGCGCCCGCAAGGACGGCCAGATGGTCGAAGCCGTCGACGTGGGCGTCGGCGG
CGGCATCGGCGAGGAGCCGACGTTCATCGAGTGGGTCCGCCAGCGCGTCCCTGCTGACGAGGTGCCGGGCATG
ATCGCCAACATTGTCGAAGCCTACGCCGCGCTTCGCTCGGAGGGTCAGACCTTCCGCGAGTGGGTCGACGCCA
CCGGTCACGAGACCATCGTCGAACTGGCCGAACCCCACGAGGTCCAGGGGTACACCGACCCGTGTCTAGCCGA

CGGCAAGCAGTCGTGGTATCCATTCGACGACGGCGAAAGCCCGGCCCCGACCGACGCCGAGGGCCAGCCGATT
TCGGCGGACGACTGA
>hma:rrnAC2992-Ar nirA2; ferredoxin-nitrite reductase; K00366 ferredoxin-nitrite reductase [EC:1.7.7.1] (N)
ATGCCCACGAAAGTCGAGAATTGGAAGGACGAGGTTTACGGTAACGAGATCCGGGAGCACTTGATGGAGTTCG
CAGAGCAGGGCTGGGATGCCATCCCGGAAGACGAACACGACGCCTGGTTCGAGCGCTTCAAGTGGTGGGCCT
GTACCACCAGCGCTCCGGCCAGGAGTCGTATTTCATGATGCGTATCGGGACACCAAACGGCGTCATCAAGCCC
GGCCAGCTTGAAGTTATCGGCGAAGTCGCAAAGGAGTACGCAACTGGTCCCGTCGATAATCCGGAGTTCGGCG
AGGCCTACTGTGACTGGACGACCCGTCAGAGCATCCAGCTCCACTGGATCAAACTGGAGGACATCCCGGAAAT
CTTCGAGAAGCTCGAAGCCGTCGGCCTGGGCACCCAGCAGGCCTGTGGTGACTCCTGGCGGAACATCGTCGGC
TGCCCGGTTGCCGGCAAGGACAAGCACGAGCACGTCGACGCCTGGCCCGTTGCCGAGGACCTCCACGAGACGT
TCAAGGGCAACGACGACTACTCGAACCTCCCGCGCAAGTGGAAGGTCGCCATCGCCGGCTGTGACGAGGGCTG
TGGCCAGGGCGACATCAACGACCTCGCTTTCGAGCCCGCTGAGAAGGACGGCGAACTCGGCTTCAATGTCCGT
ATCGGCGGCGGCCTCTCCCGCAAGGAACCGCGTCTCGCCCGCGATATCGACGTGTGGGTCCCGCCGGAGCAGG
CCGCTGACGTGGCCCACGGCATGTCTTCGCTCTTCCGTGACTACGGTGACCGCGAGGACCGCTTCAACGCTCG
CATCAAGTTCCTCATGGACGAGTGGGGGCCCGAGAAGATGCGTTCGGTCCTGCAGGAGGAGTACGTCGACTTC
GAACTCCCGACTGCGGGCGAGGATATGCGCGACCAGTACAGCTACAACACCGGGTCCCAGGACGGACACAACG
ACCACGTCGGGATTCACGAGCAGAACGACGGCAACTACTACATCGGCCTGAACGTGCTGGTCGGCCGCATGGG
TGCCGACGACGTGCTCGAACTCGCCGAACTCGCCGACGAGTACGGCTCCGGCGAGGTCCGACTGACCCAGCGT
CAGAACATAATCGTCACCGACGTCCCCGAGGAGAACCTCGACGCGTTCACCAGCGAACCCCTGCTGGAGAACT
ACTCCCCGGACCCGTCGCCGTTCATGCGCGGTTCCATCGCCTGCACGGGCACGGAGTACTGCTCGCTCTCTAT
CGTCGAGACGAAGAACCGCCAAGTGCGGTACGCCCGCTGGCTCAAGGACAACGTCGAACTGCCCGAGGACCAC
AAGGACTTCCACATCCACCTCTCGGGCTGTACGGCCTCCTGCGCCCAGCCCCAGATCGCCGACGTGTCCCTGC
GCGGGATGAAGACCCGCAAGGACGGCGAACCGGTCGAGGCGCTCGATATCGGTCTCGGCGGCGGCCTCGGCGA
CGACCCGCGCTTCGCTGACTGGGTGGAGATGCGCGTCCCGGCCGACGAAGTCCCCGGCGCGATCAAGAACCTC
GTCAACAACTTCGAGGACGCCCGCGAGGGCGGCGAGACCTTCCGTGACTTCGTTGCGGACCGCGACGAGGAGA
CGCTGGCCGACATGGTCGAACCCGAGGAGACGGACTACCACGACCCGTATATGCACAACACGAAGATGACGTG
GTACCCCTACGCAGAAGACGACGATATGCAGTCCTCGCCCGCGCCGACTGACGGCTCTGGTGAGCCCCTGCCC
TCCGACGACTAA
>hvo:HVO_1788-Ar nirA1; ferredoxin--nitrite reductase (EC:1.7.7.1); K00366 ferredoxin-nitrite reductase [EC:1.7.7.1] (N)
ATGCCCACGGACGTCGAACGCTGGAAGTCGGAGGTCTACGGTAACGAGATACGAGAGCACCTGCTGGAGTTCG
CTGAGCAGGGCTGGGACTCCATCCCGGAAGACGAGCGAGACGCCTGGTTCGAGCGCTTCAAATGGTGGGGACT
GTACCACCAGCGCAACGGCCAAGAGTCGTACTTCATGATGCGCATCGGAACGCCGAACGGCGTGCTGACGCCC
GGCCAGACCGAAGTCGTCGCGGAGGTCGCCGACGAGTACGCCCGCGGCCCCGCCGAGAACCCCATCTTCGGCA
ACGGCTACGTCGACTGGACGACCCGCCAGTCGATTCAGCTCCACTGGATTAAGCTGGAGGACATCCCGGAAAT
CTTCGATAAGCTCGAATCGGTCGGGCTGTCGACCCAGCAGGCCTGCGGTGACTCGTGGCGGAACATCGTCGGC
TGTCCCGTCGCCGGCAAGGACAAACACGAGCACGTCGACGCGCTCCCCGTCGCGATGGAGCTCAACGAGACGT
TCAAGGGCAACGACGACCACTCGAACCTCCCGCGCAAGTGGAAGGTCTCCGTCACCGGCTGCCGCGAGGGCTG
CGGTCAGGGCGACATCAACGACCTCGCGCTCGAACCCGCGACCAAGGAAATCGACGGCGAGGAGACGAAGGGC
TTCAACATCCGCATCGGCGGCGGCCTCTCGCGGAACGAACCGCGGCTCGCCCGCGACATCGACGTGTTCGTGA
CGCCCGAGCAGGCCCCCGAGGTGTCGGGAGCCATCTCCGCGCTGTTCCGCGACAACGGCGACCGCGACAACCG
CTACAACGCCCGCATCAAGTTCCTGATGGACGAGTGGGGCGCGGAGAAGTTCCGCAACGTCCTCCAGGAGGAG
TACGTCGACTTCGAGCTGGAGACCGCCGGCGAGGACCTGCGCTCGGAGTACTCCTACAACTCCGGCTACGCCG
ACGGCGGCCACTCCGACCACGTCGGCATCCACGAGCAGGCCGACGGCAACTACTACGTCGGACTCGACGTGCT
CGTCGGCCGCATGGGCGTCGACGACACGAAGGAACTCGCCCGCGTCGCCGACGAGTACGGCTCCGGCGAGATT
CGCATCACGCAGCGCCAGAACGTCATCATCACCGACGTACCCGAAGAAAAGCTCGACGCGCTGCAAAACGAGG
ACCTTCTCGACCACTACTCGCCCGACCCGCACCCGTTCATGCGCGGCTCCATCGCCTGTACGGGCACCGAGTT
CTGTTCGCTCTCCATCGTGGAGACGAAGAACCGGCAGGTCCGCTACGCCCGCTGGCTCAAGGAGAACGTCGAC
CTCCCCGCGGGCGTCGAGGACTTCCACATCCACCTCTCGGGCTGTACGGCCTCCTGCGCCCAGCCGCAGATTG
CGGACATCAGCCTCCGCGGCATGAAGACCCGCAAGGACGGCGAACCGGTCGAGGCGCTCGACATCGGCCTCGG
CGGCGGCCTCGGCGAGAACCCGAACTTCGCCGAGTGGGTCACCCAGCGCGTCCCCGTCGACGAAGTGCCGGGT
GCCATCAAGAACCTGCTCGCGAACTTCGAGGAGCGCCGCGAGGGCGACGAGACGTTCCGCGAGTTCGTCGCCC
GGACGGACGAAGAGGAGCTGGCCGAACTAGTCGAACCCGAAGAGACCAGCTACGAGGACCCGATGATGCACAA

CACGAAGCGCACGTGGTACCCCTACGCCGACGACGACGGCCTCGACGCGAGTCCCGCGCCGACCCGCGCCGAC
GGCACGCCCATCGCGAGCGACGACTGA
>hhi:HAH_0254-Ar nirA1; ferredoxin-nitrite reductase (EC:1.7.7.1); K00366 ferredoxin-nitrite reductase [EC:1.7.7.1] (N)
ATGCCCAGTAAAGTCGAGAAATGGAAGGACGAGGTCTACGGTAACGAGATTCGAGAGCATCTGATGGAGTTCG
CAGAGAAGGGCTGGGACGCCATTCCGGAAGACGAGCACGACGCCTGGTTCGAGCGCTTCAAATGGTGGGGGCT
GTACCACCAGCGTTCCGGCCAGGAGTCGTATTTCATGATGCGCATCGGGACGCCAAACGGCGTCATCAAGCCC
GGCCAGCTCGAAGTCATCGGCGAAGTCGCGAAGGAGTACGCGACCGGCCCCGTCGATAACCCCGAGTTCGGCG
AGGCCTACTGTGACTGGACGACCCGCCAGAGCATCCAGCTCCACTGGATCAAACTGGAGGACATCCCGGAGAT
CTTCGAGAAGCTCGAAGCCGTCGGTTTGGGCACCCAGCAGGCCTGCGGTGACTCCTGGCGGAACATCGTCGGC
TGTCCCGTCGCCGGCAAGGACAAACACGAGCACGTCGACGCCTGGCCCGTCGCCGAGGACCTCCACGAGACGT
TCAAGGGCAACGACGACTACTCGAACCTCCCGCGCAAGTGGAAGGTCGCCATCGCCGGCTGTGACGAAGGCTG
TGGCCAGGGCGACATCAACGACCTCGCCTTCGAGCCCGCCGAGAAGGACGGCGAACTCGGCTTCAACGTCCGT
ATCGGCGGGGGCCTCTCCCGCAAGGAACCGCGCCTCGCCCGCGATATCGACGTGTGGGTGCCGCCGGAGCAGG
CCGCCGACGTGGCCCACGGCATGTCCGCGCTCTTCCGCGACCACGGCGACCGCGAGGACCGCTTCAACGCCCG
CATCAAGTTCCTCATGGACGAGTGGGGGCCGGAAAAGATGCGCTCGGTCCTCCAGGACGACTACGTCGACTTC
GAACTGCCGACCGCCGGCGAGGACATGCGCGACCAGTACAGCTACAACACCGGGTCCCAGGACGGGCACAACG
ACCACGTCGGGATTCACGAACAGAAGGACGGCAACTACTACGTCGGCCTGAACGTCCTGGTCGGCCGCATGGG
CGCCGACGACGTGCTCGAACTCGCGGAACTCGCCGACGAGTACGGTTCCGGCGAGGTCCGCTTGACCCAGCGC
CAGAACATCATCGTCACTGACGTCCCCGAGGAGAACCTCGACGAGTTCACCAGCGAACCCCTCCTTGAGAACT
ACTCCCCCGAGCCGTCGCCGTTCATGCGCGGCTCCATCGCCTGCACGGGCACGGAGTACTGCTCGCTCTCTAT
CGTCGAGACGAAGAACCGCCAGGTCCGCTACGCCCGCTGGCTCAAGGACAACGTCGAACTGCCCGAGGACCAC
AAGGACTTCCACATCCACCTCTCTGGCTGTACGGCCTCCTGTGCCCAGCCCCAGATCGCCGACGTGTCCCTGC
GCGGCATGAAGACCCGCAAGGACGGCGAACCGGTCGAGGCGCTCGACATCGGTCTCGGCGGCGGCCTCGGCGA
CGACCCGCGCTTCGCCGACTGGGTGGAGATGCGCGTCCCGGCCGACGAAGTCCCCGGCGCGATCAAGAACCTC
GTCAACAACTTCAAGAACGCCCGCGAGGGCGGCGAGACCTTCCGTGACTTCGTGGAGGACCGCGACGAGGAAA
CGCTGGCCGAGATGGTCGAGCCCGAAGAGACGGACTACCACGACCCGTACATGCACAACACGAAGATGACGTG
GTACCCCTACGCAGAGGACGACGACATGCAGTCCTCGCCTGCGCCGACTGACGGCTCTGGTGAGCCCCTGCCC
TCCGACGACTAA
>hma:rrnAC2625-Ar nirA1; ferredoxin-nitrite reductase; K00366 ferredoxin-nitrite reductase [EC:1.7.7.1] (N)
ATGGCACATAAAAAAGAGGAGTACAAGGCGGAGCTGTACGGTGATGAGGTACGGGAGAAACTAGAGGAGTTCG
CCGAGAAAGGCTGGGACTCGATTCCCGAGGACGAACGCGAGAAGTGGTTCTCGCGGTTCAAGTTCTGGGGTGT
CTTCCACCACCGCGGCGGACAAGAGTCGTATTTCATGATGCGGCTGACAAATTGCGGCGGCGTTCTGGAGCCC
GACCAGCTCCGGGCTATCGGCGAAGTCGCCCGCGACTACGCGAAGGGGCCAGCGGAGAACCCTGAGTTCGGAA
ACGGCTGGATTGATTTCACGACACGACAGTCCATCCAACTGCACTGGCTCAAACTCGAAGACATCCCGGAAAT
CTGGGAGAAACTCGAAGCCGTCGGCGTTTCCTCGCGCTCTGCGGGCGGGGACACGATGCGGAACATCTCCGGT
TGTCCGGTCGCCGGCAAGGCCGAGGAGTACGTCGCGTCTCGCCCGATTCTCGACGAGATTCAGGAGACCATCC
GCGACGACAACGACCTCTCCAATATGCCCCGGAAGTTCAACATCTCGGTGACGGGGTGCAAGCAGGGGTGTGC
CCAGGACAGCATCAACGACATCGGGCTGGAGCCGGCCCACAAGTTCATTGACGGCGAGGAAATCGAGGGGTTC
AACGTCCGCGTCGGCGGCGGCCTCGGTGGTCGCGAACCCCGCGAGGCCCGTCCGCTGGACCTGTTCATCCGGC
CCGAACACGCCGTCGAGACGGTCCGTGCTTTTGTCGAGTACTACCACGAGGCGGCAACCGTCAGAACCGCTC
GAAGAACCGCGCCCGGTTTTTCGTCGACGAACATGGGACCGACGCCATCCGCGAGGAACTTGACGAGCGGCTG
GATTTCGAGTTTGAAACCGCTGGCACTGACTTCCGCGGGGAGTATACGTACAACGCCGGCAAGTCTGCTGAAC
ACGGTGCCCACGACCACGTCGGCGTCTACGACCAGCAAGACGGGAAGAACTACGTCGGGCTCTCGGTGCCCGT
GGGCCGACTCTCCGCAGAGGACACCATCGAACTCGCGGACCTCGCCGACGCCTACGGCTCCGGCGAAGTGCGC
CTGACCCGCCGACAGAACCCAGTCATCATGGACGTCCCCGACGGGAACATTTCGAATCTCCTCAACGAGCCGC
TACTGGACAAACACTCGCCCGAGCCGAACCCGTTTGTTCAGGGGGCGATGGCCTGTACCGGAACGGAGTTCTG
TTCACTCGCGCTCACCGAGACAAAGGCGCGGATGGCTCGCCTGCTCCGCTGGCTCGGTGACAACGTCGATGTG
CCCGATGATGTTGACCGCATCAAAATGCACTTCTCGGGCTGTACGGCTGACTGCGGTCAAGCGATGACAGCCG
ACATCGGCTTACAGGGGATGCGCGCCCGCAAGGACGGCCAGATGGTCGAAGCCGTCGATATCGGTGTCGGCGG
CGGTATCGGCGCGGAGCCGACGTTCATCGAGTGGGTCCGCCAGCGCGTCCCTGCTGACGAGGTGCCGGGCATG
ATTGCCAACATCGTTGAGGCATACGCCGCGCTTCGCTCGGAGGGGCAGACCTTCCGGGAGTGGGTCGACGCCA
CCGGCCACGAAACAATCGTCGAGCTGGCCGAACCGGAGGAAGTCGAGGGGTACACCGACCCATGTCTGGCCGA

CGGCAAGCAATCGTGGTATCCCTTCGACGACGGCGAAAGCCCGGCCCCGACCGACGCCGAGGGCCAGCCGATT
TCGGCGGACGACTGA
>csy:CENSYa_0360-Ar ferredoxin-sulfite/nitrite reductase (EC:1.7.7.1); K00366 ferredoxin-nitrite reductase [EC:1.7.7.1] (N)
ATGACAAACGACTATTCAATGATAAGGGTAAAGGTGCCCGCAGGCGAGATACTCCCCCAGCAGCTCGAAAAGA
TAGCGTACCTGTCCGAGACCTATTCGATAGGCAGCGCGCATTTTTCTACACGTGAGAATGTGCAGCTCCACTG
GGTTGTTCTCGAGGACATATCCGAGATAATGAGGGGCCTCGCCGAGGTGGGCATGACCAGCAGGGAGGCCTGC
GGGAACACGGTCCGGAATGTGATGTGCGCCCCGCTTGCCGGCGTCTGCAGGGACGAGGCGTTCGACGCGACGC
CGTACGCGCAGGCGACCGGCACGTTTTTCCTGCGCAATCCAATGTGCCAGAACCTCCCCCGCAAGTTCAAGTT
CAACTTTACATGCTGCGAAGATCACGGCATGGTCAGGATGGTAGACGTGGGCCTTGTCCCCCAGACGCGCGAG
ATCAACGGGGTCATGCAGCGCGGGTTTATGATATTCCTCGGAGGTGGACAGGGAAACAAGGCATTTTTGGGCC
ACCAGCTGGAGGGGTTCACGCCCGACGAGGATTTGTTGTACACGGCCCTGGCGGTGCTGAGCGTATTCGACAG
GCTCGGCGACAGAAAAAAGATGGCGCGCAACCGCCTGAGATATTTGGTAAGCGAGATGGGGTGGGAAAAGTTC
CGGAATTTAGTCCTCAAGGAGAGGGCGCTGGCCCGGTCCAACCAGTCCGTGCTGACCCAGCTGGATACCGATG
GAACACCCCGCGGGATAAGGACCCCCATGAGGATAGCCGACGAGGCGCCCGCCGCCGTCCCCTCCGGCTTTGC
CCGCTGGTCGCACACCAATACGATACCGCAAAAGCAGGACGGCTACCGCTCGGTATTCATAACGCTCGAAGCT
GGCGACATAACGGCGGCCCAGCTCAGGTCGGTTGCGGCACTTTGCAGGGACTATTCCGGCGAGTGCAAGGCGA
GGACCGGCTTTCATCAGAACGTGGTCATCCGGTATGTAAAGGAGGACGACCTGCCCGCGATGTACTCGAGGCT
TCTCGAGGCGGGGCTCGCAAAGACGGGCGCCCTTACCATGGCCACCACTGTCGGCTGCTCGGGGACCACCTCG
TGCAATCTTGCGCTAACCAACTCGCACAGGCTTGCAAAAGAAGTGCAGAGAAAGCTCCTCGAGCTGGGGATAG
ACGAGGATCCAGATTTCTCAAACTCTAGCATCAAGATAAGCGGATGCCCCAACTCGTGCGGCCTGCACTGGAT
GGCCACGCTGGGCTTTTACGGGGGCGGCTCAAGGGCTGGCAGCAGCATGTACCCGGTATACCAGATGCTAGTC
GGCGGCAGGTTCGACGGACACTCCTCCCTGGGAATACCCATACTTAGGATTCCTGCAAAGCGGGTCATACCGG
CGATACTCAAGGTGATAGAGCTGTTCCGGGAGCACAAAAAGCCGTCGGATACGCTAAAGTCATGGCTGCACAG
GCTGGTCAGCGGCAACGGCGATGGAACCATAGATACCCTGGACGCGCTCAAAGAAGAGCTGTCCCCGCTTACG
GTGGCGCCCTCGATAGAGGTAGACCCGGACTTTTACAAGGACTATGGGAACGAGGATAACTATCACACAAAGA
CCGGAAGAGGAGAGTGCGCCTCTTGA
>hmu:Hmuk_0435-Ar Ferredoxin--nitrite reductase (EC:1.7.7.1); K00366 ferredoxin-nitrite reductase [EC:1.7.7.1] (N)
ATGCCGACGAAAGTCGAGAACTGGAAGGACGAGGTGTACGGCAACGAGATCCGTGACCACCTCATGGAGTTTG
CCGAAGAGGGCTGGGACGCGATTCCCGAGGACGAACACGACGCCTGGTTCGAGCGCTTCAAGTGGTGGGGCCT
GTACCACCAGCGCTCCGGGCAAGAGTCGTACTTCATGATGCGGATCGGGACACCCAACGGTGTCATCGAGCCC
GGACAGCTGGAAGTGATCGCGGAGGTCGCCGACAAGTACGCCCGCGGCCCCGAGGAGAACCCGGAGTTCGGCG
ACGCCTACTGTGACTGGACGACCCGCCAGTCGATCCAGCTCCACTGGATCAAGATCGAGGACATCCCCGAAAT
CTTCGAACTGCTCGAATCCGTCGGTCTCTCGACCCAGCAGGCCTGTGGTGACTCCTGGCGAAACATCGTCGGC
TGTCCCGTCGCCGGCAAGGACAAACACGAACACATCGACGCCTGGCCGATCGCCGAAGACCTCCACGAGACGT
TCAAGGGCGACGACGACTACGCGAACCTCCCGCGCAAGTGGAAGGTCTCGATCACGGGCTGCGATCAGGGCTG
TGGCCAGGGCGACATCAACGACCTCGCCTACGAGCCAGCCGAGAAGGACGGCGAACTCGGCTTCAACGTCCGC
GTCGGCGGCGGCCTCTCCCGAAAGGAGCCCCGCCTCGCGCGTGACATCGACGTCTGGGTACCCCCGGAGAAGG
CGGCCGACATCGGTCACGGGATGTCCGCACTGTTCCGCGAGTACGGCGACCGCGACGACCGGTTCAACGCCCG
CATCAAGTTCCTCATGGACGAGTGGGGTCCCGAAAAGATGCGGTCGGTCCTCCAGGAGGAGTTCGTCGACTTC
GAGCTTCCGCGGGCCGGCGAGGACATGCGCGACCAGTACACCTACAACGCCGGGGGCAACGAGCACGGCGACC
ACGTCGGCGTCCACGAACAGCCCGACGGCAACTACTACGTCGGCCTGGACGTCCTCGTCGGTCGGATGGGCGT
CGACGACGTGTACGAACTGGCCGAAATCGCCGAGGAGTACGGCTCCGGCGAGGTCCGGCTGACCCAGCGCCAG
AACGTCATCGTCACGGACGTCGACAGTGACCGCCTCGACGCGTTCCTCGACGAGCCGCTGCTGGACGACTACT
CGCCCGATCCCCACCCGTTCATGCGGGGCTCGATCGCCTGTACGGGGACGGAGTTCTGTTCGCTGTCGATCGT
CGAGACGAAGAACCGACAGGTCCGCTACGCCCGCTGGCTCAAGGACAACGTCTCGCTGCCCGAGGGCGTCGAG
GACTTCCACATCCACCTCTCGGGCTGTACGGCCTCCTGTGCCCAGCCCCAGATCGCCGACATCTCGCTGCGGG
GCATGAAGACCCGCAAGGACGGCGAGCCGGTCGAGGCATTCGACATCGGCCTGGGCGGCGGACTCGGGGAGAA
CCCCCAGTTCGCCGACTGGGTCGAGATGCGCGTCGCCGCCGACGAAGTGCCGGGCTACATCGAGAACCTCCTC
GACGCCTTCGAGGCCGAACGCGAGGGCGACGAGACCTTCCGCGAGTTCGTCGCGGACCGCGACGAGGAGGCCC
TACAGTCCCTGGCAGATCCCGAAGAGACGAGCTACGACGATCCGTACCTGGGCAACACGAAGATGACCTGGTA
CCCCTACGCCGAGGACAACGACATGCAGTCCTCGCCGGCACCGACCGACGCCAACGACGAGCCGCTGCCGTCC
GACGACTGA

>hxa:Halxa_1302-Ar Ferredoxin--nitrite reductase (EC:1.7.7.1); K00366 ferredoxin-nitrite reductase [EC:1.7.7.1] (N)
ATGAACACCGTCGAAGAACACAAGCAAGAAAAACATCCGCTGGACGTCATCGAGGACGTCTACGACTACGCGG
AGGGGGACCTCTCCGACGAGGAGATCGAGGAACGCGCCGGCGGCGGCGAGTGGGAGCGGCTCAAGTGGGCCGG
GATGTACTCCCAGAAGCAGGACGACTACTTCATGATCCGGACCAAGGTCCCGGGCGGCTACCTCACGCCCGAG
CAGGCCGAGGTCATCGGGGAATGCGCCACCGACTACGCCACCGCGCCCGAGGAGTACGGCGGCGAGGAACAGA
ACGAGCTCTGGGGCGACGCCTTCCTCGACATCACGACCCGGCAGGACATCCAAAAGCACTGGGTCGAGGTCTC
GGACGTCCCCGAAATGTGGGAACGCTACGACGAGGTCGGCCTGACGACCGTCCAGGGTTGTGGCGACTCCGCT
CGTAACGTGCTGGGCTGTCCCGCCGCCGGGCTCGACGACCACGAGTGTTTCAACGCCCAGCCGGTCATCGACG
CCGTTTCGGACTACTTCACCGAAAACCGCGAGTACGCCAACCTGCCGCGGAAGTTCAAGATCACGATCACGGG
CTGTGCGCACGACTGCGCTCAGTCCCAGATCAATGACGTCGGGATGACCCCGGCGAAAAAAGAGATCGACGGC
GAGTACTACTACGGCTTCCACGTCCGCGTCGGCGGCGGCCTCTCCGACGGCCCGCGGATGGCCTCCGATCTCG
ATGTCTTCGTCCCACCGGAGGACGCCGTCGAGTTCTGCCGCGCCGTCGCCCAGACGTTCAAGGAACTGGGCGA
CCGCAACAACCGCGGCGTCTGCCGAATGCGCTACCTCGTCGAGCAGATGGGCGCGGACAAGTTCGAGGAAGCG
ATCCGCGACCGCTGTACCGTCGACCTGCCCACCAGCGGCGAGGACCTGACCGTCGGCTACCGCGGCGACCACG
TCGGCGTCCACGACCAGAAACAGGACGGCCTGAAGTACGTCGGCTTCAACGTCATCGCCGGCCGCATGGGCGG
CGACGAGTTCGCCGAGGCCGCCCGCGCCGCCAAGAAGTACGGCACCGAGGACACCTCGATTCGCCTGGCCACC
GACCAGAACTTCCTCATCACCCACATCCCCGAGGAGAACGTCGACGACCTCCTCGCGGAGCCGTTCGCTCAGG
AGTACAGTCCCGATCCGGGGCCGTTCTCGCGGGGCGCAGTCGGCTGTACGGGTACCGAGTTCTGTAACTACGC
CATCATCGAGACCAAGAAGCGCACCAAGCGCTGGGCCCGCGAACTCGACGAGCGCATCGACACGCCCGACGAC
CTCGAGGTCGTCCGGATGCACATGTCGGGCTGCTCGGCCTCCTGCGCCCAGCCCCAGATCGCCGATATCGGCT
TCCGCGGCGAGACCGTCAAGCTCGAGGACGAGAACAGTCCCAATGAGGAAGGTGACAACCTCGTCGAGGGGAT
GGACTTCGGGATCGGCGGTGCGCTCGGCGCCGACAACGAGTTCTTAGACTGGATCGAGCGCGCGGTGCCCGCC
GAGGGCGTGATCCCGGCGCTCGAGCAGCTGTTCGACGCCTACAACGAAGACCGCAACGACGGCGAGCGCTTCT
ACGAGTGGGCCCGTCGCGTCGACAACGAGCGACTTCGGACGCTCATGACCGACGCCGACGCGCCGGTCGCACG
AGGTGTTGCCCATGGAGACTGA
>hxa:Halxa_1554-Ar Ferredoxin--nitrite reductase (EC:1.7.7.1); K00366 ferredoxin-nitrite reductase [EC:1.7.7.1] (N)
ATGGTGCATAAGAAAGAGGAACAGAAGGAGGACTGCTACGGCGACGAGGTCCGGGAACACATCGAGGAGTTCG
CCGAAAACGGCGGCTTCGAGGCTATCCCGGACGACGAGGTCGACAAGTGGACCACCCGGTTCAAGTTCTGGGG
GGTCTTCCAGCAGCGCTCCGGCCAAGAGGAGTACTTCATGATGCGGCTGACCAACGCCAGCGGCATCTTAGAG
CCGGGCCAGCTACGCCGCATCGGCGAAGTCGCCCGCGACTACGCGAAGGGCCCCGTCGAAAACCCCGAGTTCG
GCAACGGCTGGATCGACCTCACGACGCGCCAGTCGATCCAACTGCACTGGCTCAAACTCGAGGACATCCCCGA
AATCTGGGAGAAACTCGAGGCTGTCGGCGTCCACTCCCGTTCGGCCGGCGGCGACACGATGCGCAACATCTCG
GGCTGTCCGCTCCACGGGAAGGCCGAGGAGTTCGTCGAGTCCGGCCCGCTGCTCGAGCGCTTCGAGGAGGAAC
TGCGCGGGGACGACGCCCTCGCGAACATGCCCCGGAAGTTCAACATCAGCGTCTCGGGCTGCCGGGTCGGCTG
CGCGCAGGACTCGATCAACGACATCGGGTTCGAGCCGGCGACCAAGGAGATCGACGGCGAGGAGGTCCGCGGC
TTCAACGTCCGCATCGGCGGCGGCCTCGGCGGCCGCCAGCCCCGCGCCGCGACGCCGCTTGACGTCTTCGTCC
GCCCGGAGAACGCCTACGAGGTCGGCCGCGGCTTCGTCGAACTCTACCACGATTACGGCAACCGCCAGAACCG
CTCGAAGAACCGCGCGCGGTTCTTCGCCGACGACTGGGGAATGGAAAAGATCCGCGAGACGCTCGAGGAGGAG
TACGTCGACTTCGAACTGCACACTGCGGGCGAGGACTTCCGCGGCGAGTACACCTACAACGCCGGTCGACCCC
GCGACGAGGGGCCCCACGACCACGTCGGCGTCGGCGACCAGAAGGGAGACGGCATGAAGTACGTCGGCCTGAG
CGTCCCCATCGGCCGCCTGCCGGCCGAGGACGCCATCGAACTGGCTGATCTGGCCGACGAGTACGGTTCCGGC
GAGATCCGGCTGACCCGTCGCCAGAACCCCGTTATCGTCGACGTCCCTCACGAAGAGGTCGACGACCTGCTCG
CGGAGCCGATCCTCGAGGAGTACCCGCCCGAACCCAGCGCGTTCGAGCGCGGCGCGATGGCCTGTACCGGCAC
GGAGTTCTGTTCGATCGCGCTGACCGAGACGAAAGCGCGGATGGCCCGCATGCTGCGCTGGCTCAACAAGAAC
GTCGACCTCCCCGACGACGTCGGGAAGATCAAGATGCACTACTCCGGCTGTACGGCCGACTGCGGCCAGGCGA
TGACCGCCGACATCGGCCTGCAGGGGATGCGCGCTCGCAAGGACGGCGAGATGGTCGAGGCCTTCGACATCGG
CGTCGGCGGCGGCGTCGGCGAGGAGCCCTCCTTCGTCGACTGGATCCAGCAGCGCATCCCGGCCGACGAGGCG
CCCGGCGCGATCCGCAACCTGCTCGAGGCCTACGCGGCCCACCGCAAGGAGGGTCAGACGTTCCGTCAGTGGG
TCGAGGCGACGGGCCAGGAGCAGCTCATCGAGTTCTGCGAACCCGAGGAGACGGACTTCGAGGCGCCGTACAT
GGACGACGCCAAGCAGTCGTGGTACCCCTTCGCCGACAGTCCGTCGGCGGCCGCGGCCCTCGAGGAATCGGCC
GCGCCGTCTGACGACTGA

>nmg:Nmag_1601-Ar Ferredoxin--nitrite reductase (EC:1.7.7.1); K00366 ferredoxin-nitrite reductase [EC:1.7.7.1] (N)
ATGCCTACTGACGTCGAGGAGTGGAAGGACGAGGTCTACGGCACAGACATTCGAGACCATATCGAGCGATTCG
CCGAAGCGGGCTGGGAGTCGATTCCGGACGACGAGAAGGACGCCTGGTTCGAGCGCTTCAAGTGGTACGGCCT
GTACCACCAGCGCGCCGGCCAGGAGTCGTACTTCATGATGCGGATCGGGCCACCGAGCGGCGTACTCGAGCCG
GGGCAGTTCCGTCGCATCGTCGAGATTGCAGACGAGTACGCCCGCGGTCCGGTTTCGAATCCCGAGTTCGGCA
ACGGCTGGCTCGACGTAACGACGCGTCAGGCGATCCAGCTCCACTGGATTCGAATCGAGGACGTGCCCGGTAT
TTTCGAGGCACTCGAGGACGTCGGGCTCTCGACGGTCCAGGCCTGCGGTGACTCCTGGCGGAATATCGTCGGC
TGCCCGGTCGCTGGGAAGGACAAACACGAGCACGTCGATGCTGCCGCGCTCGCGGACGACCTCCACGATACGT
TCAAAACGAACGATGCGCACTCGAACCTGCCCCGCAAGTGGAAGGTCTCTGTGACGGGCTGTGACGAGGGCTG
TGGGCAGGGTGACATCAACGACCTGGCGTTCGAGCCAGCCGAGAAGGAGATTGATGGCGAGACCGTCACGGGC
TACAACGTCCGCGTCGGCGGCGGACTGGCGCGCAACGAGCCTCGGTTTGCGAAGAACATCGATGTCTTCGTGA
CGCCCGAGCAGGCCGTTGACGTCGCTGGCGGTATCTCGGCGCTCTTCCGCGATCACGGAGACCGCGAGAACCG
CTACAACGCGCGCATGAAGTTCCTCGTCGACGAGTGGGGGCCAGAGAAGGTCCGCAACGTCCTCCAGGGAGAG
TACATCGACTTCGAACTCGAGACGGCCGGCGAGGATCTGCGTCACTCCTACAGCTACAACGCTGGTGACGCGG
ACGGCAAACACGACCACGTTGGCGTCCACGAGCAGGACGACGGCACCAACTACATCGGACTGAACGTCCTCGT
GGGACGTCTCGGAATCGACGACGGCTACGAGATCGCCGACGCGGCCGAAGAACACGCCTCCGGTGAGGTTCGC
CTGACCCAGCGCCAGAACATCATCCTGACGGACGTGCCGGACGAGGAGGTCGACGCGCTGCTCGACAATCCGG
TCTTCGACGAGTATAGCCCGGATCCACACCCGTTCCAGCGAGGCTCGATCGCCTGTACCGGCAGCGAGTTCTG
TTCGCTTTCGATTGTCGAGACGAAGAACCGCCAGGTGCGCTTCTCGCGCTGGCTGGTCGACAACGTCTCGCTT
CCTGAGGGCGTCGAGAATTTCCACATCCATCTCTCTGGGTGTACGGCATCCTGTGCCCAGCCCCAGATTGCCG
ATGTCTCCCTGCGGGGTATGAAGACCCGCAAGGACGGCGAACCGGTCGAGGCGCTCGACATCGGCCTCGGCGG
CGGCCTCGGCGAGAATCCGCAGTTTGCCTCCTGGGTGTCGGACCGCGTGCCTGCTGACGAAGTGCCGGGTGCG
ATTGAGAATCTGCTCGCCAATTTCGACGACGCGCGTGAAGACGGTGAGAGCTTCCGCGAGTTCGTTGCCGCTC
GCGACGACGAGGTACTGGCTGATCTCGTCGAGCCCGAGGAGACGAGCTACGAGGATCCGTTCCTGCACAACAC
CAAGCGAACCTGGTATCCCTACGCGGAGGACGACGGATTCGACGCCAGTCCTGCACCTGCCCGTGCGGACGGG
ACGCCGATTCCATCGGACGACTGA
>hje:HacjB3_01275-Ar ferredoxin--nitrite reductase; K00366 ferredoxin-nitrite reductase [EC:1.7.7.1] (N)
GTGGCGCCTGAGATCGAACAGTGGAAAGACGACTGTTACGGCGAGGAGCTGCGCGGGAAGATCCTCGAGTTCG
CGGCGGGCGGCTGGGAGACGATCCCCGAGGGGGAACGCAAGAAGTGGTTCTCGCTGTTCAAACACTGGGGGGT
CTTCCACCAGCGCGACGGCCAGGAGAGCTACTTCATGCTGCGCCTGCAGAACGCCGGCGGCGTGTTGCGCCCC
GGCCAGTTGCGCGCGATCGGCGAGGTCGCCCGCGACTACGCGACCGGCCCCGCCGAGAACCCCGAGTTCGGCA
ACGGCTTTCTCGACCTGACGACCCGCCAGTCGGTCCAGCTGCACTGGATCAAGCTCGAGGACGTCCCCGCGAT
CTGGGAGAAACTCGAATCGGTTGGCGTCTCCTCCCGATCGTCGGGGGGCGACTCGATGCGCAACATGGCCGGC
TGCCCGGTCGCCGGGCTCGACGCCGAGGAATTCATCGACTCGCGGGACCTGCTCGAACGGCTGAATACCGACC
TGCGCGAGGACGATACTCTCGCAAACCTCCCTCGGAAGTTCAACGTCTCGGTGACGGGCTGTCGGGAGGGCTG
TGCCCAGGACTCGATCAACGACATCGGGCTCGAACCCGCCGAGAAAGGCAGAACGAAAGGCTACAACGTCCGG
GTCGGCGGCGGTCTCGGAGGGAGACAGCCCCGGGAAGCCCGCTCGCTCGACGTGTTCGTCACGCCCGAGGAGG
CCTACGACGTGGTGCGAGGTTTCGTCGAACTGTATCAGGAACGCGGCCAGCGCGAGAACCGCCAGAAGTCCCG
CTCGCGCTTTTTCGTCGACGACTGGGGCGTCGAGGAGATCCGTGCGACCCTCCAAGCGGAGTACGTCGAGTTC
GCGCTCCGGACCGCGGGCGCGGACCTCCGCGAGGAGTACACCTATAACGCCGGGACCTGTCGCGGCGGCGACC
ACGTCGGCGTCCACGAGCAGAAAAACGGCGAGTACTACGTGGGTCTGAGCGTCCCGGTCGGTCGCCTGCGCGC
GAAGGAGGCCATCGCGATCGCGAACTGCGCGAGCGAGTACGGAAGCGGGGAGGTACGCCTGAGCCGCCGGCAG
AATCCCCTGATCATGGACGTTCCCGAGGCCGATCTCGACCCGTTGCTCTCCGAACCGGTACTGGAGACCCACA
CGCCCGAGCCCCACCCGTTCCAGCGCGGGTCGATCGCCTGTACGGGCACCGAGTTCTGCCCGCTGGCGCTGAC
CGAGACGAAAGCCCGTTTGGCGCGAACGATCCGCTGGCTGCGCGAGAACTGCGACCTCCCTGACGACGTCGAG
GACATTCATATTCACTACTCGGGCTGTACGGCCGACTGTGGGCAGGCGCTCACCGCCGACATCGGTCTGCAGG
GCATGCGCGCACGCAAGAACGGCGAGATGGTCGAGGCGCTGGATATCGGCGTCGGCGGCGGAATCGGGGAAAA
CCCCTCGTTCATCGAGTGGATCCACCAGCGCGTCCCCGCCGACGAGGCACCGATGGTGATCAAGAAACTCGTC
GAGGCCTACGGCGCCCACCGCTCTCCGGGCCAGTCCTTTCGCGAGTGGGTCGAAAACACAGGCGTCGAGAGCC
TCGTCGAACTCGCCGAACCCGAGGAGAGTTCCTACGAGGACCCGTGGATGGACGACGCGAAGGGGTCGTGGTA
CCCCTTTGCGGAAGGCGAGAGCCCCGCCCCGACGGCCGCCGACGGCACCCCGATCTCCGCTGACGACTGA

>pai:PAE2577-Ar ferredoxin-nitrite reductase; K00366 ferredoxin-nitrite reductase [EC:1.7.7.1] (N)
ATGAGCTGGGAGGAGGTCTACAAGAAGAATTTAATAGAGAAAATTAAACGGGACAAACATCCCTTTAAATTAC
TTGAGGAGCTGGAAACCTTGGCTAGGCGCCACTACAGCGATATACCCGAAGAGGAGTTTATCAGGTTATACTG
GCTAGGGGTAGTACATGATAGGCCTAGGACCGGGTATCTCATGTTACGCCTTAGGATACCTGGTGGTATTTTA
AAACCGCATCAATTGCGGAGAATTGGGGAATTGTCTTATAAATATGGCAAGAATTACGCCGAGATAACTGTTA
GACAAGACATCCAGTTACACTGGATAGAGCTTAAGTACCTTCCAGAAGTACTTGCTCAGTTAAAAGATGTGGG
GATTTTAACTATAGGAACAGAGGGGGATACGGTAAGGAATATAACCACTTGCCCTCTGGCAGGCCTTACCGTT
AATGAGTTGTTTGATGTTAGAGATGATGTATATAAAATAGATAAGGCCTTTCTCTTGGATCCCATATATGCTG
ATTTACCCAGGAAGTTTAAAATCACAATCACCGCGTGTCCTTATATGTGCAGCGTACCCGAAATTCAAGATTT
AGCTTTGATAGGAGTCACCAAAGACGGCAAATACGGTTATACAGTACTGATTGGCGGCGGTCTCTCTCTGGCG
CCTCGTGTAGGAAGGCATATGCCAGTATTTGTGCCTAGAGAAATGGTTATTGAGTTTGTAAAAGCTGTATTAG
ATGTCTGGAGAGAAGAGCCTAGATATCGTCTCTCCCGTGTTAGAGCTAGAATTAAGTTTATGATCGACGATCT
TGGGGTAGATAAGTTCAGAGAGCTTGTAGAAAATCGATTAGGTCAAAGGCTGGAAGATTATCCTTCTAAGCCT
GAGTCTCTGGGCAAGACCGATCATATAGGGGTAGAACGGCTTAAAGACGGCACATATGCTGTCGGAGTTGCCA
TACCAGCAGGGATAGTGACAGGGGAGTTCTTGATAAAGCTTGCAGATTTAGCAGAAGCTGTGGGAGTCAAGGA
AGTGAGGCTGACGCAACGTCAGAATTTAATTCTAGCAGGCGTCCCGGAGGACAAGGTTGAGATCGTGAAAGAG
TTTTGCCAGAAGTGGGGTTATGCTGTAGAACAGACGCCTCTTGTGGCCATGTCTGTGGCTTGCACTGGAGATC
CCTATTGTAATTACTCTATTTCCGACACGAAGGGACTTCTACTTGAATTAATAAAAAGCTTGAAAGAGAGATT
CGGCGAAATACAGGATCTTCCTCCTATACATATTGTGGGCTGTCCACACGCTTGTGCATGGTATTGGATTGGG
AACATAGGCCTTATGGCCACCACGGCTAAAATGCCTGATGGAAGGTTTGTTAAAGCGTATGATGTCTTAGTAG
GAGGGGATTATGGTAAAAGGACCGCCATAGGCAAAGTATTGTTTAAAAAAGTGCCTGCAGATAAGATTAAGGA
CTGCATAACTTACCTTGTTGAATATTACATCAATAAGCGGGCACCAGGTGAGGACTTTCCTACCTTCATTGCT
AGAATAGACTTAAGAGAACTAGCTGATTACATATCGGGTAAACTAGGAGTACAATTAGCGCTGGGAAAATGA

>tba:TERMP_00352-Ar NADH oxidase/nitrite reductase; K00362 nitrite reductase (NAD(P)H) large subunit [EC:1.7.1.4] (N)
ATGAAACTTTTAATAGTCGGAAACGGCATTGCCGGAGTCACGCTGGCAAAAGAGCTGTCTAATGACTTCGATA
TTACAATAATCGACAGAGAAAACATACCATATTACAGCAAGCCCATGCTGAGTCATTACATAGCTGGTTTTGT
TGATGAAAAGTCGCTCTTTCCCTATTCTTTTGACTGGTATGAAAAGAAGAGCATAGAGCTGAAGCTGGGAGCT
GACGCCAAGGTGATAGACAGGGCAAGGAAAAGGGTGATGACCTCAGAGGGAGAACTTGACTATGACATTCTTG
TTCTTGCAACGGGAGCAAGGGCAAGGGAGCCCATGGTAGAAGGCAGGGAGCACTTAAATGTATTGAGAACACT
TGAAGATGCAAGGGCAATAAAAGAGATCCTTGAGCGAGAAGGAGATGCAGTAATTCTGGGGGGAGGATTTATA
GCACTTGAGCTTGCTGGAAATCTTGCGAAAGCAGGTTATGATGTAAAGCTCATTCACAGGAGGAATACCCTCC
TTGGTCTCGATGAAGAGCTGAGCAGAATATTGAAAAAAAGGCTTGAAGATGTGGGGGTTGAGTTTTATCTCAA
CACGAATTTGGTAAAGGCTGACGAGCGTGGAATCCTTACAGATAGAGGATACGTAGAAGGGAAGCTCAAAGTG
TGTGCTTTTGGTATAATACCAAATAGAGAAATTGCAGTGAGGAGTGGAATACACGCTGGAAGGGGTATTCTGA
TAGATGATAGATTCAGAACATCCGCAAAAGACATCTATGCAATAGGGGACTGTGCAGAGTACAACGGCATAAT
TTGCGGAACTGCCAGAGCTTCCATGGAACATGCAAAGGTTCTGGCAAACCTGCTTCGTGGAAGAGATGACAAG
TATGACTTTGAATTTCGTTCCTCAATTTTCAAGCTTGCCGGCTTTTCCGTGGCAATCATTGGTAAGATTGGTG
GTTATGGCAGATGGATAGATGAAAACATCAAACTCTTCATTGATCATCGAGAGAAAATCATTGGGGCAGTAGT
TCTTGACGACTTAAGATCCGCTACGAAGCTTGAGAAACTCATCAAGAGTGGGGCAGAGTATAATGACCTCCTG
TGA
>ton:TON_0865-Ar NAD(P)H:rubredoxin oxidoreductase; K00362 nitrite reductase (NAD(P)H) large subunit [EC:1.7.1.4] (N)
GTGAGGGTTGTAATAGTCGGCGATGGGCCTGGAGGAGTTGAACTTGCTAAGAGACTCTCTGGGGACTTTGAAG
TAACAATAGTGGACAAAGAAGGTCTCCCTTACTACTCAAAGCCGATGCTGAGCCACTACATAGCAGGATTTGT
GGATGAAAAGGCTCTTTTTCCGTATTCAGCCGAATGGTACGAGCGGAAGGGGATAGACCTCAGATTAGGCGTT
GAAGCCAAGATCATAGACAGAGCGAGGAATGTTCTCGTGACGAGCAAGGGGAATATTCCCTATGATGTCCTCG
TTCTTGCGACTGGCGCAAGGGCAAGAGAGCCTTCCATACCCGGGAGGGAGCACATCCTGACCCTAAGAACCTT
TGACGACGCCAAGATGATAAAGGAAAGGCTTGAAGGAGAGGGAGAGATCACGGTCCTTGGCGGCGGCTTCATA
GGGCTTGAGCTGGCCGGGAATGTGGCCAAGGCAGGCTATTCTGTCAAGCTTATCCACAGGAGCAAGAATCTCC
TCAGACTGGACGAGGAGCTGAGCGAAAAGCTCCAGGAAAAGCTGGAGGAGGTCGGAGTTGGGTTCCACCTCGA

GGCCAACCTCCTGAAGGCCGATAAGAACGGTGTGGAGACCGATAAGGGGTACATCCCCGGGAGGCTTAAGGTC
TGCGCCTTCGGGGTAGTTCCCAACAAGGAGCTAGCTTTGAGGAGCGGAATCCACGCTGGGCGTGGAATATTTA
TAGATGCAGGGTTAAGAACCTCTGCGAAGGACATCTACGCAATAGGTGACTGCGCCGAGTACAGCGGGACGAT
ATGCGGGACTGCCAAAACTGCTGTAGAGCACGCAAAGATCCTCGCAAACCTGCTCAGGGAGCAGGATAACCAC
TACGACTTCTCCTTCTGCTCGGCGCTCTTTAAGTTCGCCGACTTAAACGTTGCATTGATTGGAAGAACAAAGG
GAGAAGGCAGGTGGCTTGATGAAAAAACGAAAGTTTTCTACAAAGATGAAAAGCCCATTGGGGCAGTTGTGCT
CGGAGACGTAAGGAGAGCCATGGAGGTCGAGAAAGCAATCAGAGAAGGACTGCTTATTGACTGA
>tsi:TSIB_1897-Ar NAD(P)H:rubredoxin oxidoreductase; K00362 nitrite reductase (NAD(P)H) large subunit [EC:1.7.1.4] (N)
ATGAGAATTGTTATAGTTGGGAACGGACCCGGAGGAGTGGAATTAGCTAATCAGCTTTCTGGGGAACACAAGG
TAACGATAGTTGAACGTGAGAACGTTCTTCATTACAGCAAACCAATGTTGAGCCATTATATAGCGGGGTTTGT
TGAGAAGGAAAAGCTCTTCCCATACTCTTTCTCTTGGTACGAAAAGAAGGGGATAGACCTTAATCTGGGAGTA
AAAGCCGAAGTAATAGATAGGGCCAGAAAAAGGTTGATAACAAGCGATGGCGAGATCCCTTACGATGTATTGG
TTATAGCTACAGGAGCTAGGCCAAGGGGACCTACCTTTGAAGGCAAAGAGTTTGTGCAAACTCTCAGGACGCT
CCAAGACGCTGAAAGAATTAAGGAGCAAATAGAAAAATACAAAGATGCCTTAATATTGGGAGGAGGATTTATA
GGGCTTGAGCTTGTGGCAAATCTTGCGAAAGCGGGCTATAGAGTGAGGCTCGTTCATAGGGGAAGTAATCTTC
TTGGGCTTGACGAAGAGCTAACAAAGGCCATAATGGAAAAGCTCGAAGGATATGGTGTTGAATTTTATCTCGA
CGCAAACACTCTTAAAGCAGATGAGAACGGCATCTTTACAGATAAGGGGTACGTAGAAGGCAAAGTTAAGATA
TGTGCCTTTGGAGTTATCCCCAACAAGGAAATAGCCGTTAAAGGCGGCATTCACGCTGGAAGAGGCATACTAA
TCGATGACCATTTTAGAACTTCAGCTAAAGATGTATATGCCATTGGAGACTGTGCCGAGTACAATGGGATAAT
TGGAGGTACTGCAAAAGCTGCCATGGAGCATGCAAGGGTTCTTGCCAACATTTTGAGGGGTAAGGAGGATAGC
TACGACTTCGAATTCCGCTCCACGGTTTTCAAATTTGGCGACATTCCAGTTGCACTAATAGGGAAAACAAAAG
GAGAGGGAAAATGGTTTGATGAGAAAACAAAAATTTTCCTTGAAGGAGAGAAAGTCATTGGTGCTGTTGTTAT
AGAAGACGTAAGAAAGGCAATGATGCTGGAGAAAAAGGCAAAAGCTGGGGTTAGCATAGATGAATTATAA

>hhi:HAH_1235-Ar nosZ; nitrous-oxide reductase (EC:1.7.2.4); K00376 nitrous-oxide reductase [EC:1.7.2.4] (N)
ATGAGCAAAAACGAAACCCCACGCGATCCGAACGAAGTCCTCGAAGAGTACGAAGAAACCCTCGACTCGGTAC
TGGTGGAGGTCGAGTCTCCGGACGAATCGACTGCGGACGACGACCTCTCGCTGGGACTGCCCGGCCTCTCGCT
CGGCCGCCGGGACTTCATGAAGGCCGGCGTCGCCGCCGGTGCGATGGGGTCCGTCGCTGGCTGTTCAGCGCTG
ACCGGCGGTGGCGATGGCAGTGCCGGCAGCCAGTCGACACCCTCGGGCAGCGGCGCGAGCCACACGGTCGAAC
CGGGCGAACACGACGAATACTACGGCTTCTGGTCCGGCGGCCACTCCGGCGAACTGCGGGTCATCGGCATCCC
GTCGATGCGGGAGCTCACCCGCATTCCGGTGTTCAACACCGACTGTGCGTCGGGCTATGGGTTCACTGACGGG
ACTCAGGAAATGCTGGAAGAGGGCGGCGGGTATAGCTGGGGTGACAATCACCACCCGAACCTCTCGGAGACGG
ACGGTGACTACGACGGCGAATACCTGTACGTCAACGACAAGGCCAACGGCCGCATCGCCCGTGTGAACCTCAC
GTATTTCGAGACGGACGCCATCACGGACGTCCCGAACATGCAGGCCATCCACGGCTGTTGTGTCCTCTCGCCG
GACACGAAGTATGTGCTGGGCAACGGCGAGTTCCGCGTGCCGCTGCCCAACGACGGGACCGACGCGAAGAACC
CGGACAACTACACGTCGCTGTTCGTCGCCGTCGACCCCGAGTCGATGGAGACCCAGTGGCAGGTCAAAGTCGA
CGGGAACCTCGACATCGTCGACACCGGCAAAGAGGGTCGGTGGGCCATCTCCTCGGCGTACAACAGCGAGGAG
GCCACCGACATCCAGGGCATGACGAAAGACGACCGGGACAACGTCAAGGCGTTCGACATTCCGGCCATCGAGC
AGGCCGTCGAGAACGGCAACTACGAGGAGGTCAACGGCATCCCCGTCGTCGACGGCACGCAGGGGAGTTCGCT
CAACCAGGGCGACCGCCCCATCGTGAAGTACATTCCGACGCCGAAAAGCCCCCACTGTGTCGAGGTCGGCCCG
AACGGCGACTACGCCTTCGTCGCGGGGAAGCTCTCGCCGACGGTGACGATGATCGACCTGAACGCGCTGGCCG
ATTCCTCCGACCCCGAAGATGTCGTCGCCGGGCGACCACGCGTCGGACTCGGCCCGCTACACACTACCTTCGA
CGGCAACGGACACGCCTACACGTCGCTGTTCATCGACTCACAGGCCGTCAAGTGGGACATCGAGGCGGCCGTC
GAGGCCGAAGAAGGGTCCGAGGACCCCATCATCGAGAAGCAGGACGTCCACTACAACCCCGGGCACATTCAGG
CCCTCGAGGCGATGACGACCGACCCCGACGGGGAGTGGCTCGTCAGCCTCAACAAACTCTCCAAGGACCGGTT
CCTCCCGGTCGGCCCGATTATGCCCGACAACGACCAGCTCATCCACATCGGGCAGGGCGAGGAGGAGATGGAA
CTGGTCGCGGACCACCCGGCCTACCCCGAGCCACACGACTGCGTGTTCGCCCACAAGGACAAGATAGACGCCA
AGAAGGTCTACGACAAGGACGACTACGAGGAGACCTACATCACCGAGGAGGACTCCGGCGTCGAGCGCACGGG
CGAGAACAGCGTCCACGTCAAAATGACGACCAAGCGCTCGGAGTTCGGCCTGCCGGACTTCACGGTTCAGGAG
GGCGACGAGGTGAAACTCTCCACGACCAACATCGAGGGCGTACAGGACATCATCCACGGCGTTGCCATCCCCG
AGCACGACATCAACTACGCCGTGGCCCCACAGGACACCCGCGAAGTCACGTTCACGGCTGACGACCCCGGGGT

GTACTGGATCTACTGCACGTACTTCTGTAGCGCCCTGCACCTTGAGATGCGCAGCCGGATGATCGTCGAACCG
GCGGAGGGGTAG
>hma:rrnAC0390-Ar nosZ; nitrous-oxide reductase; K00376 nitrous-oxide reductase [EC:1.7.2.4] (N)
ATGAGTAAGCACGAAACCCCACGCGACCCGAACGAAGTCCTCGAAGAGTACGAAGAAACGCTCGACTCGGTGC
TGGCGGAGGTCGAGTCCCCCGATGAGGCGACAACGGATGACGACATCTCCCTGGGACTACCCGGCCTTTCGCT
CGGTCGCCGGGACTTCATGAAAGCCGGCGTCGCCGCCGGTGCGATGGGGTCGGTGGCCGGCTGTTCAGCGTTG
AGCGGCGGCGACAGCAGCGCTGGCAGCCAGTCAACGCCCTCGAGCAGCGGTGCGAGTCACACGGTCGAGCCGG
GCGAACACGACGAGTACTACGGCTTCTGGTCCGGCGGCCACTCTGGCGAACTGCGGGTCATCGGCATCCCGTC
GATGCGTGAACTCACCCGTATTCCGGTGTTCAACACCGACTGTGCGTCGGGCTATGGCTTCACCGACGGCACG
CAGGAGATGCTGGAGGAAGGCGGTGGCTACAGCTGGGGTGACAACCACCACCCGAACCTCTCGGAGACGGACG
GCGACTACGATGGCGAGTATCTGTACGTCAACGACAAGGCCAATGGCCGCATCGCCCGCGTGAACCTCACGTA
CTTCGAGACGGACGCAATCACTGACGTCCCGAACATGCAGGCCATCCACGGGTGCTGTGTCCTTTCGCCAGAC
ACGAAGTACGTACTGGGCAACGGCGAGTTCCGCGCGCCGCTGCCAAACGATGGGACCGACGCGAAGAACCCGG
ACAACTACACGTCGCTGTTCGTCGCCGTCGACCCCGACTCGATGGAGACCCAGTGGCAGGTCAAAGTCGACGG
GAACCTCGACATCGTCGACACCGGCAAAGAAGGTCGGTGGGCCATCTCCTCGGCGTACAACAGCGAGGAGGCC
ACCGACATTCAGGGCATGACGAAGGACGACCGGGACAACGTCAAGGCCTTCGACATCCCGGCTATTGAGCAGG
CTGTCGAGAACGGCAACTACGAGGAGGTCAACGGCATCCCCGTCGTCGACGGCACGCAGGGAAGTTCGCTCAA
CCAGGGCGACCGCCCCGTCGTGAAGTACATTCCGACGCCGAAAAGCCCCCACTGCGTCGAGGTCGGGCCGAAC
GGCGACTACGCTTTCATCGCGGGGAAGCTCTCCCCGACGGTGACGATGCTCGACCTGAACGCGCTGGCCGACT
CCTCCGACCCCGATGAGGTCGTCGCCGGGCGACCGCGTGTTGGGCTCGGCCCGCTTCACACCACCTTCGATGG
GAACGGGCACGCCTACACGTCGCTGTTCATCGACTCTCAGACAGTCAAATGGGACATTCAGGCCGCCGTCGAG
GCCGAAGAAGGGTCCGAGGACTCCATCATCGAGAAGCAGGACGTCCACTACAACCCGGGCCACATTCAGGCCC
TTGAGGCGATGACGACCGACCCGGACGGAGAGTGGCTCGTCAGCCTCAACAAGCTCTCGAAAGACCGGTTCCT
CCCGGTCGGCCCCATCATGCCCGATAACGACCAGCTTATCCACATCGGGCAAGGTGAAAAAGAGATGGAACTG
GTCGCGGACCATCCGGCCTACCCCGAGCCACACGACTGCGTGTTCGCCCACAAAGACAAGATAGACGCCAAGA
AGGTCTACGACAAGGACGACTACGAGGAGACCTACATTACCGAGGAGGACTCGGGTGTCGAGCGCACGGGCGA
GAACAGCGTCCACGTCAAGATGACGACCAAGCGCTCGGAGTTCGGCCTGCCGGACTTCACGGTTCAGGAGGGC
GACGAGGTGAAACTGTCCACGACCAACATCGAGGGCGTACAGGACATCATCCACGGCGTTGCCATCCCCGAGC
ACGACATCAATTACGCCGTGGCGCCACAGGACACCCGGGAGGTCACGTTCACCGCTGACGACCCCGGCGTGTA
CTGGATCTACTGCACGTACTTCTGTAGCGCCCTGCACTTGGAGATGCGCAGCCGGATGATCGTCGAACCGGCG
GAGGGGTAG
>hbo:Hbor_30430-Ar nitrous oxide reductase apoprotein (EC:1.7.99.6); K00376 nitrous-oxide reductase [EC:1.7.2.4] (N)
ATGAGTTCACACGACACATCGGAGGGGGAGGAGACCGACACCAAAACAGAGCGTAGCGCAGAGACATTACTAT
CGGAACACGAGGCGCAGTTGAATGCGCTTATTGCAGACGTACAGGGGCCGGACGCGGACCCAGTAGAGACGCA
ACTTCCGAATCTCGGTCTTGGTCTCGGTCGCCGGGACTTCATGAAAGCGGGGGCAGCCGTCGGGGTTCTGGGC
TCCGTCGCCGGGTGTGCAGGGCAGGGCGACAGCCAGGGTGGTACAGCCACGCCGTCTTCGAGTCACGGCGATG
TGCCCGACCACTTCGTTCCGCCGGGCGAACACGACAAGTACTACGGCTTCTGGAGTGGCGGTCACTCCGGCGA
AGTGAGAGTAATGGGAATTCCGTCGATGCGGGAACTCACGCGAATCCCCGTGTTCAACAGGGAGTGCGCGAAG
GGATACGGTTTCGACGACGGAACGGGGGAGATGCTTAAAGACGCAGGCGACTACACGTGGGGTGACTCTCACC
ACCCGAGCTTATCCGAGACCGATGGGAAGTACGACGGCGAGTACCTATTCGTCAACGACAAAGCCAACGGCCG
CGTCGCACGGGTGAATCTGAAGTACTTCGAGACGGATGCGATTCTGGACGTGCCGAACGTCCAGTCAGTCCAC
GGATGCTGCATTCAGAGCCCCGACACCGAGTACGTCTTCGCCAACGGTGAGTTCCGTGCGCCGCTTCCAAACG
ACGGGCGCGACGTAGACGACCCGTCGAAGTACGTCTCGCTGTTTTCGGCCATCGACCCCGAAAGCATGGACGT
ACTCTGGCAGGTGAAAGTCAGCGGCAACCTCGACATCGCCGACTCCGACAAAGACGGCCGGTGGCTGCTCTCG
TCGTCGTACAACGACGAAGAGGGGGTCGAAATCGAGGAGATGACCCGCGACGACCGCGACTCGGTGAAGGCGT
TCGACGTACCAGCCATTCAGAAAGCGGTTGATGCCGGGAAGTACGAGGAGGTCAACGGCGTCCCCGTCGTCGA
TGGGACCCGTGAAAGTTCACTCAACAAGGGCGATAACCCACTCGTCCGCTACATCCCGACGCCGAAATCACCC
CACTGCGTTGAGGTGACGCCGGACGGAAAGTACGGCATCGTCGCCGGAAAGCTCTCTCCGACCGTCTCGATCA
TCGATATCGAGAAACTCGGAACCGAACCCGACCCGGCGAAGACGATTGTCGGCCAGCCCAAACTCGGTCTCGG
TCCACTGCACACTACGTACGACGACCGCGGTCACGGGTACACCTCGCTGTTCATCGACTCGCAGGTCGTCAAG
TGGGATATCGAAAAGGCAGTGGAGTCACCAAAAGGCTCCGAGGAGGCCATTCTCGGGAAGATAGACGTCCACT
ACAACCCAGGACACATCCAAGCGGTACAGGCAATGTCCGTCGAACCGACCGGCGATTGGCTCGTCGTCCTCAA

CAAACTGTCGAAGGACCGGTTCCTCCCAGTCGGACCGATTCACCCCGACAACGACCAACTCATCTACATCGGA
AACGACAAAGACAACGAAACCGGTGGAATGGAACTGGTTTCCGATCACCCCGTCTATCCCGAACCGCACGACG
CCATCATGGTCCACAAAGACAAGTTGGACCCGGCGAAGATATGGGATGCCAAGGACTACGAGGGAGAACGGGA
GTACATCTCCGAGAAGAACTCGGGAATCGAACGCCTCGACGACTCGACCGTCGAGGTGAACATGAGTTCCAAG
CGGAGCGAGTTCGGCCTCCGTGAATTCACGGTGAAAGAAGGCGACGAAGTGCAGATGACCGTGACGAACATCG
AGAAGAGTCCGGACATCATCCACGGAGTTGCCATCCCCGAACACGACATCAACCTCGCGCTCGCCCCACAGGA
CACTCGGGAAGTAACGTTCGCCGCGGATGATCCCGGCGTCTACTGGATCTACTGCACGTACTTCTGTAGCGCA
CTGCACCTAGAGATGCGCTCGCGGATGATCGTCGAACCACGGGACTGA
>fpl:Ferp_0128-Ar nitrous-oxide reductase (EC:1.7.99.6); K00376 nitrous-oxide reductase [EC:1.7.2.4] (N)
ATGGCGGAGGAGAAGAATGCGAAAAAGAACAACAAGAGAAACAATAAAAACGGGGAGACTAAATTAACAAGAA
GAGAATTTCTTAGCTTCGGAGGAGGTTTTATCGCTGGATTTGCCGTTGGAGCTGGAATTTCGAGGCTTGGTGG
AGGAGGAGCACCAGCAGCTCCAGCCTCTTTTGCTGAAATAATGGAGCAGAGAGGGTTGACGCAGCAGGATGCT
ATTGCCGCTTTAAAAACCTACACTCCCGGAGGAAAGTACGACGAGTTCATAATGTTTGCCTCTGGAGGACATT
CTGGGCAGGTGCTCGTAATCGGAGTTCCGAGCATGAGATTGTACAAAGTAATAGCCGTATTCACCCCAGAGCC
TTGGCAGGGATACGGATATGGACTGAAGGAAACCCATGAAGTTCTTAAAGGAGGGTATAACGGAATTCGCCCA
TTGAGCTGGGGAGACACTCATCATCCTGAAATCAGCAGAACCAATGCTGAGTTTGACGGAGAATGGCTGTTTA
TTAACGATAAAGCAAACGGCAGAGCTGCTGTTATAAGCCTCAAGGACTTCGAGACGAAACAAATAGTAAAAGT
TCCGAACACTCAAACAGTCCACGGAGGGTGCTTTGCCACTCCAAATACTGAATACGTTGCAATTGTCAGCCAA
TATGCAACCCCATGGGATCCTGAAAAAGGAGGATACATCCCCGGAGAAACGTATGTTGAGTTGACTGAGGAGA
ACTACAAGAAGTACTTCAGAGGAACTTACACGTTTTTGGCTTTCGACAGAAAGAGGGGAAGATTCGACCTATC
AAAGAGCTTCCAGATAGAGCTTCCTCCGTACACGCAGGATCTTGTAGCTGTAGGATGGGGTCCGAGCGATGGT
TTGGCTTTCTGCAACTCTTTCAACACCGAGTTGGCTGTTGGAGGTGTCTTAAAAGGAAAACCTCCACTTGAGA
TTGGAATGTCCCAGAACGATTTCGACTATCTTCACGTAATTCTGTGGAAGAAGGCTGAGCAGTACGTTAAGGA
AGGAGGAGGAGAAGTGATAAACGGGATAAGAGTAATAAGGTTGAAGGAGGCTGTTAAGGAGGGGCTGATTTAC
TTTGTTCCCGAACCAAAAAGCCCCCACGGATGTGATATAACGCCGAACGGAGATTACGTTTGTGTCAGTGGAA
AGCTCGATCCCGTTGTGACAGTCTATAGCGTGGAGAAGATAAAAAGGGCTATCGAAGAGAAGAAGTTCGAAAA
GAAAGATCCCTACGGTGTTCCAGTTTTGAAGTTTGACGAAGTTGTTGAGGCTCAGATAGAGGTTGGTTTAGGT
CCTCTCCACACACAGAATTCGACGATAAAGGCTATGCTTACACAAGCCTCTTCATAGACAACGCTGTAGCAAGAT
GGAAGCTTGGTCCACCCTACAACGAAGATGGATGGAAAGTTGTTGAAAGAATTCCCATAAACTACAACGTTGG
ACATCTGGCAACTCCGGAGAGTAACTCTCCGACGCCAAAAGGAAAGTATCTCGTTTCCCTAAACAAGTGGTCG
ATCGAAAGATTCCCGCTTGTCGGTCCACTGCATCCGCAGAACTTCCAGTTAATTGACATAAGTGGGGAGAGAA
TGCAGCTCCTTTACGACATGCCCATAGGACTTGGTGAACCTCACTATGCGAAGATAATCAGCATCGAGAAACT
TAAGCCACTCGATGTTTACCCAGCTGGAACTGACCCGCTTACCTTCCAGCCACATCCGAAGGCAATACAGCAG
GGACAGGAGAGAATTGAGAGAAAGCAGGAGAACGGAAAGTGGGTTACTGAGATTTATGCTACATTAATCAGAA
GTACCATAACTCCTGACATAATGAAAGCCAAAGTGGGAGACATCGTGAGAATTTATCTGACGAACGTTGAATA
CACGAGAGATGCAACACATGGATTCTGTATCGCAGAGTACAACATAAACGCGAGTCTTGAGCCGGGAGAGACT
GGAACGATAGAGTTCGTTGCGGACAAGCCAGGAGTTTATGCGATCTACTGTACGGAGTTCTGTTCACCACTCC
ACCTGGAAATGGCCGGTTGGTTAGTAATTGAACCATAA
>hla:Hlac_1165-Ar nitrous-oxide reductase (EC:1.7.99.6); K00376 nitrous-oxide reductase [EC:1.7.2.4] (N)
ATGACTACAGATCCGAACGCGAGCGACGAGCGGACGGCACGGACCGACGAACAACGCGACGAGCAGTCGGCGA
ACGCGAATGCGGGCGACGCGAAGACAGCGGGATCGGCCGCGGACGGCACCGAGACGGCGGACGACGGTCCGGA
GATCGTCGACGGCCGGACGATCGAACACTTGTTGACCGATCACGAACGCGACATCGCCGCCACCGCGGAGGGG
ACCGAGGCCACCGCGGCGAAGCGGCCGAGCCTCGATCTGCCGCGGTTGGAGCTCGGACGCCGCGACTTCATGA
AGGCCGGCGCCGCCGTCGGGGCCATGAGCGGTCTCGCCGGTTGTACGAGCATCCTCGCCGACGAGGAGAGCAA
CGACTCGGGCGACGCGGCCGCCAGCGGCGAGCACTCGGTGGCTCCGGGCGAGCACGACGAGTACTACGGGTTC
CTCTCCGGCGGCCACACCGGCGAGATCCGCGTCGTCGGGCTCCCGTCGATGCGGGAGCTGATGCGGATCCCGG
TGTTCCAAGCGGAGAGCGCCCGCGGCTTCGGCCACGACGCGAAGTCGAGCCAGATGCTGGAAGAGGAGGGCGG
CGGCTACACGTGGGGCGACACCCACCACCCGCGCGTCTCCCAGACCGACAACGACTACGACGGCCGCTGGCTG
TTCGTCAACGATAAGGCGAACGGGCGGATGGCCCGGGTCGACTTGGAGTACTTCGAGACCGACGCGATCACGA
ACGTCCCGAACTGTCAGGGCGTCCACGGGGCATGCATGCAGCTACCTGACACGCAGCTGGTCTTCGGCGTCGG
CGAGTTCCGCGTCCCGATGCCGAACGACGGCCGCGACGTGCAGAGCCCCGACGAGTACGGCTCCGTCCTCAGC
GCCATCAACCCCGAGACGATGGACGTCGAGTGGCAAGTCGAGGTCGACGGCAACATGGACAACGGGGACGGCG

GGAAGGAGGGGCGCTGGTTCTTCGCGACCGGCTACAACAGCGAGGAGGGCGTCACGGAGTCCGAGATGGCCCG
GTCGGACCGCGACTACGTGAAGGCGTTCGACATCCCCGCGATCTGGGACGCCGTCGAGGCCGGGAACTACGAG
GAGATCAGCGGCGTGCCCGTCGTCGACGGGACGATGGACAGCTCGCTCAACGAGGGCGACCGTCCGATCGTCC
GGTACGTCGCCACACCGAAGAGCCCGCACGGGATCAGCGTGACGCCCGACGGGAACTACGCCATCGCCTCCGG
CAAGCTCGATCCGAGCTGTACGGTCATCGACATCGACGCGATCGCCGAGGTCGACGACCCGCAGGAGTCCATC
GTCGGGCAGCCCCGGATCGGGATGGACCGCTCCACACCGCCTACGACGGGCGGGGGCACGCGTACACGACCC
TGTTCATCGACTCGCAGGTTGCCAAGTGGGACTACGAGGCCGCCGTCGAGGCCGAGGCCGGCTCCGAGGAGCC
GGTGATCGAGAAGCACGACGTACACTACAATCCCGGCCACCTCATCGCCTCGGAGTCGTACACGGCGTCGCCG
CAGGGCGACTACCTCGTCTCGCTGAACAAGCTCTCGAAAGACCGGTTCCTCCCCGTCGGACCGATCCATCCGG
AGAACGATCAGCTGTTCCACATCGGCGACGACGACGCCGGGATGGAGCTGATCAAGGACAAGCCGTCGTACCC
CGAGCCGCACGACGCCAGTATCGTCCACAAGGACAAGATCGAGCCGGCGAAGACGTGGGACACCGACGACTAC
GAGATCGACTACGTCGCCGAGGGCGAGGAGTCCTTCGAACGGGTCGACGACAGCACCGTCGAGATCGAGATGT
GGGCGCGCCGCAACGAGTACGCCTTCCCGGAGGTCACGCTGCAGGAGGGCGACGAGGTGACGCTCCGGATCTC
CAACGTCGAGACCACGAGCGACGTGATCCACTCGATCGCGATCCCGGAGTACGACATCAACCTCGCGTTAGCG
CCCCAGGACACCCGTGAGGTGACGTTCACCGCGGACCAGCCCGGCGTGTTCTGGGCGTACTGCGCGTACTTCT
GTAGCGCGCTCCACCTAGAGATGCGCTCGCGGATCCTCGTCGAACCGGACGAATAG
>hxa:Halxa_1765-Ar Nitrous-oxide reductase (EC:1.7.99.6); K00376 nitrous-oxide reductase [EC:1.7.2.4] (N)
ATGACCGACGAACCTACTTCCCCCACGCAGGACTCGAGCGAGACCGACGCGGGCACCGAGCGAGAACCGCTGT
TCGATCGCATTCCGCGCCGCGACTTCATGAAGGCAGGTGCAGCCGCCGGTGCGATGGGCTCGCTCGCCGGCTG
TACGGGCCTGCTCAGCGACCAGGACGACGGGCTGGCCTCGGCGTCCGACGTCGATGCGAGCGTGCCGCCGGGC
GAGCACGACGACTACTACGCGTTCCTCTCCGGCGGTCACTCCGGCGAGATCCGCGTCTACGGCGTGCCGTCGA
TGCGGCAACTGATGCGCATTCCCGTCTTCGGCCGGGAGAGCGCCCGCGGCTACGGCTTCGACGACCGAACGAG
CGAGATGCTCGAGGACGCGGGCGGCTACTCGTGGGGCGACACCCACCACCCGCGCGTGAGCCAGACGGACAAC
GACTACGACGGCCGCTGGGCGTTCGTCAACGACAAGGCGAACGGCCGGATGGCCCGGATCAACCTCAAATACT
TCGAGACGGACGCCATCGTCGACATTCCGAACCAGCAGGGGACCCACGGCGCCTGCTGTCTGCTGCCGGACAC
GAAGTACGTCTTCGGCGTCGGCGAGTTCCGCGTCCCGATGCCCAACGACGGGCGGGACCTCACCGATCCCGAC
AGCTACACGTCGACTATCGCCGCCATCGACCCCGAGACGATGAACGTCGAGTGGGAGGTGCTGGTCGACGGTA
ACATGGACAACGGCGACGGCGGCAAGGAGGGACGGTGGTTCTTCGCGACCGGCTACAACAGCGAGAACGCCAC
CACCGAAAGCGAGATGTCCTCCTCGGACACCGACTGGCTGAAAGCCTTCGACGTGCCCGCCATCGAGGAGGCC
GTCGAGAACGGCCAGTACGAGGAGATCGGCGGCGTCCCGGTCGTCGACGGCACCCGCGACAGCGACCTGAACG
GCGGCGACCGACCGATCGTCCGCTACATCGACGTTGCGAAGAGCCCCCACGGGGTCAGCGTCACGCCGGACAA
CAAGTACGCGATCGCCAGCGGCAAGCTCGACCCCACGTGCTCGGTGATCGACATCGAGCAACTCGCCGAGGTC
GACGACCCCAACGACGCGATCGTCGGCCGGCCGAGCCTCGGAATGGGGCCGCTGCACACCGCCTACGACGGCC
GCGGCCACGCCTACACGACGCTGTTTATCGACTCGCAGGTCGTCAAGTGGGACATCGAGGCCGCCGTCGAGGC
CGAGGCCCAGTCCGAAGATCCGGTCGTCGAGAAGATCGACGTCCACTACAACCCCGGCCACCTGATCGCCAGC
GAATCGTACACGGCCGATCCGCAGGGCGACTGGCTGATCTCGCTGAACAAGCTCTCGAAGGACCGGTTCCTGA
ACGTCGGCCCGCAAAAACCCGAAAACGACCAGCTGATCTACATCGGGGACGACGAGGAGGGGATGCAGCTCGT
GAAGGACACGCCCTCCTACGCCGAACCGCACGACGCGTCGATCGTCAGCGCGGACAAGATCCAGCCCGCGGAC
ATCTACGACCCCGCGGACTACGAGGAGGAGTACGTCGACCACGGCGAGTCTGAAGTCGTTCGCGAGGACGGGA
GGGCCCACGTGAAGATGTACTCGACGCGCAACGAGTTCGGCCTCCACGAAGTGACCGTCCAGGAGGGCGACGA
GGTGACGATGACCGTCACCAACATCGAGCAGACGCCGGACATCCTCCATTCGCTGGCGATCCCCGAGCACGAC
ATCAACATCAAGCTCGCACCGCAGGAAACCCGCGAGGTCACCTTCACGGCCGACGAGCCCGGCGTCTACTGGA
TGTACTGTGCGTTCTTCTGCAGCGCGCTGCACCTCGAGATGCGCTCGCGACTGATCGTCGAACCCGCGGACGA
GTAA
>pcl:Pcal_1928-Ar nitrous-oxide reductase (EC:1.7.99.6); K00376 nitrous-oxide reductase [EC:1.7.2.4] (N)
GTGGGGCTGGTGGGGGGCCTCGCCTTAAGCCAGCTCTTGGCCAAGGGGGCTGTGGCGCCGGCGCCCCCCGGCG
TGTCGGCACGGGTCCAAGAATTGGCCGCGGCAAGAGGGCTGTCGCCTCAAGACGTAGAAAATGCCGTGGCCAC
GTATCTCCCCGGAGGGAAGTACGACGAGTTCTACATGTTCTCAAGCGGCGGCCACTCGGGGCAGGTGGCGGTC
TACGGCCTTCCCTCTTTGAAGCTCTACCGCATAATAGCCGTATTCACTCCCGAGCCTTGGCAGGGCTGGGGCT
ACCACGATAGGAAGACCATGGAAGTTCTCTTCAGCGAGAGAGCCACCGCTGGCAGGCCGCTCAGCTGGGGCGA
CACCCACCACCCCGAGTTCGATAGGCAAAACGCCGAATATGTCGGCAGATGGCTGTTCATTGGCGACAAAGTG
AACGGGAGAGCAGCCGCCATTTCCCTACTAGACTTTGAGACCAAGGACATTGTAAAAATCCCCAATGTACAAA

CTATCCACGGCGGCGCCTTCACTACTCCAAACACGGAGTACGTCTTATACGCCACGCAGTATCCAGCGCCTTG
GGAGCCGGGCAGAGGATATAAGCCAGGTGTCACCAAGGTCCCGTGGAGCCAAGTGGTAAAAGATGAAAAGTAC
TTCTGGGAGTACATGAGGGGTGCCCTCACGTTCCTCGCCTTTGACCGTAACACTGGGAGATTCGACTTGTCGA
AAAGCTTCCAAATAGAGGTCCCGCCCTATGTGCAAGACCTTTCCACAATATCCTGGGGCCCCGGCGACGGTTT
GGCGTTTGTCAACTCCTTTGGCACAGAGGGCATGTTCGGAGAGACGCCATATGAGGCAAAGTTGTCTAAGAAC
GACTACGACCTGTTACACATAGTGCTGTGGAGAAAGGCTGAGGACCTCTGCTTGAGGCAAGGCCGTTGCAAGG
AGATAAACGGCATAAAAGTCCTCTCCCTAGAGGACGCGATTAAGGAGGGGGTGTTGTTCTTTGTTAGAGAGCC
CAAGAGTCCCCACGGCGTAGATACATCCCCCGGCGGGCACTTTATATCTGTGGGAGGCAAACTGGCCCCCTAC
GCCACAGTCTACAGCACGGAGAAGATAAAGCAGGCCATTGCAGATAAGAAGTTCGAGGGGCAGGACAGATACG
GAGTGCCTATATTGGACTACGGTAGCGTGGTTGCGGGACAGGTGGAGGTCTGCCTAGGCCCACTACACACAGA
ATTCGACGGGAGAGGCAACGCCTACATCAGTTGCTTCGTGGAAAATGTAGTAACAAAATTCACCCTAGGCTCG
CCCGAGTACAGAGGCGATAAGCCGTTCACAGTGGTAGACAAAGTGCAGATACACTACAACGTGGGCCACATAT
GTGTGCCAGAGTCTAATTCGCCTAATCCAGCTGGGAAGTATCTAGTGGCGTTGAACAAGTGGTCAATAGACAG
ATTCTTCCCAGTGGGGCCGCTTAAGCCTCAGAACTTCCAGCTAATAGATATATCTGGTGATAAGATGAGGCTA
CTCGCAGAGGTGCCCGTGGGCTGGGGCGAGCCGCACTACGCCAAGGCTATACGGGCTGAGAAGATAAAGCCGC
TTGACGTATATCCAGTGGGCACGGACCCCCACGTCATGAAAGATGGCCAGTTCCAGTTGAGCTCCAATGCGGT
GACTGAAATAGGCAAGGAGCGGGTGCAGAGGTATCAGCTACCAGACGGGAGGTGGGTCACTGAGGTTTGGGGC
ACGGTGATTAGGTCGACTATAAGGCCTAACATTATTGTGGCAAAGAAGGGCGACATAGTGCGCCTCCACTTAA
CCAACATAGAGAAGAGCGTAGATGCCACACATGGCCTTGCCATTGCTGAGTACAATGTGGCGGCCTCGCTGGA
GCCCGGCGAATATGCCGAGATAGAGTTTGTGGCAGATATACCCGGCGTATTTGCCTTCTACTGCTACGAGTTC
TGCTCACCGCTCCACTTAGAAATGGTGGGATGGCTGGTAGTAGAGGAGTAA
>hah:Halar_1529-Ar Nitrous-oxide reductase (EC:1.7.2.4); K00376 nitrous-oxide reductase [EC:1.7.2.4] (N)
ATGTCCTCAGAACCAACAGAAACTGACGAGCAGCAAGACCCGCTCGCTGAACACGAACAGCAACTGAACGACC
TGCTGCTGGATGTCGAAGGCCCGGACGCCCCTGACGCCGAGAGTCACCTCTCGGGATTGGGGCTTGACGTCAG
CCGCCGGGGCTTGATGAAGGCTGGCGCCGCCGCAGGCGCCATTGGAGCGTTCGCTGGCTGTGCCGGCCTGCAG
GGCGGTGGGGCAAACACCGAGGCCAAGGGCGGCAGTGGTGACGTGCCAGACCACATGGTCCCGCCGGGTGAAC
ACGACGAGTACTACGGCTTCTGGTCCGGTGGCCACTCCGGCGAGATTCGGGTGATGGGGCTCCCCTCGATGCG
GGAACTCACCCGGATTCCGGTGTTCAACCCTGAGCCTGCGAAAGGCTACGGCTTCGACAACGAGACCAGCGAG
ATGCTGGACGACGCCGGCGGCTACACGTGGGGTGACTCCCACCACCCGAACCTTTCGGAGACCGGCGGTGAGT
ACGACGGCCGGTACATCTTCGTCAACGACAAGGCCAACGGCCGAGTGGCTCGGGTGAATCTGAAATACTTCGA
AACCGACGCCATCGTCGACATTCCGAACGTCCAGTCCGTCCACGGCTGCTGTATCCAGAGCCCGGACACGGAG
TACGTGTTCGCCAACGGTGAGTTCCGGACGCCGCTGCCCAACGACGGCCGGGACGTCAACAACCCTGAGGAGT
ACGTCTCCCTGTTCAGTGCCATTGACCCGGAGACCATGGACGTGCGCTGGCAGGTGAAAGTCGACGGCAACCT
CGACATCGTTGACTCCGACAAGGACGGTCGCTGGGTGCTCTCCTCGGCATACAATGACGAGGAGGGTGTCGAG
ATTGAAGAGATGACCAAGGACGACCGGGACTCCGTCAAAGCGTTCGACGTCCCGGCCATCCAGGAACTAGTCG
ACGCCGGCGAAGGCACCGAAGTCGACGGAATCCCAGTCATCGACGGCACCGAGTCGAGCAAACACAACAGCGG
CGACGACCCGATTGTTCGGTACATCCCGACGCCGAAGTCGCCCCACTGTGTCGAAGTCACGCCCGATGGAAAG
TACGGAATGGTCGCTGGGAAACTCTCCCCGACCGTCTCCATCATCGATATCGAGAAGCTGGAGTCGGCATCCG
ACCCTGCCGACACCATCATCGGCCAGCCTAAAGTCGGGCTGGGACCGCTGCATACGACCTACGACGGCCGCGG
GAACGCCTACACCTCGCTGTTCATCGACTCGCAGGTGGTCAAGTGGGATATCGAGGCGGCGGTCAACGCCGAG
AAGGGCTCCGAAGAGCCGATTCTCGGGAAGATCGACGTCCACTACAACCCCGGCCACATCCAGGCTGTCCAGG
CCATGTCCTCGGAGCCGACCGGTGACTGGCTGGTCGTACTGAACAAGCTCTCGAAAGACCGGTTCCTCCCGGT
CGGGCCGATCTACCCGGACAACGACCAACTGGTCTACATCGGCAACGACAAAGACAACGACGCCGGCGGGATG
GAGCTGGTCTCGGACCACCCAGTCTACCCCGAACCGCACGACGCGGTGTTCGCCCAGAAAGACAAACTCGACC
CCGCGACCACCTGGGAGCCAGAGGACTACGAGGGCGAGAAAGAGTACATCTCGGAGGATGCCTCGACCGTCGA
GCGTATCGACGACAGCACCGTCGAGGTCAAAACCTCCGTCAAACGGAGCGAGTTCGGCCTTCGTGACTTCACG
GTTCAGGAGGGCGACGAGGTCCGCCTCACTGCGACCAACATCGAGGGGAGCAAGGACATCGTCCACGGGATGG
CCATCCCCGAGCACAACGTGAACCTCGCGCTCGCGCCCAAGGACACGCGCGAGGTCACCTTCACGGCGGACAA
GCCCGGTGTGTACTGGATCTATTGCACGTACTTCTGCAGTGCCCTGCACCTTGAGATGCGCTCACGAATGATC
GTCGAACCAGAGGACTAA

>hbo:Hbor_35040-Ar respiratory nitrate reductase alpha subunit apoprotein; K00370 nitrate reductase 1, alpha subunit [EC:1.7.99.4] (N)
ATGAGCCGAAACGACGCATCACACACCGACGAGACGGCCGGAGAATCGGACGATAAACGGCGGGCAGTCGATC
AGTCGGACGACGAGCAACCGAAGAAGCGAGGCGCAAACGAGGAACGAACTGACGGCGGCCCCGCCGTCGGCGA
CCCGCCGGGAGGTGACAACCCGTCCCGACGCGGGTTCCTCAAGGGTGTCGGACTCGCGTCCGTCCTCGGAATC
GGGAGCGCGTCGGCGTCCGACGACGCCCTGTTCTCGATGGACGGACTCCAACCGGTGGGTGACCCCATCGGTG
AGTATCCGTATCGTGACTGGGAGGACCTCTACCGCGAGCAGTGGGACTGGGACTCAGTATCTCGTTCGACTCA
CAGCGTCAACTGCACCGGCAGTTGCTCCTGGAACGTCTACGTCAAGAACGGGCAGGTCTGGCGCGAAGAGCAG
TCCGGCGACTACCCCCGGTTCGACGAGTCACTGCCCGACCCGAACCCGCGCGGGTGTCAGAAGGGGGCGTGTT
ACACCGACTACGTCAACGCCGAGCAGCGCATCAAACACCCGTTGAAGCGCGTCGGCGAACGTGGCGAGGGCAA
GTGGAAGCGAATCACGTGGGACGAAGCCCTCACCGAGATTGCCGAACACGTCGTTGACGAGGTAGAGGCCGGA
CGGTACGACGCTATCAGCGGCTTCACGCCGATTCCCGCGATGAGTCCCGTCTCGTTCGCTAGCGGGTCGCGGC
TCATCAACCTGCTCGGCGGCGTCAGTCATTCGTTCTACGACTGGTACTCCGACCTGCCGCCGGGCCAACCTAT
CACGTGGGGCACGCAGACGGACAACGCCGAGAGCGCAGACTGGTACAACGCCGACTACATCATCGCGTGGGGG
TCGAACATCAACGTCACGCGCATCCCGGACGCGAAGTACTTCCTCGAATCGGGCTACAACGGCACCAAGCGCG
TCGGTATCTTCACCGACTACTCGCAGACGGCCATCCACACTGACGAGTGGCTTAGCCCTGACCCCGGCTCCGA
CACTGCACTCGCACTCGGGATGGCACAGACAATCGTCTCCGAGGGACTGTACGACGAGGCGCATCTCAAAGAG
CAGACCGACATGCCGCTTCTCGTCCGGCAGGACACCGGTAAGTTCCTCCGTGCGAGCGACGTCCCGTCGGTCA
ACAGCAGCGCCGACCGGCCCGAATGGATGCTTCTGATGCTCGATTCGAACGGGCAACTCCGTGAAGCGCCGGG
ATCGCTCGGTGAACGCGACGGACAGAAGGACTACTCGAAGAGCATCGACCTCGACTTTGACCCCCGACTCGAC
GCCGAGACGACCGTCCAGACCGACGACGGAAGCGTCCAAGTACGGTCTGTGTGGGCCGAACTGCGCGACGAAC
TCGCCCAGTACGACCCCGAGACGGTCACCAAGATGACCGGCGTCGGTACGGAGACGTACCAACGAGTCGCCCG
CGAGTTCGCCGATGTCGAACGCGCGAAAATCATTCACGGCAAGGGCGTCAACGACTGGTACCACAACGACCTC
GGGAACCGCGCCATCCAGTTGCTCGTCACGCTGACCGGCAACCTCGGCCGTCAAGGAACCGGGGTTGACCACT
ACGTCGGCCAAGAGAAAATTTGGACGTTCCACGGGTGGAAAACCCTCTCGTTCCCCACCGGGAAGGTTCGCGG
GGTGCCGACGACGCTGTGGACCTACTACCACGCCGGTATCCTCGACAACACGGACGCGGACACCGCGGCGAAG
ATACGCGAATCCATCGACAAGGGGTGGATGCCCGTCTATCCTGAGGAACGCGAAGACGGATCGCGGCCGGACC
CGACGACGATGTTCGTCTGGCGGGGGAACTACTTCAACCAGGCAAAGGGGAACGTCGCCGTCGAAGAGGAGTT
GTGGCCGAAACTCGACCTCGTGGTGGACATCAACTTCCGGATGGATTCGACGGCCCTCTATTCGGATATCGTC
CTGCCGACGGCCAGTCACTACGAGAAGCACGACCTGTCGATGACGGACATGCACACCTACGTGCATCCGTTCA
CGCCCGCTGTTGAACCGCTGGGCGAGTCGAAGACCGACTGGCAGATATTCAAGGACCTCGCCGAGAAGATTCA
GGAAGTCGCAACCGAGCGCGGCGTCGAACCGATTTCGGATCGGACGTTCGACCGCGAGATCGACCTCCAGTCA
GTGTACGACGACTACGTCCGCGACTGGGAGACGGAGACGGACGGCGCACTCGCGGAGGACCGCGCGGCCGCCG
AGTACATCCTCGAACACTCAGCGGAGTCGAATCCAGCGGGAACCGACGAACAGATAACGTTCGCCGATACGGT
CGAACAACCGCAGCGACTGCTCGAAGCGGGCGACCACTGGACTTCTGATATCGAAGACGGCGAGGCGTACGCG
CCATGGAAGGACTTCGTGCAGGACAAGAACCCGTGGCCAACCGTCACGGGCCGCCAGCAGTACTACATCGACC
ACGACTGGTTCCTCGAACTCGGCGAGCAGTTGCCGACGCACAAGGAGGGACCGACGAACACCGGCGGCGACTA
TCCGATGGAGTACAACACGCCGCACGGTCGATGGGCTATCCACTCGACGTGGCGCGACAGCGAGAAGATGCTC
CGCCTGCAACGCGGCGAACCCATCGTCTACATCAACCCCGACGATGCCGCCGAACGCGGCATCGAGGACGGCG
ACACCGTCGAGGTGTTCAACGACCTCGGTGCCGTCGAAGTACAGGCGAAGATATATCCCAGCAGCGAACGGGG
GACGCTTCGACACTTCTTCTCGTGGGAGAAGTTCCAGTACGCCAGTCGGAACAACTTCAACACGCTCGTCCCG
ATGTACATGAAACCGACACAGCTCGTCCAATACCCCGAAGACACGGGCGAACACCTGTACTTCTTCCCCAACT
ACTGGGGGCCAACGGGGGTCAACAGCGACGTCCGGGTGGACGTACGAAAGAAAGGAGGTGATGGCGGATGA
>vdi:Vdis_1441-Ar nitrate reductase subunit alpha; K00370 nitrate reductase 1, alpha subunit [EC:1.7.99.4] (N)
ATGCCTAAGGCCAACTTCATTAAGGAGGTCTTCAGGCACTTCCTGCCCAGAGAGAGGTTTAACAATGGATGGA
GTGAGTTGGATAATGCGGATAGGGCTTGGGAGGACCTGTACAGGAATCGCTGGCAGTATGATAAGGTGGTTAG
GTCGACACACGGCGTTAACTGCACAGGTTCATGTAGCTGGAGGGTCTATGTTAAGGATGGTATAGTGGTTTGG
GAGCATCAGGCGGTTGATTACCCAACCAATGGACCAAACATGCCTGAGTACGAGCCGAGGGGCTGCCCGAGGG
GTGCGTCATTCTCGTGGTACATATACAGCCCATTACGTATCAAGCATCCATACGTTAGGGGTGAGCTCATGAG
GCTATGGAGGGAGGCGATATCGAAGACAGGGGATCCCGTGAGGGCTTGGGAGTACATAGTCACAAACCCAGGG
TTAGCCAGTAGGTATAAGTCAGCTAGGGGTAAGGGCGGCTTTGTTAGGGCTTCCTGGGATGAGGTCCTCGAAT
TAATATCGGCGGCGCTGATATACACAATAAGGACCTACGGGCCAGATAGAATATTTGGCTTCACGCCAATACC
CGCAATGTCCATGGTAAGCTTCTCATCTGGGTCGAGATTCCTTGAGTTAATAGGTGGTGTGATGCTAAGCTTT

TATGATTGGTATGCGGACCTACCAATCGCGTCACCACAGGTATGGGGTGAGCAGACTGACGTGCATGAGAGTG
CTGATTGGTTCAACTCAATGTACATAATTGTCTGGGGCACGAACATAGCCATGACGAGGACCCCAGACGCCCA
CTTCCTAGCTGAGGCCAGGTATAGAGGTACGAAGGTGGTCGTTGTGACCACGGATTATACCGACGTTACAAAA
TTCGCAGACTTATGGATAGCGCCTAAGCCCGGCACTGATGCTGCCTTGGCTATGTCGATGATTCACGTCATAT
TGAGGGAGTTCTACGTGGATAGGCAGGTGGATTACTTCATAAACTACGTTAAGAGGTATACAGACTTACCGTT
CTTAGTAGTATTAGAGAGGAGTGGAGATTCCTACATACCGGGTAGGTTTCTGAGGGCTTCAGACTTAGGGCTG
AATGTACCAAATGCGGAGTGGAAGACCGTAGTCCTTGACTTAAATACTAAGCAGTTAGCTATCCCCAATGGTA
GTATTGGATTTAGGTGGAGTAATGAGGGTAGGTGGAACCTTAAGCTCGAGGACTCAATAACGGGTAAACCCAT
AGATCCTGCGTTAACATTATTGGGCATGCATGATGATATAGTATTGGTTCAGACCTACGTTTTTGATGATAAG
GGGCCAATACCAATCATCAGGGGAGTTCCCGTTAAGAGATTGAGTATTGGTAATAAGGACTTACTGGTCACGA
CTGTCTACGACCTACTGCTTGCCCATGTAGGTGTCAACAGGGGCTTACCTGGTGATTATCCATCAAGTTATGA
TGATCCTAAGCCATTTACGCCGGCTTGGCAGGAGATGATAACGGGTGTTGATAGGAAGGTTGCAATTAGGGTT
GCGAGGGAGTTCGCTAGGAATGCTGAGTTGACTGGGGGTAGGTCTATGGTCATCGTAGGCTCAGGCATTAACC
ATTGGTACCACTCAGACCTCATTTATAGAGCGATAATAACAATGCTTATGCTGCTCGGAACCATAGGTGTTAA
TGGAGGTGGCTGGGCGCACTACGTGGGTCAAGAGAAGGTTAGGCCCCTTGATTCCTGGTCCCACATAGCCTTC
GCCCAGGATTGGGTTAGACCACCAAGGCTCCAGAACACCACGTCATGGTCCTACATACACAGTGACCAATATA
GGTATGAGGACCTAAGCATGGGTAAGGTTGCCGTTAACCCCGAGTATGAGCATCCAGTGGACTATAACGTTAT
TGCGGTCAAGAATGGGTGGTTACCATTCTATCCACAGTTCAACATCAATCCAATAAGACTCTATGATGAAGCC
ACCAAGGCTGGGGCAAGGAGTGATGAGGAGGTTATTAAGTACGTAAAGGAAGGACTTACGAGGGGTAACATAA
GGTTCGCTGTCGAGGATCCCGACGCACCGGAAAACTTCCCAAGGGTATTATTCATGTGGAGGGCTAACTTCCT
CGCCTCGAGTGGTAAGGGTCATGAGTACGTACTCAAGCACCTACTAGGCACAGATAATTCGGTGATGGCTAAG
GAGGGATTGATAAAGCCTAAGCTCGTCAATTGGCGTGAACCCGCACCCATAGGAAAGCTTGACTTAGTGGTCA
CGATTGACTTCAGGATGAGCACATCAGCGCTATACTCAGACATAGTGCTACCTGCGGCGACCTGGTATGAGAA
GAATGACCTAAGCACCACGGACCTACATCCGTTTATACATCCATTTAACCAGGCCATACCACCACCCTGGGAG
GCTAAGGCTGATTGGGACATATTCGTGGCGTTAGCCAAGAAATTCAGTGAGATGGCGTGCAAATACCTAGGCA
AGGTCAAGGATGTGATTGCTGTACCGTTACTGCATGATACGCCTGGTGAACTGGGCAATAGGTCTGGCCCGAA
CATTGTTATTGTTGAGCGTGATTACTGCGATGTGTACCATAAGATGATCACACTTGGTCCCCTAATTAAGGAT
TCAATAGGTGCTAAGGGTATTACGTGGAGTGCCAGGGAGGAGTATGAGGGAGTCGGTAGACTTAATGGGATTG
ACGAGCACGGTCACCCACTATTGGTTAGGGATTATCAGGTTGCTGAGGCAATACTGCATTTATCTAGTACGAC
TAATGGCGAGGCATCGTATAAGGCATACAAGGCGCTTAGTGGTAGGGTTGGTATTGACTTAACGCATATTGTG
GAAAATCAAAGAGAGGTGGTGATACATTTCAGTGATCTATCCTACCAACCAAGGAGGGTGTTAAGGACACCTA
TTTGGAGCGGTAAGGAATCTGAGGAGAGGCCATATACGCCATTTGCATTAAATGTGGAGGACTTAGTGCCTTG
GAGGACGTTGACGGGTAGGCAGCACGTGTACCTGGATCATGAGTGGATCATAGAGTTTGGGGAGCAGCTACCG
ACGTATAAACCTCCCGTTGTCCAGGTACCGTTTAATAGCGGTGAGGGACCTAGCATTAGTGGTAAGTACTTGG
TTGCACGTTACTTGACGCCGCATGGTAAGTGGCAGATACACTCAACATACATGGATAATCAAATAATGCTAAC
GCTATTTAGAGGGGGTCCCGTTATTTGGATTAATAGCGATGATGCGAAGGAGGTGGGTATTAAGGATAATGAT
TGGGTCGAGGTGATTAATAGGAATGGTGTAATCGTGGCTAGGGCTGTCACGTCGATAAGAGTTCCGAGGGGTA
CCGTGATCATGTATCATGCCCAAGAGAGGACTGTGAATGTCGGTAGGTCCAAACTGACTAATAGGCCGGGTGG
TACCCATAACAGTGCCACTAGGGTTAGGGTTAAGCCGACGCACATGATCGGTGGTTATGCCCAATTAAGTTGG
GCGGTTAATTACTACGGCCCAGTCGGTACCCAGAGAGATGAGGTTGTGATAATTAGGAGGTTGGGGTGA
>pai:PAE3611-Ar nitrate reductase alpha subunit (NarG); K00370 nitrate reductase 1, alpha subunit [EC:1.7.99.4] (N)
ATGCTTAAAACAACTAGGCGGCGGATGTTAGCCGGCGTTGCTACTATAAGCGCGGCAGCTTGGGTAATGGCGT
TGGCCCAGAATTTGCAATATTTACAGCCGCTGGCGCAGTTTGTAAACACGAGGCTTCAATACCCTGACAGGTC
GTGGGAGGAGCTCTACCGCCGCCGCTGGCAGTACGACAAAGTGGCCAGGTCTACACACGGCGTTAATTGCACC
GGCTCTTGCTCGTGGAATGTGTACGTCAAGGACGGCCTTATAGTGTGGGAGCTACAGGCCACGGACTACCCGG
ACATCTCGCCGGACATACCCAATTACGAGCCCAGGGGATGCCCGAGGGGGGCCAGTTTTTCGTGGTACGTATA
CTCGCCCTTGAGGGTGAAATATCCCTACGTCAGAGGGGTACTCATAGACATGTACCGGAGGTTTAAACAAGAG
ACCGGCGATCCAGTGGAGGCGTGGCGCCGCATTGTGGAGGATCCCGCCAATCGCGCGGCCTATCAAAAGGCAA
GGGGGAAGGCCGGGTGGGCCAGGGTCACTTGGGACGAGGCCCTTGAGCTGATATCCGCGGCGTTGATATATAC
AATTAAAAAGTACGGCCCCGACAGAATCTACGGCTTCACCCCCATTCCCGCCATGTCCCCCGTGAGTTACGCC
TCAGGGGCGAGGTTTATTGAGCTAATCGGCGGGGCAATGGGATCGTTTTACGACTGGTACGCCGACCTTCCCC
CGGCCAGCCCTCAGATGTGGGGCGAGCAGACAGACGTCCCTGAAAGCGCCGACTGGTACCACGCCCAGTATAT
GATCGTGTGGGGGACTAATTTGCCAATGACTAGGACGCCCGACGCGCCTATGTACACACAGGCTAGATATAGG

GGGGCGAAGGTAGTTGTAGTCAGCCCGGATTACAGCGAGCACGTCAAATTTGCCGACTTATGGCTCCCGGCAT
TGCCCGGCACAGACGCCGCACTGGGCTTGGCCATGGCGCACGTGGCTTTAAAAGAGTTTTACGTGGACAAGCA
GTTCAAATGCCTGAGGGAATACGCGAGGAGGTATACCGACCTCCCCTTCCTTGTTATACTAGAGCCCGCTGGA
GACGGCACATACCTACAGGGGAGGTTCCTTCGGGCTTCAGACGTACCGGAGCTTAAGGATAAGGTGGGCGGAA
ACCCGGAGTGGAAAACAGTTGTATTGCAGAGCGACGGCACTTTGGCGGCTCCCTACGGCTCTATAGGCTTTAG
ATGGGATGGGAGCGGGAAGTGGAATTTAGAGCTGAAAGGAATTACGGCGAGGGGCGAGGGCGATATAGACCCT
GTGCTTTCTGTAATGGAGGTGGGGAAGTATGAAAAAGTGCCGGTGAAGTTCTTCGCCATGGACTTCGACGTGA
AGAGTACGGTGAGGGAAGTCCCCGCGATTAAAATTGGCGATAAATACGTCACTACTGTATTCGACTTATTGGC
GGCGCATTTAGGAGTAAAGAGAGGCGACTTGGGAGGCGATTACCCAGCTGATTACAACGACCCCAAGCCCTTC
ACTCCCGCGTGGCAGGAGTCTATAACAAATATCTCGCGCGACCTAGCCATAAGAGTTGCCAGGGAGTTTATGA
GCACTGCCATTTACTCTAAGCCTGTTTACGGCAAGCCGGGCGATCCCCCGTTCTTTGGAGACGACGCCACGCC
TTGTGGGGGCAGAGCTATGATATTCGTAGGTCCTGGGATTAACCACTGGTATCACGCAGATTTAATATACCGC
TCAGTCTTATTATTAGTACTGCTGGGCGCCACCCAGGGCAAGAACGGCGGCGGCTGGGCCCATTACGTGGGGC
AGGAGAAAATAAGGACTCTCATCGGGTGGTCCACAATAGCCTTTGCGCTTGACTGGATAAGGCCGCCGAGGCT
ACAAAACTCCCCCAGCTACTGGTACGTCCACACCGACCAATGGCGTTACGACCCGGTCACCACTAAATTATAC
GCCGCCACCTGGGCGAAGAAGTGGGGCGATATTCACGAAATGGACGCCAATGTAATTGCCGTGAGGCTTGGCT
GGTTGCCCTTCTACCCGTCGCTTAATGTAAACCCGCTTGAGCTAGGCGACAAGCTAGTGAGCGAGGCTAAGGC
TCAAGGCGTCCCCGACGCGCAATTAGGCCAGTACGTGGCGAGGAGAGTGGCGCAGATGTTGAAAAACGGCGAG
ATTAAGCTGGCAATAGAAGACCCCGACGCTCCTGAGAATTGGCCAAGGGTGTTGTTCGTCTGGAGGGCAAATC
TGCTGGGCTCCTCCTCTAAGGGCCATGAATACTTCCTTAAACACTTGCTGGGCACTGACAACAACGTAATGAA
TAGGGAATACGCTGTGGAGGAGGGATTAGTTAAAGAGGTGAATATTAGAAAGCCGGCGCCTGAGGGCAAGCTG
GACTTATTAGTTGTGTTAGACTTCAGAATGGCAACGTCAGCGGTCTATGCGGACGTAGTACTCCCCGCGGCGA
CGTGGTATGAGAAATACGACTTATCCATGACAGATCTGCACACGTTTATACATCCCTTTACGCCCGCTGTGGA
TCCGCCGTGGGAGGCAAAGGCCGATTGGGATATATTCAAGGAGCTCGCAAAGAAGTTCTCTGAGCTGGCTAGG
AAATATCTGCCGCAAGAGGCGTATGACGTAGTGCTAACGGCTCTTCTCCACGACACGGCTCAAGAAATCGCCC
AGCCGTTTGGCGAAGTGAAGGACTGGAAAAAGGGCGAGGTGGATCCCGTCCCCGGCAAGACTATGGCCGCTGT
GTCTCTAGTGAGGAGGAGGTATTGGGACGTATACGACATGTACATCACGCTGGGGCCGCTCGTAGTGACGATG
GGTCTATCGACAAAGGGCATACCCGCGTTTAAACCCGTGGAGAGCTATAACTGGCTGAGGGAGATAAACGGCT
TGACAAGCGCTACGTCGCAATTCGTACAAGCGCAGTGCGCCAACGTAGGGGGATGCCCGAGCATTGAGAGGGA
TAAACAGGCGGCTGAGGCCATGCTGGCCATATCTCCTGAGTCAAACGGCGAGGTGGCCTATATGGCTTGGAGT
AATTTGGAAAAAGTAGTGGGTCTTCCGTTAAAAGACCTCCCCGCTAAAGAGCACAGAATTATGTTTAACGATA
TCGTCGCCCAGCCGAGACGCGTGACTACCTCGCCTGTGTGGAACGGAATAGAGGCTCCCGGGAGGACTTACTC
GCCATTTACTGTAAACACAGAGAGGAAAGTGCCCTGGCGCACTCTGACAGGCCGCCAGCACTTCTACATCGAC
CACGAGTGGTTTAGAGAGCTGGGCGAGTCGCTTCCAGTATACAAGCGCCCGCTCGACCAAGTAATGGCCAAGT
TAATTGCTGACGTTAATCTATCCGACTACTGGTACGATCCCGCCAAATATAGAGTGGAAACTCCCGGCGGCAA
ATACCTAGTCCTCCGCTATTTAACTCCACACGGCAAGTGGAATATACACTCTACGTATTGGGACAACCTAATT
ATGTTGACGTTATTCCGCGGCGGGCAGGTGGTGTGGATTAACGACGAAGATGCTAAGTGGCTCGGCGTTAAAG
ATAACGACTGGATTGAGGTGTATAACGCCAACGGCGTTATAGTGGCCAGAGTGGCCGTCAGTCCGAGAATTCC
GCGGGGCACAGCCATTATGTACCACGCCCAAGAGAGGCATGTGTACGTCCCCATAAGCCCCAAGACTGGCAAG
AGGGCTGGAATTCACAACAGCGTCACTATAACTCACCTCAAGCCGACAAAAATGGTGGGCGGCTATGCGCAGT
TGAGCTGGTCCTTTAATTACTACGGCCCCACTGGCGTAAATAGAGACACTCTAGTAGTAGTAAGACCCGCCGG
CAGGGTGAGGCTATGA
>fpl:Ferp_0311-Ar nitrate reductase, subunit alpha (EC:1.7.99.4); K00370 nitrate reductase 1, alpha subunit [EC:1.7.99.4] (N)
ATGGCTGAAAGGATAAAAGATGTGTATTCTCCAGAGTTGAGAAGCTGGGAAGAGTTTTACCGAAACAGATGGC
AGTACGATAAAGTTGTTAGGAGCACTCACGGAGCTAATTGCACGGGAAGCTGCAGCTGGTTTGTTTACGTAAA
AGACGGAATAGTTACTTGGGAACTCCAAGCGACCGACTATCCGAGTTTCAACCCAGATGTACCTAACTACGAA
CCAAGAGGGTGTCAGAGGGGAATTAGTGCATCTTGGTATCTCTACAGTCCGTTACGCCCGAAGTATCCTTATG
TAAGGGGAGTTCTCCTCGATCTCTGGAGAGAAGCAAAGGAGAAGCACAAAGACCCGGTGGAAGCTTGGAAGAG
CATCGTCGAAAATCCGGAGTTGAGGAACAAGTACGTCAGAAAGAGAGGAATGGGTGGATTCAGAAGATCGAGC
TGGGATGAGGTAGTTGAGATAATAGCCGCTGCAACGATTTACACAGTTAAAAAGTACGGCCCAGACAGAGTTA
TAGGATTCACGCCGATTCCAGCGATGTCGATGATAAGCTACGCCTCTGGAGCAAGATTCCTCCAGCTTTTAGG
AGGAGTTTGTCTGAGCTTTTACGACTGGTATTGCGATTTACCTCCAGCCTCTCCCCAAATATGGGGTGAGCAA
ACGGATGTAAACGAGTCGGCAGATTGGTATAATGCCGGATTCATCGCAATCATGGGTTCGAACTTAAACATGA

CCAGAACTCCAGACACCCATTACTTATCGGAAGCCAAGTATAGAGGAGCTAAAGTCGTCGTTTTCTCTCCAGA
CTATAGCCAAGTTAGCAAGTTTGCTGATGTATGGATACCAATAGAGCAAGGACACGACGCAGCCTTCTGGATG
GCAGCCAACCACGTCATAATGAAGGAGTTCTACTTCGATAAGGAAACTCCCTACTTCATCGAATACGTGAAAA
AGTACACCGATCTACCTTTCTTAGTCAAGTTAAAGCAAGAAGGGGATGTTTTCGTCTTTGACAGATTTTTGAG
GGCATCCGAAATTGAAAGAGGAAAAGATCTGAAAAATGCGGAGTGGATTCTGTGCGTTATGGACGAGAAGGGA
GAGATTAGGATTCCGAACGGAAGCATTGGATTCAGGTGGGGAGAAGAAGGAAAGTGGAATCTTGAGATGAGAG
ATGCCGTTGACGGAGAAGAAATTGACGTCAAACTCAGCCTCTTCGGAGAAGATTACGTGATGGTGAAGTTTTA
TTACGAAGACTACGGAGAGAGCATACGAGCCGTTCCAGCGAAGAAAATTAAGACAGCCAATGGAGAAGTCGTA
GTCGCTACCGTCTTTGACCTCCTCGCAGCACACCTCGGAATTTCCAGAGGATTGCCAGGGGATTATCCTAAAG
ACTATGACGACGACAAACCTTTCACTCCAGCTTGGCAGGAGAAGTACACGAAGATAAGCAGAAAGACGGTCAT
AAAGATCGCAAGAGAGTGGGCTGAGAACGGAGAGAAAACTAAGGGAAGAAACCTCGTGATAATCGGTCCCGGA
TTGAACCACTGGTACCACAACGATTTAAATTACAGAGCCGTTATCACGGCGTTGATATTAACTGGAAGCGTCG
GAAGAAACGGAGGAGGGCTTGCTCATTACGTTGGACAAGAAAAGGTAGCTCCTATTGCCTCTTGGGCTGTTGT
TGCCTTCGCACAGGACTGGGTTAAGCCACCAAGACTGCAAAACTCACCAAGCTTCTACTACGTGCACACGGAT
CAGTGGCGATACGATGGATGTAAGTATATAGACAAGGTCGATGGACTGCCGAAGCACACAATAGACTTCAACG
TGAAAGCTGTTAGGCTTGGCTGGTTACCCTTCTATCCTCAATTCAACGAAAATCCTTTGAAGCTCGTGGAAGA
GGCTCAAAAATCGGGAGCTAAGAGTGACGAAGAGATCAGGAGATGGATAGTCAATAAACTGAGGGAAGGAAGT
TTGAAGTTCGCAATAGAGGATCCGGACGCTGAAGAGAACTGGCCGAGAGTATGGTTCATATGGAGAGGGAATG
CCATAGGATCGAGCCCCAAGGGTCACGAGTTCTTCCTAAAGCACTTCGTTGGAGCTCCAAACAGCAACGCAAT
GGCTAAAGAGGTCGCCAAGGATCTGGTTGAGGAGGTTGTTTGGAGAGAAGCTCCGGAAGGTAAGCTGGACTTA
ATCGTTGATTTGAACTTCCGAATGGACACAACTGCCGTTTACTCGGACATCGTTTTGCCAGCTGCCCACTGGT
ACGAAAAGAACGACATTCAATCCACAGATTTGCACACATTCATTAACTGTCTTGGAGCTGCAACATCTCCAGC
TTGGGAGGCAAAATCCGACTGGGAGATTTTCAAGCTCATCGCAAAGAAATTCAGTGAGCTTGCTGAGAAGCAC
TTCCCGAAGCCGGTTAAAGATGTTGTAATGGCACCTCTCCTCCACGACACTCCGGAAGAGATAACCCAGCCGG
AGGTCAAGGACTGGAAGAAAGGAGAGACGGAGCCAATTCCTGGAAAGACCATGCCCAAACTCGTAATAGTCGA
AAGGGACTACGCCAACCTGTACAAGCGATTCATATCCTTAGGACCGGGAATTAAAACTCTCGGAGCTCACGGA
GTTAGCTGGGATGCAAGCGACATTTACGAGGAGCTTCTTGAGAAGTATCCCACGGCGAAGTGGAACGGAAACA
AGTACATTTCCCTTGAAGACGAGGTGACTGTTGCCAACGTAATTCTGACTTTTGCTCCCGAAACGAACGGAGA
GATTAGCTATCGAGGATTCAAGAATTTGGAGAAGAAGGTCGGTAAAAAGCTTAGTGAAATTGTCGAACCCTAC
AGAAGCTACAGAGTTACTTTCGAAGATGTCGTGACGCAGCCGAGAAGAATTATCACAAGCCCCGTCTGGAGTG
GAAACATCAACGATGGAAGAACATATTCCCCCTACACCCTCAACGTCGAATTCGAAATACCTTGGAGGACTCT
AAGCGGAAGGCAGGAGCTCTACCTTGACCATCCCTACTACATAGCCTTCCACGAAGCTTTGCCAACATTCAAG
CCCAAGCTCAGCCCCAAGCTTCTTGACGAGCTTGAAGGAGAAGAAGGACTGATTCTAAACTTTATAACACCTC
ACGGGAAGTGGAGCATTCACTCAACCTACTACGACAATCTGAGAATGCTAACTTTGTCGAGAGGAGGGCAGGT
AGTCTGGATAAACGACGAAGATGCGAGGAATGCTGGAATAAACGATAACGACTGGGTAGAAGTTTATAACAAC
AACGGAGTCATAGTTTGCAGAGCTGTCGTTTCTTCAAGAGTGCCGAAAGGAATGGCAATAATGTACCACGCTC
CAGAGAGAACTCTCTACATTCCGAAATCGAAGAAAACTGGAAGGAGAGGAGGAGTTCATAACAGCGTGACGAG
AACGAGGCTCAAACCGCTTCTCATGGTTGGAGGGTATGCGCAGTTCAGCTACTACTTCAACTACTGGGGACCG
ACGGGAGTGAACAGAGACACTTACGTTATTGTTAGGAACGTTATTGTTAGGAAGCTTGAGAGGTGA
>sia:M1425_0828-Ar nitrate reductase, subunit alpha; K00370 nitrate reductase 1, alpha subunit [EC:1.7.99.4] (N)
ATGACATGGCAAAAATTTGAAAATAAAGGACGTGAATGGGAAGAGTTTTACAGGCAAAGATGGGCTTATGCAA
AGGCAATTAGATCTACACATGGCGTTAACTGTACGGGGTCTTGCAGTTGGCTGATATATGTAAAAGATGGTAT
TATAATTACAGAAATGCAAGCACTAGATTATCCTATTATAAATGAGGAAATACCACCTTATGAACCTAGAGGA
TGTCCTAGAGGAGCCAGCTTTTCCTGGTATGAATATGCACCTCATAGAATAAAACATCCTTACATTCGTAAGC
AACTACTTAAGTTATGGAGAGAAGAATTAGAGAAGACAAAAGATCCTATCAAAGCTTGGGAAAATATAGTCGA
AGATCCGGAAAAGGTCAAGCTCTATAAGGAGAATAGAGGTAAAGGCGGATTCGTTAGAATTAGTTGGGATGAA
GTCGAGGAGTTAGTTTCTGCTGCGTTAATATATACGATTAAAAAATACGGACCAGATAGGATTGCTGGTTTTA
CACCAATACCTGCCATGTCAATGGTTAGTTATGCTGCAGGAACTAGGTTTTTATCATTAATAGGAGGAGTAAT
AATGAGTTTCTATGACTGGTATGCGGATTTACCTATAGCTTCTCCTCAAGTATGGGGCGAACAAACTGATGTT
CCAGAAAGTGCTGATTGGTTTAACTCTACCTATATAATAGATTGGGGAACCAACATTCCTCAAACAAGAACAC
CAGATGCTCATTTTCTCTCCGAAGTCAGATATAGAGGAACTAAGGTAGTTGCGATTTCTCCGGATTATGCGGA
ATATGTAAAATTTGCAGACTTATGGTTACATCCTAAACCTGGTACTGACGGAGCACTAGCTTTAGCTATGGCG
CATGTAATAGCTAAAGAGTTCCATGTTGATAGGCAAGCGAAGTACTTTACAGAATATTTAAAGAAGTATACCG

ATGCTCCATTTTTAGTAATCTTGGAACAACAAAATGGAAGAGTTATATCTGGAAGATTTCTTAGAGCATCTGA
TATTATTGAAAACGTAATAAATGGAGAATGGAAGTTAGTAGTATATGATACATTAAAGGGGAGATTCGTAATT
CCAAATGGATCTATAGGGTTCAGATGGTCAGACGAAAAGAAATGGAATCTAAAACTACAAGATAGCTTAACGG
GAGAAAATATAGAACCAGCTTTAACATTTCTAGGGATAGAAGATCAAAGGATGATGATATCTCTCACTAGACT
TGATGAGAAAGGGTTTATAGAAAGGGAAATCCCTGTCAAACTAGTAAAATTAGCTAATGGTAAACAAATTTAC
GTTACAACAGTATTTGATTTATTATTAGCCAATTTAGGTGTTAGAAGAGGAGATCTGAAAGGGTATCCAACTA
GTTATGACGATGATAAGCCTTATACTCCTGCTTGGCAAGAGAAAATAACGGGAGTCCCAAGGGATCTAGTAAT
ACAAGTAGCTAGAGAATTCGCCAAGAATGCGGAGGATACTAACGGTAAATCCATGATATTAGTTGGAGCTGGC
GTTAATCACTGGTTTAACAGTGACCTAATCTATAGGGCTATAATAACTATTTTAATGTTAATAGGTGCGATTG
GAGTTAATGGCGGGGGATGGGCACATTATGTTGGTCAAGAAAAAGTTAGACCTTTTGAGGGGTGGCAAACAAT
TGCTTTTGCAAGAGATTGGATGCTTGCTACAAGATTTCAAAACACTACTACTTGGATATACATTCATTCTGAT
CAATGGAAATATGATAGCTTAACATTAGATAAATTAACTCCTAATAACACATTGTATAAACATCCAGCAGATT
ACATAGTGTTAGCAGTAAAAAGAGGTTGGACGCCCTTCTATCCACAATTTGATAAAAATCCTCTAAATCTCTC
TCCAGAAAATGTTATCGATAAGATTTCATCCGGTGAAGTGAAGTTTGCAATAACTGACCCAGATAATCCAATT
AATTTCCCGAGAATATTTTTCATTTGGAGAGCAAATTTACTGTTTTCTAGTGGAAAAGGAAGCGAATACATTC
TAAAGCACTTATTAGGTACTCATAACAGTGTGAATAATACTGAAGATGCTAAAGATAAGGTGACTGAAATTAC
TTGGAGAGAACCTACACCAATAGGAAAGCTTGACCTAATAGTCGATCTTAACTTTAGAATGGATAGTAGTGCG
TTATATTCAGATATCGTGCTTCCAGCTGCAACATGGTATGAAAAATATGATATAAGTTCTACTGATATGCATA
CTTTTGTTCATCCATTTCAACCAGCTACAGATCCTTCTTGGGAAGCTAGGAATGATTGGGATATTTTCGTAAA
TTTAGCTAAGAAATTCTCAGAAATGGCATCGAGATATCTTAATGGAAAGCATAAGGATGTCGTCTATTTACCT
TTATCCCACGATACCCCAGATGAAATCTCACAATCTTTAATTGAGGAGGAATTAGATCCTCCTTATATTTCAG
CTCATCTTGCAAGTAAGATAATCCCAAAGAGGAACTTCGGGAATCTTATAGTCATTGAAAGAGATTATACTAA
AGTTTACGAGATGATGATTTCATTGGGGCCTCTGATAAGAGATAAGGAATTACTTTATGTCGGCATACCCGTT
AATTATCGTAAGGAATACGAAGAATTGAAAGAAGAATTAGGAGAGATAGATGATAGACCAGATATTAAGGAAG
CTAAGAAAGTGGCTGAAGTAATGCTTAGATTAAGCGGGGCTACTAACGGTGAGGTTTCAGTTAAAGAATATAA
ACAACTTGAAGTGAAAACGAGTCTTAACTTTAGCGATCTATATGAAGGAATATCCGAAATAAAGATTAAGTTT
GACGATATAATAGCTTCACCTAAAAGAGTTATAGACTCACCTATCGAGTCCGGAATAGTGAAAGGAGAAAGGA
CTTATAATTCGTTCACGTTTAATATAGAGTATGAAGTACCTTGGAGGACATTATCTGGAAGGCAACATTTTTA
CTTAGATCATCCGATTATAAGGGAATTAGGAGAACAGCTACCAATCTATGCACCACCTTTAGAGTATAATAAA
GTAATTAAAGATAAGGATACCCTAGTTCTGAGGTATCTAACTCCACATGGAAAATGGCAAATTCATAGTACTT
TTATGGATAATTTAAGAATGTTAACACTATTCAGAGGTGGTCCGATTATTTGGTTAAATGAAGAAGATGCAAG
AAGCGTGGGGATAAAAGATAATGAGTGGGTTGAAGTGTTTAACGAAAATGGAGTGACTGTAGTTAGAGCAGTA
GTTAGTAATAGAATACCCAGAGGTACAGCAATAATGTATCATGCTCAAGAACGTACAGTCTATATAAATGAGT
CTAGATTAAGGGGAATCATTGGAGGTTCCCATAATGCGGTTACTATGGTTAACATAAAACCAACGTGGTTAAT
CGGTGGATATGCACAACTCTCCTATGCTCTTAATTATTATGGAGCAGTTGGTACTCAAAGAGATACAATAGTA
GCTGTAAGGAGGTGGAGAACATGA
>pas:Pars_0499-Ar nitrate reductase, alpha subunit; K00370 nitrate reductase 1, alpha subunit [EC:1.7.99.4] (N)
ATGCAAATCACTCGTAGACAAGTTCTTGCCGCTGCTGCCACAATTGGCGCCGCTTCAGGCGTGTTGGCGTTGG
CGCAGAATCTCCAGTATTTGAAGCCGCTGGCTGAGTTCCGTGAGACGAGGCTCCAGTACCCAGATCGTAGCTG
GGAGGAGGTGTACCGCCGGAGATGGCAGTACGACAAGGTGGCGAGATCTACCCACGGCGTCAACTGCACAGGA
AGTTGCTCGTGGAACGTCTATGTGAAGGACGGGCTTATCGTCTGGGAGTTGCAGGCCACAGACTATCCCGACA
TCGCGCCAGATATACCCAACTACGAGCCTAGGGGTTGCCCCAGGGGAGCCTCCTTTTCTTGGTATGTCTACTC
CCCGCTGAGGGTTAAGTACCCATACGTACGCGGCGTGTTGATAGACATGTACCGCCGCTTTAAACAGCAGACG
GGAGATCCAGTGGAGGCGTGGCGCCGCATTGTGGAGGATCCCGCAAACAGAGCGGCCTATCAAAAGGCGAGGG
GGAAGGCCGGCTGGGCGAGGGTGACTTGGGACGAGGCGCTTGAGCTGGTCTCAGCCGCGTTGATATACACAAT
CAAGAAGTACGGCCCCGACAGGATATACGGCTTTACCCCAATTCCCGCCATGAGCCCAGTCTCGTACGCAGCC
GGCGCCCGCTTTATAGAGCTAATAGGCGGTGTCATGGGCTCGTTCTACGACTGGTACGCAGACCTGCCGCCGG
CCAGCCCCCAGATGTGGGGCGAACAGACAGACGTCCCTGAGTCGGCCGACTGGTTCCACGCCCAGTACATGAT
TGTGTGGGGGACCAACCTCCCCTTGACCCGCACCCCCGACGCCCAGATGTACACCCAGGCCAGATACAGGGGG
GCCAAGGTGGCTGTGGTAAGCCCCGACTATAGTGAACACGTGAAATTTGCCGACATATGGGTCCCGGCGCTCC
CAGGCACAGACGCGGCGCTGGGTATGGCAATGGGCCACGTGGCGCTTAAGGAGTTCTACGTGGATAGGCAGTT
CAAATGTCTGAGGGAATACGCAAGGAGGTATACCGATCTTCCCTTCTTAGTCATCCTCGAGCCCGCTGGCGAC
GGGACGTACCTCCAGGGGAGGTTCTTGAGGGCAAGCGATGTGCCTGAGCTTAAGGACAAGGTGGGGGCCAACC

CCGAGTGGAAGACTGTGGTCGTCCAGAAGGACGGCACACTCGCCGTGCCATACGGCTCCATCGGCTTTAGGTG
GGACGGTAGCGGGAATTGGAATCTCCAGCTGAAGGGCATAACTGCAAAGGGCGAGGGGGACATAGACCCCGTG
CTCTCCATCATGGAACTAGGCCGCTATGAGAAAGTTCCGGTGAAGTTCTTCGCAATGGACTTCGACGTCAAGG
CCTTTGTGCGGGAGGTCCCGGCGGTAAAGATAGGCGATAAATACATCACGACCGTATTCGACCTATTGGCGGC
ACACCTAGGCGTCAAGAGAGGCGATCTCGGAGGCGACTACCCCGCCGACTACAACGACCCCAAGCCCTATACC
CCGGCGTGGCAGGAGGCGATAACCGGCGTGTCCCGCGATCTCGCCATACAAGTGGCCAGGGAGTTTATGAACA
CTGCCGTTTACTCAAAGCCGGTATTTGGGAAGCCTTCAGATCCGCCGTTCTTCGGCGACGACGCAACGCCCTG
CGGCGGGTGGGCGATGATATTCGTGGGGCCTGGCATAAACCACTGGTACCACACCGACTTGATATACCGTTCT
GTGCTACTGCTGGTATTGCTGGGAGGGACGCAGGGAAAGAACGGCGGCGGCTGGGCGCACTACGTGGGCCAAG
AAAAGATAAGGACGCTTATCGGGTGGTCTACCATAGCCTTTGCCCTCGACTGGGTGAGACCACCTAGGCAACA
AAACTCTCCTAGCTATTGGTATGTCCACACCGACCAGTGGCGCTACGACCCGGTTACCACAAAGTATTACGCC
GCGCCTTGGGCCAAGAAGTGGGGCAACCTACACGAGATGGACGCAAACGTAATTGCGGTGAGGCTCGGCTGGC
TTCCCTTCTACCCGTCGCTTAACGTAAACCCGCTTGAGCTGGGCGATAAGCTGGTGAAGGAGACAACTGCCCA
AGGCATTCAGCAGGCGCAACTGGGCGCCTACGTGGCGAGGAGGGTGGCGGAGATGTTGAAAAACGGCGAGATA
AAGCTGGCTATCGAGGATCCCGACGCGCCGGAGAACTGGCCAAGGGTGCTCTTTGTGTGGAGGGCGAACCTCC
TCGGCTCCTCGTCTAAAGGCCACGAGTACTTCCTAAAACACCTCTTAGGCACGGACAACAACGTAATGAATAG
GGAGGTGGCCGTGGAGGAGGGCCTAGTCAAGGAGGTAAACATACGCAAGCCCGCCCCTGAGGGGAAGCTCGAC
CTCTTAGTAGTGCTGGACTTCAGAATGGCCACGTCAGCCGTTTACGCAGACGTTGTGCTACCCGCGGCGACGT
GGTACGAGAAGTACGATCTCAGCATGACAGACATGCACACATTCATACACCCGTTCACCCCGGCCGTCGACCC
GCCGTGGGAGGCCAAGGCCGACTGGGACATCTTCAGAGAGCTGGCAAAGAAGTTCTCAGAGATGGCGCGCAAG
TACCTACCGCAGGAGGCATACGACGTGGTGTTAACCGCTTTGATGCACGACTCAGCCGCGGAGATCGCCCAGC
CCTTCGGCGAGGTGAAGGACTGGAAGAAGGGCGAGGCGGATCCAGTGCCCGGCAAGACCATGGCCGCGGTGGC
GCTTGTAAGGAGGAGGTACTGGGACGTCTACGACATGTACATCACCCTAGGCCCGCTTGTAGTAACCACAGGC
GTCACGGCTAAGGGCATACCGGCGTTTAAGCCGGTTGAGAGCTACAAGTGGCTTGTGGAGAAAAACGGCGCGA
CGAAGGCCGCCTCCGATTACGTGCTCAGCCAATGCGCAAACGTGGGCGGATGCCCCAGCATTGAGCGGGATAA
GCAGGCGGCAGAGGCCATGTTGGCGATATCCCCTGAGTCTAACGGCGAGGTGGCCTACATGGCTTGGAGTAAT
CTGGAAAAGGTCGTGGGGTTGCCGCTAAAGGATCTCCCAGCAAAGGAGCATAGGATTGCGTTTAACGACATTG
TGGCTCAGCCTAGGAGGGTGACTACCTCTCCGGTGTGGAACGGCATTGAGGCGCCTGGCAGGACCTATTCGCC
GTTTACCGTAAACACAGAGCGCCTCGTCCCGTGGCGCACCCTCACAGGTAGGCAGCACTTCTACATTGACCAC
GAGTGGTTTAGGGAGCTGGGCGAGGCGTTGCCGACGTATAAGAGGCCTATGGACAGGATCATGGCGGGACTGC
TAGCCGGCGTTAATCTCGGCGACTGGTGGTACGACGAGAAGAAGTACAGGGTGGAGTCAGGCGGCCAGAAGTT
CCTAGTACTGCGCTACCTCACTCCACACGGCAAGTGGAATATCCACTCCGAGTTTTGGGATAACTTAATTATG
CTCACGCTGTTCCGCGGCGGCCAGGTTGTGTGGATCAACAACGAGGACGCCACTTGGCTGGGCATTAAGGACA
ACGACTGGATTGAGGTATATAACGCCAATGGCGTAATTGTGGCGAGGGCAGCCGTCAGCCCTAGGATTCCCAG
GGGGACGGCCATCATGTATCACGCCCAGGAGAGGCACGTATATGTCCCCATATCGCCGCTTACAGGTAAGAGG
GCCGGCATCCACAACAGCGTCACGGTGACGCATCTAAAGCCGACTAAGATGGTGGGCGGCTACGCCCAGCTGA
GCTGGTCCTTTAACTACTACGGCCCTACCGGCGTGAACAGAGATACGCTAGTAGTAGTGAGGCCTGCTGGGAG
GGTGAGGCTATGA
>pcl:Pcal_1907-Ar nitrate reductase, alpha subunit; K00370 nitrate
reductase 1, alpha subunit [EC:1.7.99.4] (N)
TTGTTGGCGCTGGCTCAGAACCTTCAGTATCTAAAGCCCCTGGCGCAGTTTGTAGACGCCCGCCTCCAGTACC
CCGACAGGTCGTGGGAGGATATGTACAGGAGGAGGTGGCAGTACGACAAGGTTGCCCGCTCTACCCACGGCGT
GAACTGTACGGGGTCTTGTTCGTGGAATGTCTACGTCAAAGACGGCTTGATTGTGTGGGAGCTTCAGGCCACG
GACTACCCCGACATATCTCCCGACATTCCCAACTACGAGCCTAGGGGGTGCCCAAGGGGTGCGAGCTTTTCCT
GGTACGTCTACTCGCCGCTTAGGGTGAAGTACCCCTACGTGAGGGGGGTGTTGATGGACATGTATAGGAGGTT
TAAGCAGGAGACTGGGGACCCTGTGGAGGCTTGGAGGAGGATTGTGGAAGATCCCAACAACCGCCGGGCGTAT
CAGCGGGCGAGGGGCAAGGCGGGCTGGCGCCGGGTGAGCTGGGACGAGGCGCTGGAGCTAATTGCCGCCGCCT
TGATATATACAATTAAGAAGTATGGGCCTGACAGGATCTACGGCTTTACCCCAATCCCGGCGATGTCGCCTGT
GAGCTACGCCGCGGGCGCCAGATTTATAGAGCTCATCGGCGGCGTCATGGGCTCCTTTTACGACTGGTATGCA
GACCTCCCGCCGGCCAGCCCCCAGATGTGGGGCGAGCAAACCGATGTGCCAGAGAGCGCAGACTGGTTCCACG
CCCAGTACATGATTGTGTGGGGGACGAACCTCCCCATGACTCGTACCCCCGACGCACAGATGTACACCCAGGC
CAGGTACAGGGGGGCTAAGGTGGCCGTGGTAAGCCCAGACTACAGCGAACATGTGAAATTTGCAGATGTGTGG
GTGCCGGTGGCCCCTGGCACAGACGCCGCGTTTGGCCTAGCGGCGGCCCACGTGGCGCTTAAGGAGTACTACG
TGGACAAGCAGATTAAGTGTCTGAGGGAGTACGCGAGGCGGTACACCGATTTGCCGTTCTTAGTCGTCCTCGA

GCCGCTGGGCGATGGGACCTACGCCTATGGCCGCTTCTTAAGGGCCTCAGACGTGCCAGAGCTCAAGGACAAG
GTTGGCCAAAACCCCGAGTGGAAGACTGTGGTGGTGCAGAGTGACGGATCGCTGGCGGTGCCCTACGGCTCTA
TTGGGTTTAGGTGGGATGGTAGCGGCAAGTGGAATCTCCAGCTGAAGGGGATAACGGCGAGGGGCGAGGGGGA
CATCGACCCTGTGCTCTCGGTGATGGAGCTTGGGAAGTATGAAAAGGTGAAGGTCAAGTTCTTCGCCGTCGAC
TTTGACGCAAAGGAGTTTGTCAGAGAGGTCCCGGCCATTAAAGTGGGCGATAGGTACGTCACCACGGTCTTCG
ACCTCTTGGCGGCCCATCTGGGGGTGAGGCGGGGCGACCTCGGCGGCGACTACCCCGCCAGCTACGACGACCC
CAAGCCTTTCACGCCGGCGTGGCAGGAGGCCATCACAGGCGTGTCTAGGCAGCTGGCCATACAAGTGGCGAGG
GACTTCATGAACACCGCAATTTACTCGAGGCCGGTCTTCGGCAAGCCGAGCGACCCGCCCTTCTTCGGCGACG
ACGCCACGCCTTGCGGCGGGAGGGCCATGATCTTTGTGGGGCCGGGGATAAACCACTGGTACCACGCAGACAT
CATTTACCGCTCTGTGCTGTTGCTGGTGTTCCTCGGCGCAACGCAGGGCAAAAACGGCGGCGGGTGGGCCCAC
TATGTGGGACAGGAGAAGATTAGGACTTTGATCGGCTGGTCTACGCTGGCCTTTGCCCTGGACTGGGTCAGGC
CGCCTAGGCAACAGAACTCTCCCAGCTACTGGTACGTGCACACTGACCAGTGGCGCTACGACCCAATAACCAC
TGACAAGATTTACCCAGCGCCGTGGGCTAAGAAGTGGCGCAATATGCATGAGATGGACGCCAACGTAGTCGCG
GTGAGGCTGGGCTGGTTGCCGTTTTACCCAACTCTCAACGTCAACCCCATTGAGCTTGGGAGCAGACTGGTGC
AAGAGGCGAGGCAGGCGGGGGTACCCGACGCGCAACTGTCGGCCTACGTGGCTAGGAGGGTAGCCGAGATGCT
TAAAAGCGGCGAGGTCAAATTCGCAATAGAGGACCCAGACGCGCCGGAGAATTGGCCCAGGGTGCTGTTCGTC
TGGAGGGCCAACCTCATGGGCTCCTCGTCCAAGGGGCACGAGTACTTCCTCAAGCACCTACTTGGCACAGACA
ACAACGTAACGGCTGAGGAAGTGGGGACAAAGCTTGTGAAAGAGGTAAACGTGAGGCCAGCTCCCGAGGGCAA
GCTAGACCTCCTTGTGGCAATAGACTTCCGCATGGCCACGACCCCCGTGTACGCAGATGTGGTTCTGCCGACG
GCCACGTGGTATGAGAAGTACGACTTGTCTATGACAGACATGCACACCTTTATCCACCCGTTTACCCCCGCCG
TGGATCCGCCGTGGGAGGCCAAGAGCGATTGGGAGATCTTCAAGGCGTTGGCTAAGAAGTTCTCTGAGATGGC
GCCTAAGTACCTCCCGCAGGAGGCCTTTGACGTAGTTATGACGCCGTTGATGCACGACTCGGCGGCGGAAATT
GCACAACCATACGGCGAAGTGAAGGACTGGAAGAAGGGCGAGGCGGATGTAGTACCAGGCAAGACTACGCAGA
ACGTGTCGCTGGTGAGGAGGAGGTATTGGGACGTGTACGACATGTATGTGTCGCTGGGGCCGCTGGTGGTTAC
AGTGGGCCTATCGGCCAAGGGCATTCCGGCGTTTAAGCCAGTGGAACAGTACAGCTGGCTTAGGGAGAGGAAC
GGCGTGTCCCAGCCTACCTCGGACTACGTGAAGACGCAGTGTGCAAATGTAGGAGGCTGTCCGAGCATTGAGC
TAGATAAACAGGCCGCCGAAGCCATCTTGGCCATCTCGCCGGAGTCCAATGGCGAAGTGGCCTACATGGCGTG
GTCAAACCTGGAGAGAGTGGTGGGCCTCTCGCTTAAGGACCTCCCCGCCAGGGAGCTCCGCATCACGTTTGAC
GACGTCGTCGCTCAGCCGAGGAGGGTCACGACGTCGCCTGTGTGGAACGGCATTGAGGCGCCTGGGAGGACGT
ATTCGCCGTTTACTGTGAACACGGAGCGCCTAGTGCCTTGGCGCACTTTGACGGGAAGGCAGCACCTCTACAT
TGACCACGAGTGGTTTAGGGAGTTGGGAGAGTCGTTCCCCATATATAAGAGGCCTTTGGATGTCGTGTTGGTG
AACGTTGTTAACAAGTTTGCCAGCGTGTCAGACTTCTGGTACGACCCTGCCAAGTATAGGAGGGAGGTGGGGG
GCGAGCGGAGGCTTGTGTTGCGCTTCTTGACGCCGCATGGGAAGTGGAACATACACTCTGAGTACTGGGACAA
CCTCATTATGCTCACGCTGTTTAGAGGTGGGCAGGTGGTCTGGCTCAACGACGAGGACGCCAAGTGGGCTGGC
ATAAACGACAACGACTGGATAGAGGTGATCAACGCAAACGGCGTAATTGTGGCTAGGGCCGCCGTGAGTCCCA
GGATTCCGAGGGGCACCGCGATTATGTACCACGCCCAGGAGAGGCACGTGTATGTTCCACTTTCTCCCACCAC
TGGAAAGCGCGCTGGCATTCACAACAGCGTTACTATTACTCACTTAAAGCCCACTAAGATGGTGGGCGGCTAC
GCGCAACTAAGCTACTCCTTTAACTACTACGGCCCCACTGGCGTAAATAGGGATACTCTTGTGGTAGTAAGAA
AGGCGAGTGGGGTGAGGCTATGA
>sim:M1627_0832-Ar nitrate reductase, alpha subunit; K00370 nitrate
reductase 1, alpha subunit [EC:1.7.99.4] (N)
ATGACATGGCAAAAATTTGAAAATAAAGGACGCGAATGGGAAGAGTTTTACAGGCAAAGATGGGCTTATGCAA
AGGCAATTAGATCTACACATGGCGTTAACTGTACGGGGTCTTGCAGTTGGCTGATATATGTAAAAGATGGTAT
TATAATTACAGAAATGCAAGCACTAGATTATCCTATTATAAATGAGGAAATTCCACCTTATGAACCTAGAGGA
TGTCCTAGAGGAGCCAGCTTTTCCTGGTATGAATATGCACCTCATAGAATAAAACATCCTTATATTCGTAAGC
AACTACTTAAGTTATGGAGAGAAGAATTAGAGAAGACAAAAGATCCTATCAAAGCTTGGGAAAATATAGTCGA
AGATCCGGAAAAGGTCAAGCTCTATAAGGAGAATAGAGGTAAAGGCGGATTCGTTAGAATTAGTTGGGATGAA
GTCGAGGAGTTAGTTTCTGCTGCGTTAATATATACGATTAAAAAATACGGACCAGATAGGATTGCTGGTTTTA
CACCAATACCTGCCATGTCAATGGTTAGTTATGCTGCAGGAACTAGATTTTTATCATTAATAGGAGGAGTAAT
AATGAGTTTCTATGACTGGTATGCGGATTTACCTATAGCTTCTCCTCAAGTATGGGGCGAACAAACTGATGTT
CCAGAAAGTGCTGATTGGTTTAACTCTACCTATATAATAGATTGGGGAACCAACATTCCTCAAACAAGAACAC
CAGATGCTCATTTTCTCTCCGAAGTCAGATATAGAGGAACTAAGGTAGTTGCGATTTCTCCGGATTATGCGGA
ATATGTAAAATTTGCAGACTTATGGCTACATCCTAAACCTGGTACTGACGGAGCACTAGCTTTAGCTATGGCG
CATGTAATAGCTAAAGAGTTCCATGTTGATAGGCAAGCGAAGTACTTTACAGAATATTTAAAGAAGTATACCG

ATGCTCCATTTTTAGTAATCTTGGAACAACAAAATGGAAGAGTTATATCTGGAAGATTTCTTAGAGCATCTGA
TATTATTGAAAACGTAATAAATGGAGAATGGAAGTTAGTAGTATATGATACATTAAAGGGGAGATTCGTAATT
CCAAATGGATCTATAGGGTTCAGATGGTCAGACGAAAAGAAATGGAATCTAAAACTACAAGATAGCTTAACGG
GAGAAAATATAGAACCAGCTTTAACATTTCTAGGGATAGAAGATCAAAGGATGATGATATCTCTCACTAGACT
TGATGAGAAAGGATTTATAGAAAGGGAAATCCCTGTCAAACTAGTAAAATTAGCTAATGGTAAACAAATTTAC
GTTACAACAGTATTTGATTTATTATTAGCCAATTTAGGTGTTAGAAGAGGAGATCTGAAAGGGTATCCAACTA
GTTATGACGATGATAAGCCTTATACTCCTGCTTGGCAAGAGAAAATAACGGGAGTCCCAAGGGATCTAGTAAT
ACAAGTAGCTAGAGAATTCGCCAAGAATGCGGAGGATACTAACGGTAAATCCATGATATTAGTTGGAGCTGGC
GTTAATCACTGGTTTAACAGTGACCTAATCTATAGGGCTATAATAACTATTTTAATGTTAATAGGTGCGATTG
GAGTTAATGGCGGGGGATGGGCACATTATGTTGGTCAAGAAAAAGTTAGACCTTTTGAGGGGTGGCAAACAAT
TGCTTTTGCAAGAGATTGGATGCTTGCTACAAGATTTCAAAACACTACTACTTGGATATACATTCATTCTGAT
CAATGGAAATATGATAGCTTAACATTAGATAAATTAACTCCTAATAACACATTGTATAAACATCCAGCAGATT
ACATAGTGTTAGCAGTAAAAAGAGGCTGGACGCCATTCTATCCACAATTTGATAAAAATCCTCTAAATCTCTC
TCCAGAAAATGCTATCGATAAGATTTCATCCGGTGAAGTGAAGTTTGCAATAACTGACCCAGATAATCCAATT
AATTTCCCGAGAATATTTTTCATTTGGAGAGCAAATTTACTGTTTTCTAGTGGAAAAGGGAGCGAATACATTC
TAAAGCACTTATTAGGTACTCATAACAGTGTGAGTAATACTGAAGATGCTAAAGATAAGGTGACTGAAATTAC
TTGGAGAGAACCTACACCAATAGGAAAGCTTGACCTAATAGTCGATCTTAACTTTAGAATGGATAGTAGTGCG
TTATATTCAGATATCGTGCTTCCAGCTGCAACATGGTATGAAAAATATGATATAAGTTCTACTGATATGCATA
CTTTTGTTCATCCATTTCAACCAGCTACAGATCCTTCTTGGGAAGCTAGGAATGATTGGGATATTTTCGTAAA
TTTAGCTAAGAAATTCTCAGAAATGGCATCGAGATATCTTAATGGAAAGCATAAGGATGTCGTCTATTTACCT
TTATCCCACGATACCCCAGATGAAATCTCACAATCTTTAATTGAGGAGGAATTAGATCCTCCTTATATTTCAG
CTCATCTTGCAAGTAAGATAATCCCAAAGAGGAACTTCGGGAATCTTATAGTCATTGAAAGAGATTATACTAA
AGTTTACGAGATGATGATTTCATTGGGGCATCTGATAAGAGATAAGGAATTACTTTATGTCGGCATACCCGTT
AATTATCGTAAGGAATACGAAGAATTGAAAGAAGAATTAGGAGAGATAGATGATAGACCAGATATTAAGGAAG
CTAAGAAAGTGGCTGAAGTAATACTTAGATTAAGCGGGGCTACTAACGGTGAGGTTTCAGTTAAAGAATATAA
ACAACTTGAAGTGAAAACGAGTCTTAACTTTAGCGATCTGTATAAAGGAATATCCGAAATAAAGATTAAGTTT
GACGATATAATAGCTTCACCTAAAAGAGTTATAGACTCACCTATCGAGTCCGGAATAGTGAAAGGAGAAAGGA
TTTATAATCCGTTCACGTTTAATATAGAGTATGAAGTACCTTGGAGGACATTATCTGGAAGGCAACATTTTTA
CTTAGATCATCCGATTATAAGGGAATTAGGAGAACAGCTACCAATCTATGCACCACCTTTAGAGTATAACAAA
GTAATTAAAGATAAGGATACCCTAGTTCTAAGGTATCTAACTCCACATGGAAAATGGCAAATTCATAGTACTT
TTATGGATAATTTAAGAATGTTAACACTATTCAGAGGTGGTCCGGTTATTTGGTTAAATGAAGAAGATGCAAG
AAGCGTGGGGATAAAAGATAATGAGTGGGTTGAAGTGTTTAACGAAAATGGAGCGACTGTAGTTAGAGCAGTA
GTTAGTAATAGAATACCCAGAGGTACAGCAATAATGTATCATGCTCAAGAACGTACAGTCTATATAAATGAGT
CTAGATTAAGGGGAATCATTGGAGGTTCCCATAATGCGGTTACTATGGTTAACATAAAACCAACGTGGTTAAT
CGGTGGATATGCACAACTCTCCTATGCTCTTAATTATTATGGAGCAGTTGGTACTCAAAGAGATACAATAGTA
GCTGTAAGGAGGTGGAGAACATGA
>hhi:HAH_1798-Ar narG; nitrate reductase subunit alpha (EC:1.7.99.4);
K00370 nitrate reductase 1, alpha subunit [EC:1.7.99.4] (N)
ATGAGTAGAAACGACCTGACTGACGACGAAACGGACAGTGCGGGAATCAGTCGCCGCGACTTCGTGCGCGGTC
TCGGAGCCGCGTCGCTGCTTGGCGCGACGGGGCTGTCCTTCGCCGACGACGGCATGGACGGCCTCGAAGCGGT
CGACGACCCCATCGGGTCGTATCCGTATCGGGACTGGGAGGACCTCTACCGCGACGAGTGGGACTGGGACTCC
GTCGCCCGCTCGACCCACAGCGTCAACTGCACCGGCTCCTGTTCGTGGAACGTCTACGTCAAGGACGGCCAGG
TCTGGCGCGAGGAACAGGCCGGCGACTACCCGACGTTCGACGAAAGCCTCCCCGACCCGAACCCGCGCGGGTG
CCAGAAAGGGGCCTGTTACACGGACTACGTCAACGCCGACCAGCGCGTGCTCCACCCCTTGCGCCGCACCGGC
GAGCGCGGCGAGGGCCAGTGGGAGCGGATTAGCTGGGACGAGGCGCTGACCGAAATCGCCGACCACGTCATCG
ACGAGGTGCAGGCGGGCCGGTACGACGCCATCTCCGGGTTCACGCCGATTCCGGCGATGAGCCCGGTGTCGTT
CGCCTCCGGCTCCCGCCTCGTCAATCTGCTGGGCGGCGTCTCGCATAGCTTCTACGACTGGTACTCGGACCTC
CCCCCGGGACAGCCGATCACCTGGGGCACCCAGACGGACAACGCCGAATCCGCCGACTGGCACAACTCTGACT
ACATCATCGCCTGGGGGTCGAACATCAACGTCACGCGCATCCCCGACGCGAAGTACTTCCTCGACGCCGGCTA
CGAGGGCGCAAAGCGGGTCGGTATCTTCACCGACTACTCCCAGACGGCCATCCACACCGACGAGTGGCTCTCG
CCCCACGGCGGCAGCGACACCGCGCTCGCGCTGGGCATGGCCCAGACCATCGTCGACGAGGGGCTGCACGACG
AGGAACACCTCAAAGAGCAGACCGACATGCCGCTGCTCGTCCGGGAGGACACCGGCAAGTTCCTCCGGGCGAG
CGAGGTCGGCCTCGCCGAGGACGCCGACGACCCGGACAAGGTGTTCGTCATGGTCGACGCCGACGGCAACCTC
CGTCGTGCACCCGGTTCGCTCGGCGAGCGGGACGGCCAGAAGGACTACTCGTTAAGTATCGAACTCGACTTCG

ACCCGCAGCTCTCGGTCGAGCGTAGTGTCGACACCGACGAGGGCAGCGTCGCGGTCCGGTCGGTCTGGGAGAA
CCTCACTGACGAACTCAGTCAGTACACGCCGGACGTTGTCCACGAAGAGACCGGCGTTGGGACGAACACCTAC
CAGCGGGTCGCCCGCGAGTTCGCCGAGGCCGACGCCGCGAAAATCATCCACGGCAAGGGCGTCAACGATTGGT
ACCACAACGACCTGGGCAACCGCGCCATCCAGTTGCTCGTGACGCTGACGGGCAATCTCGGCGACCCCGGCAC
CGGACTGGACCACTACGTGGGCCAGGAGAAGATCTGGTCGTTCCACGGCTGGAAGGTGCTCTCGTTCCCGACC
GGAAACGTCCGCGGCGTGCCGACGACGCTGTGGACCTACTTCCACGCGGGCATCCTCGACAACACTGATCCGG
ACACCGCAGAGAAGATACGCGAGTCCATCGACAAGGGATGGATGCCGGTCTACCCCGAGGAGCGCGAGAACGG
GAGCCGACCGGACCCGTCCACGATGTTCGTCTGGCGCGGGAACTACTTCAACCAGGCCAAGGGCAACGTCGCC
CTCGAAGAACAGCTCTGGCCCAAACTCGATCTCGTCGTCGACATCAACTTCCGGATGGACTCGACGGCGATGT
ACTCCGACATCGTCCTGCCGGCGGCCAGCCACTACGAGAAGTACGATCTCTCGGAGACGGACATGCACACCTA
CGTGCACCCGTTCACGCCGGCCGTCGAACCGCTCGGCGAGGCCAAGACCGACTGGGAGATATTCCGCCTGCTC
GCCGAGAAGATACAAGAGCGCGCCCAGGAACGCGGTGTCGAGCCGATCGAGGACCGCAAGTTCGACCGAACCA
TCGATCTCACGACCATCTACGACGACTACGTCCGCGACTGGGAGACCGGCGAGGAAGAAGGGCTCGTCGAAGA
CAAAGCCGCGGCGGAGTTCATCCTCGAACACTCCGAGGAGACCAACCCCGAGGGTAGCGACGAGCAGATAACC
TTCGACGACATCGACGAGCAACCACAGCGGTTCCTCGAAGCCGGCGACCACTGGAGTTCGGACATCAAGGACG
GCGAGGCCTACGTCCCGTGGCAGGACTACGTCCACGACAAGAACCCCTGGCCGACCTTCACCGGCCGCCAGCA
GTACTACATCGACCACGACTGGTATCTCGAACTCGGCGAGGAACTCCCGACCCACAAGGAGGGGCCGGAGAAC
ACCGGCGGGGACTACCCGCTGTCGTACAACACGCCTCACGGTCGCTGGTCCATCCACTCGACGTGGCGCGACG
ACCCCAAGATGCTCAGGCTCCAGCGGGGGGAGCCGGTGGTGTACATCAACCCCGAGGACGCCGAAGAACGCGG
CATCGAGGACGGCGACACCGTGGAGGTGTACAACGACCTCGGCGCGGTAGAGGTGCAGGCCAAAATCTACCCC
TCCAGCGAACCGGGGACCGTTCGGCACTTCTTCTCCTGGGAAAAGTTCCAGTACCCCGACCGGGACAACTTCA
ACACGCTCGTCCCGATGTACATGAAGCCGACGCAGTTGGTCCAGTACCCGGAAGACACCGGGGAACACCTGCA
CTTCTTCCCGAACTACTGGGGGCCGACCGGGGTCAACAGCGACGTGCGCGTGGACGTGCGGCCCACCGGAGGT
GACACCGAATGA
>hma:rrnAC1199-Ar narG; nitrate reductase subunit alpha; K00370 nitrate
reductase 1, alpha subunit [EC:1.7.99.4] (N)
ATGAGTAGAAACGACCTGACCGACGACGAGGGGGACAGTGCTGGAATCAGTCGCCGCGACTTCGTTCGTGGCC
TCGGAGCCGCATCGCTGCTTGGCGCGACGGGGCTGTCCTTCGCCGACGACGGCATGGACGGCCTCGAAGCGGT
CGACGACCCCATCGGGTCGTATCCGTATCGGGACTGGGAGGACCTGTACCGCGACGAGTGGGACTGGGACTCC
GTCGCCCGCTCGACCCACAGCGTCAACTGCACCGGCTCCTGTTCGTGGAACGTCTACGTCAAGGACGGCCAGG
TCTGGCGCGAGGAACAGGCCGGCGACTACCCGACGTTCGACGAGAGCCTCCCCGACCCCAATCCACGGGGCTG
CCAGAAGGGGGCGTGTTACACCGACTACGTCAACGCCGACCAGCGCGTGCTCCACCCCTTGCGCCGCACGGGC
GAGCGCGGCGAGGGCCAGTGGGAGCGGATTAGCTGGGACGAGGCGCTGACCGAAATCGCCGACCACGTCATCG
ACGAGGTGCAGGCGGGCCGGTACGACGCGATTTCGGGCTTTACCCCCATTCCGGCCATGTCCCCAGTAAGCTT
CGCCTCCGGCTCCCGGCTGGTCAATCTGCTCGGCGGTGTCTCCCATAGCTTCTACGACTGGTACTCGGACCTG
CCGCCGGGCCAGCCGATTACGTGGGGCACCCAGACGGACAACGCCGAGTCCGCCGACTGGCACAACGCCGATT
ACATCATCGCCTGGGGGTCGAACATCAACGTCACGCGCATCCCCGACGCGAAGTACTTCCTCGACGCCGGCTA
CGAGGGCGCAAAGCGGGTCGGTATCTTCACCGACTACTCCCAGACGGCCATCCACACCGACGAGTGGCTCTCG
CCCCACGGCGGCAGCGACACCGCGCTCGCGCTGGGCATGGCCCAGACCATCGTCGACGAGGGACTGCACGACG
AGGCACACCTCAAAGAGCAGACCGACATGCCGCTGCTCGTTCGGGAGGACACCGGGAAGTTCCTCCGGGCGAG
CGAGGTCGGCCTCGCCGAGGATGCCGACGACCCCGAGAAGGTCTTCGTTATGGTCGACGCCGACGGCACCCTC
CGTCGTGCACCCGGTTCGCTCGGCGAGCGGGACGGCCAGAAGGATTACTCCGCGAGCATCGAACTCGACTTCG
ACCCGCAGTTATCGGTCGAGCGGAGCGTCGATACCGACGAGGGCAGCGTCGCGGTCCGGTCGGTCTGGGAGAA
CCTCACCGACGAGCTCAGCCAGTATACGCCGGACGTTGTCCACGAAGAGACCGGCGTCGGCGAGAACACCTAC
CAGCGAGTCGCACGTGAGTTCGCCGAGGCCGACGCCGCGAAAATCATCCACGGCAAGGGCGTCAACGACTGGT
ACCACAACGACCTTGGCAACCGCGCCATCCAGTTGCTCGTGACGCTGACGGGGAACCTCGGCGACCCCGGCAC
CGGACTGGACCACTACGTCGGCCAGGAGAAGATCTGGTCGTTCCACGGCTGGAAGGTGCTCTCGTTCCCGACC
GGGAACGTCCGCGGCGTCCCGACGACGCTGTGGACCTACTTCCACGCAGGTATCCTCGACAACACTGATCCGG
ACACCGCAGAGAAGATACGCGAGTCCATCGACAAGGGATGGATGCCGGTCTACCCCGAGGAACGGGACGACGG
GAACCGACCGGACCCGTCCACGATGTTCGTCTGGCGCGGGAACTACTTCAACCAGGCCAAGGGCAACGTCGCC
CTCGAAGAACAGCTCTGGCCCAAACTCGATCTCGTCGTCGACATCAACTTCCGGATGGACTCGACGGCGATGT
ACTCCGACATCGTCCTGCCGGCGGCCAGCCACTACGAGAAGTACGATCTCTCGGAGACGGACATGCACACCTA
CGTGCACCCGTTCACGCCGGCCGTCGAACCGCTCGGCGAGGCCAAGACCGACTGGGAGATATTCCGCCTGCTC
GCCGAGAAGATACAAGAGCGCGCCCAGGAACGCGGTGTCGAGCCGATCGAGGACCGCAAGTTCGACCGGACCA

TCGATCTCACGACCATCTACGACGACTACGTCCGTGACTGGGAGACCGGCGAAGAACAAGGGCTCGTCGAAGA
CAAAGCCGCGGCGGAGTTCATCCTCGAACACTCTGAGGAGACCAACCCCGAGGGCAGCGACGAGCAGATAACC
TTCGACGACATCGACGAGCAACCACAGCGGTTCCTCGAAGCTGGCGACCACTGGAGTTCCGACATCAAAGACG
ACGAGGCGTACGTCCCGTGGCAGGACTACGTCCACGATAAGAACCCGTGGCCGACCTTCACCGGCCGCCAGCA
GTACTACATCGACCACGACTGGTATCTCGAACTCGGCGAGGAACTCCCGACCCACAAGGAGGGGCCGGAGAAC
ACCGGCGGGGACTACCCGCTGTCGTACAACACGCCTCACGGTCGTTGGTCCATCCACTCGACGTGGCGCGACG
ACACCAAGATGCTCAGGCTCCAGCGCGGCGAGCCCGTGGTGTACCTGAACCCCGAGGACGCCCAGCAGCGGGG
CATCGAGGACGGTGACACCGTGGAGGTGTACAACGACCTCGGGAGCGTCGAGGTGCAGGCGAAGATCTACCCC
TCCAGCGAACCGGGGACCGTCCGGCACTTCTTCTCCTGGGAGAAGTTCCAGTACCCCGACCGGGACAACTTCA
ACACGCTCGTCCCGATGTACATGAAGCCGACCCAGCTGGTCCAGTACCCGGAAGACACCGGGGAACACCTGCA
CTTCTTCCCGAACTACTGGGGGCCGACCGGGGTCAACAGCGACGTGCGTGTCGATGTCCGACCGAGCGGAGGT
GACACCGAATGA
>ape:APE_1288.1-Ar narG; nitrate reductase alpha subunit; K00370 nitrate reductase 1, alpha subunit [EC:1.7.99.4] (N)
TTGTCTCAGCAGAAGGGTGAAGGCAATGGTGGAGGTCCTGTCAAGCTGAGCAGGCGCGGCTTCTTGAAGGTGC
TCGCGGCCGCCGGCCTGCTGTCGAGCCTCGGCCCGCTGGCTTCCGCTCTCTCCAGCAACAGATATCTCACCAC
AATCGAGACCCCCCGCCTCGGCTACCCCGAGGACCTCAGGGGCTGGGAGAAGTTCTACAGGGACATGTGGGCC
TATGATAAGGTGGCGAGGAGCACCCACGGAGTCAACTGTACTGGGAGCTGCAGCTGGATGGTTTACGTCAAGG
ACGGTATAGTAGCTTACGAGCTCCAGGCTGGCGACTACCCTGACATAGGGCCCAGCTACCCCAACTACGAGCC
CAGGGGCTGTCCCAGGGGTGCAAGCACCTCCTGGTACCTCTACAGCCCCTTGAGGGTGAAGTACCCCTACGTC
AGGAAGCCTCTCCTGGAGCTTTGGAGGGAGGCTAGGAGCAGCTACAGCGATCCTGTGGAGGCCTGGGCTGGTA
TAGTCGAGGATCCTGATAAGAGGAGCAGGTATGTGAGGGCGAGGGGTCTTGGAGGCTGGGTCAGGGCCGACTG
GGACACCGTTCTGGAGATAATAGCCTCGGCCCTCGTGTACACAATCAAGAAGTACGGGCCCGACCGGATATTC
GGCTTCACCCCCATACCCGCAATGAGCCCCGTCAGCTACGCCAGCGGCGCCAGGTTCATAGAGCTAATAGGAG
GCTCCATGGGCAGCTTCTACGACTGGTACGCCGATCTTCCGCCGGCGAGCCCCCAGGTTTGGGGTGAGCAGAC
GGACGTCCCGGAGAGCGCTGACTGGTTCAACGCAGCCTACATTATAAACTTCGGCACCAACATACCGATGACC
AGGACTCCGGACGCCCAGTTCTTCACGGAGGTTAGGTACAAGGGCACGAAGATCGTGGTGGTGAGCCCAGACT
TCAGCGAGCACACCAGGTTCGCCGACGTCTGGGTCCCCATTAAGGAAGGCACCGACGGGGCCTTCGCCATCTC
TATGGCGCACGTTATACTGAGGGAGTACTACGTGGAGAGGCAGGTAGACTTCTTCATAGACTATGTCAAGAGG
TTTACAGACCTGCCCTTCCTGATCGTGCTGGAGCCCTATGAAGGCGGCTACAGGCCCGGCAAGTTCCTGAGGC
TCTCCGACCTACACCCCGAGGAGTACTACAGCGAGGGCTACAGCGGCGAGCCTAACAAGGAGTGGAAGCTCCT
CGTGTACGACTCGAACACAGGCAGGCCGAGGCTTGTGAACGGGAGCATAGGCTACCGGTGGGACGGCAGCGGC
AAGTGGAACCTCAAGCTCGAAGACCCGATCACCGGGGAGCCTGTGGACCCGAAGCTCTCCTTAATAGACGATA
GCGACGAGGTGGTGATGGTAAAGTTCCCGGTGTTCGGGGCCTCCTTCGGCGAGAAGGGCATAGTGGAGAGGGG
TGTGCCGGCGAAGAGGGTGAAGCTCGCCGACGGCAGCGAGGCCCTTGTGGCCACGGTGTTCGACCTCCTGGCG
GCCTATCTGGGCGTTGACAGGGGCCTCCCCGGCGACTATCCGGCGGGATACGATGATAAGAAGCCCTTCACCC
CGGCTTGGCAGGAGGACATAACCGGTGTGAGCAGGGAGCTCGCCATAAGACTCGCCAGGGAGTGGGCTGACAC
GGCGGTGAAGACCCGGGGCAGGGTCATGGTCATGCTGGGCCCGGGCGTCAACCACTGGTTCCACAGCGACCTC
CACTACAGGGCCATACTGACCCTAGTCAAGCTGACTGGAGCGGAAGGCAGGAACGGGGGAGGATGGGCCCACT
ACGTCGGCCAGGAGAAGATCAGGACGATAGCGGGCTGGGCCAAGCTGGCCTTCGCCCTGGACTGGGTCTTGCC
GCCGAGGCACCAGAACAGCCCGAGCTTCTACTACGTCCACACGGACCAGTGGAGGTATGACAGGCTGACCACG
GACTACTATGAGGGGCCCTGGAGCGACGTGAGGGAGAAGATGAAGTGTAGCCACCCGATGGACTGCAACATAA
AGGCCGCCAGGCTGGGCTGGCTACCCTTCTACCCCCAGTTTAACAGGAGCACGCTGGACCTAGCGGCGGAGGC
TAGGAGGCTTGGGAAGGATCCCGTGCAGCACGTGGTAGACCTTCTGAAGAGCGGCCAGCTGAAGCTGGCGATA
GAGGACCCGGACGCCCCTGAGAACTGGATTAGGGTCATGTTCGTCTGGAGGGCTAACCTCCTCGGCAGCAGCG
GCAAGGGCCACGAGTACTTCCTGAAGCACCTGCTGGGGAGCCCGATGGCCCAGCCTATGAGCGAGGAGGTGGA
CAGGAGCCTCATCAAGGAGGTTGTATGGAGGGCCGCCCCCGAGGGGAAGCTGGACCTCCTGGTGGCAATAGAC
TTCAGGATGGCCACCACCCCGATATACGCCGACATAGTGCTCCCCGCCGCGACATGGTATGAGAAGTACGATC
TGAGCATGACAGACCTCCACACGTTCCTACACCCCTTCACCCCCGCAGTGGACCCGCTTTGGGAGAGCAAGAG
CGACTGGGACATATTCAAAGAGCTGGCGAGGAAGTTCAGCGAGATAGCGGCCAACCACTTCCCAGACGAGGTC
GAGGACATCGTAGCCAGGCCCCTCTGGCACGACACGCCCATGGAGATAGCCCAGCCCTACGGTGTCGAGAAGG
ACTGGAGGTATGGCGAGACGGAGCCGGTGCCGGGCAAGACGATGTGGGACCTTAAGGTGGTTAAGAGGGACTA
CAGGAACGTGTACAAGATGTTCATAAGCCTGGGGCCGAAGGCCAGGAAGGCCGGCGTCAAGGGAGTAAGCTAC
GACACCAGCGACATCTACGAGATGCTGAAGGAGGACCTGGGCGTAGTGTACTGGGAGAAGTGTCCCGACGGCT

GTCCAAGCGTAGAGAGCGACAAGAACGCAGCCGAGGCTATACTAGCCCTGAGCCCTGAGGCCAACGGCCTGGT
GGGTGAGAGGAGCTTCGCCAGCCTCGAGGCCAAGGCTGGCGTGCCGCTAAAGGACCTGGCGAAGAGTAGGGAG
TGGTATAAGTTCGACGACCTTGTAGGCCAGCCTAGGAGGTCCCACACGAGCCCGATGTGGAGCGGTATAGAGG
ACTTCAACAGGGCCTACGCACCCTTCACCATAAACACGGAGAAGCTGGTCCCCTGGAGGACCCTCACCGGTAG
GCAGCACTTCTACCTCGACCACGACTGGTTCATAGCCCTCGGAGAGATGCTGCCTACCTACAAGCCTCCCGTT
GACCCTGTTAGGCTCGGCTACCTGAAGAACGCTGCGGCGAGGCTTGGGATCACGAGGATGAACGGCTACAGGA
TCGAGGAGGGCGGCAGGAGGTATCTCTTGCTGAGGTACCTCACGCCCCACGGTAAGTGGAACATACACAGCGA
GTTCTGGGACAACCTGAAGATGCTGACCCTGTTCAGGGGAGGCCAGGTGGTGTGGCTGAACGACGAGGACGCG
AGGTGGGCCGGGATAGAGGACAACGACTGGGTGGAGATAGTAAACGACAACGGAGTCATAGTGGCGAGGGTCG
CCACCAGCCCGAGGATACCCAAGGGGGTCGCCATAATGTACCACGCCCAGGAGAGGCATATCTACGTCAGGCC
GAGCAAGCTAACCGGCAAGAAGGGAGGGATCCACAACAGCGTCACCAGGGCAGACCTTAAGGTCAACCTCATG
GTGGGAGGCTACGCGCAGCTGAGCTACTTCTTCAACTACTACGGCCCGACGGGTGTCGACAGGGACACCATGG
TTATAGTATACCTACACGACAAGCTAGGCGCCAAGGCACCCCAGCCTGGAGCCTAG
>hvo:HVO_B0164-Ar narG; nitrate reductase, catalytic subunit
(EC:1.7.99.4); K00370 nitrate reductase 1, alpha subunit [EC:1.7.99.4]
(N)
ATGAACCGAACGAACACCTCGACCCCCGACGACGAGTCGGGAACGGACGAATCGCAAGCCGAACAGACGGACG
ACCCGCCGGCGGTCGGTGACCCGCCGGGAGAGCCGGCGACCGAGGGAACGAGCGTCTCCCGTCGTCGGTTCCT
CGAAGGCGTCGGAATCGCCTCGCTTCTCGGCATCGGCCGGTCCGCTGCCGACGACGACCCGCTGTTCCAGATG
GGCGGGCTGAACCCCGTCGACGACCCCATCGGGAACTACCCCTACCGGGACTGGGAGGACCTCTACCGCGAGC
AATGGGACTGGGACTCGGTCTCTCGGTCGACGCATAGCGTCAACTGTACCGGCAGCTGCTCGTGGGACGTCTA
CGTGAAAAACGGGCAGGTGTGGCGCGAGGAGCAGGCCGGTGACTACCCGCGGTTCGACGAGAGCCTGCCCGAC
CCGAACCCCCGCGGCTGTCAGAAAGGGGCGTGTTACACCGACTACGTCAACGCGGACGAGCGAATCAAACACC
CGCTGAAGCGCGTTGGCGACCGCGGCGAGGGGAAGTGGCGGCGCATCTCGTGGGACGAGGCGCTCACCGAAAT
CGCCGAGCACGTCGTCGACGAGGTGCAGGCCGGTCGCTACGACGCCATCAGCGGCTTCACTCCCATCCCGGCG
ATGAGCCCCGTCTCGTTCGCCAGCGGGTCGCGGCTCATCAACCTCCTCGGCGGCGTCAGCCACTCGTTTTACG
ACTGGTACTCCGACCTGCCGCCGGGCCAGCCCATCACGTGGGGCACCCAGACCGACAACGCCGAGAGCGCCGA
CTGGTACAACGCCGACTACATCATCGCGTGGGGGTCGAACATCAACGTCACCCGCATCCCCGACGCGAAGTAC
TTCCTCGAATCGGGCTACAACGGGACGAAGCGCGTCGGCGTCTTCACCGACTACTCGCAGACGGCCATCCACA
CCGACGAGTGGCTCAGCCCCGATTCGGGCACCGACACCGCGCTCGCGCTCGGTATGGCCCGGACCATCGTCGA
CGAGGGACTGTACGACGAGGCCCACCTCAAAGAGCAGACCGACATGCCGCTTTTGGTCCGGCAGGACACCGGG
AAGTTCCTCCGCGCGAGCGAGGTTCCCTCGGTCTCGACCGACGCCGACCGACCCGAGTGGATGTTGCTCATGC
TCGACTCTGACGGACGCCTCCGCGAGGCACCGGGGTCGCTCGGTGAACGCGACGGCCAGAAAGACTACTCCAA
GAGCATCGAACTCGACTTCGACCCGCAACTGGACGGCGAAACCACGGTCCGAACCGAAGACGGGCGCGTCCGC
GTCCGGACCGTGTGGGCCGAACTGCGGGACGAACTCGATGACTGGGGCCCGGAGACCGTCCACGACGAGACCG
GCGTCGGCGAAGAGACCTACCAGCGCGTCGCCCGCGAGTTCGCCGAGGCCGACAAGGCGAAGATAATCCAGGG
CAAGGGCGTCAACGACTGGTACCACAACGACCTCGGGAACCGCGCGCTCCAGTTGCTCGTCACGCTGACGGGC
AACCTCGGCGAGCAGGGGACCGGCCTCGACCACTACGTCGGCCAAGAGAAGATATGGACGTTCCACGGCTGGA
AGTCGCTGTCGTTCCCGACGGGCAACGTCCGCGGGGTGCCGACGACGCTGTGGACCTACTACCACGCGGGCAT
CCTCGACAACACCGACGCCGACACGGCCGCGAAGATACGGGAGTCCATCGACGAGGGATGGATGCCGGTCTAC
CCCGAGGAACGCGACGACGGCTCTCGTCCCGACCCGACGACCATGTTCGTCTGGCGCGGCAACTACTTCAACC
AGGCGAAGGGGAACGTCGCCGTCGAGGAGGAACTGTGGCCGAAACTCGACCTCGTCGTCGACATCAACTTCCG
GATGGACTCGACGGCGCTGTACTCGGACATCGTGTTGCCGACCGCGAGCCACTACGAGAAACACGACCTCTCG
ATGACGGACATGCACAGCTACGTGCATCCCTTCACGCCCGCGGTCGAACCGCTGGGGGAGTCGAAGACCGACT
GGCAGATATTCCGCGAACTCGCCGCGAAGATTCAGGAGGTCGCCACGGAGCGCGGCGTCGGGCCGATTTCGGA
CCGGAAGTTCGACCGCGACATCGACCTCCAGTCCGTCTACGACGACTACGTCCGCGACTGGGAGACCGGCGAA
GCCGACGCGCTCGACGAGGACCGCGCCGCCTGCGAGTACATCCTCGAACACTCCGAGGAATCGAATCCGTCCG
AGAGCGACGACCAGATAACGTTCGCCGACACGGTCGAACAACCCCAGCGGCTCTTGGCCGCCGGCGACCACTG
GACCTCCGACATCGAGGACGGGGCGGCGTACACGCCGTGGAAGGACTTCGTCCAGGACAAAAACCCGTGGCCG
ACCGTCACCGGCAGACAGCAGTACTACATCGACCACGACTGGTTCCTCGAACTCGGCGAGCAGTTGCCGACGC
ACAAAGAGGGGCCGACGCGGACGGGCGGCGACTATCCGATGGAGTACAACACGCCCCACGGGCGGTGGTCTAT
CCACTCGACTTGGCGAGACAACGAAAAACTGCTCCGCCTCCAGCGCGGGGAGCCGGTGGTCTACCTCCATCCC
GAGGACGCCGCCGAGCGCGGCATCGAAGACGGCGACACCGTCGAGGTGTTCAACGACCTCGCGGAGGTCGAAC
TGCAGGCGAAGCTCTACCCGAGCAGCCAGCGCGGCACGGCGCGGATGTACTTCGCCTGGGAGCGGTTCCAGTT

CGACGGCGACACGAACTTCAACTCGCTCGTCCCGATGTACATGAAGCCGACGCAGTTGGTCCAGTATCCGGAA
GACTCCGGCGAACACCTCTACTTCTTCCCCAACTACTGGGGGCCGACGGGGGTCAACAGCGACGTCCGCGTCG
ACGTGCGGAAAGGCGGGGGTGATGCCGAATGA
>htu:Htur_1649-Ar molybdopterin oxidoreductase; K00370 nitrate reductase 1, alpha subunit [EC:1.7.99.4] (N)
ATGAGTGACGAGGAACCCGACGGGCTCGAGCTGACCCGTCGGGAACTCGGCGCCGCGGCGGCGGGTATCGCCG
GCGCGTCCGGCCTCGGTTTCCTCGGCTACCGGCGGCTGACCGGCGAGGAAACCGCCGACGAGGACGGCGAGGG
ACCGATGAACCCCCTCGAGGAGTACCCAAACGAGGAGTGGGACCAGAAGTACCGCGACATCTGGGAGCCCGAC
GACTCCTACATGCTCACGTGTACGCCCAACGACACCCACAACTGCTACCTCGAGGCCACGATCAAGAACGGCC
AGATCACGCGCCTCGGTCCCTCGATGAACTACGGCGAGGCGACCGACCTCGACGGCAACCAGGCCTCCCAGCG
GTGGGACCCCCGCGTCTGTAACAAGGGGCTGGCGATGGTCGAGCGCTTCTACAACGAACGGCGCGTCAAGGCC
CCGATGATCCGTCAGGGCTTCAAGCAGTGGGTCGACGACGGCTTCCCGCGCGATTCCGACGGCTCGATGCCCG
AGGAATATGCCCGGCGCGGAGAGGACGACTTCGTCGAGGTCTCCCACGAGGAAGCCCACGAGTACGCCGCCCG
CACCTTCCTCGAGCTCGCCGACCAGTACAGCGGCGAGAGCGGGATCCAGTCGCTGCTCGATCAGGGCTACGAC
GAGCGCGTCGTCGAGGAGACGCAGGGCGCCGGCGTCCGGACGATGAAGTTCCGCGGCGGGATGCCGCTGCTCG
GCGTCATCAAGCTGTTCGGCCAGTACCGAAACGCCAACTCGATGGCGTTGCTGGACAACTACGTCCGCGACGT
TAGCGAGGACGAAGCCCTGGGCGCGATGGGGCTGGACAACTACTCGTTCCACACCGACCTGCCGCCGGGCCAC
ACGATGGTCACCGGTCAGCAGACGGTCGACTTCGACCTCGCGAGCATCGAGTACGCGGACCACATCGTGCTCG
ACGGGATCAACTACCTGACCTCGAAGATGGCCGACTGTCACTGGCTGACCGAGGCCAAACTGAAGGGGTCGAA
GGTCACCGGGATCTTCACCGACTACAACGCGACGGCCTCGAAGTGCGACGAACTCATCACGGTCCGGCCGGCG
TCGGACACGGCGCTGTTCCTCGGCGCTGCGCGCGTACTCATCGAGGAAGAGCTCTACGACGAGGAGTACGTCC
GGCAGTTCACGGACCTCCCGCTGCTGGTCCGCATGGACGACAACGAACTGCTCCGCGCCAGCGACCTCTCCGC
GGACTACGAGCCCGCGGACTTAGAGAAGACCGACGCCGTCGCCGACGACGCGGACCGCCCCGGCGTCGACGTG
ACGAACATCGACGATCAGGTCATCACGGAGGAGCTCCGCCGCGAGTGGGGCGACTTCGTCGTCCACAACGCCG
AGAGCGGCGAGTTCGAGCCGGTCACGCGCGACGACATCGGCGACGACTTCGACGTTCCCGCGACGATCGAGGG
CGAGTTCGAGATCGAGCTCGCCGACGGCGAGACCGTCGAAGTGCGGACGGTGTTCGACCTGATCAAGGAACAC
CTCGTCGGCACCTGGGACCTCGAGTCGACCGCCGAAGTCACGGGGACCGATCCGCAGGCGATCGAGAACCTGG
CCCAGGACTTCGCGGACAACCAGCAGAGCACGATGCTGCTGACCGGGATGGGGCCGAACCACTACGCGAACGC
CGACCAGCACGGCCGTGCGGTGTTCCTGCTGGCGTCGCTGACGCGGAACGTCGGCTACCAGACGGGGAACGTC
GGTTCCTACTCCGGGAACTACCGGATGGCGTACTTCAACGGCGTCGGCCAGTACGCCAACGAGGATCCGTTCG
ACGTCGAACTCGATCCCGAGGAGCCGGCCACCACCGAGAAGCGCTGGGACGCCCACTCGGCGCACTTCTACAC
GAACCTCGACAAGCCGCTGAAGGTCGACGGCGAGTACTTCCAGGGCGACTCGCACATGCACACGCCCACGAAG
TCGATCTGGGTGTCGGGGTCGAACTCCATCCTCGGCAACGCGAAGGGGACCTACAAGATCATCGAGAAGGCCC
TGCGCACCGGCGACATCGAGGCCTTCTTCACCAACGAGTGGTGGTGGTCGATGACCTGCGAGTACTCCGACAT
CGTCTTCCCGGCGGACTCGTGGGCCGAACACCACGTCCACGACATCACCGCCTCCGTCACGAACCCCTTCATC
ACGACGATGCCGGAGACGGAGATCGACCGGATCTACAACACGCTCAACGACACTCAACACTACAAGGGCGTCG
CCGAGAAGCTCGCCGAACTCACCGGCGACAGTCGCTTCGAGGACTACTGGGCGTTCATCGACGAGGAGGAGTA
CCAGGCCAAACCCTACATCCAGCGCATCTTCGATCACTCCAACGCCGTCAAAGGGTACGACGTCGAGAACCTG
CTGGAAGACGCCCGCGAGGGGACACCGGCGATCATCATGACCCGGACCTACCCCAAGCACGTCGGCAACGAGC
AGACCCAGGACTCCCAGCCCTGGTACACGAAGACCGGGCGCCTCGAGTTCTTCCGCGAGGAGGAGGAGTTCGC
CGAGGCCGGCGAGCACATCCCCCTCCACCGGGAGCCGATGGACGCGACGCCCTACGAGCCGAACGTCATCGTC
GACGACAGCGACAGTCCGGTGATCGCGCCCGAGACCCCCGGCGACCGCGGCTGGGACAGCGAGGCGAAGGCCC
GCGACACCAACGACCGACAGGTCCGCAATGAGATCCGGTCGCCGTCGGAACTGGTCGACACGACCCATCCCCT
GACCGATGTCGACGACGGCTACGACTACGTCTACATGACGCCGAAGTACCGCCACGGGACCCACACCTTCGGC
GCCGACCTGAAGGACATGGCCGTCTGGTGGAGCAACTTCGGCGACATGGGCCGGGAGACGGAGCGCGACTCCT
TCGACCAGCGCAAACCGTTCGTCGGCGAGGGCTACGTCGAGATGAACCCCGAGGACGCCCGCGAGGAGGGGCT
CCAGGACGGCGATTACGTGTGGGTCGACGCCGACCCCTCGGACCGTCCGTACCCGGGCTACGACCCCGACGAC
GAGAAGACTGAGTACACGCGGGCGATGATGCGAGTCCGCTACCAGCCGAGCATCCCGCGAGGGGTCACGCGGA
GCTGGATGAACGTCAGCCAGACCTCCCACAAGTCCTACGAGGCCCAGGAGGAACGCGAGGACGGGAAGGCCCA
GTCCGAGGACACGAACTACGTGTCGATGTACCGCAGCGGCGGTCACCAGTCGATGACCTCGACGTGGCTGCGA
CGGACCTGGCTGACCGATACGCTGCCCCGAAAGGGCATGTTCGGACAGAACGTCGACGTCGGCTTCGAGCCCG
ACGTCCACGCCGCCAACGGCGCGCCCAAGGAGTCGTTCGTCAAGATCGAGAAGGCCGAAGACGGCGGCGTGAA
CGAGGACGGCGAGCCCGATGGTGACTGGCGACCAGTCGGAGAGGGCGTCCGTCCGACCCAGGAGAACGACCGG
ATGAAACAGTACCTAGAGGGCGGTTTCACGAGCGAATCAGACTGA

>hxa:Halxa_3206-Ar molybdopterin oxidoreductase; K00370 nitrate reductase 1, alpha subunit [EC:1.7.99.4] (N)
ATGAGTGACGGGGAGGCCGACGCCGAGGACGGCGGACTGAATCTGACCCGCCGCGAGCTCGGTGCGGCGGCCG
CCGGACTCGCCGGCGCGTCCGGGCTCGGATTCCTCGGCTACCGCCGGCTGAGTCCCGAGGAAGCCGCCGACGA
CGAGGGCGGCCCCGAGGTCGAGATGAACCCCCTCGAGGAGTACCCAAACGAGGAGTGGGATCAGAAGTACCGG
GACATCTGGGAGCCCGACGACTCCTACATGCTCACGTGTACGCCCAACGACACCCACAACTGCTACCTCGAGG
CGACCGTCAAGAACGGCCAGATCACCCGCCTCGGTCCGTCGATGAACTACGGCGAGGCGACGGACCTGCAGGG
CAACCAGTCCTCCCAGCGCTGGGACCCCCGCGTCTGTAACAAGGGCCTGGCGATGGTCGAGCGCTTCTACAAC
GAACGCCGCGTCAAGGCCCCGATGATCCGCGAGGGGTTCAAGCAGTGGGTCGACGACGGCTTCCCGCGCGACG
ACGACGGCTCCATGCCCACCGAGTACGCCCAGCGCGGCGAGGACGACTTCGTCGAGGTCTCCCAGGAGGAGGC
CCACGAGTACGCCGCTCAGACCTTCCTCGAGCTCGCCCAGCAGTACAGCGGCGAGAGCGGGATGGAGTCGCTG
CTCGAGCAGGGCTACGACGAGCGCGTCGTCGAGGAGACCCAGGGCGCCGGCGTCCGGACGATGAAGTTCCGCG
GCGGGATGCCGCTGCTCGGCGTCATCAAGCTGTTCGGCCAGTACCGCAACGCCAACTCGATGGCGCTGCTGGA
CAACTACGTCCGGGACGTCTCGGAGGACGAGGCCCTGGGCGCAATGGGGCTCGACAACTACTCGTTCCACACC
GACCTGCCGCCGGGCCACACGATGGTGACCGGCCAGCAGACGGTCGACTTCGACCTCGCGAGCATCGAGTACG
CGGACCACATCGTGCTCGACGGCATCAATTACCTGACCTCGAAGATGGCCGACTGTCACTGGCTGACCGAGGC
CAAGCTCAAAGGGTCGAAGCTCACCGGCATCTTCACCGACTACAACGCCACGGCCTCGAAGTGCGACGAGCTC
ATCACGGTCCGCCCGGCGACCGACTCGGCGCTGTTCCTCGGCGCCGCGCGGGTCATCATGGAGCAGGAGCTCT
ACGACACGGAGTACGTCCGCAAACACACGGACCTCCCGCTGCTGGTCCGGATGGACGACAACGAACTCCTCCG
CGCCAGCGACGTCTTCTCGGACTACGAACCCGCCGACCTCGAGAAGACCGAGGCCGTGGCCGACGACGCCGAC
CGGCCCGGCGTCGACGTGACGGACATCGACGATCAGGTCATCACCGAGGAACTCCGCCGCGAGTGGGGCGACT
TCGTCGTCCGCAACGACGAGAGCGGCGAGTTCGAGTCGGTCACCCGCGACGACGTCGGCGACGACTTCGACGT
CCCCGCGACGATCGAGGGCGAGTTCGAGGTCGAACTCGCCGACGGCAGCACCGTCGAAGTCCGGACGGTCTTC
GACCTGATCAAGGAGCACCTGACCGAGACCTGGGACCTCGAGTCGACCGCCGAGGTCACGGGGACGGACCCGC
AGGCCGTCGAGAACTTAGCTCAGGACTTCGCGGACAACCAGCAGAGCACGATGCTGCTAACCGGGATGGGGCC
GAACCACTACGCGAACGCCGACCAGCACGGCCGCGCCGCGTTCCTGCTGGCGTCGCTGACGCGCAACGTCGGC
TACCAGACCGGGAACGTCGGCTCCTACTCCGGGAACTACCGGATGGCCTACTTCAACGGCGTCGGGCAGTACG
CCAACGAGGATCCGTTCGATCCCGAACTCGATCCCGACGCGGAGGCTCGCACCGACAAGCGCTGGGACGCCCA
CTCGGCGCACTTCTATACCAACCTCGACAAGCCGCTGAAGGTCGACGGCGAGTACTTCCAGGGCGACTCGCAC
ATGCACACGCCCACGAAGTCGATCTGGGTGTCCGGCTCGAACTCCATCCTCGGCAACGCCAAAGGCGCCTACA
AGATCATCGAGAAGGCCCTGCGCACCGGCGACATCGAGGCCTTCTTCACCAACGAGTGGTGGTGGTCGATGAC
CTGCGAGTACTCCGACATCGTCTTCCCGGCGGACTCGTGGGCCGAACACCACGTCCACGACATCACCGCCTCC
GTCACGAACCCCTTCATCACGACGATGCCGGAGACGGAGCTCGACCGGATCTACAACACCCTAAACGACACCC
AACACTACAAGGGCGTCGCCGAGAAGCTCGCCGAACTCACCGGCGACGACCGGTTTACCGACTACTGGGCGTT
CATCGACGAGGAGGAGTACCAGGCCAAACCGTACCTCCAGCGCATCCTCGACAACTCCAACGCCGTCAAGGGC
TACGACGTCGAGGACCTGCTGGAAGACGCCCGCGAGGGGACGCCTGCGATCATCATGACCCGGACCTACCCCA
AGCACGTCGGCAACGAGCAGACCCAGGACTCCCAGCCCTGGTACACGAAGACCGGGCGGCTCGAGTTCTTCCG
CGAGGAGGAGGAGTTCGCCGAGGCCGGCGAGCACATTCCCCTCCATCGGGAGCCGATGGACGCGACGCCGTAC
GAGCCGAACGTCATCGTCGACGACAGCGACAGTCCGGTGGTTGCGCCCGAGACGCCCGACGACCGCGACTGGG
ACAGCGAGGAGAAGGCCCGCGACACCAACGATCGGCAGGTCCGCAACGAGGTCCGGACGCCGTCGGAACTGGT
CGATACCAGCCACCCGCTGACCGACCTCGACGAGGGCTACGACTACGTCTACATGACGCCGAAGTACCGCCAC
GGGACCCACACCTTCGGCGCCGACCTCGAGGAGATGGCGGTCTGGTGGAGTAACTTCGGCGACCAGGGTCGCA
AGACGGAGCGCGACTCCTTCGACCAGCGCAAGCCGTTCGTGGGCGAGGGCTACGTCGAGATGAACCCAGAGGA
CGCCCGCGAGGAGGACTTACAGGATGGCGACTACGTGTGGGTCGACGCCGACCCGTCCGACCGGCCGTTCCCG
GGCTACGACCCCGACGACGAGGAGACCGAGTACACCCGCGCGCTGATGCGGGTCCGCTTCCAGCCGAGCCTCC
CGCGCGGGGTCACTCGCAGCTGGATGAACGTCAGCCAGACGTCCCACAAGTCCTACGAGGCCCAGCAGGAGCG
CGACGACGGCAAGGCCCAGTCCGAGGACACGAACTACGTGTCGATGTACCGCAGCGGCGGCCACCAGTCGATG
ACCTCGACGTGGCTGCGCCGGACGTGGCTGACCGACTCGATGCCCCGCAAGGACATGCTCGGGCAGAACATGG
ACGTCGGCTTCGAGCCCGACGTCCACGCCGCCAACGGCGCGCCCAAGGAGTCGTTCGTCAAGATCGAGAAGGC
CGAAGACGGCGGCGTGAACGAGGACGGCGAGCCCGGCGGCAACTGGCGACCAGTCGATGAGGGCGTCCGGCCG
ACGCAGGAAGACGACCGGATGGAACAGTACCTGAAGGGCGGTTACACGAGCGAATCGGATTAA
>hhi:HAH_4416-Ar hypothetical protein; K00370 nitrate reductase 1, alpha subunit [EC:1.7.99.4] (N)

GTGAAACTGTCACGGCGGGACTTCCTGACCGCGGCTGGCGCAGGGACGGTCGGCGCGCTCGCCGAAGGTGCTT
GGGGCGCGACACAGTCCACGGAACCGATAACGAGCGTGGACAACCCGCTCAAATCCTATCCGAACCGGGACTG
GGAGCAGGTGTACCACGACATCTACGCCTACGACGAGGTGGACTGGACGGTGTGTCACCCCAACTGCACCCAG
TCGTGTGCGCTGAACTTCTACATGAAAAACGGCGTCCCGATACGGGCCGAGCAGGTGTATCACAACGAGGAAC
CCTCGGTCGGCCCGTCGGGCTACGAGGACGCCGGCGTCAGCCAGCACTGGAACCCCCGTGGCTGTATGAAGGG
GCTCTCCCTGCACCGCCGGACGTTCGAGCCGTCGCGCATCAAGTACCCGATGGTCCGCAAGGGGTGGGACCCC
GAGGACCCTAACCCCGAGGGCCGCGGCGAGGACGAGTTCGAGCGCGTCTCCTGGGACGAGGCCATCGACCTCA
TCGCCGAGAAGATGGCGAACTTAGAGGACAACAAGCGGTTCCACATCTTCAACGCCATCAAGGCAGATGGACT
GTTCACCCGGCACGGCTCGGGCCGCCGACTGGCGTCCATCTTCGGCGGCTGTGAGTGGACCGAGTACGACTGG
TACGCCGACCTGCCGCCGGGCCACGTCATCACCACTGGTTACCAGACATCCGATTCTGACGCCGTCGCCTGGC
GCAAAGCCGACTACACCATCATCCAGGGGAAGAACCTCATCCACAACAAGCTCGCAGACAACCACTTCCTGCA
GGAGACCCGCGAGCGCGGTGGGAAGATGGTCGGCGTCTACCCGGACTACTCGCCGACGGTCCAGAAGTGTGAC
CGGTGGCTGCCGATCCGCCCCGGGAGCGACCCGGCGTTCGCCCTCGGCATGGCCCACGTCATCATGGCCGAGG
AACTGTACGACGCCGAGTTCATGCGGAAGTTCACGAGTCTTCCCCTACTCATCCGGGAGGACACAGGCAAATA
CCTTCGCGCACACGAGGTGTTCGAGGACGTCGAACCGCCCGCGGAGGACATCGACGAGCAGCACGAGCACAAC
GACTGGGGCGACTTCGTGGTGCTGGGTGAGGACGGCCAGCCCGTAGCGATGACCCGCGAGGAGGTCGGCGAGG
AGATGCCGACCACGCCACAGCTAGACGTCGACAGCGAGGTGGCACTGACCGATGGCTCGACTGTCGCCGTCAG
GAGCGACTTCGCCCGCCAGCGGGACAACATCATCGAGAACTACGACCCGGAGACGGTCGAGGAGATCACCACG
ATTCCGGCGGACAAGCTCGCCCAGACCGCGCGGGAGTTCGCCGACGCCGAGAAGGGCCAGTGGTTCACCGGCG
AGGGGATCAACCACTGGTTCCACTCCAACGACGCGCTCCAGCGGGCGGTGTTCCTCGTCCAGGCGATGCTGGG
CAACATCGGCGACGCCGGCCAGGGGTACTACAACTACTCCGGCCAGTACAAGATCGAACTGCTGGACGGGTAC
CCCCACTACGTCAACCCGGACGGCAACGCCGCCCACGGGATGTATCCGGGCTACGCCTTCGCCTTCTTCGGCG
GCGAGAAACTCGACCCCCACGAGATTCGCGGCGACTTCGACCGCGAGATGGACCTCGTCCCTCAGGGCGACGC
CGAGGAGCCGGTGATGCCGAAGAACGCCGGCTCGTACACGATGTCCAAGCCGTCGATCCTGTGGACGATGAAC
TGCAACCTCCTGAACCAGACCAAACATCAGGAGCACGTCATCGAGAACTTCGTCAAACACCCAGACACCGAGA
ACGACCTGTTCATCGTCTCGGACATGCACATGACCTACTCCGCCAGACACGCCGACATCGTGCTCCCGGTGCC
GTCCTGGCTGGAGTGTGAGTACCCCGACATCACCATCGGGCCGGAGAACCCGTTCGTCCAGATGGACCACGGC
GTGATGGACCCCATCTACGACACCAAACAGGACGGGGAAGCCGTCGCGATGGTCGCCGAGAAACTGGACGAGA
AGATTCCGCCCGAGGAGCGAAACGTCGACTCCTACCGGGCGTACTTCGAGGAGTTCCTGGAGGAAGACGGCGA
CGTCAAGAACTACATCCAAGAGACCTTCGACCGCGGGATGACGACCCAGAGCATCGACGTCGAGGACTTGGAG
GATGGCCCGGAACGGCTAGACCTCAAGACCTATCCGCGGATTCCGTTCTACTCGCAGGTCCACGAAGACCGAC
CGTTCTACACGAAGACCGGCCGGATGGAGTTCCACAAGGAGGAGGACCGGTTCATCGAACTCGGTCGGGACGA
CATCGACCACGTCGAGAGCGTCGAGGGGACTCCCTACGGCGTGAACAAGAAGTGGGACGAAGCCAAGGACGAG
GAGAACCCGCTGTATCACGACGAGGGCAACCAGTTCTACTACAACACCCCCCACCCCAAGTACCGGACCCACT
CCTCGTGGGGGATGACGGACTGGAACCTCATCTGGGCCTCGCGGGACTTCGGCAGCGTCAGCGGCGACCCGGA
GGGGACCGACCGGTTGGTCGATGACTTCTCGTTCCCCACCGGGGACGGCGAGTCCGAAGACGTCGCGCCGCTC
GGTGAAGCGTTCGTCGAGATGCATCCAAGCGACGCAGCAGACATCGATGTAGAGAACGGTGACTACGTCCGCA
TCACCGGCAAGCGCGGCGACCTCGTCGTCAGAGCGATGGTCAGCGAGCGTCAGCGCCCGCGGTCGGCCGGCGA
CATGGGACAGCTGACGCTGTGGCACGGCTGGTGGCCCCAGCACTTCCCCGACGACGAGGCCGACGAGGACGGC
GTCAAGGGGTACAACACGATCACCAACATCTGGCTCGATCCGCTGCAGGAGACCGACGACCTCGTCCACAAGG
CGGTGTTCGGCGACCCCAACGTCTCCGACATCGTCGACGAGGACATCGCCTGGCACGGCGCAGAACTGGAGCA
CGGCTACGAGGAGACGGTGTGGGCTCCGACGGGGACCAACCGGGACGACCTCGTGGACGTGGAGAAGTACGAG
GAAGCGGACTGGTGGCCCGGCGACGCCCGCCGGGACGACCTCGTCGAGGACTACATCGGCGGGTCCTTGCAAC
CCAGCGGTAGCGGCTCTGGCAGTACCAGCAGGAAGACCCCAGGAGGCGATGACTGA
>hla:Hlac_1335-Ar DMSO reductase family type II enzyme, molybdopterin subunit; K00370 nitrate reductase 1, alpha subunit [EC:1.7.99.4] (N)
ATGACTGACACGACAAACACGTCCGACGAATCGACACGCGACGAGTCCGGTACCGCGCGCCGCGACTTCCTGA
AGGGGGCCGGGGTGGCGGCCGCCGTCGGCGCCACCGGCTTGGGCTCCGCGCAGGACCTCACCGAGATGACCGC
GCTGGAGGTCGTCGACGACCCGATCGGCAACTACCCGTACCGCGACTGGGAGGACCTCTACCGCGAGGAGTGG
GACTGGGACGGGAAGGCCCGCTCGACGCACAGCGTCAACTGCACGGGCAGCTGCTCGTGGCAGGTGTACACGC
GGAATGGGCAGGTGTGGCGCGAGGAGCAAGCCGGCGACTACCCCCGATTCGACGAGTCGCTCCCGGATCCGAA
CCCGCGGGGCTGTCAGAAGGGAGCGTGTTTCAGCGACTACGTGAACGCCGACCAGCGCGTCACGCACCCGCTC
CGTCGGACCGGCGAGCGCGGCGAGGGGAAGTGGGAGCGGATCTCCTGGGACGAGGCGCTCACCGAGATCGCCG
AGGAGGTCATCGACGCCGTCGAGGACGAGGAGTACGACGCGATCAGCGGCTTCACCCCGATCCCGGCGATGAG

CCCGGTCTCGTTCGCCTCTGGGAGCCGCCTCGTCAACCTGCTCGGCGGCGTCAGCCACTCCTTCTACGACTGG
TACTCCGACCTCCCGCCGGGACAGCCGATCACGTGGGGGACCCAGACCGAGAACGCCGAGAGCGCGGACTGGT
ACAACGCCGACTACATCATCGCGTGGGGCTCGAACATCAACGTCACGCGGATCCCCGATGCGAAGTACTTCCT
CGAAGCCGGCTACGACGGCGCCAAGCGCGTCGGCATCTTCACCGACTACAGCCAGACGGCGATCCACTGCGAC
GAGTGGATCGGCCCGGAGCCCGGCAGCGACACGGCGCTCGCGCTCGGGATGGCGCGCACCATCGTCGACGAGG
GGCTCTACGACGAGGAGCATTTAAAAGAGCAGACGGACATGCCGCTGCTCGTCCGCGAGGACACCGGGAAGTT
CCTCCGCGCGAGCGAGGTGTCCGGGCTGAGCGTCGACGCCGACCGGCCGGAGAAGGTGTTCGTCATGCAGGAC
GCCGACGGCACCCTCCGGACGGCGCCCGGATCGCTGGGCGAGCGCGACGGACAGCACGACCACTCCGTGAGCA
TCGAGCTCGACTTCGACCCCGAACTCGCCGTCAAGCGCACGGTCGGCACGACCGACGGCGACATCGAGACGCG
CTCGGTGTGGCTGAATCTCCGCGACGAGCTGTCGGAGTACACCCCCGAGCGCGTCAACGAGATCACGGGCGTC
GGCCGGCAGACTCACCAGAAGATCGCCCGCGAGTTCGCCGAAGTCGACCGCGCGAAGATCATCCACGGGAAGG
GGGTCAACGACTGGTACCACAACGATCTGGGGAACCGTTCGATCCAACTGCTCGTCACGCTCACGGGGAACCT
CGGCCGACAGGGAACGGGTCTCGACCACTACGTCGGCCAGGAGAAGATCTGGACGTTCAACGGGTGGAAGAAC
CTCTCGTTCCCGACCGGAAGCGTCCGGGGCGTCCCGACGACGCTCTGGACGTACTACCACGCCGGGATCATGG
AGAACACCGACCCGGACACGGCGGCGAAGATTCGGGAGTCGATCGACAAGGGGTGGATGCCGCTGTACCCGAG
CGAACGCGACGACAGCGGCCGCCCGGACCCCCGAGTCATGTTCGTCTGGCGCGGGAACTACTTCAATCAGGCC
AAGGGGAACATCGCGGTCGAAGAGGAGCTGTGGCCGAAGCTCGATTTGGTCGTCGACATCAACTTCCGGATGG
ACTCGACGGCGCTCAACAGCGATATCGTCCTGCCGACGGCGAGCCACTACGAGAAACACGACCTCTCGGAGAC
GGACATGCACACCTACGTGCACCCGTTCACGCCGGCGGTCGAGCCGCTGGGCGAGTCGAAGACCGACTGGCAG
ATCTTCCGCGATCTCGCCGCGAAGATACAGGAACTGGCCGCGGACCGCGGTACGGAGCCGGTCGACGATCGGA
AGTTCGACCGCCAGATCGACCTCCAGTCGGTCCACGACGACTACGTCCGCGACTGGCTCGACGACGAGCCCGG
TGCGCTGTCCGAGGACAAGGCGGCCGCGGAGTTCATCCTCGAGAACTCCGAGGAGACGAACCCCGAGGGGACC
GACGAGCAGATCACGTTCGACGAGATCGACGAACAGCCCAAACGGCTCCTCGAAACCGGCGACCACTGGTCGT
CGGACATCGAGGAGGGCGAGGCGTACACGCCGTGGAAAGACTACGTGCAGGACAAGAACCCGTGGCCGACGTT
CACGGGGCGACAGCAGTACTACATCGACCACGACTGGTTCTTGGAGCTCGACGAGCAGCTTCCGACCCACAAG
GAGGCGCCGGTGTTACAGGAGAAGTCGGAGTACCCGCTGGGGTACAACACGCCCCACAGCCGCTGGTCGATCC
ACTCGACGTGGCGCGACAGCAGCAAGATGCTCCGGCTGCAGCGCGGCGAGCCGACCGTCTACCTCAACCCCGA
CGACGCCGAGGAGCGGGGGATCGAGGACGGCGACACCGTGCGGGTGTACAACGACCTCGACTCGGTCGAGGTG
CAGGCGAAGATCTACCCGAGCGGGGAGCCCGGCACCGTCCGGCACTTCTTCAGCTGGGAGCGGTTCCAGTACC
CCGACCGCAACAACTTCAATTCGCTCGTCCCGATGTACATGAAGCCGACGCAGCTCGTCCAGTACCCCGAGGA
CACGGGCGAGCACCTCCACTTCTTCCCGAACTACTGGGGTCCGACCGGCGTGAACAGCGACGTTCGGATCGAG
GTCGAGAAGGTCGAAGAGGGGGCAGCGTCGCTCGACGCCGGTTTGCTCGGAGAGTCGGCGACGGAGTTGGAGT
CGCCCGATTCGGGCTCGAAATCGCGCGGCGCGTTGGGTCGCGTCTCGGACCTGCTCGGAGGTGACGACTAA
>hmu:Hmuk_2470-Ar DMSO reductase family type II enzyme, molybdopterin subunit; K00370 nitrate reductase 1, alpha subunit [EC:1.7.99.4] (N)
ATGAGTGAACACGACATCGACGACGACCGGTGGATGGACAGTTCCGGAATTACCCGACGCGACTTCGTCCGCG
GCCTCGGGGCCGCCTCGATCGTCGGCGCGACCGGCCTGTCGTTCGCCGACGAAGAGATGGACGGGCTGCAGGC
GGTCGACGATCCGATCGGATCGTACCCCTACCGCGAGTGGGAGGACCTCTACCGCGAGGAGTGGGACTGGGAC
TCCGTGGCGCGGTCGACCCACAGCGTCAACTGCACCGGCTCCTGTTCGTGGAACGTCTACGTCAAGGACGGGC
AGGTCTGGCGCGAGGAACAGGCCGGCGACTACCCCGTGATCGACGAGGACCTCCCAGATCCCAACCCGCGGGG
CTGCCAGAAAGGGGCCTGCTACACGGACTACGTCAACGCCGACCAGCGCGTGCTCCACCCGCTGCGCCGCACC
GGCGAACGCGGCGAGGGCCAGTGGGAGCGCATCTCCTGGGACGAGGCACTGACGGAGATCGCCGACCACGTCA
TCGACGAGGTGCAGGCGGGCCGGTACGACGCAATCTCGGGCTTTACGCCGATCCCGGCCATGTCGCCCGTGTC
GTTCGCCAGCGGCTCCCGGCTCGTGAACCTGCTGGGCGGCGTGTCCCACAGCTTCTACGACTGGTACTCCGAC
CTGCCGCCGGGTCAGCCGATCACCTGGGGGACCCAGACGGACAACGCCGAGTCCGCCGACTGGCACAACGCCG
ACTACATCATCGCCTGGGGGTCGAACATCAACGTCACCCGTATCCCCGACGCCAAGTACTTCCTCGACGCCGG
CTACGAGGGAGCGAAACGCGTCGGGATCTTCACCGACTACTCCCAGACCGCGATCCACACCGACGAGTGGCTC
GCGCCGGAGGGTGGCACCGACACCGCGCTCGCGCTCGGGATGGCCCAGACGATCGTCGACGAGGAGCTGTACG
ACGAGTCCCACCTCAAAGAGCAGACCGACATGCCGCTGCTCGTGCGAGAAGACACCGGGAAGTTCCTCCGGGC
GGGCGAGGTCGGTCTCGCGGCGGACGCCGACGACCCGGACAAGGTGTTCGTGATGGTCGACGAGGACGGGGAG
CTCCGGCCCGCGCCCGGTTCGCTCGGCGAGCGCGACGGCCAGCACGACCCCGAGTCGAGCATCGAACTCGACT
TCGACCCCCAGCTGGGCGTCGAGCGGAGCGTCGAGACCGACGACGGCGAGGTGGCGGTCCGGTCGGTCTGGGA
GAACCTCCGTGAGGAACTGTCACAGTACACGCCCGCGTTCGTCCACGAGGAGACCGGGGTCGGCGAGAACACC
TACCAGCGCGTCGCCCGAGAGTTCGCCGACGCCGACGCGGCCAAGATCATCCACGGCAAGGGCGTCAACGACT

GGTACCACAACGACCTGGGGAACCGGGCGATCCAGTTGCTCGTGACCCTGACCGGGAACCTCGGCGAACCCGG
CACCGGGCTGGATCACTACGTCGGCCAGGAGAAGATCTGGACGTTCCACGGCTGGAAGACGCTCTCGTTCCCG
ACCGGCAGCGTGCGGGGCGTGCCGACGACGCTGTGGACCTACTTCCACGCCGGCATCCTCGACAACACCGATC
CCGACACCGCCGAGAAGATCCGCGAGTCCATCGACGAGGGCTGGATGCCGGTGTACCCCGAGGAGCGCGAGGA
CGGGTCCTGGCCGGACCCCTCGACGATGTTCGTCTGGCGTGGCAACTACTTCAACCAGGCCAAGGGCAACGTC
GCCGTCGAGGAACAGCTCTGGCCGAAACTGGATCTGGTCGTGGACATCAACTTCCGGATGGACTCGACGGCGA
TGTACTCCGACATCGTCCTCCCGGCGGCGAGCCACTACGAGAAGCACGACCTGAACATGACCGACATGCACAC
GTACGTCCACCCGTTCACGCCCGCCGTCGAGCCGCTGGGCGAGGCCAAGTCTGACTGGGAGATCTTCCGCCTG
CTGGCCGAGAAGATCCAGGAGCGGGCCCAGGAACGGGGGGTCGAACCGGTCGAGGACCGATCGTTCGACCGCG
AGATCGACCTGACGACGATCTACGACGACTACGTGCGCGACTGGGAGACCGGCGAGGAGGGAGCCCTCGAAGA
CGGCCGCGAGGCCTCCGAGTTCGTCCTCGAACACAGCGAGGAGTCCAACCCCGCAGACAGCGACGAGCAGATC
ACGTTCGCCGACACCGTCGAGCAGCCCCAGCGTCTCGAAGCGGCGGGGGATCACTGGACCTCCGACATCGAGG
ACGGAGCACCGTACGTCCCCTGGCAGGACTACGTCCAGGACAAACAGCCCTGGCCCACCTTCACCGGCCGCCA
GCAGTACTACGTCGACCACGACTGGTTCCTCGAACTCGGCGAGGAGTTGCCCACGCACAAGGAGGGGCCACAG
GACACCGGCGGCGACTACCCCCTCTCCTACAACACGCCCCACAGCCGCTGGTCGATCCACTCGACGTGGCGCG
AGAACACGAAGATGCTCCGGCTCCAGCGGGGCGAACCGACGGTCTTCCTCAATCCCGAGGACGCCGAGGAGCG
CGGGATCGAGGACGGCGACACCGTCGAGGTGTACAACGACATGGGCAGCGTCGAGGTACAGGCGAAGATCTAC
CCGTCGGGTGACCCCGGAACGGTCCGGCACTTCTTCTCGTGGGAGAAGTTCCAGTATCCGGGCCGAGACAACT
TCAACACGCTCGTCCCGATGTACATGAAGCCCACGCAGCTGGTCCAGTACCCCGAGGACACCGGCGAACACCT
CTACTTCTTCCCGAACTACTGGGGTCCGACCGGCGTCAACAGCGACGTGAACGTGGACGTCCGACTGACCGAC
GGGCAGTCGGGCTCGGGAGACGAGCAGCGCGAGTCTGTCGGTGTGCGCCCCGCAGGAGGTGACGACCAATGA
>hut:Huta_0024-Ar DMSO reductase family type II enzyme, molybdopterin subunit; K00370 nitrate reductase 1, alpha subunit [EC:1.7.99.4] (N)
ATGAGTGATCCAACCCAATCCGACGACAGTACAGACGGCGTCTCACGTCGCGACTTCCTGCTGGGTGCCGGCG
CGGCCGGCGTGGTCGGCGCGACCGGCCTGACCGTCGCCGATCGCGCCCTCGACGGGCTCGAAACCGTCGACGA
TCCGATCGGCAATTATCCCTACCGCGACTGGGAGGACTTCTACCGTGAGGAGTGGGACTGGGATTCGGTCGCC
CGGTCGACGCACTCGGTCAACTGCACCGGCTCCTGTTCGTGGGACGTCTACGTCAAGAACGGCCAGGTCTGGC
GCGAACAACAGGCCAACGACTACCCGACCTTCGACGAGAGTCTCCCCGATCCCAACCCCCGGGGCTGCCAGAA
GGGGGCCTGTTACAACGACTACGTCGACGCCGAACAGCGTGTCCAGTACCCGATGCGTCGCACCGGCGAGCGC
GGGGCCGGCGAGTGGGAGCGCATCTCCTGGGACGAGGCGCTGACCGAGATCGCCGAGCACGTCATCGAGGAGA
TCCAGAACGGCCGCTACGACGCGATTTCCGGGTTCACGCCGATCCCCGCGATGAGTCCCGTGAGCTTCGCCTC
GGGGACGCGGCTGGTCAACCTGCTCGGCGGCGTCTCTCACTCCTTCTACGACTGGTATTCGGACCTCCCGCCG
GGCCAGCCGATCACCTGGGGCCATCAGACCGACAACGCCGAGAGCGCCGACTGGCACAACGCCGAGTACATCA
TCGCGTGGGGGTCAAACATCAACGTCACGCGCATCCCCGACGCGAAGTACTTCCTCGACGCGGGCTACGAGGG
CGCAAAGCGGGTCGGCGTCTTCTCCGATTACTCCCAGACCGCCATCCACACCGACGAGTGGATCGCGCCTGAT
CCCGGTACCGACACCGCGCTGGCGCTTGGGATGGCCCGCACCATCGTCGAGGAGGAGCTCTACGACGAGGCAC
ACCTCAAGGAACAGTCCGACATGCCACTGCTCGTTCGGAACGATACGGGCAAGTTCCTCCGGGCGAGCGAGGT
CCCCGGCCTCTCGGTTGCAGCCGACGAACCCGAGAAGGTCATGGTCATGCAGGACGGGGAGGGCAACCTGCGT
GCTGCGCCGGGGTCGCTCGGTGAACGCGAGGCCAAGTACGACGACTCCTTGTCGATCGAACTCGATTTCGACC
CGCAACTGGCCGTCGAGGACACCGTCGGGACGACCGACGGCGAGGTGGCCGTCACGTCGGTCTGGAACAACCT
CCGGGAGGAACTGGCCAACTACACGCCCGAGTACGTCGCCGACGAGACCGGCGTCGGGAAGGAGACTCACCAG
AAGATCGCCCGGGAGTTCGCCGACGTCGACCGCGGGAAGATCATCCACGGCAAGGGCGTCAACGACTGGTATC
ACAACGACCTGGGCAACCGTGCGATCCAGTTGCTCGTCACGTTGACGGGCCACATCGGGCGGAACGGCACCGG
CGTCGACCACTACGTCGGCCAGGAGAAGATCTGGACGTTCAACGGCTGGAAGACCCTCTCGTTCCCGACCGGT
TCCGTCCGGGGCGTCCCGACGACGTTGTGGACCTACTACCACGCGGGCATCCTGGAGAACACCGACGCGGAGA
CCCGCCGGAAGATCGAGGAGGCCGTCGAGAAGGACTGGATGCCCGTCTACCCCGAGGAGCGCGGGGACGGCAC
CCGACCCGATCCCTCGACGATGTTCGTCTGGCGGGGCAACTTCTTCAACCAGGCGAAGGGCAACGTCGCCGTC
GAGGAGGTCCTCTGGGACAAACTCGACCTCGTCGTGGACATCAACTTCCGGCTGGACTCGACAGCGCTGTACG
CCGACATCGTCCTGCCGGCCGCGAGCCACTACGAGAAACACGACCTCAACATGACGGACATGCACACCTACGT
GCATCCGTTTACGCCCGCGGTCGAACCGCTGGGTGAGTCCAAGTCCGACTGGCAGATCTTCCGGGAACTCGCG
GCGAAGATCCAGGAAATCGCCCGCGATCGGGACATCGACCCGATCGACGACCGGAAGTTCGACCGCCAGATCG
ACCTCCAGTCGGTCCACGACGACTACGTCCGGGACTGGGTGAGCGACGAAGACGGTGCCCTGGAAGAGGACCG
GGCGGCCTGCGAGGCCATCCTCGAACACTCCACGGAGACCAATCCGGACGACGGCGGCGAGATCACCTTCGCC
GACACCGTCGACCAGCCCCAGCGCTTCGAGGCCGCGGGCGACCACTGGACCTCCGACATCGAGGACGGCACGG

CCTACGCGCCCTGGAAGGACTTCGTCCAGGACAAGGAGCCCTGGCCAACCCTGACGGGTCGCCAGCAGTACTA
CATCGATCACGACTGGTTCCTCGATGTCGACGAGCAACTCCCGACGCACAAGCGCCCGGTCGAGACCAACGAT
CAGAGCGAGTACCCCCTGCGGTACAACACGCCCCACGGTCGGTGGTCGATCCACTCGACGTGGCGCGACAGCG
AAAAAATGTTGCAGTTGAACCGGGGTGAGCCGGTGGTCTTCATTCACCCCGAGGACGCAAAGCACCGCGGGAT
CGAGGACGGCGACACGGTCGAGATCTACAACGACCTGGCGACGATCGAGGCCAACGCCAAGCTCTACCCGGCC
AGCGAACCCGGGACCGTCCGGCATTACTTCGCCTGGGAGCGCTACCAGTACCCCAGCCGGAACAACTTCAACT
CGCTGATCCCGATGTACATGAAACCCACCCAGCTCGTCCAGTACCCCGAAGACTCGGGCGAGCACCTCCATTT
CTTCCCGAACTTCTGGGGCCCGACCGGGGTCAACAGCGACGTCCGCTGTGATATTAGGCCGAAAGAAGGGGGT
GACGACTGA

>mac_MA1205-Ar
TTGACAAGAAAAATTGCTTTTTACGGGAAAGGTGGTATTGGAAAATCCACCACTCAACAAAATACTGCAGCGG
CTATGGCATATTATCATGGCAAAAATGTTTTCATCCACGGGTGTGATCCCAAAGCAGACTGTACTCGTTTGGC
CCTTGGAGGCGTACCCCAGACCACAATAATGGATACGCTCAGGGAACTGGGAGAAGAAGCCGTAACAGTTGAT
AATGTAGTTAACACCGGTTTCAAAGGAATCAGATGTGTTGAATCCGGAGGTCCGGAACCGGGTGTTGGGTGTG
CTGGCAGGGGTGTAATCACTGCTATTAACCTTATGGAGGAGTTGGGAGCCTATTCCGATGACCTGGATTTTGT
TCACTTTGACGTGCTTGGCGACGTTGTCTGCGGCGGATTTGCAATGCCAATTCGGGAGGGTAAAGCTCAGGAA
GTATACATAGTCGCTTCGGGAGAAATGATGGCGACTTATGCAGCAAATAATATCTGCAAAGGTCTGCTGAAGT
ATGCAGAACAGAGCGGGGTGAGATTGGGTGGAATCATTTGTAACAGCCGTAAGGTCGATAATGAGCTGGAAAT
GATGGAGGAGTTTGTTTCCGCGCTTGGAACACAGCTGATACACTTTGTGCCAAGAGATAACATTGTTCAAAAG
GCGGAGTTTAATAAAAAGACAGTTGTGGAGTTCGACCCGGAATGCAACCAGGCAAAAGAATACGGAAAACTTG
CCAAGAAAATTCTTGAGAATGATATGTTCGTGATCCCCAAACCGTTAAGTATGGATCAACTGGAGAAAATGGT
GGCAAGATACGGTCTTATGGATTAA
>mac_MA1213-Ar
ATGACCAGAAAAATTGCTTTTTACGGGAAAGGTGGTATTGGAAAATCCACCACTCAACAAAATACTGCTGCAG
CTATGGCATATTATCATGGCAAAAATGTTTTCATCCACGGGTGTGATCCCAAAGCAGACTGTACTCGTCTGGC
ACTCGGGGGCGTACCCCAGACCACAATAATGGATACGCTCAGAGAGCTGGGTGAGGAGGCCGTAACAGTTGAT
AATGTAGTTAACACCGGTTTCAAAGGAATCAGATGTGTTGAATCCGGAGGTCCGGAACCGGGTGTTGGGTGTG
CTGGCAGGGGTGTAATCACCGCTATTAACCTCATGGAGGAGCTGGGAGCCTATTCCGATGACCTGGATTTTGT
TCACTTTGATGTGCTTGGCGATGTTGTCTGCGGCGGATTTGCAATGCCAATTCGGGAGGGTAAAGCTCAGGAA
GTATACATAGTCGCTTCGGGAGAAATGATGGCGACTTATGCAGCAAATAATATCTGCAAAGGTCTACTGAAGT
ATGCAGAACAGAGCGGGGTGAGATTGGGTGGAATCATTTGTAACAGCCGTAAGGTCGATAATGAGCTGGAAAT
GATGGAGGAGTTTGTTTCCGCGCTTGGAACACAGCTGATACACTTTGTGCCAAGAGATAACATTGTTCAAAAG
GCGGAGTTTAATAAAAAGACAGTTGTGGAGTTCGACCCGGAATGCAACCAGGCAAAAGAATACGGAAAACTTG
CCAAGAAAATTCTTGAGAATGATATGTTCGTGATCCCCAAACCGTTAAGTATGGATCAACTGGAGAAAATGGT
GGCAAGATACGGTCTTATGGATTAA
>mba_Mbar_A2281-Ar
ATGACAAGAAAAATTGCTTTCTATGGAAAAGGTGGAATCGGAAAGTCCACCACGCAACAAAATACTGCAGCTG
CTATGGCATATTATCATGGCAAAAAAATTTTCATTCACGGCTGTGACCCAAAAGCTGACTGTACTCGTCTTGT
GCTTGGTGGAGTAGCTCAAACTACGATAATGGATACTTTAAGGGAGCTTGGTGAGGAGGCTGTTACAGCTGAA
AATGTCATCAATACCGGCTTTGATGATATAAAATGCGTTGAGTCCGGTGGTCCAGAGCCCGGTGTTGGCTGTG
CTGGCCGAGGAGTTATCACTGCAATTAATCTCATGGAGGAAATGGGAGCCTATTCGGAGGACCTGGATTTTAT
TCACTTTGATGTATTGGGCGATGTTGTCTGCGGTGGTTTTGCAATGCCTATTCGGGAAGGAAAAGCCCAGGAA
GTATACATCGTTGCTTCTGGAGAAATGATGGCGACTTATGCAGCAAATAACATCTGTAAGGGCCTGTTGAAGT
ATGCCGAACAAAGCGGAGTGAGATTGGGAGGAATAATTTGCAACAGTCGTAGGGTGGATAATGAACTGGAAAT
GATGGAGGAATTTGCTTCTGCACTGGGAACGCAGCTGTTATACTTTGTACCACGTGACAACATTGTCCAAAAA
GCAGAGTTCAATAAAAAGACTGTAGTGGAGTACGACCCTACATGCAATCAAGCACTTGAATACAAAGAACTCG
CCAAAAAAATCCTAGAAAATGACATGTTTGTTATCCCTAAACCATTAAGTATGGATCAGCTAGAGAAAATGGT
TGAAAGATATGGTCTTATGGATTAA
>mae_Maeo_1434-Ar
ATGAGTTTCGACGAAATTGCTCCGGAAGCAAAAAAAATAGCAATATATGGAAAAGGAGGTATTGGAAAATCAA
CCACTACACAAAATATGGCAGCAGCTCTTGCTCACTTTTTAGGCCATAAAGTAATGATACACGGATGTGACCC
AAAAGCAGATTCAACAAGAATGATTCTCCACGGAATGGGGCAAAATACAGTAATGGATGTATTGAGGGATGAA

GGGGAAGATGCAATTACACTTGAAAAAGTTAGAAAACAAGGATTTGGAGGTATCTACTGTGTTGAGAGTGGAG
GTCCTGAGCCAGGAGTAGGATGTGCTGGTAGAGGAGTTATTACAGCTGTTGATACAATGAGAGAATTAGAAGG
ATATCCAGATGATTTGGATAATTTATTCTTCGATGTTCTTGGGGATGTTGTATGTGGTGGTTTCGCAATGCCA
CTTAGAGATGGATTAGCAGAAGAAATATATATCGTTACATCAGGGGAAATGATGGCATTATATGCTGCAAACA
ATATTGCAAAAGGTATTTTAAAGTATGCCGACCAGTCAGGAGTTAGATTAGGGGGAATTATCTGTAATTCAAG
AAAAGTAGATGGAGAAAAAGAATTAATGGAAGAATTTGTTGAAAAATTAGGAACAAAATTAATCCACTTTGTC
CCAAGAGACAATATCGTTCAGAAAGCAGAATTTAACAAACAAACAGTAATCGAATATGACCCAGAATGTAATC
AAGCAAAAGAATACAGAACCTTGGTCGAAAATATTACGGCAAATGAAGATTTAGTAAAACCTACGCCATTGTC
AATGGAGGAATTAGAGCAGTTAGTGGAAAAATACGGATTATTAGATTAA
>mok_Metok_1120-Ar
ATGAGTTTTGACGAAATAGCTCCAGATGCAAAAAAAATAGCAATTTATGGAAAAGGAGGTATAGGGAAATCGA
CAACAACGCAAAATACTGCCGCAGCTATGGCATATTACTTCAATAAAAAAGTGATGATTCATGGATGTGACCC
AAAAGCAGATTCAACTAGAATGATTCTTCATGGAAAACCCCAAAAAACCATCATGGATGTTCTTAGAGAAGAG
GGAGAAGAAGGAGTAACTCTTGAAAAGGTAAGAAAAGCAGGATTTAAAGATATATTATGTGTAGAAAGTGGTG
GTCCAGAACCTGGTGTAGGATGTGCAGGAAGAGGGGTTATTACTGCTGTGGATATGATGAGGGAGCTCGAAGG
ATATCCAGATGATTTAGATAATCTATTCTTTGATGTCCTTGGGGATGTTGTGTGTGGTGGATTTGCCATGCCA
CTTAGAGATGGTCTTGCACAGGAGGTATACATCGTTACATCGGGAGAGATGATGGCATTGTATGCTGCAAACA
ACATTGCAAAAGGTATATTAAAATATGCTGAACAGTCAGGCGTAAGATTGGGTGGGATTATCTGTAATTCAAG
AAATGTGGATGGAGAAAAAGAATTGATAGATGAATTTGTTGATAAATTGGGAACAAAATTAGTACATTTCGTT
CCAAGAGACAATATTGTCCAAAAGGCTGAATTCAATAAAATGACCGTTGTAGAATTTGACCCAGAATGCAATC
AAGCTAATGAATATAAAACACTTGCCAAAAACATTAATGAAAATGACGAACTTGTAATACCTACGCCATTAAC
AATGGATGAATTGGAAGGATTAGTATTAAAATATGGATTACTTGATTAA
>mfs_MFS40622_0031-Ar
ATGAGTGTTTATGATGAAATTGCACCAAATGCAAAAAAGGTTGCTATTTACGGAAAAGGAGGTATTGGTAAAT
CTACAACCACACAGAACACTGCTGCTGCATTAGCATACTATTACAAATTAAAGGGTATGATTCACGGATGTGA
CCCAAAGGCAGATTCAACAAGAATGATTTTACATGGAAAGCCGCAAGAGACCGTTATGGATGTTTTGAGAGAG
GAAGGAGAGGAGGGAGTTACCTTAGAAAAAATTAGGAAAGTTGGATTTGGAGGAATCTTGTGTGTTGAGTCAG
GAGGTCCTGAGCCAGGAGTTGGATGTGCTGGAAGAGGGGTTATTACAGCAGTTAATTTGATGATTGAACTTGG
AGGATACCCAGATGACTTAGACTTCTTGTTCTTTGACGTCCTTGGGGACGTTGTCTGTGGAGGATTTGCAATG
CCATTGAGAGATGGATTGGCAAAAGAGATTTACATTGTCTCTTCAGGAGAAATGATGGCTCTCTATGCAGCTA
ACAACATTGCAAGAGGTATCTTGAAGTATGCTGAACAGTCAGGGGTTAGATTAGGAGGAATTATTTGTAACTC
AAGAAAAGTCGATGGAGAAAAAGAATTGATGGAAGAGTTCTGCGATTTGTTGGGAACTAAGTTAATACACTTC
ATTCCAAGAGACAACATAGTCCAAAAAGCAGAGTTTAACAAGATGACTGTTGTTGAATTCGCTCCAGACCATC
CACAGGCACATGAATACAAGAAGTTAGGTAAGAAGATTATGGACAACGATGAATTGGTTATTCCAACACCATT
GTCAATGGACCAATTAGAAAAGTTAGTCGAGAAATATGGATTACTTGATAAATAA
>mvu_Metvu_1081-Ar
ATGAGTGTTTATGATGAGATTGCTCCAAATGCAAAGAAAGTTGCTATTTATGGAAAAGGAGGAATTGGTAAAT
CAACCACAACTCAAAATACTGCTGCTGCGTTGGCATACTACTACAATTTAAAAGGAATGATCCACGGATGTGA
TCCAAAAGCCGATTCAACGAGAATGATTTTACACGGTAAACCACAAGAGACGGTTATGGATGTGTTAAGGGAA
GAAGGGGAGGAAGGAGTTACGTTGGAAAAAGTCAGAAAAGTTGGATTTGGAGGAATTTTATGCGTAGAAAGTG
GAGGGCCTGAGCCAGGAGTTGGCTGTGCTGGAAGAGGAGTTATAACCGCAGTTAATATGATGATCGAACTTGG
AGGGTATCCTGATGATTTGGATTTTCTGTTCTTTGATGTATTGGGGGATGTTGTATGTGGAGGATTCGCAATG
CCACTAAGAGATGGGCTGGCAAAAGAGATCTATATTGTCTCTTCGGGAGAGATGATGGCTCTCTATGCAGCTA
ACAACATTGCGAGAGGTATCTTAAAATATGCTGAGCAATCAGGTGTTAGGTTGGGAGGAATTATCTGTAATTC
AAGAAAAGTTGATGGAGAAAAAGAGTTGATGGAAGAGTTTTGCGAAATGCTGGGAACCAAGTTAATACACTTT
ATTCCCAGAGACAATATTGTCCAAAAAGCTGAGTTTAATAAGATGACAGTTGTTGAATTCGCTCCAGATCATC
CCCAAGCACATGAATACAAAAAATTGGGGAAAAAAATTATGGATAATGATGAACTGGTGGTTCCAACTCCACT
TTCAATGGATCAACTGGAAAAACTCGTTGAAAAGTATGGATTGCTTGATTAA
>mig_Metig_1736-Ar
ATGAGTTTTGATGAAATTGCTCCAAACGCAAAAAAAGTGGCTATCTACGGAAAAGGAGGTATTGGTAAATCTA
CAACCACACAAAATACTGCTGCAGCATTGGCATACTATTACAAATTAAAAGGTATGATTCATGGATGTGACCC
AAAAGCAGACTCAACAAGAATGATATTACACGGAAAACCACAAGAAACCGTCATGGATGTTCTTAGAGAAGAA
GGAGAGGAAGGAGTTACATTAGAAAAAGTTAGAAAAGTTGGATTTGCAGGAATCTATTGTGTTGAGTCAGGAG
GTCCTGAGCCAGGAGTTGGATGCGCTGGAAGGGGAGTTATTACGGCAGTTAACTTAATGAGAGAACTCGGAGG

ATATCCAGATGATTTAGACTTCCTCTTCTTTGATGTCCTTGGGGACGTTGTCTGTGGTGGATTCGCAATGCCA
TTGAGAGATGGACTTGCTAAGGAAATCTATATCGTCTCATCTGGAGAGATGATGGCATTATATGCAGCAAACA
ACATTGCAAAAGGTATCTTAAAATATGCAGAACAATCTGGGGTTAGATTAGGAGGAATTATCTGTAACTCAAG
AAAGGTCGATGGAGAATTAGAATTGATGGAAGAATTCTGTGACAAATTAGGAACAAAATTAATCCACTTCATT
CCAAGAGACAACATTGTCCAAAAAGCAGAGTTCAACAAGATGACTGTTGTTGAATTCGCTCCAGATAGCAACC
AAGCAAAAGAATACAAAAAATTGGGTAAAAAAATCATGGATAACGATGAACTCGTAATTCCAACACCAATGAC
AATGGATGAAATGGAAAAATTAGTCGAAAAATACGGATTATATGACAAATAA
>mif_Metin_0041-Ar
ATGAGCTTTGACGAAATTGCCCCAAATGCTAAGAAAGTAGCAATTTACGGAAAAGGAGGGATAGGGAAGAGTA
CAACTACACAAAATACAGCAGCAGCTTTAGCATACTACTACAAATTAAAGGGAATGATTCACGGCTGTGATCC
TAAGGCGGATTCAACAAGAATGATATTACATGGAAAGCCCCAAGAGACAGTTATGGATGTTTTGAGAGAAGAA
GGAGAAGAAGGAGTTACCTTAGAAAAGGTTAGAAAAGTAGGATTCTGTGGAATTTATTGTGTTGAATCTGGAG
GACCAGAGCCAGGGGTTGGATGTGCAGGAAGAGGGGTTATAACTGCTGTCAATCTAATGAAAGAGCTTGGTGG
ATATCCAGATGATTTAGACTTTCTCTTCTTTGACGTCCTTGGGGATGTTGTCTGTGGAGGTTTTGCCATGCCT
CTAAGGGATGGGTTGGCTAAGGAAATTTACATAGTTTCCTCTGGAGAAATGATGGCACTTTACGCAGCTAACA
ACATTGCTAAGGGTATCTTAAAATATGCTGAGCAGTCAGGAGTTAGATTAGGAGGAATAATTTGTAACTCAAG
AAATGTTGATGGAGAAAGAGAGTTAATGGAAGAGTTCTGTGATAAGTTAGGAACTAAGCTTATTCACTTCATT
CCAAGAGACAATATAGTTCAGAAGGCTGAGTTTAACAAGATGACAGTAGTTGAATTTGCTCCAGATCATCCAC
AGGCTTTAGAGTACAAGAAGTTAGGAAAGAAGATTATGGACAATGATGAACTTGTAATTCCAACTCCATTAAG
CATGGATGAGTTGGAGAAGTTGGTTGAGAAGTATGGGTTATATGACAAATAA
>mel_Metbo_1819-Ar
ATGGTAAGAAAAAATTGCGATTTATGGAAAAGGTGGAATTGGAAAATCTACAACTACTCAGAACACAGCATCAG
CCATGGCTCATTTCCATGGAAAAAAAGTTATGATTCACGGATGCGACCCTAAAGCAGACAGTACAAGAATGAT
ACTTAGGGGTAAAATGCAAACCACCATGATGGACACACTTAGGGAAGAAGGCGAAGAAGCATGCATGAACCTG
GACAATGTTATGTCAACAGGTTTTGAAGGAATAAAATGTGTTGAATCTGGAGGTCCAGAACCAGGTGTAGGAT
GTGCTGGTCGTGGTGTAATAACAGCTATCACACTCATGGAACAACTCAAAGTCTACGATGACAACGACTTTGT
TTTCTTCGATGTTTTAGGTGACGTTGTTTGTGGAGGTTTTGCAATGCCAATCAGGGATGGTAAAGCCGAAGAA
ATATACGTTGTAGCATCTGGAGAAATGATGGCACTTTACGCAGCAAACAACCTGTGTAAAGGTATGGTAAAAT
ATGCTAATCAGAGTGGAGTGAGGCTTGGAGGAATCATCTGTAACAGCCGTAACGTGGACGGTGAAAAGGAACT
ACTGGAAGAATTCTGTAAAAAAATTGGAACTCAACTTATCTACTTCGTACCTAGGGACAACATGGTGCAAAAG
GCAGAGTTCAACAAAAAAACAGTTGTTGACTTCGATGCTGAATGTAACCAAGCACACGAATACGAAGCATTGG
CTGGAAAAATTATCAACAATGAAAACTTCGTGATTCCAAAACCAATGTCCATGGACGAACTGGAAGAAATGGT
ACTACAGTACGGTTTAACTGACTAA
>mew_MSWAN_2014-Ar
ATGGTAAGAAAAAATAGCAATTTATGGAAAAGGTGGAATAGGAAAATCAACAACTACTCAAAACACAGCATCAG
CAATGGCACATTTCCATGATAAAAAAGTCATGATACACGGCTGCGATCCAAAAGCAGACAGTACAAGAATGAT
ACTTGGGGGAAAAATGCAGAGAACCATGATGGACACCTTGAGAGAAGATGGAGAAGAAGCCTGTATGGTACTG
GATAACATCATGTCAATAGGTTACGAAGGAATAAAATGTGTAGAATCCGGTGGTCCTGAACCTGGTGTAGGAT
GTGCTGGTAGGGGTGTGATAACAGCAATTACTCTCATGGAACAGCAGAAAGTCTACGAAGATAACGATTTTGT
TTTCTTTGATGTACTTGGAGATGTTGTGTGCGGTGGATTTGCAATGCCTATCAGGGACGGTAAAGCTGAAGAA
ATCTACGTTGTGGCTTCAGGAGAGATGATGGCACTCTACGCAGCAAACAACCTGTGTAAAGGTATGGTGAAAT
ACGCTAACCAGAGTGGTGTGAGACTTGGAGGAATAATCTGTAACAGTCGAAACGTTGATGGAGAAAGAGAACT
CCTTGAAGAATTCTGTAAAAAAATAGGAACCCAAATGATCCATTTCGTTCCAAGGGACAACATAGTCCAGAAA
GCAGAGTTCAACAAAAAAACCGTGGTAGACTTTGAACCAGACTGTAACCAGGCCCATGAATACGAAGCACTTG
CAGGTAAAATAATCAACAACGAAAATTTCGTCATTCCAAAACCAATGTCCATGGATGAGCTTGAAGAGATGGT
TGTTCAGTACGGTTTAATGGACTAG
>mmp_MMP0853-Ar
ATGGTAAGAAAAAATCGCAATTTACGGAAAAGGTGGAATTGGAAAATCAACCACCACACAAAACACAGTAGCTG
CAATGGCTCACTTTCACGACAAAAAAGTATTCATTCACGGATGTGACCCAAAAGCAGACAGTACAAGGTTAAT
CTTGCACGGTAAACAGCAAGTAACAATGATGGATACATTAAGAGAAAAAGGGGAAGACGAATGTACTCCTGAC
AAAGTTATCGAAGTTGGTTTCGGTGGCGTAAAATGTGTAGAATCAGGAGGACCTGAGCCAGGAGTAGGCTGTG
CAGGTAGAGGTGTTATCACTGCAATTACACTCATGGAACAGCACGGTGTTTACGAAGACGACCTTGACTTTGT
ATTCTTCGATGTTTTAGGGGACGTTGTATGTGGTGGATTCGCAATGCCTGTAAGAGACGGTAAAGCAGACGAA
ATCTACGTTGTAGCATCTGGAGAAATGATGGCTCTTTACGCTGCAAACAACATCTGTAAAGGAATGGTTAAAT

ACGCAGAACAAAGCGGTGTAAGACTTGGAGGAATCATCTGTAACAGTAGAAACGTAGATGGTGAGCTTGACTT
ATTACAAGAATTCTGTGACAAAATTGGAACACAATTAATCCACTTTGTTCCAAGAGACAACATCGTTCAAAAA
GCAGAATTCCAGAAAAAAGCTGTTGTTGACTACGACGATACATGCAACCAAGCACTGGAATACAAAGAACTCG
CAAGAAAAATCATTGAAAACGAAAACCTCGTAATTCCAACCCCAATGACAATGGACGAATTAGAAGAATTAAC
TTCAAAATACGGATTCTTAGATTAA
>mmx_MmarC6_1803-Ar
ATGGTAAGAAAAAATCGCAATTTACGGAAAAGGTGGAATCGGAAAATCAACCACCACACAAAACACAGTAGCTG
CAATGGCTCACTTTCACGACAAAAAAGTATTCATTCACGGATGTGACCCTAAAGCAGACAGTACAAGGTTAAT
CTTGCACGGTAAACAGCAAGTAACAATGATGGATACATTAAGAGAAAAAGGAGAAGACGAATGTACTCCTGAC
AAAGTTATCGAAGTTGGTTTCGGTGGCGTAAAATGTGTAGAATCAGGAGGACCTGAGCCAGGAGTAGGCTGTG
CAGGTAGAGGTGTTATCACTGCAATTACACTCATGGAACAACACGGTGTTTACGAAGACAACCTTGACTTTGT
ATTCTTCGATGTTTTAGGGGACGTTGTATGTGGTGGATTCGCAATGCCTGTAAGGGATGGTAAAGCAGACGAA
ATCTACGTTGTAGCATCAGGAGAAATGATGGCTCTTTACGCTGCAAACAACATCTGTAAAGGAATGGTTAAAT
ATGCAGAACAAAGCGGTGTAAGACTTGGAGGAATCATCTGTAACAGTAGAAACGTAGATGGTGAGCTTGAGTT
ATTACAAGAATTCTGTGACAAAATTGGAACACAATTAATCCACTTCGTTCCAAGAGACAACATCGTTCAAAAA
GCGGAATTCCAGAAAAAAGCTGTTGTTGACTTCGACGACACATGCAACCAAGCAATGGAATACAAAGAACTCG
CAAGAAAAATCATTGAAAACACAAACCTCGTAATCCCAACCCCAATGACAATGGACGAATTAGAAGAATTAAC
TTCAAAATACGGTTTCTTAGATTAA
>mmq_MmarC5_0658-Ar
ATGGTAAGAAAAAATCGCAATTTACGGAAAAGGTGGAATCGGAAAATCAACCACCACACAAAACACAGTAGCTG
CAATGGCTCACTTTCACGACAAAAAAGTATTCATTCACGGATGTGACCCAAAAGCAGACAGTACAAGGTTAAT
CTTGCACGGTAAACAGCAAGTAACAATGATGGATACATTAAGAGAAAAAGGAGAAGACGAATGTACGCCTGAC
AAAGTTATCGAAGTTGGTTTCGGTGGTGTAAAATGTGTAGAATCAGGTGGGCCAGAGCCTGGAGTAGGCTGTG
CAGGTAGAGGTGTTATCACTGCAATTACACTCATGGAACAACACGGTGTTTACGAAGACGACCTTGACTTCGT
ATTCTTCGATGTTTTGGGGGACGTTGTATGTGGTGGATTCGCAATGCCTGTAAGAGATGGTAAAGCAGACGAA
ATCTACGTTGTAGCATCAGGGGAAATGATGGCTCTTTACGCTGCAAACAACATCTGTAAAGGAATGGTTAAAT
ACGCAGAACAAAGCGGTGTAAGACTTGGAGGAATTATCTGTAACAGTAGAAATGTAGATGGTGAGCTTGACTT
ATTACAAGAATTCTGTGACAAAATTGGAACACAATTAATCCACTTCGTTCCAAGAGACAACATCGTTCAAAAA
GCAGAGTTCCAGAAAAAAGCTGTTGTTGACTACGACGACACATGTAACCAAGCAATGGAATACAAAGAACTTG
CAAGAAAAATCATTGAAAACGAAAACCTCGTAATTCCAACCCCAATGACAATGGACGAATTAGAAGAATTAAC
CTCAAAATACGGATTCTTAGATTAA
>mmz_MmarC7_0098-Ar
ATGGTAAGAAAAAATCGCAATTTACGGAAAAGGTGGAATTGGAAAATCAACCACCACACAAAACACAGTAGCTG
CAATGGCTTACTTTCACGACAAAAAAGTTATGATCCACGGATGTGACCCAAAAGCAGACAGTACAAGGTTAAT
CTTGCACGGTAAACAGCAAACAACAATGATGGATACATTAAGAGAAAAAGGGGAAGAAGAATGTACTCCTGAA
AAAGTTATCGACGTCGGATTCTGTGGCGTAAAATGTGTAGAATCAGGAGGACCTGAGCCAGGAGTGGGATGTG
CAGGTAGAGGTGTTATCACTGCAATTACACTCATGGAACAACAAGGTGTTTACGAAGACAACCTTGACTACGT
ATTCTTCGATGTTTTAGGGGACGTTGTATGCGGTGGATTCGCAATGCCTGTAAGAGATGGTAAAGCAGACGAA
ATCTACGTTGTAGCATCAGGAGAAATGATGGCTCTTTACGCTGCAAACAACATTTGTAAAGGAATGGTTAAAT
ACGCAGAACAAAGTGGTGTAAGGCTTGGAGGAATCATCTGTAACAGTAGAAACGTAGATGGTGAACTTGAATT
ATTACAAGAATTCTGTGACAAAATTGGAACACAATTAATCCACTTCGTTCCAAGAGACAACATCGTTCAAAAA
GCAGAGTTCCAGAAAATGGCTGTTGTTGACTTCGATGACTCATGTAACCAAGCATTGGAATACAAAGCACTTG
CACAAAAAATCATTGACAACAAAAACCTTGTAATTCCAAACCCAATGACAATGGACGAATTAGAAGAATTAAC
TTCAAAATACGGTTTCTTAGATTAA
>mvn_Mevan_0065-Ar
ATGGTAAGAAAAAATCGCAATTTATGGAAAAGGTGGGATTGGGAAGTCAACAACCACCCAAAATACGGTAGCAG
CAATGGCATATTTTCACGATAAAAAGGTATTTATCCACGGCTGTGACCCGAAAGCAGACAGTACAAGGTTAAT
CTTGCACGGAAAACAACAAACAACAATGATGGACACATTAAGGGAAAAAGGGGAAGACGAATGTACTCCTGAT
AAAGTTATTGAAACCGGATTTTGTGGAGTCAGGTGTGTAGAATCAGGTGGGCCGGAACCAGGTGTGGGGTGTG
CAGGTAGAGGCGTTATAACCGCAATTACACTCATGGAACAACACGGTGTTTACGAAGACAACCTTGACTTCGT
ATTCTTCGATGTTTTAGGGGACGTTGTATGCGGTGGATTTGCAATGCCTGTAAGGGACGGTAAAGCAGACGAA
ATCTACGTTGTAGCATCGGGAGAAATGATGGCTCTTTACGCTGCAAACAACATCTGTAAGGGAATGGTTAAAT
ACGCACAACAAAGCGGTGTAAGACTCGGTGGTATCATCTGTAACAGCAGAAACGTGGATGGTGAACTTGAATT
ATTACAAGAATTCTGTGATAAACTCGGAACACAATTAATCCACTTCGTTCCAAGGGACAACATCGTTCAAAAA

GCAGAGTTCCAGAAAAAAGCCGTAGTTGACTTCAACGACACATGCAACCAAGCAATGGAATACAAAGAACTCG
CAAGAAAAATCATCGAGAACAAAAACCTCGTAATCCCAACACCAATGACAATGGACGAATTAGAAGAATTAAC
TTCAAAATACGGTTTCTTAGATTAA
>mmg_MTBMA_c01460-Ar
ATGGTACGTAAAATTGCAATATATGGTAAGGGTGGAATCGGAAAATCCACAACACAGCAGAACACTGCAGCGG
CAATGAGCTACTTCCATGGAAAAAACGTGATGATCCATGGATGCGACCCCAAGGCAGACAGCACACGCCTTAT
ACTGGGCGGTAAAATGCAGACAACCATGATGGACACCCTGCGTGAACTGGGAGAAGGTGCATGCACACCAGAC
AAGGTTATAGAAACAGGATTTGGAGGTATAAGGTGTGTTGAATCAGGAGGTCCAGAACCGGGTGTCGGATGCG
CAGGCAGGGGTGTTATAACCGCCATAACCCTCATGGAGAGGCATGGGGTCTATGAAAACGACCTGGACTTTGT
ATTCTTTGATGTGCTCGGTGACGTGGTATGCGGGGGGTTTGCAATGCCGGTGAGAGACGGAAAGGCGGAGGAA
ATATACATCGTGGCATCAGGTGAGATGATGGCACTCTATGCCGCCAACAACATCTGCAGGGGTATGGTGAAAT
ATGCACGGCAGAGTGGCGTCAGACTTGGGGGGATAATATGCAACAGCAGAAACGTTGATGGCGAAAGGGAACT
CCTTGAAGAATTCTGTGAAAGAATAGGGACACAGATGATACACTTCGTGCCAAGGGATAACATCGTACAGAAG
GCAGAATTCAACAAAAAATCTGTCATAGAGTTCGACCCCGAGTGCAACCAGTCACAGGAGTACAGGGAACTTG
CAAGGAAAATAATTGAAAACACAGATTTTGTAATACCAGAACCCATGACAATGGATGAAATGGAGGACCTCGT
TGTAAAATACGGGGTTCTGGACTAG
>mth_MTH1560-Ar
ATGTTTCAACAATTGAAAATTGTCGGAAGGCAGAAGCATACTTCCGGCAATGTTGAAGATGGTGAATGGTGCA
TGGTAAGAAAAATCGCGATATATGGAAAGGGTGGAATCGGAAAATCCACAACACAGCAAAACACTGCAGCTGC
AATGAGCTACTTCCATGGTAAAAATGTGATGATCCATGGATGTGACCCAAAGGCAGATAGCACACGCCTGATA
CTTGGAGGTAAAATGCAGACAACAATGATGGATACCCTCCGTGAACTTGGAGAGGTCGCATGTACACCGGATA
AGGTTATTGAAACAGGCTTTGGGGGAATAAAATGTGTTGAATCAGGAGGTCCTGAACCCGGCGTAGGATGCGC
TGGCAGGGGGGTCATAACTGCAATAACCCTCATGGAAAGACATGGAGTCTATGAGAAGGACCTGGACTTTGTA
TTTTTTGATGTACTGGGTGACGTTGTGTGTGGGGGCTTTGCAATGCCTGTTAGGGATGGAAAGCGGAAGAGA
TATACATAGTGGCATCTGGGGAGATGATGGCACTCTATGCAGCCAATAACATCTGTAAAGGGATGGTTAAATA
TGCCAGGCAGAGCGGAGTGCGTCTCGGGGGCATAATATGCAACAGCAGAAACGTTGATGGGGAGAAAGAGCTT
CTTGAAGAATTCTGTGAAAGAATAGGTACACAGATGATACACTTTGTTCCAAGGGACAACATAGTACAGAAGG
CTGAATTCAACAAAAAATCGGTCATAGAATTCGATCCAGAGTGTAACCAGTCCCAGGAGTACAGGGAACTCGC
AAGGAAAATAATTGAAAACAAGGACTTCGTGATACCTGAACCAATGACAATGGATGAAATGGAGGAACTCGTT
GTAAAATACGGAGTTATGGATTAG
>mac_MA3895-Ar
ATGAGACAAATAGCAATTTATGGAAAGGGCGGCATTGGAAAATCAACTACTACGCAGAATCTTACTGCGGCCC
TCTCGACTATGGGGAACAATATTCTTCTTGTAGGGTGCGACCCAAAAGCAGACTCTACCAGAATGCTGCTTGG
AGGCCTGAACCAGAAGACCGTACTTGACACTCTTAGAAGCGAAGGTGACGAAGGAATCGACCTTGATACTGTT
CTGCAGCCAGGCTTTGGAGGCATCAAATGTGTAGAATCCGGGGGTCCTGAACCGGGGGTAGGATGCGCAGGCC
GAGGTATTATCACCTCCATCGGACTGCTCGAAAACCTGGGAGCTTACACTGACGATCTTGACTATGTATTCTA
CGACGTGCTTGGTGACGTTGTATGCGGTGGTTTTGCCATGCCTATCCGCGAAGGAAAAGCCAAGGAAATTTAC
ATCGTGGCCAGTGGAGAACTGATGGCAATTTATGCAGCAAACAATATCTGCAAAGGTCTGGCCAAATTCGCTA
AAGGTGGAGCCCGTCTCGGAGGCATCATCTGTAACAGCAGGAAAGTAGATGGAGAACGTGAACTCCTTGAAGC
ATTCGCAAAGAAACTGGGAAGTCACCTGATCCACTTCGTGCCCAGAGACAACATCGTCCAGAGGGCAGAAATC
AACAGAAAGACAGTAATCGACTTTGATCGAGAATCCGACCAGGCAAAGGAATACCTCACCCTTGCAGACAACG
TCCAGAACAACAACAAACTCGTGGTTCCAACCCCACTCCCGATGGAAGAGTTAGAAGCTATGATGGTAGAATT
CGGAATCGTTGAGCTGTAA
>mma_MM_0719-Ar
ATGAGACAAATAGCAATCTACGGAAAGGGCGGTATAGGAAAATCCACTACCACGCAGAATCTTACTGCAGCCC
TTGCGACTATGGGAAACAAAATTCTCCTTGTGGGATGTGACCCCAAAGCAGACTCTACCAGAATGCTGCTTGG
CGGCCTGAACCAGAAAACCGTGCTTGACACACTCAGAAGTGAAGGAGACGAAGGAGTAAACCTTGACGTCGTT
GTACAGCCAGGTTTTGGGAATATTAAATGTGTGGAATCAGGAGGGCCCGAGCCCGGTGTAGGATGTGCAGGCA
GGGGCATTATTACTTCCATAGGGCTGCTCGAAAACCTGGGAGCCTACACTGAAGACCTTGACTATGTATTCTA
CGACGTGCTTGGTGACGTCGTGTGCGGAGGTTTTGCCATGCCAATCCGTGAAGGAAAAGCCAAGGAGATCTAC
ATTGTTGCCAGTGGGGAACTGATGGCAATTTACGCAGCAAACAACATCTGTAAAGGACTTGCCAAGTTTGCCA
AAGGTGGAGCCCGTCTTGGAGGCATCATCTGTAACAGCAGAAACGTAGACGGAGAACGTGAACTTCTTGAGGC
ATTCGCGAAGAAACTGGGAAGCCAGCTAATCCATTTCGTGCCCAGGGACAACATCGTTCAGAGGGCGGAAATC
AACAGAAAAACCGTGATCGACTTTGACCCAGAATCCAATCAGGCAAAAGAATACCTGACTCTCGCCAGCAACG

```
TCCAGAACAACACCAAACTAGTGGTCCCGACTCCGCTCCCTATGGAAGAGTTAGAAGCCATGATGGTGGAATT
CGGGATTGTTGAGCTATAA
>mba_Mbar_A0171-Ar
ATGAGACAGATAGCAATCTACGGAAAAGGTGGAATTGGAAAATCCACTACTACTCAGAATCTGACCGCGTCCC
TCTCGACCATGGGCAACAAAATTATGCTTGTAGGATGTGACCCGAAGGCAGATTCTACTAGAATGCTGCTTGG
TGGCCTGAACCAGAAGACCGTACTTGACACTCTTAGAAGTGAAGGCGATGAAGGAGTTGATCTTGATGTTGTT
ATGCAGCGGGGATTTGGCGACATAAAATGTGTGGAATCCGGAGGGCCTGAACCAGGTGTAGGATGTGCAGGCA
GAGGAATTATCACTTCCATCGGGCTGCTTGAAAACCTTGGAGCATATACCGATGATCTTGATTATGTATTCTA
CGACGTACTTGGTGATGTTGTGTGTGGTGGATTTGCTATGCCTATCCGCGAAGGAAAAGCAAAAGAGATTTAC
ATAGTTGCCAGTGGAGAACTGATGGCTATCTATGCTGCAAATAACATCTGTAAAGGACTTGCTAAGTTTGCCA
AAGGTGGAGCCCGTCTGGGAGGTATCATCTGCAACAGTAGAAATGTCGACGGAGAGCGTGAACTTCTCGACGC
TTTTGCAAAAAAACTGGGAAGTCAGCTAATTCACTTCATACCCAGGGACAACATTGTTCAGAGAGCAGAAATT
AACAGGAAGACTGTAATTGATTTTGACCCAGAATCCAACCAGGCAAAAGAGTACCTGACTCTTGCCCACAACG
TCCAGAACAACGACAAACTTGTTGTCCCAACTCCGCTTCCAATGGAAGAGTTAGAATCAATGATGGTTGAATT
CGGAATTGTTGATATGTGA
>mbn_Mboo_1142-Ar
ATGACACGACAGGTGGCAATTTACGGAAAAGGCGGTATCGGAAAATCGACAACAACCCAGAACACGGTGGCGG
CGCTTGCCGAGGCCGGAAAGAAGATCATGGTGGTCGGCTGCGACCCGAAAGCGGATTCAACCCGGCTCCTGCT
GCACGGGCTCTGCCAGAAGACGGTGCTTGATACGCTCCGTGATGAAGGGGACGATATCGAGCTTGACGCAATC
CTGAAACCCGGCTTTAAGGAAACAATGTGCGTGGAATCGGGAGGCCCCGAGCCCGGCGTCGGCTGTGCGGGAC
GCGGTATTATTACCTCGATCAACCTGCTCGAATCGCTGGGAGCGTACACGCCCGATCTCGACTACGTGTTCTA
CGATGTGCTCGGTGATGTGGTCTGCGGGGGTTTTGCCATGCCGATCCGTGAGGGCAAGGCCCAGGAGATCTAC
ATTGTCGCATCCGGGGAACTCATGGCCCTCTATGCGGCAAACAACATCTCCAAAGGTATCCAGAAATACGCGG
TAAACGGCAAAGTCCGGCTTGGCGGGATTATCTGCAACAGCCGGAAGGTCGATAACGAGCGTGCACTGCTCCA
GGCGTTTGCCGAAGAACTCGGGTCGCAGTTGATCTACTTTGTCCCGCGTGACAACCTTGTCCAGCGTGCCGAG
ATCAACAAGAAAACCGTTGTGGACTTCGATCCGACATCCGGCCAGGCCAGCGAATACCGCAGCCTTGCCGAGG
CTATCGACAAAAACAAGATGTTTGTTGTGCCAAAGCCCATGACCCAGGATCGGCTTGAGGAACTCATGATGCA
ACACGGGTTCCTGGATGCAATTGCCTAA
>rci_RCIX1606-Ar
ATGCGACAAGTGGCAATTTACGGAAAAGGCGGCATAGGCAAGTCTACCACGACGCAGAATACGGTAGCTGCGC
TGGCCGAGTCGGGCAAGAAGGTCATGGTCGTAGGCTGCGACCCGAAAGCCGATTCCACCAGGCTGCTGCTCCA
CGGGCTGAACCAGAAGACGGTGCTGGACACCCTGCGGGACGAGGGCGACGACATAGAGCTCGAGTCCATCCTC
AAGACGGGCTTCGGCGAAACCCGGTGTGTGGAGTCGGGCGGCCCCGAGCCGGGAGTGGGCTGCGCTGGCCGTG
GCATCATCACCTCGATCAACATGCTGGAGCAGCTCGGCGCTTACACCGACGACCTGGACTACGTCTTCTACGA
TGTGCTGGGCGACGTCGTCTGCGGTGGTTTCGCGATGCCCATTCGCGAGGGTAAGGCCCGGGAGATCTACATC
GTGGCGTCGGGCGAGCTGATGGCCCTCTACGCGGCGAACAACATCGCCAAAGGCATAAAGAAGTACGCCGAGA
CCGGCGGAGTCAGGCTGGGCGGCATCATCTGCAACAGCAGGAAGGCGGACAACGAGTACGCCCTGGTCAAGGC
GGTCGCCGAAGAGATAGGCACCCAGATGATCCACTTCGTCCCGAGGGACAACATCGTCCAGCGGGCGGAGATC
AACAAGATGACGGTCATCGACTTCGACCCCGCTGCAAACCAGGCGAACGAGTACCGGAAGCTCGCGAAGGCGA
TCGACGAGAACCAGATGTTCGTCGTCCCGAAGCCCATGACCCAGGACAGGCTGGAGGAGCTGATGATGAAACA
CGGGTTCCTGGACGCGGTGTGA
>mpl_Mpal_0561-Ar
ATGAAAAGACAGGTAGCAATCTACGGAAAAGGCGGAATCGGAAAGTCGACAACAACCCAGAACACTGTCGCGG
CACTGGCTGAACAGGGGAAGAAGATCATGGTGGTTGGATGCGACCCCAAAGCCGACTCCACCAGACTTCTGCT
CCACGGACTCTGTCAGAAGACAGTGCTGGACACCCTGCGGGACGAAGGAGACGATATCGAACTGGACGAGATC
CTCAAACCGGGATATCGGGACACCCTCTGTGTTGAATCAGGAGGCCCGGAGCCTGGCGTTGGATGTGCCGGCC
GTGGTATCATCACGTCGATCAACCTGCTGGAGTCACTCGGGGCATATACCGACGACCTGGACTATGTCTTCTA
CGATGTGCTTGGGGATGTCGTCTGCGGGGGGTTCGCGATGCCAATCAGGGAAGGAAAGGCAGAGGAGATCTAT
ATCGTCGCCTCCGGGGAGTTGATGGCGCTGTATGCGGCCAACAACATCGCCAAGGGGATCCATAAGTACGCCG
TATCAGGCAAGGTCAGGCTGGGAGGAATCATCTGCAACAGCCGACAGGTCGACAATGAGTATCCCCTCCTCAA
GGCATTTGCCGAGGAACTTGGATCCCAGTTGATCTACTTTGTCCCACGGGACAATCTGGTGCAGCGGGCTGAG
ATCAATAAGAAGACGGTGATCGACTTCGAACCGGAGTCCAACCAAGCTGAGGAATACAGGCAGCTGGCCAAGG
CCATTGATGAGAACAGGATGTTTGTCATCCCGAAACCGATGACACAGGACCGGCTGGAAGAACTGATGATGCA
GCATGGATTCATGGATGCACTTGTGTAA
```

>mpi_Mpet_0263-Ar
ATGCGACAGATAGCAATTTACGGAAAGGGTGGTATCGGAAAGTCGACTACCACACAGAACACAGTAGCGGCAC
TTGCAGCTTCAGGAAAGAAGGTCATGGTAGTCGGGTGCGACCCGAAGGCCGATTCAACGAGGCTTTTGCTCCA
CGGACTATGCCAGAAGACGGTGCTTGACACACTAAGGGATGAAGGAGACGACATAGACCTTGAGGATGTCCTC
AAGCCCGGTTTCGGAGAGACTATGTGCGTTGAATCCGGAGGTCCTGAACCTGGTGTAGGCTGTGCAGGAAGGG
GTATCATCACATCGATCAACCTCCTTGAATCACTCGGTGCATATACCGACGATCTCGACTACGTCTTCTACGA
TGTCCTTGGTGATGTCGTATGCGGAGGATTCGCAATGCCTATCCGTGAAGGAAAGGCTCAGGAGATCTATATC
GTTGCCTCAGGAGAACTGATGGCACTCTACGCTGCAAACAACATCTCCAAGGGTATCCAGAAGTATGCCGAAA
CAGGCGGTGTAAGGCTCGGGGGAATCATATGCAACAGCCGTAAGGTCGACCACGAACTCGAACTCCTCCAGGC
ATTTGCAACAGAACTCGGCTCGCAGCTCATCTACTTCGTTCCCCGTGACAACCTCGTCCAGAGGGCGGAGATC
AACAAGAAGACAGTCGTCGATTTCGATCCCGAATCCAACCAGGCAAACGAGTACCGCAGCCTTGCAGCTGCAA
TCGACGGCAACAAGATGTTCGTCATCCCGAAGCCCATGAGCCAGGATCGCCTCGAAGAACTAATGATGGAGCA
CGGTTTCCTCGGACTCTGA
>mka_MK1416-Ar
TTGAAAAGGATCGCCGTCTACGGGAAGGGCGGGATAGGGAAATCCACGATCGCGGCGAACGTGGCCGCAGCCC
TGGCCGAGGAAGGTTATCGGGTCATGCTGGTAGGATGTGATCCAAAGGCGGACTCGACCCTGACGCTCGCTGG
ACGTAGGATTCCCACGGTGATGCACGAGTATCGGAGGAAAGGGGAAGGCCTGAAGCTCGAGGACATCATCGTC
GAAGGTGATTTCGGGGTCTTGTGCGTCGAGTCCGGTGGGCCGAAGCCCGGGGTCGGATGCGCGGGGCGCGGTG
TGCTGAAGGCCCTCGAGATGTTGACTAGAATGGGGGCGTTCGAGGACGTCGACGTGGTGATCTTCGACGTGCT
CGGTGACGTGGTGTGCGGTGGATTCGCCCTACCGATCCGCCGAGGCTACGCGGATACCGTTTTCGTGGTTACA
TCCAGTGAACCGATGTCCCTATACGCCGCCAACAACATCTGCCGCGGGATCGCCGAGTACGCCGATCGAGGGG
GCGCGAAACTCGGTGGGGTTATCCATAACCGGCGCTCCCGAGACTCGGATTCTAGAGTGGTGACCGAGTTCTG
CCGGCGAATCCGAGCCGAGCTGATTTACGATCTGTTTTACATGGAAGAGGTGCGGAAGGCTGAGTCGCGGTAC
CGTACCGTGATCAGGGAGTTTCCGGATTCGGACGCGGCAGAGGCGTTCCGCGAGCTTGCGCACAGGATGCTCG
AGACGGAAGGTGTCGTACCTCAACCGCTCGAAGAAGAGGAGGTGCTGAGACTCGCGGGCGTGCGGTTCTGA
>mcj_MCON_0607-Ar
ATGAGGCAGATAGCCATTTACGGCAAAGGCGGAATCGGCAAGTCGACCACCACCCAAAATCTCACAGCAGCCC
TCTCGACCATGGGCAAGAAGATCATGCAGATAGGCTGCGACCCCAAAGCAGACTCCGTGAAGATGCTCATGAA
CGGCAAGAGACAGCCATCAGTTCTTGACACCCTTCGAAAGGAAGGAGAAGGAGAATTGGATGACGTACTGAAG
ACCGGCTTTGGAGGCATTCGCTGCGTCGAGTCCGGAGGCCCGGAACCGGGCGTGGGCTGTGCAGGTCGCGGGA
TAATCACCTCCATCGGCCTCCTTGAGAACCTGGGAGCCTACACCCCAGACCTCGACTATGTCTTCTACGACGT
CCTGGGAGACGTCGTCTGCGGCGGCTTCGCTATGCCCATCCGGGAAGGAAAGGCCCAGGAGATCTACATCGTG
GCCTCCGGAGAGATGATGGCTCTTTATGCCGCCAACAACATCTGCAAGGGCATCCAGAAGTTCGCCGAGTCCG
GCGGAACGCGTCTGGGCGGCATCATCTGCAACTCCAGAAACGTCGACCGGGAACGGGAGCTGGTAAGCGCTTT
TGCCGAGAAGATCGGCAGCCAGATGATCCAGTTCGTGCCCAGAGACAACATCGTCCACCAGTCAGAGATCCAC
AAGCAGACAGTGATCCAATATGCTCCAGACTCAGACCAGGCCAAAGCCTATTTGGCGCTGGCCAAGGCCGTGG
ACGAGAACCAGATGTTCGTTATCCCAAAGCCGATGGAGTATGAAGAGCTGGAACAGGTCATGATGGAATGGGG
TGTTGTGGAATGA
>mig_Metig_1207-Ar
ATGAAGCAAATAGCATTTTATGGGAAAGGAGGAATTGGAAAATCAACAACTGTCTGTAATATAGCGGCGGCAT
TGGCAGATGAAGGGAAAAGGGTTATGGTTGTTGGTTGCGACCCGAAGCATGATTGTACATCGAATTTAAGGGG
AGGAGAAGAGATTCCAACGGTTTTAGATACGTTGAGAGAGAAAGGTATGGAGAAAATGAGTTTAAGTGATATA
GAAAAGAGAATAGACATAGATGAGATTGTTTATAAGGGATATAAGGGCATTTATTGTATTGAGGCGGGGGGTC
CTAAGCCGGGTTATGGGTGTGCTGGGAGGGGTGTTATTGTTGCTATTGATTTGTTAAAGAAGATGAATGTTTT
TGAAGAGTTGGGAGTTGATGTTGTTTTATATGATGTCCTTGGGGATGTTGTTTGTGGGGGGTTTGCCATGCCT
TTGAGGATGGGGTTGGCTGAGCAAATTTATATTGTTACTTCTTCCGATTACATGGCTATGTATGCTGCAAACA
ATATTTGCAGAGGGATGAAAGAATTTGCAAAAAGAGGTGGAAGTAGGTTAGGTGGTTTAATTTATAACGTTAG
GGGTTCTTTGGATGCGGAGGATATTGTTACTGAGTTTGCTAAAAAATTGGGAACTGAGATTATTGGTAAAATT
CCAAATTCTTTGTTAATTGCTGAGGCGGAGATTGAGGGTAAGACGGTTATTGAATATGCCCCAGACAGTGAGA
TTGCATCAATTTATAGAGAACTTGCTAAGAAGATTTACAAAAATAAAAATGGAGTTATCCCAAATCCATTGGA
AAATGAAGAGATTATGCAGATTGGAAAGAGAGTTAAGGAGAGAATTAGGAGTTTAAGGTCTTAA
>mig_Metig_1419-Ar
TTGAAACAGATCGCTTTTTATGGGAAAGGGGGAATTGGTAAATCGACGACTGTCTGTAATATAGCGGCGGCAT
TGGCAGATGAGGGGAAAAGGGTTATGGTTGTTGGTTGTGATCCGAAGCATGATTGTACATCGAATTTAAGGGG

```
AGGAGAAGAGATTCCAACGGTTTTAGATACGTTGAGGGAGAAGGGTATGGAGAAACTCAGTTTGGAGGATGCA
ATAGAGGGAAAAAAGATTGAAATGGATGAGATTGTTTATAAGGGATATAAGGGTATTTATTGTATTGAGGCAG
GGGGTCCTAAACCGGGTTATGGGTGTGCTGGGAGGGGTGTTATTGTTGCTATTGATTTGTTAAAGAAGATGAA
TGTTTTTGAAGAGTTGGGAGTTGATGTTGTTTTATATGATGTCCTTGGGGATGTTGTTTGCGGTGGATTCGCG
ATGCCTTTGAGGATGGGGTTGGCTGAGCAAATTTATATTGTTACTTCTTCCGATTACATGGCTATGTATGCTG
CAAACAATATTTGCAGAGGGATGAAAGAATTTGCAAAAAGAGGTGGAAGTAGGTTAGGTGGTTTAATTTATAA
CGTTAGGGGTTCTTTGGATGCGGAGGATATTGTTACTGAGTTTGCTAAAAAATTGGGAACTGAGATTATTGGT
AAAATTCCAAATTCTTTGTTAATTGCTGAGGCGGAGATTGAGGGTAAGACGGTTATTGAATATGCCCCAGACA
GTGAGATTGCATCAATTTATAGAGAACTTGCTAAGAAGATTTACAAAAATAAAAATGGAGTTATCCCAAATCC
ATTGGAAAACAAGGAATTAATGCAAATTGGATTGGCTATTAAGGAAAGGATTAAAAAAATGAGGAACTAA
>mae_Maeo_0506-Ar
GTGAAACAAATAGCATTTTATGGAAAAGGAGGAATTGGTAAATCTACAACAGTATGCAATATAGCGGCGGCAC
TTGCAGATGAGGGAAAAAAGGTAATGGTAATCGGGTGCGACCCAAAACACGACTGCACTTCAAATTTAAGGGG
AGGGGAAGAGATTCCTACAATATTGGACACGCTGAGAGAAAAAGGAATGGAAAAACTCAGTTTAGAGGATGCA
ATAGAGGGAAAAAAGATTGAAATGGATGAAATCATCCATAAAGGGTATAATGGCATATACTGCATTGAGGCAG
GAGGACCAAAGCCAGGATACGGATGTGCTGGAAGGGGTGTCATTGTAGCAATAGATTTGTTAAAGAAAATGGA
TATATATGAGGAATTAGGCGTGGATATAGTTCTTTATGATGTTCTTGGAGATGTTGTTTGTGGAGGATTTGCC
ATGCCTTTAAGAATGGGATTGGCTAAGCAAATTTATGTTGTAACTTCCTCCGACTACATGGCAATGTATGCGG
CAAATAATATATGCAGAGGCATGAAGGAATTTGCTAAAAAAGGAGGAAGTAGGCTGGGAGGATTAATTTACAA
TGTTAGGGGCTCATTAGATGCTTATGATTTAGTTGAAGAAATGGCCAATAAAATAGGAACTGAGATTACAGGC
AAAATTCCAAATTCCATGCTTATTGCTGAGGGTGAGATTGAAGGAAAAACTGTAATTGAATATGCTCCTGACA
GTGAAATTTCATCTATTTATAGAGAGCTCGCCAGGAAGATTTATGAAAATGATATGGGCGCAATCCCAAATCC
CCTGGAAAATGAAGAAATAATGCAAATTGGGCAGGCAGTCAAGGAAAGAATTAAAAAAATGAAGAACTAA
>mmx_MmarC6_0803-Ar
TTGAAACAGATTGCGTTCTACGGAAAAGGAGGCATTGGAAAATCAACTACAGTTTGTAATTTGGCTGCGGCAT
TATCAAAAAGTGGTAAAAAAGTTATCGTTGTAGGATGCGATCCAAAACATGACTGTACTTCTAATTTAAGGTG
TGGCGAAGATATACCTACTGTTTTAGATGTTTTGAGAGAAAAAGGAATTGATAAACTCGGAATTGAAACCATA
ATTCGCGAGAATTTATTAAAAAAAGAAGATATAATTTATGAAGGATTCAATGGAATTTACTGTGTTGAGGCAG
GCGGTCCAAAACCAGGATACGGCTGTGCCGGACGGGGCGTGATAGTTGTAATTGATCTTCTCAAAAAAATGAA
GGTTTTTGAAGAACTTGGCGTTGATGTAGTTCTTTACGATGTTTTAGGGGACGTTGTATGCGGAGGATTTGCA
ATGCCACTTAGAATGGGGCTTGCTGACCAGATTTACGTTGTAACTTCTTCAGATTACATGGCGCTCTATGCAG
CAAACAATATCTGCAATGGAATAAACCAGTTTGTAAAACGTGGAGGAAGTACGCTTGGAGGAATAATATACAA
TGTCAGGGGATCAATGGATGCGTTTGATATTGTAAGCGAATTTGCAAGTCAATTAAATGCAAATATCATTGGA
AAAGTTCCAAATTCTCCAATAATTAATGAAGCTGAGATTGATGGACAGACTGCAATAGAATACGCTCCAGAAG
AAGAAATAAGTAAAATATACATGGAACTTGCAGAGAAAATATATGAAAATAATACTGGAACGACCCCTAACCC
CTTAGAAAACGCCCAAATAATGCAGATTGGAAAAATGATTAAAGAAAGAATAAAAAAGCAGAAAATTGTCGAG
TAA
>mmz_MmarC7_1147-Ar
TTGAAACAGATTGCGTTCTACGGAAAAGGAGGCATTGGAAAGTCGACTACTGTTTGTAATTTGGCAGCGGCAT
TATCAAAAAGTGGTAAAAAGGTTATCGTTGTAGGATGTGATCCAAAACACGATTGTACGTCCAATTTAAGACG
TGGTGAAGATATACCTACTGTTTTAGATGTTTTGAGAGAAAAAGGAATTGATAAACTTGGAATTGAAACCATA
ATTCGCGAGAATTTATTAAAAAAAGAAGACATAATTTACGAAGGATTTAATGGAATTTACTGCGTTGAAGCAG
GCGGCCCAAAACCCGGGTACGGCTGTGCGGGAAGAGGAGTGATTGTTGTAATCGACCTCTTAAAAAAAATGAA
CGTTTTTGAAGAACTCGGTGTAGATATAGTTATTTATGATGTTTTAGGGGATGTTGTGTGCGGAGGATTTGCA
ATGCCGCTTAGAATGGGGCTTGCTGACCAGATTTACGTAGTAACTTCTTCAGATTACATGGCGCTCTATGCAG
CAAACAATATCTGCAACGGAATAAGCCAGTTTGTAAAACGTGGAGGAAGTACACTTGGAGGCATTGTATACAA
CGTCAGGGGATCTATGGATGCATTTGATATTGTAAGTGAATTTGCGAGTCAGTTAAATGCAAATATCATTGGA
AAAGTTCCAAATTCTTCAATAATTAACGAAGCTGAAATCGATGGACAGACTGCAATAGAATACGCTCCAGAAG
AAGAAATAAGTAAAATATACATGGAACTTGCAGAAACGATATATAAAAATAATACTGGAACGACTCCAAAACC
GCTTGAAAACGCCCAAATAATGCAGATTGGAAAAATGATTAAAGAAAGAATAAAAAAACAGAAAACTGTCGAA
TAA
>mmq_MmarC5_1528-Ar
ATGTACTATATTGTTTTAATTAAATTGCGAGAATCCCCCATATTTAGGTTAATCGGTGATATTTTGAAACAGA
TTGCGTTCTACGGAAAAGGTGGCATCGGAAAGTCGACTACGGTTTGTAATTTAGCAGCGGCATTATCAAAACG
```

TGGCAAAAAAAGTTATCGTTGTAGGATGCGACCCGAAACACGATTGCACCTCCAATTTAAGAAGTGGTGAAGAC
ATACCCACTGTTTTAGATGTTTTGAGAGAAAAAGGAATTGATAAACTCGGAATTGAAACCATAATTCGCGAGA
AATTACTTGAAAAAGAAGATATAATTTACGAAGGATTTAATGGAATTTACTGCGTTGAAGCAGGAGGTCCAAA
ACCAGGATACGGCTGCGCTGGAAGGGGTGTGATTGTTGTAATTGACCTTTTGAAAAAAATGAAAGTTTTTGAA
GAACTCGGTGTTGATGTAGTTCTTTACGATGTTTTAGGAGATGTTGTGTGCGGAGGATTTGCAATGCCACTTA
GAATTGGCCTTGCTGACCAGATATACGTTGTAACTTCTTCAGATTACATGGCACTTTACGCGGCAAACAACAT
CTGCAGTGGAATAAGCCAGTTTGTAAAACGTGGAGGAAGTACACTTGGAGGAATAATATACAATGTCAGGGGC
TCTATGGATGCATTTGATATTGTAAGTGAATTTGCAAACCAGTTAAATGCAAACATTATTGGAAAAGTTCCAA
ATTCCCCAATAATTAACGAAGCCGAAATCGATGGGCAAACTGCAATAGAATATGCTCCAGAAGAAAAAAATAAG
TGAAATATATATGGAACTTGCAGAAAAAATATATGAAAATAATACTGGAACGACACCTAACCCCCTAGAAAAC
GCTCAAATAATGCAGATTGGAAAAATGATTAAAGAAAGAATAAAAAAGCAGAAAATTGTCGAATAA
>mmp_MMP0147-Ar
ATGTACTATATTGTTTTATTAATTTTACGAGAATCCTTCATATTCATGTTAAACGGTGATATTTTGAAACAGA
TTGCATTTTACGGGAAAGGAGGCATTGGAAAGTCCACTACTGTTTGTAATTTAGCAGCTGCGCTATCCAAAAG
TGGTAAAAAAGTTATCGTTGTTGGATGCGACCCAAAACACGATTGTACATCTAATTTAAGAAGTGGAGAAGAT
ATTCCTACTGTTTTAGATGTTTTGAGGGAAAAGGGAATCGATAAACTCGGGATTGAAACTATAATTCGCGAAA
ATTTACTAAAAAAAGAAGATATAATTTATGAAGGATTTAACGGGATTTACTGCGTCGAAGCAGGCGGCCCAAA
ACCAGGATATGGATGTGCAGGAAGAGGGGTGATTGTTGTAATAGACCTCCTCAAAAAAATGAACGTTTTTGAA
GACCTAGATGTTGATGTAGTACTTTACGATGTCTTAGGCGATGTTGTGTGCGGAGGATTTGCAATGCCGCTTA
GAATGGGTCTTGCTGACCAGATTTACGTTGTAACATCTTCAGATTACATGGCGCTCTATGCTGCAAATAATAT
ATGCAGTGGAATAAGCCAGTTTGTAAAACGTGGAGGAAGTACCCTTGGAGGTATAATTTACAACGTAAGGGGT
TCAATGGACGCATTTGATATTGTAAGTGAATTTGCAAACCAGTTAAATGCAAACATTATTGGAAAAGTTCCAA
ATTCCCCAATAATCAATGAAGCTGAAATTGATGGACAGACCGCAATAGAATATGCTCCAGAAGAAGAAATAAG
TAAAATATATATGGAACTTGCAGAAAGTATATACCAAAATAATACTGGAACCATGCCCAATCCATTGGAAAAC
GCCCAAATAATGCAGATTGGAAAAATGATTAAAGAAAGAATAAAAAAACAGAAAATTACTGAATAG
>mvn_Mevan_1152-Ar
TTGAAACAGATTGCATTTTACGGAAAAGGAGGGATTGGAAAGTCAACTACAGTATGTAACTTAGCCGCAGCAC
TGTCAAAAAGCGGAAAAAAGGTTATTGTCGTTGGTTGTGATCCAAAACATGACTGTACATCAAATTTAAGAGG
TGGAATAGAAATTCCAACCGTTTTAGACATTTTAAGAGAAAAAGGAATAGATACGTTGGGAATAGATACAATA
ATTCATGAAAATTTGCTTAAAAAAGAAGATATACTTTACAAAGGATTTAATGGCATATACTGCGTTGAAGCTG
GGGGCCCTAAACCCGGATACGGTTGTGCAGGGCGAGGAGTTATTGTAGTTATTGATTTACTTAAAAAAATGAA
TGTTTTTAAAGATTTAGGCGTTGATATTGTTCTTTATGACGTTTTAGGAGATGTTGTTTGTGGAGGATTTGCA
ATGCCCTTAAGAATGGGATTAGCCGACCAAGTGTATGTTGTAACATCTTCAGACTACATGGCATTATATGCTG
CAAATAATATATGTAATGGAATGAGTCAATTTGCCGTTCGTGGCGGGAGCAATTTAGGCGGCATTATTTATAA
CGTTAGGGGTTCAATGGACGCCTTTGATATCGTTGATGAATTTGCAAAACATTTAAATGGTAAAATAATTGGA
AAGGTTCCAAATTCGTCAATAATAAATGAAGCAGAAATTGATGGAAAAACTGCGATTGAATATTCGCCTGATT
CAGAAATAAGTAATATTTACATAGAACTTTCAAAAAAAATTTACGAAAACAATTCTGGAACAGTTCCAAAACC
ACTTGAAAATATTCAAATAATGCAGATTGGAAAAATGATTAAGGAAAGGCTTAAAAAAGAGAGGATTTTAGAA
TAA
>mif_Metin_0054-Ar
TTGAAACAGATAGGGTTTTATGGGAAAGGGGGAATAGGGAAGAGTACAACAGTTTGTAATATAGCCAAGGCCC
TCTCTGAGGAGTATAGGGTTTTAGTTATTGGCTGTGATCCTAAGGCTGATTGTACAGCTTTACTAAGAGAGGA
AGATATAAAAACTGTCTTAGAAGTCTTAAGGGAGAAAAGAAGTGTGAAGATTGAAGATATTGTTGTAGAGGGA
GGAAAAAAATATTTACTGTGTTGAAGCTGGAGGACCTAAGCCAGGAGTTGGCTGTGCTGGAAGAGGGGTTATAG
TGGCTATAGAAACATTAAAAAAATTAAATGTCTTTGAAAATTTAGATATTGACATTGTTCTCTATGACATCTT
GGGAGATGTTGTCTGTGGTGGCTTCTCCCTACCTTTAAGGTTCTTAGATGATGTTTATATCATAACTTCATCT
GACTACATGTCTCTTTATGCAGCCAACAATATTGCTATGGGAGTTAAAGAGCTAAAAAATAAATTGTCAGGGG
TTATTTATAATGTTAGAGGTTCTTATGACAATGAAGAGGTTGTTAAAGAATTCTGTAAAAGAATCAATACTAA
TTACATTGGAAAGATTCCAAACTCTCCATTAGTAGCTAAGTCTGAAGTCTTAGGAAAGACTGTTATTGAAGCC
TTTCCTGATAGTGATATAGCTAAAATTTATAAAGAGTTAGCAAGGAAAATTTATGAAAATAGAGAGAAGAGAG
AGCCAATGCCATTAAGTAAGAGAGAGCTAAAGGAGTTGGCTTTAAAATATTTGAAGTGA
>mae_Maeo_1224-Ar
ATGGTTAGAAAATTTTGCGTCTATGGAAAGGGAGGAATTGGAAAATCTACAAATGTATCAAACATAGCGGCGG
CACTAGCGGAATCTGGTAAAAAAGTAATGGTCATAGGATGCGACCCAAAGGCAGACTCAACAAGAAATTTAAT

GGGAAGGAAAATTCCAACAGTTTTAGATGCCCTTAGAAAAAATGGTGCCAACAATCTGGAACTTGAAGATATT
GTTTTTAAAGGATTTGGTGAAACCTACTGTGTAGAAAGCGGAGGACCCGAACCCGGCGTAGGCTGTGCGGGAA
GGGGAGTAATCACGGCAATAGATATATTGAATAGATTGGAAGCATTTGAAACAATAAAACCCGATGTAATTAT
TTACGATATTTTAGGAGATGTAGTATGTGGCGGATTTGCCATGCCATTACAGAAACATCTTGCAGATGATGTT
TATATTGTTACAACCTGCGACCCAATGGCAATTTATGCAGCAAATAACATATGTAAAGGAATAAAAAGATATG
CCAATAGGGGAAAGGTAGCATTGGGGGGAATTATATACAGTGGAAGAAGTGTAATAAATGAGCCATCTATTGT
AGAAGAATTTGCCTCAAAAATTGGAACAAATGTAATGGGAAAGGTTCCAATGAGTAATATTATTACAAAGGCA
GAAATTTACAAAAAAACAGTAATAGAATATGCGCCAGATAGTGATGTTTCAGAGGTTTTTAGAAAACTTGCCG
ATACAATATATAAAAATGATAAAAAAGTTGTTCCAAAACCTTTATCTGAAGAAGGAATTGATGAAATTACTGA
AAAAATAGACGATTTAATTAAAAATACCATTTAA
>mok_Metok_0807-Ar
ATGGTTAGAAAATTTTGTATCTATGGAAAGGGAGGTATTGGAAAATCCACAACCGTATCCAATATAGCCGGAG
CTCTGGCTGAATCTGGTAAAAAAGTAATGGTCATAGGATGCGACCCAAAGGCAGATTCAACAAGAAATTTGAT
GGGTAGGAAAATACCAACAGTTTTGGATGTTTTTAGAAAAAAGGGTAATGCTATTAAACTTGAAGATATTGTT
TTTGAGGGATTTTGTGGAACTTACTGTATTGAAAGTGGGGGTCCTGAACCTGGTGTAGGATGTGCAGGAAGGG
GAGTAATTACGGCAATAGAGGTATTGAATAGGTTAGGGGCATTTGAAACATTAAATCCTGATGTAATTATCTA
TGATATATTGGGGGATGTTGTTTGTGGTGGATTTGCCATGCCATTACAAAAACACCTTGCAGATGATGTTTAT
ATCGTTACAACATGCGACCCAATGGCAATTTATGCTGCAAATAATATATGTAAGGGAATAAAAAGATATGCAA
AAAGAGGAAAAGTGGCATTGGGAGGAATTATATATAATGGAAGAAGCGTAATAAACGAGCCTTCCATTGTAGA
GGAGTTTGCTTCAAAAATTGGAACAAATGTTATGGGAAATGTTCCAATGAGTAATATCATCACAAAGGCAGAA
ATTTACAAAAAAACCGTAATAGAATATGCACCAGATAGCGAGATTGCAGATGTTTTTAGGGAGCTCGCAGATG
CCATTTATAAAAACGATAAGAGGGTTATTCCAACTCCTTTATCTGAGGAAGAAATTGACGAGATTACGGAAAA
GATAGACGATTTATTAAAAGAAAAGATAGTTATTGTATAA
>mfs_MFS40622_1279-Ar
ATGAGGAAATTTTGTGTCTATGGAAAGGGAGGAATTGGAAAATCCACTACGGTGAGTAATATAGCAGCAGCTT
TGGCAGAGGATGGAAAGAAGGTTTTGGTTGTTGGCTGTGACCCAAAGGCAGATACAACAAGAAACTTAGTAGG
GAGGAAGATTCCAACTGTCTTAGATGTCTTTAGAAAAAAAGGGCCGGACAATATGGAATTGGAAGATATTGTT
TTTGAAGGCTTTGGTGGAGTTTATTGTGTTGAATCTGGAGGGCCTGAACCTGGAATAGGATGTGCTGGGAGGG
GAGTTATAACGGCAGTTGATATGCTAAATAGATTAGGGGTTTTTGAAGAACTAAAGCCAGATGTGGTTATCTA
TGATATTCTGGGAGATGTTGTTTGTGGTGGCTTTGCAATGCCTTTACAAAAGCACTTGGCTGATGATGTTTAT
ATTGTAACCACTTGCGACCCAATGGCAATCTATGCGGGAAACAATATATGCAAGGGGATAAAGAGGTTTGCAA
GCAGAGGAAAGATTGCACTGGGAGGAATAATTTACAATGGAAGGAGTGTTATAGATGCCCCAGAGATTGTTGA
AGAATTTGCCAAAAAAATTGGAACTCAAGTTATTGGAAAAATCCCAATGAGTAATTTAATAACAAAGGCAGAG
ATTTACAAAAAGACAGTTATTGAATATGCCCCAGACAGTGAGATAGCAAATATATTTAGAGAGATTGCAAGGG
CAATATATGAAAATGAAAGTAGAGTTGTCCCAAATCCGTTATCAGAGGAGGAGTTGGATGAAATTACTGAAAA
GATTGATGTACTGTTAAAGGAGAGTGTTAAAGAATAA
>mja_MJ_0879-Ar
ATGAGAAAAATTTTGTGTCTATGGAAAGGGAGGAATTGGAAAATCTACAACTGTCTCAAATATTGCAGCAGCTT
TGGCAGAAGATGGAAAGAAGGTTTTAGTTGTTGGTTGTGACCCAAAAGCAGATACGACAAGAAATTTAGTTGG
AAGAAAGATTCCAACAGTTTTAGATGTTTTTAGAAAAAAGGGAGCAGAAAATATGAAATTGGAGGATATAGTT
TTTGAGGGTTTTGGAGGAGTTTATTGTGTTGAGTCTGGAGGACCTGAGCCAGGGGTTGGATGTGCTGGGAGGG
GAGTTATTACAGCGGTTGATATGCTAAACAGATTAGGGGCTTTTGAAGAACTAAAGCCAGATGTTGTTATTTA
TGATATTTTAGGGGATGTTGTTTGTGGTGGTTTTGCAATGCCTTTACAAAAACACTTAGCAGATGATGTTTAT
ATTGTAACAACCTGCGACCCAATGGCAATCTATGCGGCAAACAATATATGTAAAGGGATAAAGAGGTATGCAA
GTAGGGGAAAGATTGCATTAGGGGGGATTATTTACAATGGGAGGAGTGTTATAGATGCTCCAGAAATTGTAAA
AGATTTTGCCAAAAAAATTGGAACTCAAGTTATTGGAAAAATCCCAATGAGCAACATTATTACAAGAGCAGAG
ATTTACAAAAAGACAGTTATTGAATATGCTCCAGACAGTGAGATAGCAAATACATTCAGAGAGATTGCAAAGG
CAATTTATGAAAATGAAAATAGAGTTATTCCAAATCCGTTATCAGAGGAGGAATTGGATGAGATTACAGAAAA
AATCGATGTTCTTTTAAAAGAGAGTGTTAAAGGATAA
>mfe_Mefer_1138-Ar
ATGAGAAAATTCTGTGTCTATGGGAAAGGAGGGATTGGAAAATCAACAACTGTAAGTAATATAGCAGCAGCTT
TAGCAGAAGAAGGAAAAAAGGTTTTAGTTGTTGGCTGTGATCCAAAGGCAGATACAACAAGGAATTTAGTTGG
AAGAAAGATTCCAACGGTTTTAGATGTTTTTAGGAAAAAAGGAGCAGACAATATGGAGTTAGGGGATATAGTT
TTTGAAGGTTTTGGAGGAGTTTATTGTGTTGAAAGTGGAGGACCTGAGCCAGGAGTAGGATGTGCTGGGAGGG

GAGTTATTACAGCGGTTGATATGTTAAATAGGTTGGGAGCTTTTGAAGCATTAAATCCAGATGTCGTTATTTA
TGATATATTGGGAGATGTGGTTTGTGGAGGATTTGCAATGCCTTTACAGAAGCACTTGGCTGATGATGTTTAT
ATCGTAACAACCTGCGATCCAATGGCAATTTACGCAGCAAACAATATATGCAAAGGAATAAAGAGGTATGCAA
GTAGAGGAAAAATTGGGTTGGGAGGGATTATTTACAATGGAAGGAGTGTTATAAACGCTCCAGAAATTGTTGA
GGTCTTTGCTAAAAAAATAGGAACTCAAGTTATTGGAAGGATTCCAATGAGTAATCTTATAACAAAGGCAGAG
ATTTATAGAAAGACAGTTATTGAATATGCTCCAGATAGTGAAATAGCAGATAGATTTAGAGAATTGGCAATTG
CAATCTATAAAAATGAAAATAGGGTTATTCCAAATCCGTTATTAGAGGATGAGTTGGATAAGATTACTGAAAA
AATTGACGACCTTTTAAAAGAAAGTGTTAAAGAATAA
>mvu_Metvu_0884-Ar
ATGAGAAAATTTTGTATTTATGGGAAAGGAGGTATTGGAAAATCCACAACTGTGAGTAATATAGCAGCAGCGT
TGGCAGAAGATAAAAAGAAGGTCTTGGTTATTGGTTGTGATCCCAAGGCAGATACAACAAGAAATTTAGTTGG
GAAAAAAATTCCAACGGTTTTAGATGTTTTTAGAAAAAAAGGAGCAGAAAATATAACATTAGAGGATATTATC
TTTCAGGGCTTTGGAGGCGTTTATTGTGTTGAAAGTGGAGGACCTGAGCCAGGAGTCGGGTGTGCTGGAAGGG
GAGTTATTACCGCAGTAGATATATTAAATAAATTAGATGCATTTGAAATAATAAATCCAGATGTTGTGATTTA
TGATATTTTGGGAGATGTTGTATGCGGAGGATTTGCAATGCCCTTACAGAAACACTTAGCCGATGATGTTTAT
ATTGTAACAACATGCGATCCAATGGCAATATACGCGGCTAATAATATATGCAAAGGAATAAGAAGATATGCGA
ATAGAGGAAAAATAGCATTAGGAGGAATTATTTACAATGGAAGAAGTGTTATAGATGCCCCTGAGATCGTTGA
GAATTTTGCTGAAAAAATCGGAACCAAAGTTATTGGAAAAATACCAATGAGCGATCTTATCACAAGAGCAGAG
ATCTATAAAAAGACCGTTATTGAATACGCTCCCGATAGTGAAGTTGCAGAGAATTTTAGGAAACTCGCAAATG
CAATTTATAAAAATAAAAATAGAATTATTCCAAATCCACTAACAGAAGAAGAATTAGATAAAATTTCTGAAAA
GATCGACAGTTTAATAAAGCAAAACTTATAA
>mvo_Mvol_0700-Ar
ATGAGGAAATTTTGTATATATGGTAAAGGAGGGATTGGAAAATCCACAAATGTCGGTAATATGGCAGCAGCTT
TGGCAGAAGATGGTAAGAAAGTTTTAGTTGTAGGTTGTGACCCAAAAGCCGACTCAACAAGAACTTTAATGCA
CGGCAAGATTAATACTGTTTTAGATACATTCCGTGATAAAGGACCAGAATATATGAAAATTGAAGATATTGTT
TACGAGGGTTTTAATGGCGTTTACTGTGTAGAGAGCGGTGGTCCAGAGCCTGGTGTGGGCTGTGCAGGGCGTG
GAGTAATAACCGCAGTTGATATGTTAGATAGATTGGGTGTTTATGACGAACTAAAACCTGATGTAGTTATGTA
CGATATATTGGGGGACGTCGTTTGCGGTGGTTTTGCTATGCCACTTCAAAAGAGACTCGCAGAAGACGTATAT
ATCGTAACCACTTGCGACCCGATGGCAATTTATGCAGCAAACAACATATGTAAAGGTATACAAAGATACGGCA
ATAGGGGTAAAATAGCACTCGGTGGTATTATATACAATGGTAGGAGCGTTGTAGATGAGCCTGAAATTATCGA
TAAGTTTGTACAAGGCATAAATACGCAAGTAATGGGTAAAATACCTATGAGTAATATAATCACTAAAGCAGAG
CTTAGGAAACAGACAACAATTGAATATGCGCCAGATTCCGAAATTGCGAATAAATTTAGAGAACTTGCGAATG
CAATTTATGAGAACAAAAAAACCACAATACCGACCCCATTAAGTGAGCAAGGTCTTGACGACTTAACAGAAAG
TATTGAAGAATTAGTTAGAAAAAAATATGAATAA
>msi_Msm_1707-Ar
ATGATTAAAAAGATAGCTATTTATGGAAAAGGCGGAATTGGAAAAAGTACAACAGTAGCTAATTTATCTGCAG
TTTATGCAAATAATGATTTAAACTGTTTGGTTATTGGATGTGATCCGAAAGCTGATACAACACGTACATTGTG
CGGTAGAAGAATCCCGACAGTTGTAGATACACTTAAAAATAACAGAAAACCCAGTGAAGAGGATATTATTGTA
AAAGGATACAATGATATTTTATGTGTTGAAAGCGGAGGTCCTGAACCGGGTGTTGGCTGTGCCGGACGTGGTG
TAATAGTAGCTATGAAAAGACTTGAAAACTTAGGTGTTTTTGATAAGGATTTGGATGTAGTCATTTATGATGT
ACTTGGAGATGTTGTTTGCGGAGGTTTTTCAGTGCCTTTACGTGAAAAGTATGCTGATGAAGTAATAATTGTA
ACTTCCGGAGAATACATGTCATTATATGCGGCTAATAATATTGTTAAAGGAATTAAAAAACTTAAAGGAAATT
TAAGCGGAATAATCTGCAACTGCAGAAATGTTGACCATGAAAAGGAAATTGTCAGCGAATTTGCATCAAAGAT
AGGAACTCGTATTATTGGAACTATTAATAGAAGCAATTTAATTCAGGAAAGTGAATTAGATGCAAAAACCGTT
GTAGAAAAATACCCTGAATCTAAGGAAGCTAGTGAATATGAATCCTTAGCTACAAATATTATGGAGAATAAAG
TTTTCACAACACCGGAACCTATGGATGATGAGGAATTTGAAAAATTTTTCAAATCATTTTTAGAATAA
>mst_Msp_1120-Ar
ATGGTTTTAAAAAAAATAGCAATATATGGTAAGGGTGGAATAGGAAAATCAACAACAGTATCAAATATGGCTG
CAGCATATGATAATTCAACATTTGTTATTGGCTGTGATCCTAAGGCTGATACTACAAGAACATTGGTAGGTAA
ACGTATACCAACAATACTTGATACAATGAGAGATAATACTCAACCTGAACTTGAAGATATTGTATATGAAGGA
TATAATAATACTTTATGTGTAGAATCTGGTGGACCTGAACCAGGAGTAGGTTGTGCTGGTAGGGGTGTAATTG
TTGCAATGAACCTACTTGATAAAATTGGTGCATTTGATAATGATCCTGATTTAGTAATATATGATGTTTTAGG
TGATGTTGTATGTGGTGGTTTTAGTGTACCATTACGTGAGGATTATGCTGATGAAGTATATATTGTAACTAGT
GGAGAATACATGTCATTATATGCTGCAAATAATATTGCTAAGGGAATTAAGAAATTAAATGGTAAACTTGGTG

GTATTATTTGTAACTGTAGGAATGTGAAAAATGAGGTGGAAGTGGTTACAGAGTTTGCATCACTTATTTCAAG
TAAGATTATGGGTATAATTCCCAGATGTGAACTTGTTCAGACAAGTGAATATAAGGCAAGTACAGTTGTTGAA
ACTTTCCCTGATTCTAGTCAGTCAAAAATCTATTCTGAACTTATAAACAATATTATGAATAATCAGGAATTTT
CTACACCAACACCTATGGGTATTGAGGAATTTGAAGAGTTCTTCTATTCATATGTTTAA
>mru_mru_1466-Ar
ATGAAAAGACAGAAAAAAATAGCTATCTATGGAAAAGGTGGTATAGGTAAAAGTACTACAGTAGCTAATATTG
CAGCAGCTTACAGTGAAGACAATAAGAAAGTCATGGTTATTGGCTGCGATCCCAAGTCAGATACAACAAGAAC
ATTATGCGGTAAGAGAATACCTACCATAGTTCATACCTTAAAGGACAATAAGAAGCCAGAGCTTGACGATTTG
GTTTTCGAAGGATTTAATAAAATCAAATGTGTTGAAAGCGGAGGGCCAGAGCCGGGGGTAGGCTGTGCAGGTC
GTGGTGTCATTGTTGCTATGAAACGTTTGGAGAACTTAAATGCCTTTGAAGAGGAATTTGATGTAATCTTATA
TGATGTTCTTGGAGATGTGGTCTGCGGAGGATTTTCAGTTCCTCTAAGGGAGGATTATGCTGATGAGGTCTTT
ATAGTATCCTCTGGAGAGTATATGTCATTATATGCTGCAAATAATATATCAAGAGGAATTAAAAAACTAAAAG
GAAATCTTGGAGGCATTATCTGTAACTGCAAAGGTATAGACAATGAGGAAGAGATTGTTAATTCTTTTGCAAA
GGAGATTGGCACTCAAGTGATTGGAGTGATTGGCAGAAGCAATCTCATTCAAAGGAGCGAGTTGGATGCAAAG
ACTGTAGTTGAGTATGCTCCTGAATCAATAGAGTCTGATGCTTATAGAAAGCTTGCAAAGGACATTTTTGATA
ATGATAATTATTCAACTCCACAGCCGATGGAAGATGAAGACTTCGAGAATTTCTTTAAATCATTTATTGATTA
A
>mfv_Mfer_0380-Ar
TTGAAAAAAAGGAAACAACGTAGAATAGCAATATATGGAAAAGGAGGGATTGGAAAATCAACCATAGTTTCAA
ACATTGCAGCTGCATATTCTGATAAGTATAAAGTTTTAGTAATTGGTTGTGATCCAAAATCTGATACAACACG
GACATTGTATGGCTCCAGGATACCCACTGTACTACATATTTTAAAGGAAAAGAAAGAACCAAAAGTTGAAGAT
GTAGTGTATGTAGGATATAATGGTGTTAAATGTGTTGAATCTGGGGGTCCAGAACCTGGTGTTGGTTGTGCAG
GTAGAGGAGTAATTGTAGCTATGAATTTACTTGAAAAACTCGGGGTATTTAGGGAATCTTTAGACATAATAAT
ATATGATGTACTTGGAGATGTTGTTTGTGGAGGATTTGCTGTACCTTTACGTGAAGATTATGCAGATGAAGTT
TATATAGTCACTTCGGGCGAATATATGTCATTATATGCCGCAAACAACATATGTAAAGGTATCAAAAGATTAA
AAGGAAGACTTGGCGGTATCATTTGTAATTGTAGAGGTATAAAGAATGAAGTAGAAATTGTTGAAAAATTTGC
AAAAAAAATAGGTAGTAAAGTTGTAGGTGTAATTCCTAGAAGTGATTTAGTCCAAAAAAGTGAAATAGAAGGA
AAAACTGTAATCGAAAAGTTTCCAAAATCTGAACAAGCAAAAATATATAGAGATCTAGCGAAATCAATATATT
TAAATAAAGATTTCGTTGAACCAAAACCTATGAGTATAGACGAGTTAGAAAAATTTATTAAAAATCTATATAA
TGTCTCATAG
>mel_Metbo_2093-Ar
ATGAGTAAAACCAAAAAAATCGCTATTTATGGGAAGGGAGGGATTGGTAAATCCACCACAGTATCCAACATAG
CTGCTGCCCGGTCCAAGGACAGTGAAGTTCTGGTAATAGGTTGTGATCCTAAAGCAGACACTACTAGAACCCT
TGTTGGAAGAAGAATCCCAACTATATTAGATATTTTAAAGCTTAAAAAGAAGGATCTAAATCAAGAAGACATA
ATAGTAAAGGGCTTTGGTGATGTGAAATGTGTAGAGTCTGGAGGACCTGAACCTGGAGTGGGATGTGCTGGAA
GAGGAGTCATAGTAGCTATGAACCTCCTGGAACGTCTAGGTGTTTTCAAAGAAAACCTGGACCTCATCATCTA
CGACGTGCTTGGAGACGTGGTATGCGGAGGATTTGCAGTTCCACTCCGTGAAAACTTCGCAGATGAAGTTTAC
ATAGTCACCTCAGGTGAATACATGTCTCTGTATGCTGCAAACAACATCTGCAAAGGAATAAAAAAACTCAAAG
GCAATCTTGGAGGCATAATCTGCAACTGTCGGGGAATTGAGAATGAACTCGAAATTGTGGAAGAATTTGCAAA
AATTGTCAACACCAAGATCATAGGAGTAATGCCCCGTAACGAACTGGTTCAAAAGGCAGAGATCGAAGCAAAA
ACAGTGATAGAAAAGTTTCCAGATTCTGAACAGGCTGAGCTTTACATAGAACTGGGAGAAAACATCTACAACA
ACCAGAAATTTGACATTCCAAAACCCATGGAAGTGGATGATTTTGAAAAATTCTTCAGAAAGTTTCACTAG
>mew_MSWAN_0543-Ar
ATGAGTAAGACCAAACGAATAGCTATCTATGGAAAGGGGGGAATTGGAAAATCCACCATTGTCTCCAACATAG
CAGCAGCATACTCTGAAAACTGCAACGTGCTGGTTATAGGCTGCGACCCCAAGGCGGACACCACTAGAACCCT
GGTGGGAAGCAGGATACCAACCATCCTTGAAATTGTGAAGCGTAAAAAAGGGGCATCACCTGATGAAATCCTT
TTTAAAGGCTACGGTGGTGTGAGATGCGTGGAAAGTGGGGGCCCGGAACCTGGAGTTGGATGTGCAGGTCGTG
GTGTTATAGTGGCCATGAACCTCCTTGAGAAGCTTGAAATATTCTCAGAAGACCTCGACGTTATCATCTATGA
TGTACTAGGTGATGTGGTCTGTGGAGGATTTGCAGTTCCTCTAAGGGAAGAATTTGCAGATGAAGTTTACATT
GTAACATCTGGGGAGTACATGGCATTATACGCGGCTAACAACATCTGTAAAGGTATAAAAAAGCTTAAAAGTC
GTTTTGGTGGTGTCATATGCAATCGCAGAGGACTCAAAGATGAAGTAAAAATAGTAGGGGAATTTGCTGAGCG
GGTTGGAAGCAAGATCATTGGAGTCATACCAAAAAGCGAGCTGGTCCAGAAGAGTGAGTATGAGGCCAAAACT
GTTGTTGAAAAATTTCCTGATTCTAAGCAAACTGAACTCTACAGAAAACTTGCAAAAGCCATATATCTAAATG
AAGATTTTGTAGTTCCTGAACCTATGGGTATAGATGAATTTGAAGAGTTTTTCAGGCAATTTTAA

>mmg_MTBMA_c10230-Ar
ATGAAGAGGATAGCGATCTACGGTAAGGGTGGTATAGGGAAGTCCACAATCGTATCGAATATGGCCGCAGCCT
ATTCCTCTCATCACAGGGTACTCGTCATAGGCTGTGATCCAAAGGCGGATACAACCAGGACACTCTACGGTGA
AAGGCTCCCCGCAGTACTTGATGTCCTCAGGGAGAACCGTAAACCTGACGCCTCTGAGGTTATCCATGAGGGT
TTTGGCGGTGTTAGGTGTGTGGAGTCAGGGGGGCCTGAACCCGGTGTTGGATGTGCCGGGAGGGGGGTGATCG
TTGCAATGAACCTCCTTGAAAAACTTGGTGTATTCGAGGAGGAAATTGATGTCATCATATACGATGTCCTCGG
TGATGTGGTCTGTGGGGGATTCGCGGTGCCACTCCGTGAGGAATTTGCAGATGAGGTCTATATAGTAACCTCC
GGGGAGTACATGTCCCTTTACGCTGCAAACAATATTGCAAGGGGTATAAGGAAACTGAGGGGTAAACTTGGGG
GTGTCATCTGTAACTGCCGTGGCATCAGAAATGAGGTTGATATTGTATCTGAATTCGCCTCAAGGATAGGTTC
ACGTGTTATTGGTGTGGTCCCAAGAAGCTCCCTTGTTCAGGAGAGTGAAATTGAGGCGAAAACCGTGATTGAG
AGCTTCCCTGAATCTGAACAGGCAGAGGTTTACAGGAAACTGGCAGATGAGGTTTACCTTAACACGGAATTCG
TGGTACCTGAACCCATGGACCCTGAGGAGTTTGAGGAGTTCTTCAGAAAATTCAGGGGAGATGATTGA
>mth_MTH643-Ar
ATGAAGAGGATAGCCATCTATGGTAAGGGAGGTATAGGTAAATCCACCATCGTCTCAAACATGGCAGCGGCTT
ATTCATCGGAGCACAGGGTCCTTGTGATAGGCTGTGACCCCAAGGCAGATACCACAAGGACACTCTATGGTGA
GAGGCTCCCCACGGTTCTGGATGTGCTGAAGGAGAACAGAGAACCCGATGTTTCTGAGGTAATACACACAGGA
TTTGGGGGTGTAAGGTGCGTTGAATCAGGGGGCCCTGAACCAGGTGTGGGGTGTGCCGGGAGGGGCGTCATAG
TTGCAATGAACCTCCTGGAAAGGCTTGGAGTATTCAGGGAGGACATTGATGTCGTTATCTATGATGTCCTTGG
TGACGTTGTCTGCGGAGGCTTCGCAGTTCCCCTGAGGGAGGACTTTGCAGATGAGGTCTACATAGTTACATCC
GGTGAGTACATGTCCCTTTACGCAGCCAACAACATTGCACGGGGCATAAGGAAGCTCAAGGGTAAACTTGGAG
GTGTCATATGTAACTGCAGGGGCATCAGGGATGAGGTGGAAATCGTATCTGAATTTGCTTCAAGGATAGGTTC
AAGGCTGATTGGAGCTGTGCCCAGAAGCAATCTTGTCCAGGAGAGTGAGCTTGAGGCCAGGACTGTCATAGAA
CGGTTCCCTGAGTCTGAACAGGCCTCTGTTTACAGGAAACTTGCGGAGGATATCTACAGGAACACTGAATTCA
CGGTACCCGAACCAATGGATCAGGAGGAGTTTGAGGAATTCTTCAGAAAATTCAGGGTTGAGGGTTAA
>mbn_Mboo_2008-Ar
ATGGCAAAACAGAGAAACATTGCGATATATGGAAAAGGCGGCATCGGAAAGTCCACCACCTCGTCCAATATCA
GTGCCGCACTCTCGGAACTTGGCCTCAAAGTGATGCAGATCGGATGTGATCCCAAGAGCGATTCAACAAACAC
GCTCCGGGGAGGCCGGTTCATCCCGACAGTTCTGGATTCCCTGAGAAGCGGAAAGAGGGTTGAGACAAGTGAC
ATTATCCACGAAGGATTCAATGGTGTCCTCTGCGTTGAAGCCGGGGGCCCTGAGCCCGGCGTCGGCTGTGCCG
GCCGCGGCATCATCACCGCAATTGAACTCCTCCGCCAGCGGAAGGTCTTTGAAGAGTTCAAACCGGATGTCGT
TATTTACGATGTCCTTGGGGATGTGGTCTGCGGGGGATTTGGTATACCCATCCGTGAAGGCGTGGCCGAACAG
GTCTACACCGTGAGTTCGTCCGACTTCATGGCGATTTATGCGGCAAACAATCTCTTCAAAGGCATCAAGAAGT
ATGCGAACAGCGGGGGAGCATTGTTCTCCGGTATCATAGCAAATTCCACAAACCTGCCTGTACAGCGCGAGAT
TGTCGAGGACTTTGCCGCTTCCACAAAGACAACTATTGCAGAATATGTTCCCCGTTCGCTTACCGTAACCAAA
AGTGAGCTCCAGGGAAAGACTGTGATAGAGGCGGCTCCGGAGTCGGAGCAGGCCGAGGTCTACCGTGTCCTTG
CAAAGAAGATCCTCAGTAATCAGGACCGGTATGTCCCGGCTCCGCTGGATACCGACCAGCTCAAAGACTGGGC
CGAGAGCTGGTCTGACAAACTGCTCGAGCAGCGCGACTCCCCGACCCATGTGAAGTGTGAACTCGAATGCATT
GTCCCCGGCGCAGAGCCAATTATTGCAAAACCCCGATCCCAGCGATCTCCTGCAGTAAAAGCGGCCACTCCCC
GGAGAACTGCCAAAGCAAAAGGATGA
>mpl_Mpal_0095-Ar
ATGACAGCATCACGTAATATTGCCATTTATGGAAAAGGAGGTATAGGAAAGTCGACGACCTCATCAAATCTCT
CTGCAGCCCTCTCTGAACTGGGCCTCACTGTCATGCAGATCGGGTGCGATCCCAAGGCCGACTCGACCAACAA
CCTCCGTGGCGGGGAGTCGATCCCCTCGGTGCTGGACGCCCTGCGAAGTGGAAAGAAGATCGAGATCGGAGAT
ATCGTCTTCAAAGGATTCAATGGGGTTCTCTGTGTCGAGGCCGGCGGGCCCGAGCCCGGAGTCGGCTGCGCAG
GCCGGGGAATCATCACGGCCATCGAACTGCTGAAACAGAAGAAGGTCTTTGAGGAGTTCTCCCCTGATATCGT
GCTGTACGATGTGCTCGGCGATGTGGTCTGCGGTGGTTTCTCTGTCCCGATCCGGGAAGGAGTGGCAGAGCAG
GTCTATACCGTCGCCTCTTCCGACTTCATGGCTCTCTATGCCGCCAATAATCTCTTCAAGGGGATCAAGAAGT
ATGCGAACAATGGTGGAGCTCTCTTCAGCGGGATCATCGCAAACTCGATGAGCAAGCCGGTCCAGCGGGAGAT
CATCGATGATTTCGCCGTTCATACGTCGACGACCGTCGCAGGCTACGTGGAGCGATCTCTGGCCGTGACCAGG
AGTGAACTCCGGGGGCAGACGGTCATCGAACGGGATCCTGACTCCCCCCAGGCCGATGTCTATCGAAGGCTTG
CGAAGGGGATTGTCGAGAATCAGAAGCGGTATGTTCCCGCTCCGCTCGATGCCGAAGAACTCAAAAAGTGGGC
TTCCAGCTGGTCTGATCGGATCATCGGGCAGGACGCTGACGAGGAGGCCCCCAAGAGCACATCATATCCACCA
GCAGGGAGTGAGGGACTCAGTTAA
>mel_Metbo_1180-Ar

ATGAGTAAACGAAAACAAATTGCAATTTATGGAAAAGGCGGGATTGGGAAGTCGACTACAACTTCCAACCTAA
GTGCGGCCCTGTCAGATTTAGGTTACAGTGTAATGCAGATAGGATGTGACCCTAAAAACGATTCAACAACCAC
ACTCAGGAAAGGAAAACCAATACCTACCGTTATGGATACAGTAAGAAGTGGCTCCAACAAACTGGATAATTTA
ATATTTGAAGGTTACAATGGGGTTCATTGTGTTGAAGCAGGAGGTCCTGAACCTGGTGTTGGATGTGCTGGAC
GAGGTATCATTGCAGCCATCGAATTGCTAGACAACAATGGAATAATTGACGACATCGACCCAGATATCGTTAT
ATACGATGTTTTAGGAGATGTTGTTTGTGGTGGATTTGCAATCCCTATCAGGCAGGGAATTGCAGAACAAGTG
TACACAGTTACATCCTCCGATTACATGGCAATTTACGCCGTAAACAACCTCTTCAAAGGAATTCTTAAATATT
CCGGTAGTGGAGGAGCTTTACTTGGAGGAATAATTGGAAATTCCATCTCAAAAATATCACAAAAGGAAATGAT
AACAGATTTCAGCGAGAAAACTGAAACCAATGTTATAGAATTTGTTCCAAGATCATCCACTGTAACTAAATGC
GAATTAGATGGTGTCACCACCATTGAAGGTGCACCAAATTCAGAGCAGGCTGAAGTTTACAGGCAACTTGCAA
GGAACGTAATAAACAACAAAGATTCCTACATTCCAAAACCATTTGAAGCTCAAGATCTTGCAGATTGGGCTGC
TTCATGGATTAACAATCTTCTTAAAGAAGAAAAGATCGAACAGAACGGTATTCGAAGCGGTAGTGGAGAAGGA
ATTTAA
>mew_MSWAN_1020-Ar
ATGACGAAAAGGAAGCAAATTGCAATATATGGAAAGGGCGGTATTGGAAAGTCCACAACAACCTCCAATTTAA
GTGCGGCCCTTTCAGATATTGGTTACAGTGTAATGCAGATCGGCTGTGACCCGAAGAATGATTCAACAACCAC
ATTACACAAGGGAAAAGCAATACCCACAGTTCTTGATACGGTCAGGAGTCGTTCAAACGATTTTGATAAGGTT
ATACATGAAGGTTACAATGGAGTGCAGTGTGTCGAAGCTGGAGGTCCAGAGCCAGGTGTGGGCTGTGCTGGTC
GTGGTATTATTGCGGCCATTGAACTTCTGGATACAAACGGTATAATTGACGATTACGACCCGGACATTGTTAT
TTACGATGTGCTGGGTGATGTTGTATGCGGTGGATTTGCCATTCCAATCAGACAGGGAATTGCAGAACAGGTT
TACACAGTAACATCTTCTGATTACATGGCAATTTATGCTGTTAACAATCTTTTTAAGGGAATATTAAAATATT
CTGGAAGTGGTGGGGCTTTATTAGGTGGAATTATTGGTAACTCCATCAAGAGGATAGCTCAAAAAGACATGAT
AGATGATTTCAGTGATAAAACAGGTACAAATGTAATTGAATACATTCCAAGGTCTTCAACTGTCACTCAATGT
GAGCTCGATGGTGTGACCACAATTGAAGGTGCTCCTGATTCACAACAGGCTCAGATTTACAGAGATCTGGCTC
AAAAAATTGTAGACAACAAACAGAAATATATTCCTAAACCATTTGATGCAGATGATCTATCTGATTGGGCTTC
AACATGGATTAATCGTATACTTACTGAAGAAAAGGTAAGTTCAAGGGGAATACAGAAAGAGGGGGGCGGTGTT
TAA
>mhu_Mhun_0793-Ar
ATGAAACAGATCGCTCTCTATGGCAAGGGAGGTATCGGTAAATCCACCACATCAGCAAATCTTTCCGCAGCCC
TGGTAAACCGTGGTCTTTCAGTTATGCAGATTGGGTGTGATCCGAAACGTGATAGTACCCGGATGCTTATGAA
AGGGATACTTATCCCGACGGTGTTGGATCTTATCCGTGAACGTGGTGAAGAGAACCTTACCCTTGATGATGTT
GTATTCACCGGATATAAGGGGGTTCGGTGCGTGGAGGCAGGAGGACCAGAACCTGGTGTCGGATGTGCAGGCA
GAGGCATTATTGCGACTTTTCAACTTCTTGAACGACTTTCAGCATTTGATGAAGACATAATTGTGTATGATGT
TCTGGGCGACGTTGTCTGTGGCGGGTTTGCTATGCCCATGCGAAAAGGATATGCACAGGAAATATATCTTGTC
ACCTCCGGAGAACTCATGTCTCTCTATGCTGCAAATAATATATGTAAGGCAATCTCCCGGATATCTCAAAATG
TCAGACAGGTCTGCAGACTTGGAGGAGTTATCTGTAACAGCAGAAATCTTCCAGATGAAGAGAAACTGGTGGG
AGCATTTGCATCTGAAGTCGGTTCGAAGATAATCGCCTATATCCCGAGGAGTGGCCTGGTACAGTATGCTGAA
TTAAATAACCAGACAGTTATTGAATTTGCACCTGATTCATCACTCTCTGCAACATATCAATCCCTTGCCGAAG
AAATAATGACGAATACAGATTTCGTAATCCCCAAACCACTAGAGATTGAAGAACTTGAAAAACTTGCCAGATC
TTATCTTCCAACGATCGATCATCAGGGAATCAATCACTAA
>mla_Mlab_1041-Ar
ATGAAAAAAATTGCATTTTATGGTAAAGGCGGAATCGGAAAATCAACCTGTGTCTCAAATATTTCTATGGCTC
TCGCCGAGCAGGGATTCACGGTCATGCAGATCGGCTGCGATCCGAAAGCAGATTCAACGAGAAATCTAACTCA
CGGAGATTTGATCCCATCTGTTCTCTCAGTAATAAGGGAGAAAAAGAATGATATTTGTTTGGAAGATATCGTT
TCCTGTGGATCAGGTGGAGTATTGTGTGTAGAGGCGGGGGGGCCAAAACCCGGGTCAGGTTGTGCAGGTCGCG
GCATTATTACTGCTTTTGAACAATTGGAGGAGCTTGGAGCTTATACTACCTATCATCCAGATATTATTCTCTA
TGATGTTTTAGGAGATGTGGTCTGTGGAGGATTTGCCCTGCCGATACGCCAAGGATATGCCGATGAGATCTGT
ATTGTGACATCCGGCGAGATGATGTCCTTGTATGCCGCGGATAACATCTCTCAGGCGGTGAATAATTATTCAA
AGCGTGGATATGCGGCATTGAAGGGACTCATCTACAATGCAAAAAATATTGAAAATGAAGATGAGATTGTGGA
AAAAGCGGCTGCAGAAATGAATACAAAGGTGTTGTCCAGGATTAACCGTGACCCGATTGTCCAAAAGGCTGAA
AATGCTGGTATGACTGTTGTGGAATATGCACCGAATTCATCTCAAGCAGATTTGTATAGAAAACTAGCAGGAA
TTTTACTTGAGGATTAA
>mac_MA1633-Ar

ATGAGACAGATTGCGATCTACGGTAAAGGCGGAATAGGGAAGAGCACTATCTCCTCGAACCTATCTGCCGCAT
TGCATGAGATGGGACATACGGTCATGCAGGTTGGCTGTGATCCCAAAAGGGATTCGACGCGGATCCTTGCAGA
AGGAAAGTTTATTCCTGCGGTGCTGGAAGAACATCGGGAACAGTTAAGGATAGGAAAAGATGAGTATGCGATC
AATCTGAAGAATATCGTCTTCAAGTCACCCGGCGGGATCTACCTTGTTGAAGCGGGGGGACCGGAACCGGGTA
TAGGGTGTGCAGGCCGGGGAGTCCTGACAGCCCTGCAAATCCTAAAAGACCTCAAGGCATTTTCTACCTACAA
CATCGACGTGGCAATCTACGACGTGCTTGGCGATGTCGTCTGCGGAGGTTTCTCAATGCCGATCCGCGAAGGT
TTCGCAGAAGAGATCTATCTCATCTGTTCCGGAGGGTTCATGAGCATCTATGCAGCAAACAATATTGCTCGGG
CCGTGCAGCGCCTCGCAAAACGTGGGGATGCCGGTCTTGCCGGAATAATCTGCAATAGCAACGGAGATGAGGC
TTTCGAGCATGCGTTCATTCCTGCATTTGCAAAAAGACTGGGGACCCCGTTTGTCCAGTTCGTCCCCCGGAGT
CCGGTGATCCAGGCATGTGAACTGGGAGGGAGGCCTGTAGTAGAGTATGCTCCCGACTCTCAGGAGGCGGAAA
TTTTCCGGAATCTTGCGAAAGCGATCATAGAAAACGATTCGCGTGTTATCCCTACGCCGATAAATGACCTTGC
CGAGCTTGAGACGATGTACCGGCAATGTCTGGTAAAAGATAAGAAATAA
>mpd_MCP_0905-Ar
ATGAGACAGATAGCGATCTATGGTGAGGGAGGCATCGGGAAGAGCACGATCGCCTCGAACCTGTCCGCCGCCC
TTAACGAAATGGGTTACACCGTCATGCAGGTCGGCTGCGACCCCAAGAGGGATTCCACCAGGAACCTGGCGGG
CGGACGCCTGATCCCCGCTGTCCTTGAAACATACAGGGACCAGCTAAGGGTAGGCAAAGATGAATATGCGATC
TCATTAGATAGCATCGTTTTCAAGTCTCCCGGCGGCATCTACTGTGTCGAGGCCGGAGGGCCTGAGCCGGGCA
TAGGCTGTGCCGGCAGGGGCGTATTGACAGCCCTGCAGATATTAAAGGATTTGAAAGCCTTCGAAACCTACAA
GATCGACGTGGCCATCTACGATGTCCTGGGCGATGTCGTCTGCGGCGGGTTTGCCATGCCCATCAGGGAAGGT
TTCGCAAAGGAGATATACCTGGTCTGTTCAGGCGGTTTCATGAGTATCTATGCGGCGAATAATATCGCCCGTG
CTGTACAGCGCCTGTCACGTAGAGCTGAAGCCGGGACAGGGCTTGCCGGTATTATTTGTAACAGCAACGGCGA
CGAAACGCTTGAGCACTCGGTCATACCAGAATTCGCCCGGCGTCTCGGGAGCGAGTTTGTCCAGTTCGTTCCA
AGGACCCCGGTGATCCAGGCCTGCGAGTTCGAGGGAAGAGCTGTCGTGGAGCATTCCCCTAACTCTAAGGAGG
CGGGGATATTCAGAGATTTAGCTAAACAGATCATGGAAAATAAAAAGACGGTAGTTCCGACGCCGATTAATGA
TTTAACCGAGCTCGAGCAGATGTACAGGCAGCATCTCAATAAAAAGTAA
>mem_Memar_1937-Ar
ATGAAACAGATCGCCCTCTACGGGAAGGGGGGAATCGGGAAATCCACCACTGCGGCGAACCTGTCGGCAGCAC
TCGCGGGAGAAGGGCTCGATATCCTGCAGATCGGCTGCGATCCGAAGCACGACAGCACCCGCATGCTGATGCA
CGGGACCTGGATCCCGACGGTCCTCGATCTCATCCGGGAGCGCGGAGACGAGAAGATAACCGTCGACGACGTG
GTCTACCGGGGCTTTCGGGGCGTCCGCTGCGTGGAGGCCGGAGGGCCCGAGCCCGGGATCGGGTGCGCCGGCC
GGGGGATCATCGCGACGTTCCAGCTCCTCGAACGCCTGAACGCCCTCAAGGGCGACGTGATCGTCTACGACGT
CCTGGGTGACGTCGTCTGCGGCGGGTTTGCGATGCCGATGCGCGAGGGCTACGCCCAGGAGATCTACCTGGTC
ACCTCCGGCGAGTTGATGTCCATCTACGCGGCAAACAACATCGCAAAAGCCATTGCGCGGCTCTCGCGCCGGG
TGCGGAGCAGGTGCACGCTCGGGGGCGTGATCTGCAACGCAAAGAACATCGAAGGCGAGCGGGAACTCGTTGA
CGAGTTCGCCCGCCGGATCGGCTCCCGGCTCATCGCCTACATCCCCCGCTCGAGGATCGTCCAGGTCGCGGAG
CTGCAGAAACAGACGGTCGTCGAATACGCCCCCGAATCCGACCAGGCGGCGGTCTACCAGGAACTCGCGCGGA
CGGTCTACGGAAACGAGACGACGAGCATCCCCACACCTCTCGAGATGGATGAACTCGAATCCTTCGCCCTCGA
ATTCGTCCAGGTTTGA
>mpl_Mpal_0237-Ar
ATGAAGCAGATTGCCCTGTACGGCAAGGGCGGTATCGGCAAGTCTACGACCTCGGCCAACCTCTCCGCAGCAC
TCGCTGAGCAAGGGCTCGGTGTCCTCCAGATCGGATGCGATCCCAAGCACGATAGTACCCGGATGCTGATGCA
TGGCACCTGGATACCGACCGTGCTCGACCTGATTCGGGAACGCGGAGGGACCAACCTGACCATCGACGATGTA
GTGTACACCGGATATCGGGGTATACGCTGCGTCGAAGCCGGTGGACCTGAACCGGGGATCGGCTGCGCCGGCC
GTGGGATCATCGCGACCTTCCAGGTGCTCGAGAAACTCGAAGCATTCTCAGCCGACGTGGTCGTCTACGACGT
CCTCGGGGATGTGGTCTGTGGCGGGTTTGCGATGCCGATGCGCGAGGGGTACGCCGAGGAGATCTACCTGGTC
ACCTCAGGCGAGTTGATGTCCCTGTACGCAGCCAACAACATCTGCAAGGCGATCAACCGGCTTTCCCAGCGAC
CGAAGTGTACCTGCCGGCTCGCAGGGGTGATCTGCAATGCCAAGAACATCGACCGCGAGGAGGATCTGGTCAG
GGAGTTCGCCGAACGGGTCGGCAGCAACCTGGTCGCCTATATCCCCCGCGACCGCATCGTGCAGCTGGCCGAG
GTGCACAAGCAGACAGTGCTTGAATATGCCCCCGACTCAGCCCAGGCCGCAACGTACCGAACCCTTGCAGAGG
TGGTGCTGACGAACACCACACTGACGATCCCGAAACCTTTAGAGCTGGATGAACTCGAAGACCTCGCATTCAA
GTACATATCGATATGA
>mbn_Mboo_2083-Ar
ATGAAGCAGATCGCCCTGTACGGAAAAGGCGGTATCGGAAAGTCCACGACTTCTGCGAACCTGTCTGCAGCCC
TTTCCCTTTCAGGGTACAACGTTCTCCAGATTGGTTGCGATCCCAAGCGCGATAGTACCAGGATGCTGATGCA

GGGACGGTTGATTCCTACAGTTATGGATCTGGTGCGCGAACGGGGAGCGGCATCAGTAACCCCGGCGGATGTA
GTCTATACCGGTTTTAATGGCATCCGCTGCGTTGAAGCCGGAGGGCCGGAACCTGGCGTTGGTTGCGCCGGGC
GGGGGATTATCGCCACGTTCCAGCTGCTGGAAAAATTCGGTACGCTCACGGGAGATGTGATCGTGTACGATGT
GCTGGGCGATGTGGTCTGTGGCGGGTTTGCCATGCCGATGCGGGAAGGGTACGCCCAGGAAGTGTACCTTGTA
ACTTCGGGAGAACTTATGTCCCTGTATGCGGCAAACAACATCTGCAAGGCTGTACAGAGGCTGAGTGCCCGCC
CGAGGAGCATGTGCCGCTTGGCCGGGGTTATCTGCAACGCCAAGAACCTGCCCCGGGAAGAGGAGCTGGTGGC
AGAGTTTGCCCGCCGGGTCAACAGCCATCTTCTCCAGTATATCCCGCGTGATCGCACGGTCCAGCTGGCAGAA
CTGGACCGCAAGACCGTGCTTGAGTACGAGCCGTCATCTGAACAGGCACAGAGGTACAGGGACCTTGCAGCCC
GGGTTATGGAAAACACGCGCTTTACCATACCCACCCCCCTTGAGATCGATGAGCTTGAATCACTTGCCCGCCA
GTTCGTGTAG
>mpi_Mpet_2379-Ar
ATGAAGCAGATCGCCCTGTACGGAAAAGGCGGAATCGGAAAATCCACGACCTCGGCCAACCTTTCTGCCGCAC
TTTCTGAAAAATCCCTCGAGATCCTCCAGATCGGATGCGATCCAAAGCATGACAGCACCCGTATGCTTATGCA
CGGAGAATGGATACCCACGGTTCTGGATCTTGTCAGGAAGAAAGGAGAAGCAAATATCGCAGTCGACGAGATC
GTTTTTAAGGGATATAACGGAATCAGGTGTGTAGAGGCCGGCGGGCCGGAGCCTGGGATTGGATGTGCAGGAC
GGGGAATTATCGCAACATTCCAGCTTCTTGAAAAGCTCAACGCACTCTACGGGGACGTAATCGTCTATGATGT
CCTTGGCGATGTCGTATGCGGGGGATTTGCGATGCCGATGAGGGAAGGCTACGCACAGGAGGTCTATCTTGTG
ACATCCGGGGACTTCATGGCCCTTTATGCCGCGAACAACATCTGCAAGGCGATAGCCCGGCTTTCGAAAAGGA
CAAAGAACAGGTGCACGCTCGGGGGAGTCATCTGCAACTCTGCGAATATCGAAAACGAATATGAGCTGGTCAA
AGAGTTTGCAGAAAAGATAAACTCAAAGCTCGTTGCCTATATCCCGAGGAGTCCGATCGTCAGGGTTTCGGAG
GTCAACAGGAAGACAGTTATAGAGTATGCGCCTGAATCCGAACAGGCGGATATATACAGAAAACTTGCCGATA
CGATAATGGAAAATGTGCCTGACAAGAACAGGATCCCTGTACCGATGGAGATGGATGAACTCGAATCGCTCTC
TCTCAAATACATCAAGACATAA
>mac_MA3627-Ar
TTGAAAAAACAAAAGATCGTTGCAATTTACGGAAAAGGCGGCATAGGCAAGTCAAGTACAGCCTCAAACGTTG
CAGCTGCCTGTGCGGAAGCAGGAAAGAAAGTCATGATCATAGGCTGTGACCCCAAAAGCGATTCGTCCATAAC
CCTTCTCAGAGGCAAAAGAATCCCGACAATCCTCGATCTCCTCAGAGAGGGGGTGGATGTACAGGAAAAAGAT
GTGGTTTTTGAAGGGTATGCAGGGGTTAAATGCGTGGAAGCAGGAGGTCCCGAGCCCGGGATAGGCTGTGCAG
GGCGCGGAATTATCGTTGCAATCCAGAAACTCAAGAGCATTTCCGGAGATCTCCTTAAAGAGCAGGATCTCAT
CATCTACGATGTGCCGGGAGACATCGTCTGCGGCGGCTTTGTTGCTCCTGTCAGGAAGGGTTACGTAAATGAG
GCCTATGTCCTGACCTCCGGAGAGTACATGCCCCTCTATGCAGCAAATAACATCTGCAAAGGGCTTTCCAAGA
TCGGGATGCCGCTTAGCGGGGTTATCTGCAACTCGAGGAATGCAAGCCGGGAAGAGGAAATCGTCAGGAAGTT
TTCCGAAGAAATCGGAAGCCAGCTGATGGCTTTCATCCCGAAGCGGCAGATAGTGCAGGACTGCGAAAGAGAA
GGGTATTCCGTTATGGAAAAAGCTCCGGACTCCGACATTGCCGAAGTCTACCGCCAGCTCGGAAAATCAATCC
TTACAAACGAAAAGAAGGTTATGGCATCCCACCTGAGCGATGAGCGGTTAAGGGAAATGACAAAATAA
>mma_MM_0514-Ar
TTGAAAAAACAAAAGATTATTGCAATTTACGGAAAAGGCGGCATAGGCAAGTCGAGCACAGCTTCAAATGTTG
CAGCCGCCTGTGCGGAAGAAGGCAAGAAAGTTATGATCATAGGCTGTGACCCCAAAAGCGATTCTTCGATAAC
CCTTCTCAGAGGGCGGAGAATTCCGACAATTCTTGACCTCCTCCGCGAAGGCGTGGATGTTAAGGAAAAAGAC
ATTGTCTTTGAAGGGTATGCAGGCGTCAAATGCGTGGAAGCCGGAGGCCCGGAGCCTGGAATAGGCTGTGCAG
GACGCGGAATTATAGTCGCAATCCAGAAATTAAAGAGCGTCACCGGAGACCTTCTGAAAGAACAGGACCTCAT
AATCTACGATGTACCCGGGGATATCGTCTGCGGCGGGTTTGTTGCCCCTGTCAGGAAAGGCTTTGTAAACGAG
GCTTATGTCCTGACCTCGGGAGAGTACATGCCCCTTTATGCAGCAAACAATATCTGTAAAGGGCTTTCCAAAA
TCGGGATGCCTCTCAGCGGAGTTATCTGCAACTCAAGAAACGCAAGCAGGGAAGAGGAAATTGTCAGAAAGTT
TTCCGAAGAAATCGGCAGCCAGTTAATGGCTTTCATTCCAAAGAGGCAGGCAGTACAGGACTGGGAGAGGGAA
GGCTATTCCGTTATGGAGAAAGCTCCTGACTCCGATATTGCCGAAGTCTACCGCCAGCTTGGAAAAGCAATCC
TGGAAAATGAAAAAAAGGTTATGGCAGACCACCTGGGCGATGAGCGGCTGAGGGAAATGACAAAGTAA
>mba_Mbar_A0347-Ar
TTGAAAAACCAGAAGATCATAGCGATCTACGGAAAGGGCGGCATAGGTAAATCGAGTACAGCTTCAAACGTTG
CCGCTGCCTGCGCCGAGGCAGGAAAGAAGGTTATGATTATAGGCTGCGATCCTAAAAGTGACTCATCAATCAC
CCTTCTAAGAGGCAGGAGAATTCCAACCATTCTCGACCTCCTGCGTGAGGGTGTGGACATAAAGAAAGAAGAT
GTGGTCTTTGAAGGATATGCAGGCGTCAAATGCGTGGAAGCAGGCGGCCCAGAACCAGGCATAGGTTGTGCAG
GGCGAGGAATCATTGTTGCCATCCAGAAATTGAAGAGCATTTCAGGAAATCTCTTAAAAGAGCAGGATTTGAT
CATTTACGATGTGCCCGGAGACATAGTCTGCGGAGGGTTTGTCGCCCCTGTCAGGAAAGGATTCGTAAATGAA

GCTTATGTCCTGACCTCAGGAGAGTACATGCCCCTTTATGCAGCAAATAATATATGCAAAGGCTTATCGAAGA
TAGGAATGCCGCTTAGCGGGGTAATCTGTAATTCAAGAAACGTGAGCAGGGAAGAGGAAATCGTTTCGAAGTT
TTCTGAGGAGATCGGCAGCCAGCTTATGGCTTTTATCCCGAAGAGGCAGGTAGTTCAGGACTGTGAAAGAGAA
GGTTACTCGGTAATGGAAAAAGCGCCTGAATCCGATATTGCAGAGATCTACCGCAAACTCGGAAAAGCAATCC
TTGAAAACGAAAAACGAGTTACAGCCGATTCCCTTAGTGATGAACGGTTGAGGGAATTAACAAAATAA
>mbu_Mbur_1038-Ar
ATGAGCGTACAGAAAAGAATAGCTATCTATGGAAAAGGTGGCATCGGAAAATCCAGTACTGCATCCAACGTTG
CAGCCGCATGTGCCGATGAGGGATACACGGTAATGATAATTGGCTGTGACCCGAAAAGCGATTCATCCATCAC
CCTTCTTGGAGGAAAACGCATCCCGACCATCCTTGATCTTCTGCGTAAGGAAAAGGATGTGAAAGAGGAAGAT
GTCGTATTTACCGGTTACAATGGCGTGAAGTGTGTGGAAGTGGGCGGACCCGAGCCAGGTATCGGCTGTGCCG
GACGTGGGATCATCGTGGCCATCCAGACACTTAAGAAGATATCAAAAACGCTGAACGAAATGGACCTTATCAT
CTACGATGTCCCTGGAGATATCGTATGTGGTGGCTTTGTAGCACCTATACGCAAGGGACTTGTAAAGGAAGCA
TATGTCCTGACTTCCGGCGAATATATGCCCCTTTATGCAGCGAACAATATCTGCCGAGGACTGGCAAAGATAA
ACACTCCCCTCAGCGGGATCATATGTAATTCCCGCAGTGTGAGCCGTGAAAAAGAGATAGTGACCAAGTTCGC
AAGTGAGATAGGAACCGACCTTATGGCGTTCATACCAAAGGAACAGATCGTGCAGGATTGCGAAAGGGATGGT
TTCTCGGTCATGGAAAAAGCACCAGACTCCAGCATTGCACAAGTTTATCGTGACCTTGCGAAGGCCATCATGC
AACGCGATACTTCCGTTATGCCTGTTGCTCTTGACGATGAACGTCTCAGAGAGCTTACCAGATAA
>mmh_Mmah_1413-Ar
ATGAATCCGCAGAAAAGAATTGCAATCTATGGCAAAGGCGGTATCGGTAAATCAAGCACTGCATCCAATGTAG
CCGCTGCCTGTGCCGATGAGGGCTATAATGTCATGATAATCGGATGTGACCCAAAAAGCGATTCATCCATAAC
ACTCTTGGGAGGAAAACGTATCCCCACTATCCTGGACCTACTCAGAGAGGGCATGGAGATAGAAGAGGAAGGA
GACGTTGTTTTTGAGGGATACAAGGCAGTGAAATGTGTAGAGGTCGGTGGACCTGAACCGGGCATCGGTTGTG
CCGGCAGGGGGATTATTGTTGCCATCCAGAAACTCAAAAAGATATCCCAATCCATGAAAGAAATGGACCTTAT
CATTTATGATGTGCCCGGGGATATTGTCTGTGGCGGATTTGTCGCCCCGATCAGAAAAGGACTGGTCAATGAG
GCCTATGTCCTCACCTCCGGGGAATACATGCCACTGTATGCAGCCAACAACATTTGCAAGGGACTGTCCCGTA
TCAATACGAAACTTAGCGGTGTAATCTGTAACTCACGCAGCGTCAGCCGGGAAAAGGAGATAGTGAGCAAATT
TGCAAATGAGATCGGAAGTGAACTGGTGGCCTTTATACCAAAGGAACAGATCGTGCAGGACTGCGAAAGGGAT
GGTTATTCAGTGCTTGAAAAGGCAGGAGGCACAGATATTGCTGAAGTGTATCGCGCCCTTGCAAGAAATATAA
TGAGCAGTGATTGTGCAGTTGACCCCCGGGCACTGACAGATGAGCGGCTCAGGGAACTTACCCGGTAA
>mev_Metev_0555-Ar
ATGAACAATAAAACCAATGATAACAATCAGAAACAGAAACGAATAGCTTTGTATGGTAAAGGAGGCATCGGTA
AATCGAGTACAGCATCAAATGTTGCGGCTGCATGCGCTGATGAAGGATACAAAGTTATGATTATCGGATGTGA
CCCAAAAAGTGATTCGTCCATAACTTTGCTTGGTGGTAAAAGAATTCCTACAATACTTGATCTTTTGCGTGAT
GGTGAGGGTTTAAATGAAGAGGATGTTGTATTTGAGGGATACAACGGAGTTAAATGTGTAGAAGTTGGAGGTC
CAGAACCCGGTATAGGTTGTGCAGGACGCGGAATTATTGTTGCTATCAATCAGTTGAAAAGAATCTCAGATTC
CATGAAAGAAATGGATCTTATAATCTATGATGTTCCCGGAGATATAGTGTGCGGGGGATTTGTGGCACCGGTA
AGAAAGGGTATGGTGAATGAATCCTATATAATAACCTCAGGGGAATATATGCCGATGTATGCTGCAAACAATA
TATGCAGTGGTCTCTCAAAAATCAATACACCGTTAAATGGAATTGTATGTAATTCACGCGATGTCACAAATGA
GAAAGAAATAGTAGAAGAATTTGCTCATGAGATAGGAAGTGAACTTCTGTCATTCATCCCCAAAGAACAGATT
GTCCAGGATTGTGAACGCGAGGGTTATTCTGTAATGGATATAGCACCCGATTCAAAGATTGCACAGGTTTATC
GGGAACTGGCACAAAAAATAATGTCAAAAACAGAGCCAGATTCGGTTTTACCGAATTCAATGGATGATGAACA
GTTACGGGAACTTGCAAGAAAGCAAAGCAAACAAGAAACAAAATCATGTCACAACAAAAAAACGATAAGAACC
GAATAA
>mzh_Mzhil_0468-Ar
ATGAAAAATAGGAAGATCATTGCAATTTATGGAAAGGGCGGTATTGGAAAATCAAGTACCGCGTCCAATATTG
CCGCAGCATGTGCAGATGAAGGATATAAAGTAATGATCATTGGCTGTGATCCAAAGAGTGATTCTTCAATTAA
TCTGCTTGGTGGACAGCGAATTCCAACTATTATGGGGCTACTGCGTGAGAGCGTAGATATTCAGGAAGAAGAC
ATTATTTTTGAAGGGTACAATGGAGTAAAGTGTGTTGAAGTTGGTGGTCCTGAACCCGGTATTGGATGTGCAG
GAAGAGGTATTATTGTTGCAATTCAAAAACTAAAAAGTGTTTCAAAGGCCCATCAGGAAATGGATCTTATTAT
TTATGATGTGCCTGGCGATATTGTCTGTGGTGGGTTTGTAGCTCCTATCAAAAAAGGGTTTGTTAACGAAGCA
TATGTTTTGACATCAGGAGAATACATGCCACTTTATGCTGCAAACAATATTTGCAGAGGACTGAATAAAATAA
ATACTCCTCTTAGCGGAGTCATCTGCAATTCCCGTGATGTAAGCCGTGAGAGGGAAATTGTTAAAAAGTTTTC
TGAAGAGCTTGGAAGTGAACTTATAGCTTTTATACCAAAAGAGCAGATTGTACAGGACTGTGAGCGTGAAGGT

TTCTCTGTGATTGAGAAGGAACCGGACTCTAGTATTGCAGATGTTTACAGGGAACTTGCCAGAAAGATCATGT
CACAGAAAAAAGCTTCAAATCCAGACCCAATGGATGATGAAAAGTTGCGCAGCCTTACAAGGTGA
>mcj_MCON_0503-Ar
ATGTCATCTATTAAACACGTTGCGATATATGGCAAAGGCGGTATCGGCAAGTCGTGCACTGCCTCGAACATCG
CTGCTGCCTGTGCCGAGGAAGGCCACAAGGTTTTAATGGTGGGCTGCGATCCGAAGAGCGACTCAAGCATCAC
CCTCATAGGAAGGAGAATTCCCACGATGATGGACCTCATCCTCAAGGGAGGAGAGTTCAAAGAGGAGGATGTG
GTCCATCAGGGCTTCAGAGGAGTCAGGTGCATTGAGGTGGGCGGGCCCGAGCCAGGCGTGGGATGCGCGGGAC
GGGGCATAATCGTTGCCATTGATTTTTTGCGGAAGGTATCAAAAGCGATGGAGGAGGTGGATCTGGTGCTCTA
TGATGTGCCAGGGGACATCGTCTGCGGGGGATTCTCCGCTCCCATCAGGAAAGGGCTGGTGAGCCAGGCCTAC
ATCATAACCTCAGGGGAGTACATGCCCCTTTATGCGGCGAACAATATCAGCAAAGGCCTCCTGCGATTGAATA
CCCCTCTTGCAGGGGTGATATGCAACTCTCGCGAGGCAGCTGGAGAAAGAGAGCGCGAGATAGTCAACCAGTT
TGCCCAGGAGCTGAACAGCCGGATGGTGGCCTTCATTCCCAAGGAGCCGATAGTCCAGCAGTGCGAGAGGAGG
ACGGTGACTGTGATCGAGGGCGCGCCCGATTCGAAGATCGCCGCTGTCTACCGGGCCCTGGCAAGGGAGATCA
TGTCCGGCGGAGAGGCCAGGATCCCTGAACCTCTTAGCGATGAGAGGCTGAGAGAGCTCTCCCGCGATCTGGG
CTCTGAGGAGTGA
>mtp_Mthe_1178-Ar
ATGTCACCTATTAAGCATGTTGCCATCTACGGCAAGGGAGGTATTGGAAAGTCCTGCATAGCATCAAATGTGG
CGGCTGCATGCGCCGAACGCGGGGTCAGGGTCCTGGTCGTCGGATGCGACCCGAAGAGCGACTCCTCGATAAA
TCTCATAGGACGAAGGATTCCCACGATGATGGACATGATCCGGGAGGGGAAGGAGATCCGTGAGGAGGATGTC
GTCTTCACAGGATACAGAGGAGTGAGGTGCATAGAGGTCGGCGGCCCGGAGCCGGGAGTCGGATGCGCTGGCA
GGGGAATAATAGTGGCAATAGATTTCCTTAGAAAGGTCTCCAGGATAATGGATGATGTAGACCTCGTGCTATA
TGACGTTCCCGGGGATATCGTGTGTGGTGGATTCTCAGCCCCGATAAGAAAGGGTCTCGTCACGGAGGCGTAC
ATAATAACATCTGGGGAGTACATGCCTGTTTACGCTGCCAACAACATATGCAGGGGCCTGAAGCGGCTTGGGG
CAAGGCTCGCCGGGGTCGTTTGCAACTCCAGGGATGTTGAGGGGGAGAGGGAGATCGTCGATGATTTCGCCAG
GGCGATAGGTAGCAGGATGGTCGTGTTCATACCAAAAGATCCCGTTGTACAGAGGTGCGAGAGGAGAGCTGTA
ACGGTGATCGAGGGTGAGCCTGAAAGCAGCATCGCAGGGGTTTATCGCGCCCTCGCAGAGCATGTTCTGAGCT
GTACGGATGCATCGGTGCCGGAGCCGCTCGACGACGAGAGTCTGCGTGAGATAACAAGGATGTGA
>mpd_MCP_0364-Ar
ATGAAGAAGCAGCTGGCCATATACGGGAAGGGCGGCATCGGAAAGTCCTCGACGGCCTCGAACGTGGCCGCGG
CCATGGGCGAAAAGGGCATCAGGGCCATGCTCATCGGGTGCGACCCGAAGAGCGACTCGTCCATCACGCTGCT
CGGCGGAAGGCGGATGCCCACCATCATGGACACCCTGCGAAAAAAGGGCTCCATCGAGGAAGAGGACGTGGTA
TTCGAGGGCTTCAACGGGGTAAAGTGCGCCGAGGTCGGCGGCCCCGAGCCCGGCGTGGGCTGCGCCGGGAGGG
GCATCATCGTGGCCGTCCAGGCGCTGCAAAAAGTATGCGACGCCATGAAAGATTCGGACGTAATTATATACGA
CGTCCCGGGCGACATCGTGTGCGGCGGATTCGCCGTGCCCATCACGAAGGGCATGGTGAGGGAGGCGTACATC
ATCACTTCGGGCGAGTACATGCCGCTCTACGCCGCCAACAATATCTGCCGGGGCCTGAAGACGCTGAACACTC
CGCTGTCCGGCGTGATCTGCAACGAGAGGGAGGCGGAGCACGAGAGGGAGATCGTCGAAAAGTTCGCCGCCGC
CCTGGGAGTGCCCATGCTGGCGTACATACCCCGGGACAAGCTCGTGCAGAACTGCGAGCGCGCCGGCAGGAGC
GTCATCGAGGGCGCGCCGGGCTCGGAGATGGCCGGAGTATACCGGTTACTGGCCGACCGCATACTGACGGAAA
AAGATAAAAAGGTTCCCGATTCATTAGAAGACGAAGACCTGAGGAAGCTAACCCAATGA
>rci_RCIX2002-Ar
TTGAAACAGTCTACGAGCCAGAAGCAATTGGCAGTCTATGGCAAGGGCGGCATCGGCAAGTCTACTACGGCTT
CGAACATGGCCGCAGCGCTGGGGGAGATGGGGAAGAAAGTCATGCTCATCGGGTGCGATCCCAAGAGTGACTC
TTCTATTACTCTGCTCGGCGGCCGCCGCATCCCCACTATTATGGACACGGTCAGGGAGCGGAAGAATAAGATC
GATGGTATTAAAGAAGAAGACGTGGTCTTCGAAGGCTTCGGCGGCGTCAGGTGTACCGAAGTGGGCGGCCCCG
AGCCCGGCATCGGCTGTGCCGGCAGGGGCATCATTGTTGCTGTCGATGTGCTCATGAAGAATTCGACTTTCAT
GAAAGAGGCGGATGTCCTGATCTTCGACGTGCCGGGCGACATCGTATGCGGAGGCTTCGCCGTCCCCATCACC
AAAAAGATGGTCAGCGAAGCATATATTATTACTTCCGGGGAGTACATGCCCCTGTATGCGGCGAACAACATAT
GCCGGGGCCTCAACACCCTCAGGACTCCGCTCGGCGGCATCATCTGCAACGAGCGGGAGGCGGCGGACGAAAA
AGAGATCGTCGGACGGTTTGCGGACGCGCTGGGCGTGCCGCTGCTGTCATACATTCCCCGCAGCCGGATCGTC
CAGCAGTGCGAGCGGGAGGGGAAGACCGTCATAGAGGCAAGCCCTGACTCAGGGACCGCTTCAATTTACAGAG
GTCTCGCAGCGAAAGTTTTAATGGAAAAGGATAATAAGGTTCCAGAAGCATTAGAAGACGCATACCTGAGGGA
ACTGACACTATTATGA
>mla_Mlab_0165-Ar

ATGAGACAGATCGCCCTCTATGGAAAGGGAGGTATTGGAAAATCCACTACATCGGCAAATCTTTCGGCGGCAT
TTTCGGAGATGGACCTGGATGTCATGCAGATCGGGTGTGACCCGAAACACGACAGCACACGGATGCTCATGCA
CGGACGCTGGATCCCGACCGTTCTTGAGCAGATGTATGATCATAAAGAGATCAAAAGCGAGGACATCATCTAT
CAGGGATTTGGAAACATCCGCTGTGTCGAGGCTGGAGGGCCGGAGCCCGGGATCGGGTGTGCAGGCAGAGGAA
TCATCGCAACATTCCAGCTTCTGGAAAAGATGGATGCTCTCTACGGAGACGTTGTCGTATACGATGTTCTCGG
GGACGTTGTCTGCGGAGGGTTTGCGATGCCGATGCGTGACGGCTATGCGCAGGAAGTGTATCTTGTAACAAGC
GGAGAGCTGATGAGTCTCTATGCTGCAAACAATATCTGCAAAGCGATCGCGAGAATATCCGAACGAAGCACGG
CAAAATGCCGGCTTGGTGGAGTGATCTGCAATGCAAAGAATATGGATAACGAACGCGAACTTGTCGAGGAGTT
TGCAAAAAGGATCGGATCACGCCTTGTCTGTTATATTCCACGAAGCAAATCGGTCAGAGCAGCGGAGGTGAAC
TGTATGACGGTCATCGAACACGATCCAACATCGGAACAGGCGGCCGTCTACCGGGACTGTGCCAAAACCATTC
TGGAAAACACCGATCTTCGGATTCCGACCCCGCTTGCATTAGAGGAACTGGAAGAGCTTGCAAGACACTATCT
CTGA
